# Synthesis, Characterization, and Photochemistry of a Ga_2_L_3_ Coordination Cage with Dithienylethene-Catecholate Ligands

**DOI:** 10.1021/acs.inorgchem.4c03279

**Published:** 2024-10-08

**Authors:** Adrián Carbonell, Ignacio Izquierdo, David B. Guzmán Ríos, Gantulga Norjmaa, Gregori Ujaque, Antonio J. Martínez-Martínez, Uwe Pischel

**Affiliations:** † Center for Research in Sustainable Chemistry (CIQSO) and Department of Chemistry, University of Huelva, Campus El Carmen, Huelva 21071, Spain; ‡ Departament de Química and Centro de Innovación en Química Avanzada (ORFEO−CINQA), Universitat Autònoma de Barcelona, Bellaterra, Cerdanyola del Vallès, Catalonia 08193, Spain

## Abstract

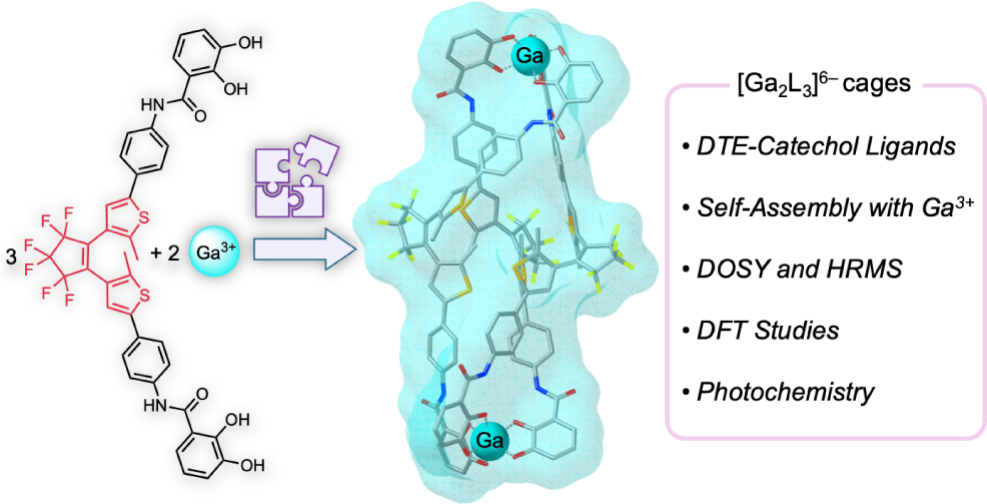

Two new photoswitchable dithienylethene (DTE)-catechol ligands, specifically designed for group 13 metal coordination, were synthesized via Suzuki coupling reactions from a dichloro-DTE building block, each with varying longitudinal extensions. The shorter DTE-catechol ligand did not efficiently assemble with Ga^3+^ metal ions; however, elongation with a phenylene-amide spacer group enabled the successful formation of a novel triply DTE-functionalized coordination [Ga_2_L_3_]^6–^ cage. This cage represents a unique example of integrating DTE photoswitches with main group metals in a supramolecular coordination framework. The [Ga_2_L_3_]^6–^ cage was thoroughly characterized by NMR spectroscopy, including DOSY hydrodynamic volumetric analyses, high-resolution mass spectrometry, computational DFT, and photochemical analyses. The DFT studies highlighted the structural integrity and dynamic interplay within the helicate and mesocate isomeric forms of the [Ga_2_L_3_]^6–^ cage upon photoswitching. While the free ligands exhibited all-photonic reversible switching at up to mM concentrations upon alternating irradiation at 365 and >495 nm, the [Ga_2_L_3_]^6–^ cage demonstrated these capabilities under dilute μM conditions, albeit with lower efficiency and fatigue resistance. This behavior highlights the intricate relationship between rigid coordination with main group metals and the flexibility of the photoswitchable DTE ligands within the [Ga_2_L_3_]^6–^ cage.

## Abstract 2

Structurally elongated dithienylethene (DTE)-catechol ligands enable the efficient self-assembly of a novel [Ga_2_L_3_]^6−^ main group metal coordination cage. This study details the nuances and impact of ligand design on the coordination assembly and photoswitching properties of supramolecular DTE-functionalized frameworks with gallium.

## Introduction

The use of light to stimulate functional systems draws attraction from the possibility of achieving spatiotemporal control. A key strategy to achieve this goal is the use of light-responsive photoswitchable functions, which find applications across diverse fields including photopharmacology,^1,2^ catalysis,^3^ materials science,^4^ molecular information processing,^5−8^ or imaging.^9−11^ Integrating these photoswitchable units into supramolecular host–guest systems enables the precise control of their properties.^12−19^

Photoswitches can be either incorporated as part of macrocyclic receptors or introduced through guest components, serving as cornerstones of innovative supramolecular designs by modulating their photoreactivity and functional properties. Examples include dithienylethenes (DTEs), azobenzenes, and spiropyrans used as guests in organic macrocycles such as cucurbiturils.^11,20−26^ Furthermore, the switching properties of these photoactive guests can be significantly altered when encapsulated in supramolecular Pd_4_L_6_ coordination cages.^27−33^ Additionally, the integration of photoswitchable units as integral parts of supramolecular receptors has garnered significant interest. For instance, azobenzene-containing ligands have been used in Zn_4_L_4_ or Pd_2_L_4_ cages to enhance the photoswitching properties through the functional modulation of the light-responsive units.^34−36^

DTEs are particularly noteworthy photoswitches for their high fatigue-resistance to all-photonic switching,^37−40^ which occurs without the need for thermally activated isomerization processes, unlike in spiropyrans or azobenzenes. Hence, DTEs have been integrated into ligands for transition metal Pd_2_L_4_ coordination cages,^41−43^ utilizing the light-induced modulation of the internal space of the cage for guest release/uptake scenarios.^44−46^ Additionally, Feringa’s overcrowded alkene molecular motors have been used in similar supramolecular Pd_2_L_4_ assemblies,^47^ though no significant light-induced changes in binding properties were observed, underscoring the unique functionality of the DTEs in these applications.

While transition metal-derived supramolecular cages have been extensively studied in the context of photoactive assemblies, examples involving main group metals are less explored. Pioneering examples of supramolecular coordination assemblies using group 13 metals, particularly Ga^3+^, were introduced by the groups of Raymond, Toste, and Bergman.^48−53^ These designs use catecholate ligands to form helical M_2_L_3_ assemblies or tetrahedral M_4_L_6_ cages.^48,49^ In this study, we introduce new catechol ligands functionalized with DTE photoswitches, a novel approach for creating light-responsive supramolecular coordination M_*n*_L_*m*_ systems with main group metals. Specifically, these DTE-catechol ligands self-assemble with Ga^3+^ metal ions into a new type of supramolecular coordination Ga_2_L_3_ cage capable of photoswitching under dilute conditions. This work not only highlights the versatility of the DTE-catechol combination for functional coordination assemblies but also sets the stage for further developments aimed at modulating the photoresponsive properties of these DTE-catecholate metal assemblies.

## Results and Discussion

### Synthesis and Characterization of Dithienylethene Ligand **3** and Attempted Self-Assembly with Ga^3+^ Ions

**Scheme 1:**
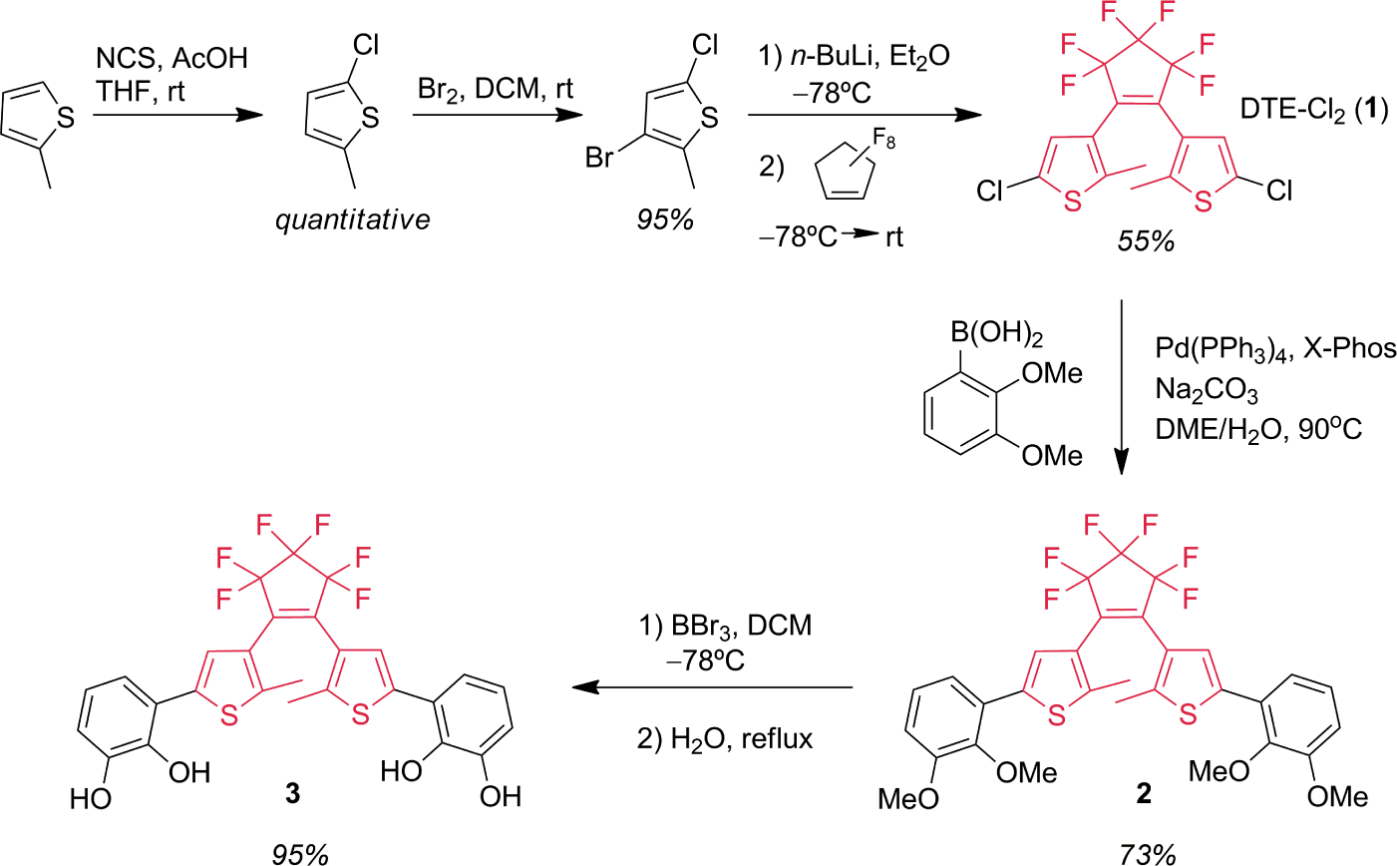
Synthetic Route for the Preparation of the DTE-Catechol Derivatives **2** and **3** from **1**

The DTE core, DTE-Cl_2_**1** (Scheme 1), was synthesized from 3-bromo-5-chloro-2-methylthiophene by bromine–lithium exchange with *n*-BuLi, followed by reaction with octafluoro cyclopentene, producing **1** in 55% yield.^54^ Subsequently, the intermediate synthon **1** was then coupled with commercially available 2,3-dimethoxyphenylboronic acid through a Pd(PPh_3_)_4_-catalyzed Suzuki reaction,^55^ leading to the formation of product **2** in 73% yield. Finally, the demethylation of **2** with BBr_3_ produced **3** in 95% yield.

**Figure 1:**
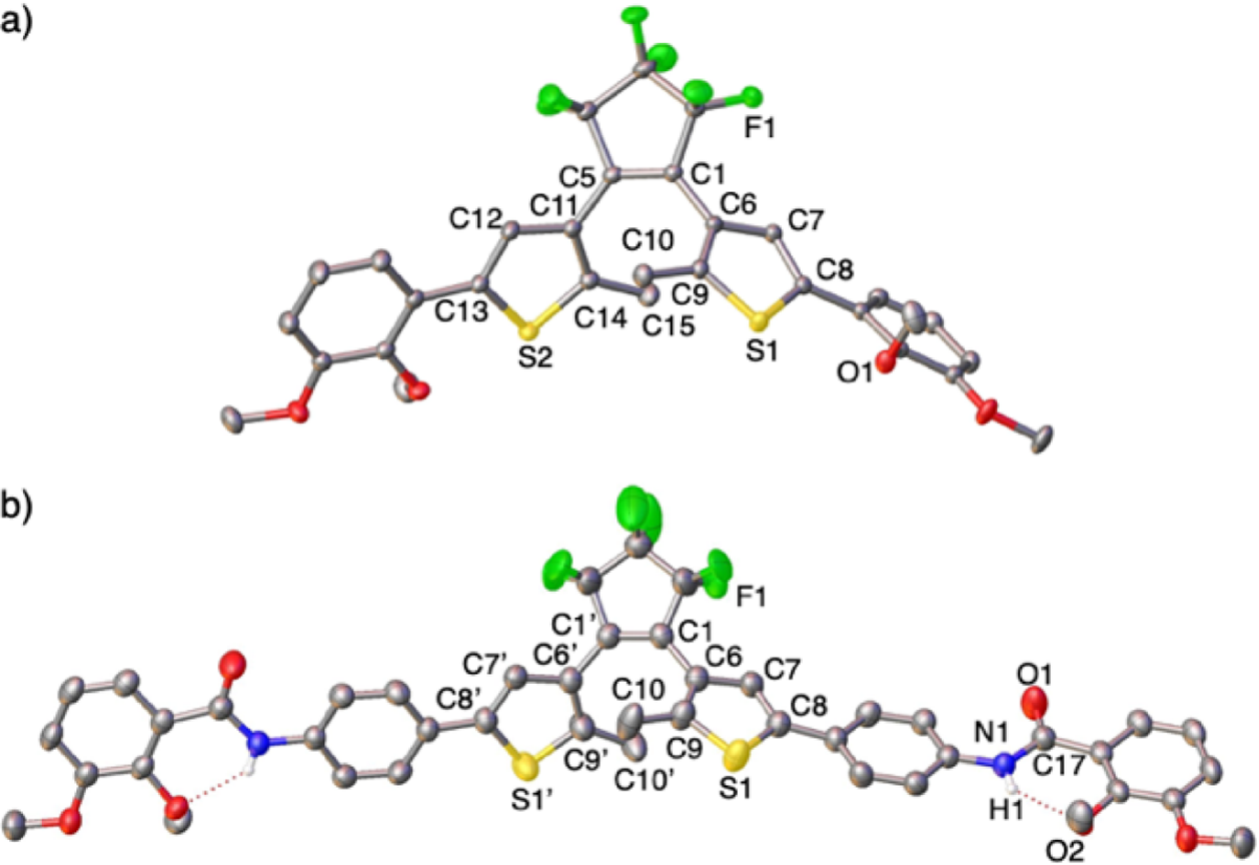
a) Molecular structures of the DTE-catechol derivatives **2** (CCDC 2364246) and b) **6** (CCDC 2364247). Hydrogen atoms, except for those in the NH groups of **6**, are omitted for clarity. Selected bond lengths (Å) and angles (deg): for **1**, C1–C5 1.351(2), C1–C6 1.466(2), C5–C11 1.466(2), C6–C7 1.430(2), C6–C9 1.376(2), C7–C8 1.364(2), C9–C10 1.500(2), C11–C12 1.425(2), C11–C14 1.377(2), C12–C13 1.367(2), C14–C15 1.500(3), S1–C8 1.7364(18), S1–C9 1.7180(18), S2–C13 1.7384(17), S2–C14 1.7166(18); torsion DTE angles C1–C5–C11–C14 45.5(3), C5–C1–C6–C9 52.8(3); for **6**, S1–C8 1.737(4), S1–C9 1.727(4), N1–C14 1.411(5), N1–C17 1.352(5), N1–H1 0.75(5), O1–H1 2.11(5), O1–C17 1.220(5), C1–C1’ 1.345(8), C1–C6 1.474(6), C6–C7 1.428(5), C6–C9 1.368(6), C8–C7 1.354(6), C9–C10 1.509(6); torsion DTE angles C1’–C1–C6–C9 and C1–C1’–C6’–C9’ 42.2(8). The symmetry transformation used to generate equivalent atoms labeled with (’) is 1/2–*x*, *y*, 1–*z*.

Compounds **2** and **3** were fully characterized by ^1^H, ^13^C, and ^19^F NMR spectroscopy, as well as by high-resolution mass spectrometry (HRMS), which unambiguously confirmed their chemical identities (see Supporting Information). In addition, colorless block crystals of **2**, suitable for X-ray diffraction analysis, were obtained by the diffusion of *n*-octane into a 1,2-dichloroethane solution of **2**. Compound **2** crystallized in the triclinic *p*-1 space group with two independent molecules within the asymmetric unit; however, for the sake of simplicity, one molecule was selected for discussion (Figure 1a). The structure of **2** features two *O*-methylated catechol groups bridged by a fluorinated DTE core. The DTE moiety adopts an antiparallel conformation between both methylated thiophene heterocycles, characterized by torsion angles of 45.5(3)° and 52.8(3)° (C1–C5–C11–C14 and C5–C1–C6–C9, respectively; refer to Figure 1). This antiparallel arrangement influences the crystal packing, preventing π···π intermolecular interactions within the crystal lattice (see Supporting Information). Furthermore, the molecular structure of **2** is characterized by alternating double C=C bonds within the open configuration of the DTE skeleton (1.351(2), 1.376(2), and 1.377(2) Å for C1–C5, C6–C9, and C11–C14, respectively), as seen in similar DTE derivatives.^56^

**Scheme 2:**
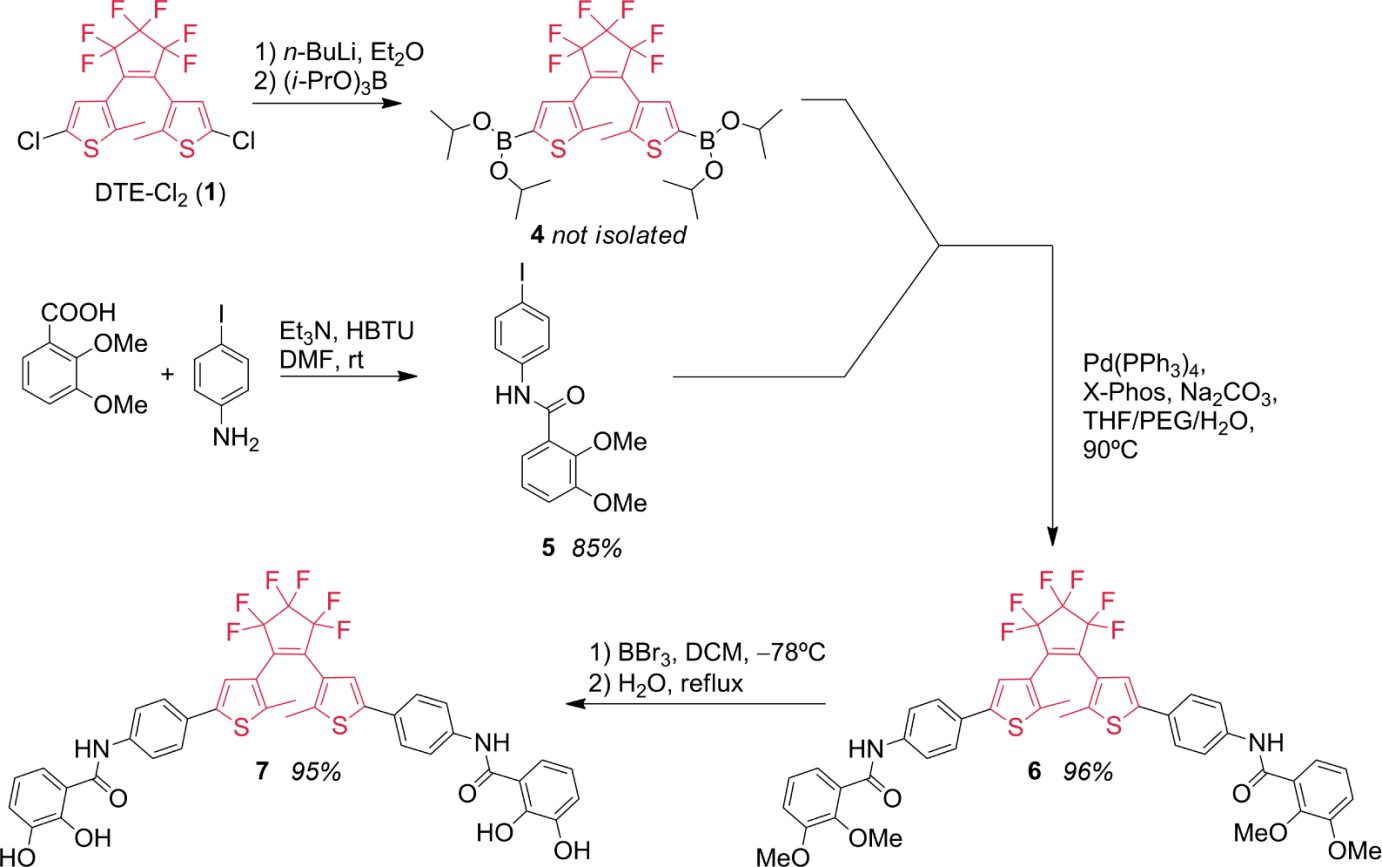
Synthesis of the DTE Derivatives **6** and **7**

However, despite the X-ray crystal structure of *O*-methylated derivative **2** showing the essential linear disposition of the catechol groups prime for creating supramolecular coordination entities, deprotonation of the bis-catechol ligand **3** with KOH in the presence of Ga(acac)_3_ (acac: acetylacetonate) did not lead to the formation of an identifiable product via coordination to Ga^3+^ metal centers. Instead, a complex reaction mixture of extremely reduced solubility was obtained, suggesting polymerization of deprotonated ligand **3**^4–^ through coordination to Ga^3+^ and K^+^ metal ions. Further attempts to assemble ligand **3** in its closed form also did not result in a well-defined structure. Additionally, efforts to assemble **3** in the presence of a small-size ammonium salt, specifically Me_4_NBr, which has been shown to sustain the formation of supramolecular cages through template–guest effects,^57,58^ also failed to produce an identifiable coordination assembly. This outcome can be attributed to the relatively small size of ligand **3**, which is insufficient to form a structurally well-defined discrete monomeric assembly of the type M_*n*_L_*m*_. To overcome this limitation, we synthetically elongated DTE-catechol ligand **3** by introducing a phenylene C_6_H_4_ ring at each side of the DTE core. This strategy was successfully implemented by using bridging amide CONH groups on the catechols, leading to elongated ligand **7** (Scheme 2). Introducing these CONH groups in **7** not only extends the ligand but also adds flexibility, which could facilitate conformational adaptation and potentially aid the self-assembly process. Notably, the bridging amide groups have previously been reported to be compatible with the formation of supramolecular M_2_L_3_ and M_4_L_6_ entities with Ga^3+^ metal ions.^48,49^

### Synthesis and Characterization of the Elongated Dithienylethene Ligand **7**

For the preparation of ligand **7**, the catechol branch **5** was first synthesized from 2,3-dimethoxybenzoic acid and 4-iodoaniline via an HBTU-promoted amide coupling (85% yield, Scheme 2). The catechol-containing unit **5** was then coupled with DTE-Cl_2_ core **1**, which was first transformed into the corresponding borylated DTE **4** by reaction with B(O*i*-Pr)_3_. The borylated DTE **4** was reacted in situ with **5** in a Pd(PPh_3_)_4_-catalyzed Suzuki reaction^59^ to give the methylated derivative **6** (96% yield). Finally, **6** underwent demethylation with BBr_3_, yielding the bis-catecholate extended ligand **7** in 95% yield.

Both the methylated derivative **6** and the final ligand **7** were characterized by ^1^H, ^13^C, and ^19^F NMR spectroscopy, as well as by high-resolution mass spectrometry (HRMS); see Supporting Information. In particular, the ^1^H NMR resonance corresponding to the NH groups in **7** appears at 10.57 ppm, which was clearly identified by a cross-peak with the ^13^C signal of the C=O group observed in the HMBC spectrum. Additionally, the two chemically different OH groups in **7** resonate as broad singlets at 9.56 and 12.10 ppm, with the latter being deshielded due to the internal hydrogen bonding with the amide C=O group.

Crystals suitable for an X-ray diffraction study of **6** (Figure 1b) were obtained by slow diffusion of the same solvent mixture (*n*-octane and 1,2-dichloroethane) used for **2**. Compound **6** crystallized in the monoclinic *I*2/a space group. The molecular structure of **6** reveals an elongated ligand with phenylene-amide C_6_H_4_NHCO moieties flanking both sides of the DTE core. The structure exhibits internal hydrogen bonding between the amide and the neighboring OMe group (N1–H1 0.75(5) Å; O1–H(1) 2.11(5) Å), fostering a nearly coplanar arrangement between the methylated catechol outer ring and the phenylene C_6_H_4_ ring. This alignment supports electronic delocalization along the alternated thiophene/phenylene/amide/methylated-catechol sequence on both sides of the DTE core. This structural feature effectively elongates the DTE ligand **6** by *ca*. 13 Å compared to **2** (O···O interatomic distance measured for the most distal oxygen atoms: 14.8 Å for **2** and 27.9 Å for **6**). Akin to **2**, the DTE core in **6** maintains an antiparallel conformation, marked by a unique torsion angle of 42.2 (5)° (C1’–C1–C6–C9 and C1–C1’–C6’–C9’; Figure 1b), slightly smaller than those found in **2** (45.5(3) and 52.8(3)°). The other bond lengths in the DTE core of **6** remain comparable to those observed in **2** (see the caption of Figure 1).

### Preparation and Characterization of the Supramolecular Coordination M_2_L_3_ Cage

**Figure 2:**
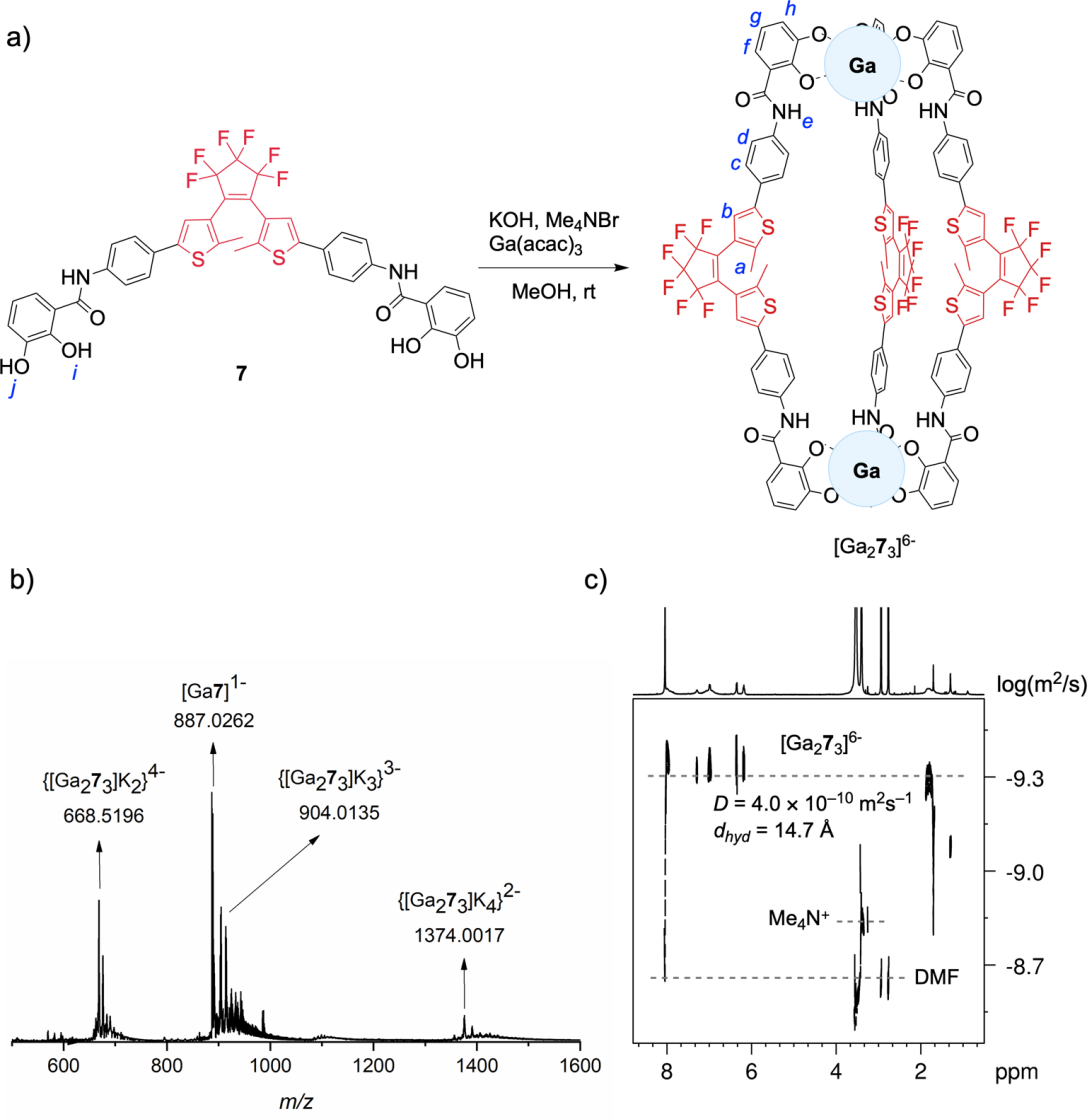
a) Synthesis of the supramolecular coordination [Ga_2_**7**_3_]^6–^ cage. b) High-resolution mass spectrum of the supramolecular [Ga_2_**7**_3_]^6–^ cage with various numbers of K^+^ counterions. c) DOSY spectrum (500 MHz, 298 K) of the DMF-*d*_7_ solution of the cage [Ga_2_**7**_3_]^6–^ (*D* = 4.0 × 10^–10^ m^2^ s^–1^; *d*_hyd_ (hydrodynamic diameter) = 14.7 Å).

**Figure 3:**
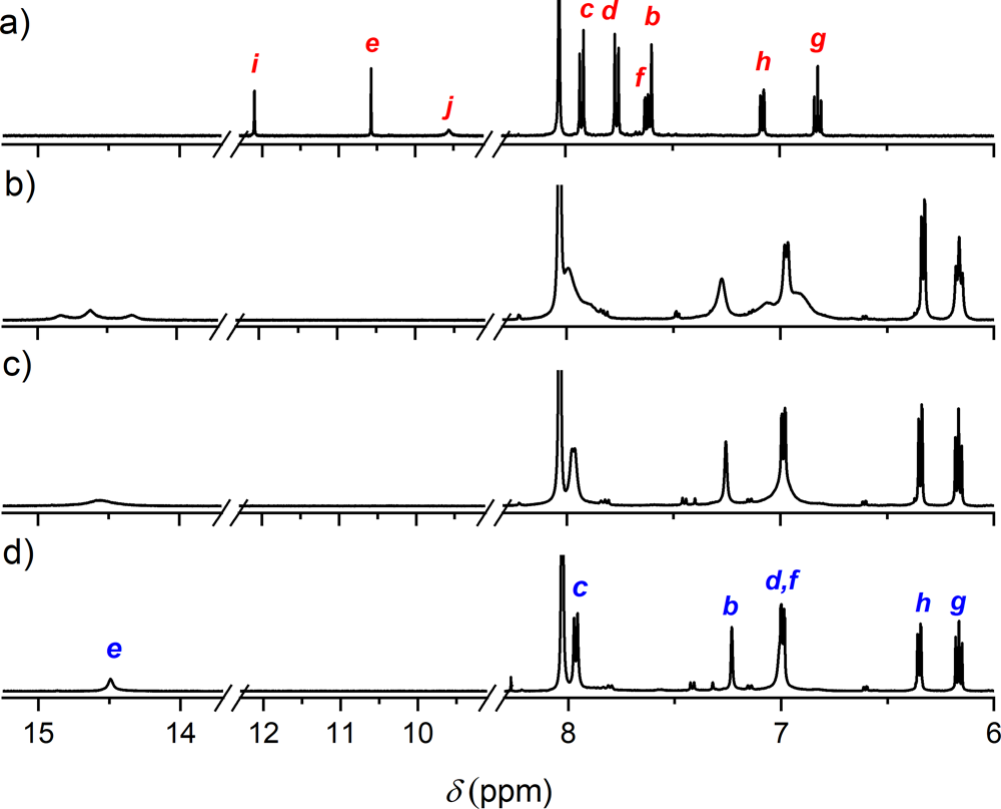
^1^H NMR (500 MHz, DMF-*d*_7_) spectra of the free ligand **7** in its opened form at a) 298 K and the [Ga_2_**7**_3_]^6–^ cage at variable temperatures, b) 298, c) 323, and d) 348 K. The letters correspond to the protons highlighted in Figure 2. The small peaks in the aromatic region are attributed to traces of the closed form of ligand **7**, resulting from photoisomerization in ambient light. The full-range NMR spectra can be found in the Supporting Information.

Following the synthesis of ligand **7**, we explored its potential for self-assembly into discrete supramolecular coordination arrays with Ga^3+^ metal ions; see Figure 2a. For this purpose, ligand **7**, in its ring-opened form, was first deprotonated with KOH and then allowed to assemble with Ga^3+^, provided in the form of Ga(acac)_3_, in the presence of Me_4_NBr in methanol.^48,49^ The latter reduces the solubility of the product, resulting in the precipitation of a supramolecular Ga_2_L_3_ coordination assembly by the addition of diethyl ether to the mixture. This synthetic protocol yields a coordination assembly carrying a charge of 6–, [Ga_2_**7**_3_]^6–^, with four K^+^ and two Me_4_N^+^ serving as counterions, resulting in [Ga_2_**7**_3_][K_4_(Me_4_N)_2_]. The M_2_L_3_ stoichiometry of the cage was confirmed by HRMS, and ^1^H NMR spectroscopy confirmed the presence of two Me_4_N^+^ counterions (see Figures 2b and 3, and Supporting Information). Similarly to what was observed for ligand **3**, attempts to assemble ligand **7** in its closed form did not result in a well-defined structure. Instead, a complicated reaction mixture was obtained, where no identifiable [Ga_2_**7**_3_]^6–^ cage was observed, suggesting a preference for the opened form of **7** under these conditions.

In the HRMS data (Figure 2b), several signals are indicative of the formation of the supramolecular anionic [Ga_2_**7**_3_]^6–^ cage with K^+^ counterions. Particularly, peaks at *m*/*z* 668.5196, 904.3467, and 1376.0017, corresponding to [Ga_2_**7**_3_(K)_4_]^2–^, [Ga_2_**7**_3_(K)_3_]^3–^, and [Ga_2_**7**_3_(K)_2_]^4–^, respectively, show a distribution of the supramolecular system [Ga_2_**7**_3_]^6–^ by ion-pairing association with diverse contents of K^+^ in the gas ionization phase. For all of the identified peaks, the experimentally observed isotope pattern coincides with the theoretically expected one, highlighting the presence of a proportion of two Ga^3+^ ions to three molecules of ligand **7** within the structure of [Ga_2_**7**_3_]^6–^; see Supporting Information.

**Figure 4:**
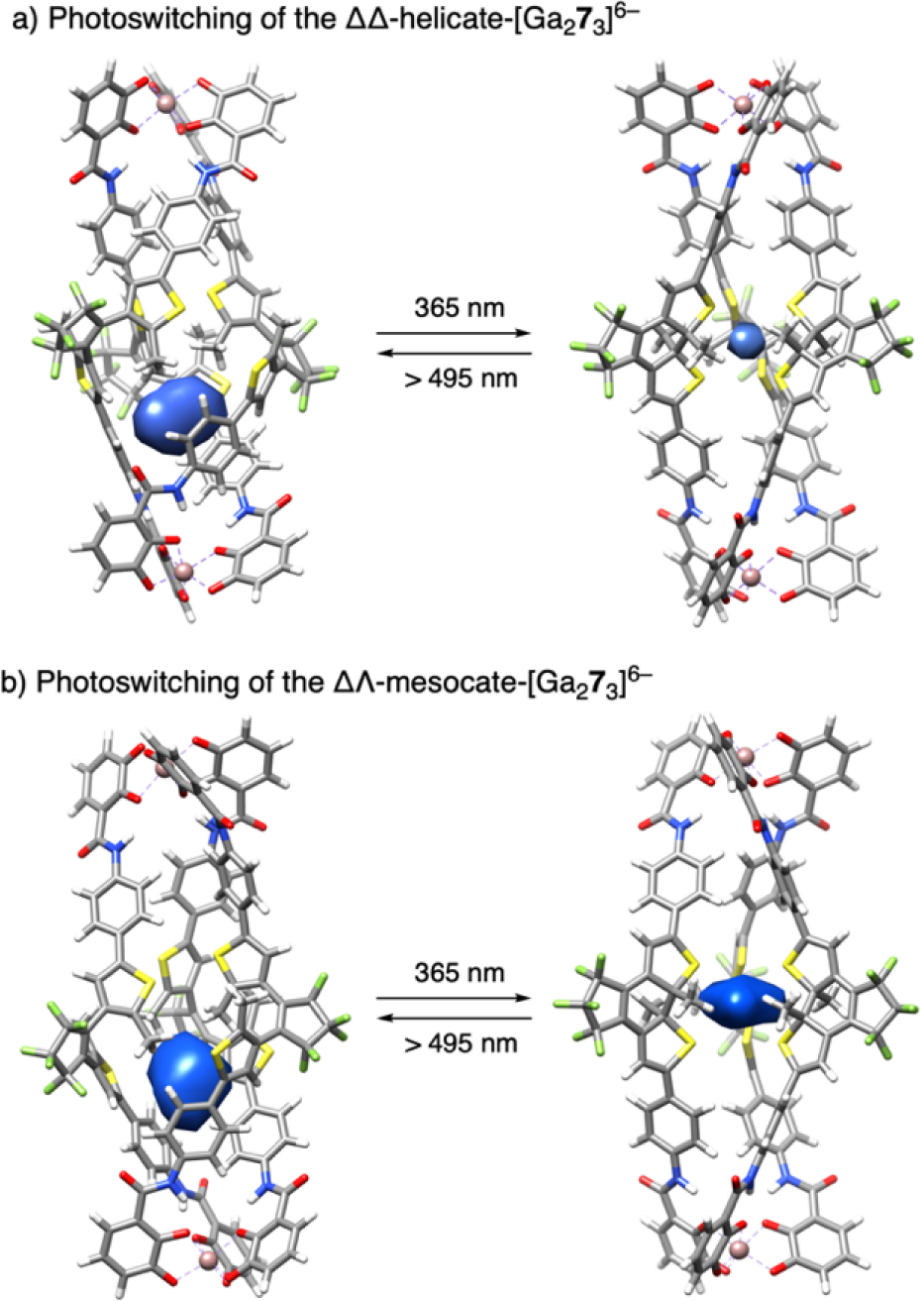
a) Photoswitching showing the optimized geometries of the lowest-energy conformers in DMF of the ΔΔ-helicate-[Ga_2_**7**_3_]^6–^ cage with the DTEs in open (left, *V* = 30 Å^3^) and closed (right, *V* = 3 Å^3^) forms. b) ΔΛ-mesocate-[Ga_2_**7**_3_]^6–^ cage with the DTEs in open (*V* = 32 Å^3^) and closed forms (*V* = 17 Å^3^). The internal cavity volumes (blue voids) were calculated using the SURFNET program^63^ implemented in the UCSF Chimera software^64^ package with a grid interval spacing of 1 Å.

The ^1^H NMR spectrum of the [Ga_2_**7**_3_][K_4_(Me_4_N)_2_] assembly in the DMF-*d*_7_ system shows broad signals at 298 K. However, upon heating to 348 K, the signals become significantly sharper; see Figure 3. The resonance signal of the NH proton provides insightful information. At 298 K, the NH signal manifests in three resonances between 14 and 15 ppm, likely corresponding to different conformational situations of DTE ligands within the [Ga_2_**7**_3_]^6–^ system. However, at 323 K, these signals coalesce into one broad signal at about 14.5 ppm. On heating further to 348 K, the NH signal sharpens even further. The chemically inequivalent ligand environments observed at 298 K suggest that the [Ga_2_**7**_3_]^6–^ assembly is desymmetrized at this temperature, potentially due to the coexistence of both mesocate and helicate isomers (see the DFT calculations section). Additionally, these isomers can adopt different conformational orientations of the Me groups on the DTE cores (parallel and antiparallel), which could further add to the desymmetrization. As the temperature increases, the NMR signals simplify, indicating dynamic averaging or the prevalence of a single thermodynamically stable isomer. Upon cooling back to 298 K, the original mixture reappears in the NMR spectrum, suggesting a reversible equilibrium between the isomers. This behavior is consistent with the interconversion of mesocate and helicate forms, as observed in related supramolecular coordination arrays.^60^ The significant deshielding of the NH protons, as compared to the situation in the free ligand **7** (see above), points to a prominent location inside the [Ga_2_**7**_3_]^6–^ cage and presumably interactions with the catechol O atoms, as seen in the molecular structure of **7** (Figure 1b). This is further corroborated by the DFT-optimized geometries of the [Ga_2_**7**_3_]^6–^ assembly (see Figure 4). In agreement with the catecholate-Ga^3+^ complexation, the aromatic CH (*f*, *g*, and *h*; see Figure 2a) signals of the catechol are significantly shifted upfield by *ca*. 0.7 ppm; see signal assignments in Figure 3. Importantly, it appears that the Me_4_N^+^ ions (observed at 3.33 ppm) are not encapsulated within the [Ga_2_**7**_3_]^6–^ cage, as no typically upfield-shifted Me_4_N^+^ proton signals were noted.

The diffusion coefficient *D* of free ligand **7** and the [Ga_2_**7**_3_]^6–^ cage was determined by DOSY NMR (500 MHz, DMF-*d*_7_) spectroscopy (see Supporting Information). The free ligand **7** exhibits a *D* value of 7.4 × 10^–10^ m^2^ s^–1^, which, as expected, is significantly higher than that of the coordination [Ga_2_**7**_3_]^6–^ cage (*D* = 4.0 × 10^–10^ m^2^ s^–1^, Figure 2c). Using the Einstein–Stokes equation, these values translate into hydrodynamic diameters (*d*_hyd_) of approximately 7.8 and 14.7 Å for **7** and [Ga_2_**7**_3_]^6–^, respectively. Interestingly, the experimental *d*_hyd_ of 14.7 Å aligns well with the rod-like shape of the [Ga_2_**7**_3_]^6–^ cage, as observed in the computed supramolecular geometries (see below). We estimated the hydrodynamic diameter based on the computed geometric parameters of the cage in the all-open DTE form to be approximately 15.5 Å (see Supporting Information). This close agreement between the experimental and computed diameters reinforces the structural integrity of the [Ga_2_**7**_3_]^6–^ cage in solution.

### Density Functional Theory (DFT) Calculations

Despite our extensive efforts to obtain a crystal structure of the supramolecular cage, we were not successful. With the intention to obtain more detailed structural insights into the [Ga_2_**7**_3_]^6–^ assembly, a computational study was performed. First, a conformation study for the two diastereomeric complexes (ΔΔ and ΔΛ configurations of the two Ga^3+^ coordination spheres) was performed. Then, the lowest-energy conformers were fully optimized in the gas phase, and the lowest-energy conformer was further optimized in DMF. The geometries in solvent for the open and closed forms of the two isomeric [Ga_2_**7**_3_]^6–^ complexes (ΔΔ and ΔΛ; see Figure 4) are very similar to those in the gas phase. The calculations show that the ΔΔ assembly is lower in energy than ΔΛ in both DMF and the gas phase, with a larger difference in solvent (see Supporting Information). Moreover, the ΛΛ assemblies (open and closed forms) were also optimized, showing that they have the same energy as their ΔΔ enantiomers. The computed structures reveal that, with the open configuration of the DTE ligand **7**, the ΔΔ- and ΛΛ-[Ga_2_**7**_3_]^6–^ cages adopt chiral helicate conformations, while the ΔΛ-[Ga_2_**7**_3_]^6–^ cage is achiral, forming a mesocate (see Figure 4 and Supporting Information). This chiral and achiral distinction is consistent with typical M_2_L_3_ systems^61,62^ and is primarily driven by the coordination geometry of the Ga^3+^ metal centers. ΔΔ- and ΛΛ-helicates are formed when the DTE ligands twist around a central axis, creating chiral assemblies that match the coordination preferences of the Ga^3+^ metal centers. This results in either ΔΔ or ΛΛ enantiomers, thus maximizing structural stabilization through optimal metal–ligand interactions. In contrast, the unique ΔΛ-mesocate configuration does not require such a twist, leading to an achiral structure. This occurs with the DTE ligand and Ga^3+^ metal center assembly with unmatching Δ and Λ coordinations. Interestingly, helicate and mesocate configurations are retained between open and closed forms of ligand **7** within the [Ga_2_**7**_3_]^6–^ cage (see Figure 4 and Supporting Information). The alignment and coordination environments of the DTE ligands and Ga^3+^ metal centers play a crucial role in determining whether a helicate or mesocate structure will form.

Besides these stereochemical implications, the optimized structures also provided interesting insights into the structural changes upon photoswitching. In Figure 4a, the inner volume of the [Ga_2_**7**_3_]^6–^ cage as ΔΔ enantiomer is indicated. For the opened form of the DTE, an inner cavity with a volume of 30 Å^3^ and a decentralized location is observable. The photoinduced DTE ring closing leads to a symmetrization of the geometry, resulting in a significant volume reduction (3 Å^3^) and a central location of the inner cavity space. Similar observations, confirming these trends, are made for the ΔΛ diastereomer (see Figure 4b).

### Photoswitching

**Scheme 3:**
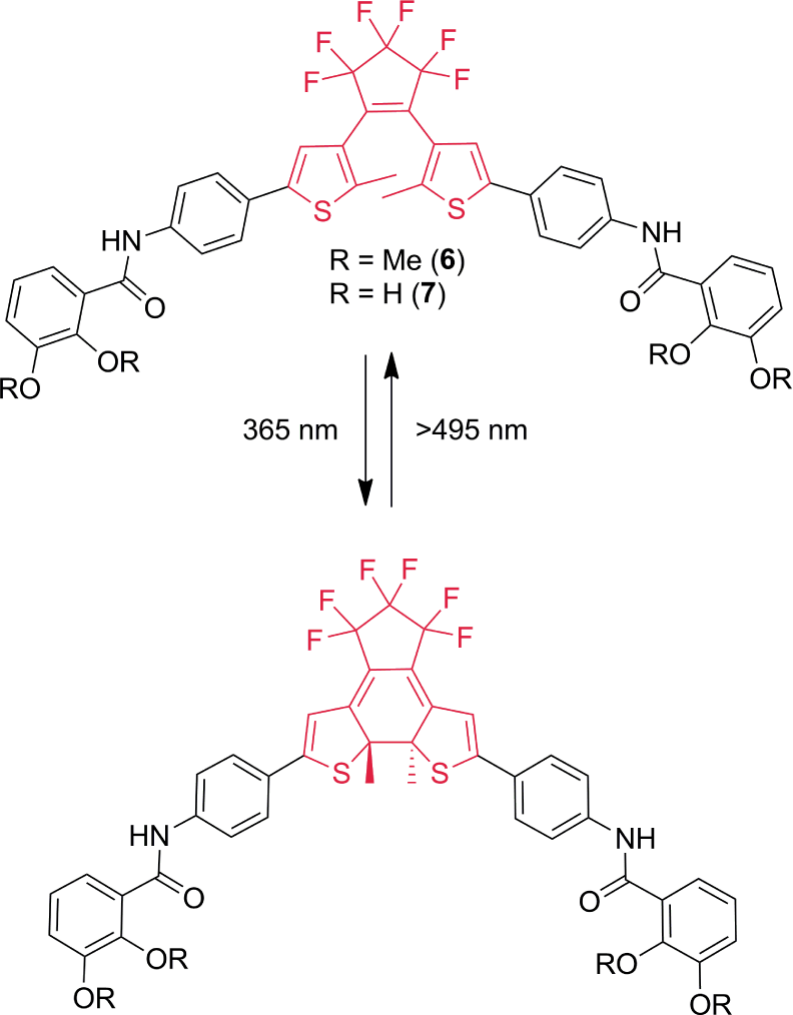
Switching of DTE Derivatives, Exemplified by **6** and **7**

**Figure 5:**
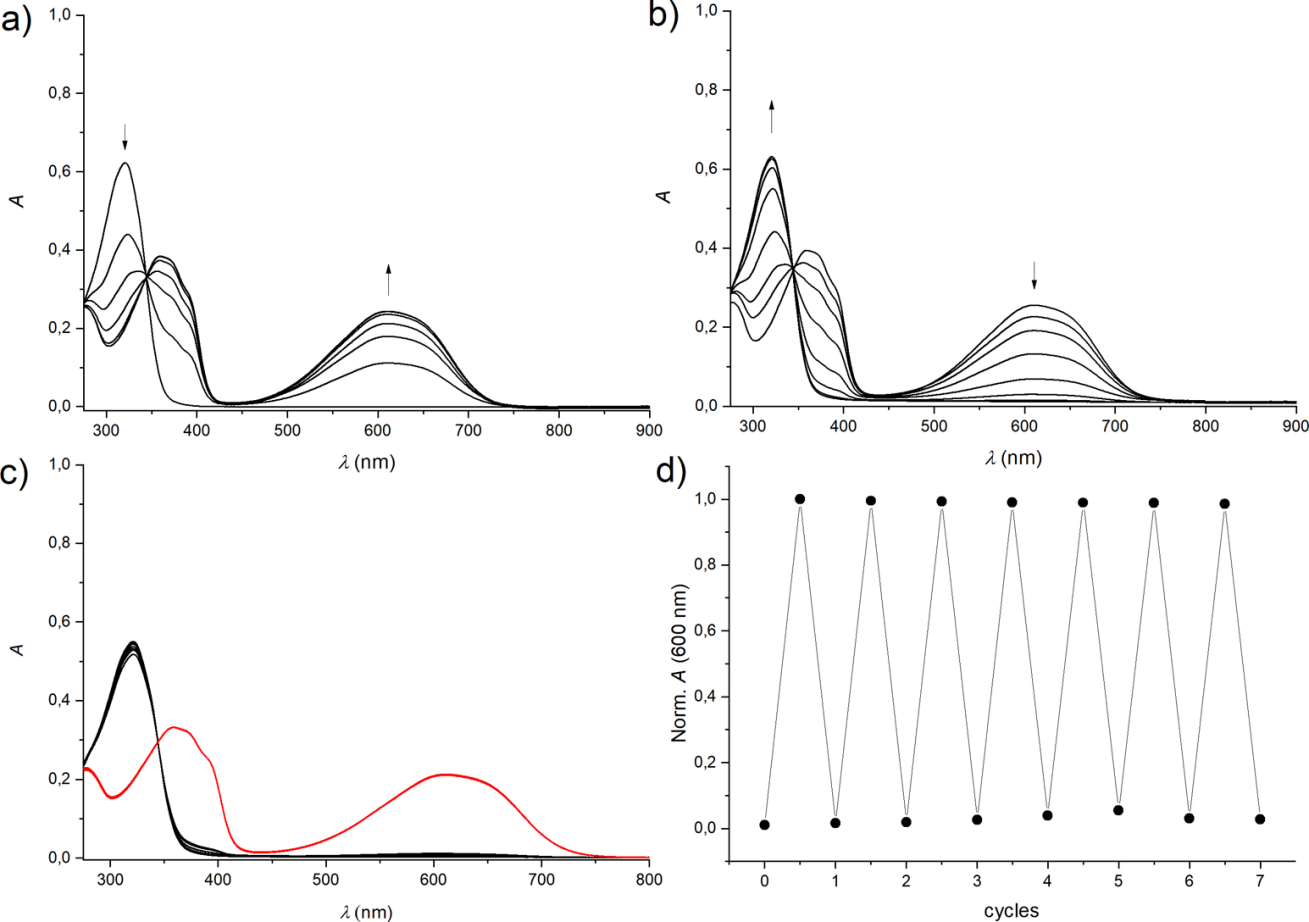
Photoswitching of DTE **6** (10 μM) in DMF. a) UV/vis absorption spectra upon irradiation at 365 nm (up to 140 s) of the ring-opened form, b) UV/vis absorption spectra upon irradiation at >495 nm (up to 240 s) of the ring-closed form, c) UV/vis absorption spectra upon alternating irradiation at 365 nm (red spectra) and >495 nm (black spectra), and d) cycling with observation of the absorbance at 600 nm of the ring-closed isomer.

**Figure 6:**
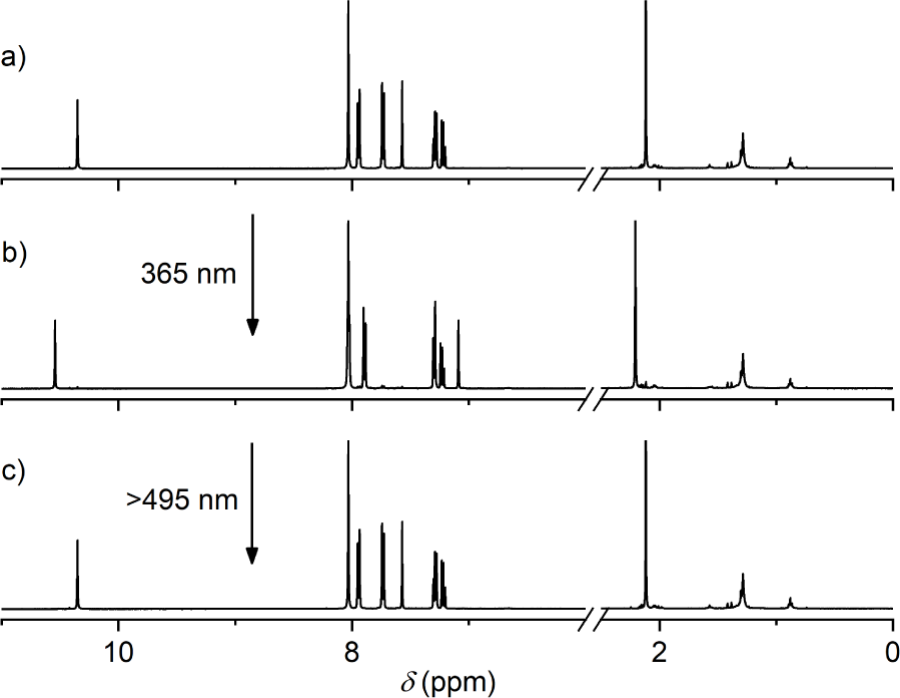
Partial ^1^H NMR spectra (500 MHz, DMF-*d*_7_) of DTE **6** (1 mM). a) Initial spectrum of the ring-opened isomer **6**o, b) the spectrum upon irradiation at 365 nm until reaching the photostationary state (corresponding in this case to 100% ring-closed isomer **6**c), and c) the spectrum upon irradiation of **6**c with >495 nm light, yielding back 100% of the ring-opened form **6**o.

The photochemical properties of free DTE ligand **7**, its methylated derivative **6** (Scheme 3), and the supramolecular [Ga_2_**7**_3_]^6–^ cage were investigated in *N*,*N*-dimethylformamide (DMF). The UV/vis absorption spectra of the reversible switching of methyl derivative **6** are shown in Figure 5. The colorless ring-opened form **6**o has an absorption maximum at 321 nm. Irradiation with 365 nm light yields the blue-colored ring-closed form **6**c, which features a prominent long-wavelength absorption band with a maximum at 611 nm (see Figure 5a). A well-defined isosbestic point at 343 nm is observed, which hints at the uniformity of the 6π-electrocyclic photoreaction. The inverse process is triggered by irradiation at >495 nm (long-pass filter), reverting the switch to **6**o; see Figure 5b. The switch can be toggled between both isomeric forms with high fatigue resistance, as practically no loss in performance is seen after seven UV/vis irradiation cycles; see Figure 5c,d. As determined from ^1^H NMR measurements (see Figure 6), the photostationary state distribution is 100% for the corresponding pure forms for the ring-closing and the ring-opening processes. The quantum yield for the ring-closing process (Φ_o→c_) was determined as 0.43, while the ring-opening process was far less efficient (Φ_c→o_ = 0.0012; irradiation at λ = 590 nm). This is in agreement with the general observations of the photoswitching of DTE derivatives.^37^

We then turned our attention to ligand **7**. Similar spectral observations as for derivative **6** were made for the switching between the ring-opened (λ_abs,max_ = 328 nm) and ring-closed form (λ_abs,max_ = 625 nm); see Supporting Information. However, the conversion from the opened to the closed isomer is *ca*. 7 times less efficient than that for **6**, i.e., Φ_o→c_ = 0.06. The close-to-open ring conversion is characterized by a quantum yield similar to that measured for **6**; Φ_c→o_ = 0.0023 (irradiation at λ = 590 nm). The photostationary state distribution points to a quantitative conversion (100%) for each of the switching directions (**7**o → **7**c and **7**c → **7**o) and the fatigue resistance is comparable to that of **6** (see Supporting Information).

**Figure 7:**
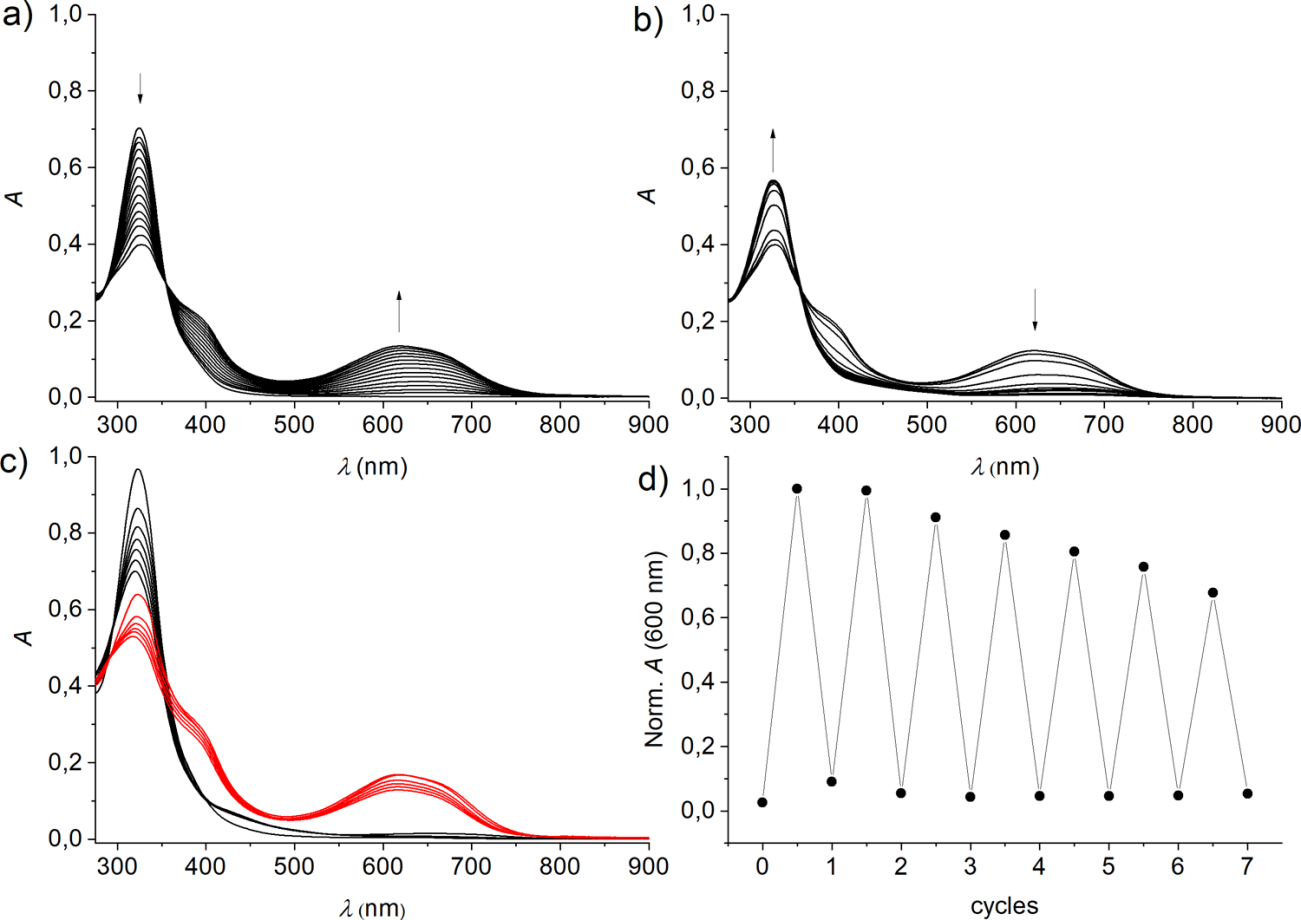
Photoswitching of the [Ga_2_**7**_3_]^6–^ cage (*ca*. 3 μM) in DMF. a) UV/vis absorption spectra upon irradiation at 365 nm (up to 1800 s) of the ring-opened form, b) UV/vis absorption spectra upon irradiation at >495 nm (up to 3400 s) of the ring-closed form, c) UV/vis absorption spectra upon alternating irradiation at 365 nm (red spectra) and >495 nm (black spectra), and d) cycling with observation of the absorbance at 600 nm of the ring-closed isomer.

Finally, the photoswitching of the [Ga_2_**7**_3_]^6–^ cage was investigated; see Figure 7. At micromolar concentration (*ca*. 3 μM), the irradiation with 365 nm light yields the typical blue colorization of the solution with the concomitant growth of an absorption band with a maximum at 618 nm, pointing to the formation of the ring-closed form. The photoreaction is accompanied by the observation of an isosbestic point at 355 nm. The irradiation of the colored solution with >495 nm light yields back the colorless ring-opened form, with the spectra showing the same isosbestic point.

However, the ring-closing photoswitching of the DTE units, when integrated into the cage structure, is much less efficient than that observed for the free ligand. Despite the observation of an isosbestic point (355 nm) for the switching of a freshly prepared solution, this feature is lost upon repeated cycling between the opened and closed forms (see Figure 7c). Hence, notable fatigue is evident, and after six cycles, the absorbance of the closed form reaches just 70% of the initial signal in the first cycle; see Figure 7d. An instructive comparison is the ratio of the absorbance at the maximum of the opened form versus the closed form for the cage and ligand **7** (*A*_324_/*A*_618_ for [Ga_2_**7**_3_]^6–^ and *A*_328_/*A*_625_ for **7**) in the photostationary state. This ratio amounts to 5.2 for the cage and only 2.4 for ligand **7**. Notably, the molar absorption coefficients of the DTE chromophore in the ligand and in the cage are not significantly altered, and the photostationary state composition of the ligand **7** is that of the pure closed isomer; see above. Hence, it can be concluded that the ring-closing is only *ca*. 50% as efficient for the cage as for ligand **7** itself. Considering the reduced fatigue resistance, it is reasonable to assume that the metal centers are not merely innocent bystanders but actively interfere with the excited state of the DTE, potentially through processes like photoinduced electron transfer (PeT). Calculations using the Rehm–Weller equation (Δ*G* = *E*_ox_ – *E*_red_ – E* + *C*)^65^ suggest that PeT between the DTE and Ga^3+^ centers is thermodynamically feasible, with an estimated Δ*G* of −0.98 eV. This supports the possibility of such quenching interactions (*E*_ox_ = 1.46 V for DTE,^39^*E*_red_ = −1.40 V for Ga(ClO_4_)_3_,^66^E* = 3.78 eV for the opened form of **7**, and the Coulomb term *C* = −0.06 eV; all redox potentials vs SCE in acetonitrile). In addition, it should be considered that some of the switch units in their opened form are fixed in their parallel conformation, which, as opposed to the antiparallel conformation, is photochemically inactive for producing 6π electrocyclization. At higher concentrations (*ca*. 1 mM), as used for the NMR monitoring of the photochemical processes of the cage, no noticeable photoswitching seems to be going on. Instead, an irreversible process applies to UV-light irradiation that cannot be inverted by visible light; see Supporting Information. The observed concentration dependence of the photochemistry of the cage points to the additional involvement of intermolecular quenching, which is naturally more noticeable in the mM range (NMR experiment) than at μM concentration (UV/vis experiment).

## Conclusions

This study demonstrates the successful integration of dithienylethene (DTE) photoswitches with catechol ligands to engineer novel coordination cages involving group 13 metals, specifically Ga^3+^. The new DTE-catechol ligands, designed with varying longitudinal extensions, were optimized for metal coordination, leading to the formation of a novel [Ga_2_L_3_]^6–^ metallo-supramolecular cage when elongated with a phenylene-amide spacer group. This cage represents a unique example of a supramolecular coordination framework that combines photoswitchable DTE ligands with main group metals. Characterization through NMR, DOSY, HRMS, and photochemical analyses, supported by computational DFT studies, confirmed the structural integrity and dynamic interplay of the helicate and mesocate isomeric forms within the [Ga_2_L^3^]^6–^ cage during photoswitching. Photoswitching studies showed that while the free DTE-catechol ligands demonstrate robust all-photonic reversible switching at up to mM concentrations, the [Ga_2_L_3_]^6–^ cage operates only under diluted μM conditions, albeit with reduced efficiency and fatigue resistance. These effects are attributed to the occurrence of additional excited state pathways, other than the 6π DTE electrocyclization, and the conformational hindrance, which restricts the flexibility of the DTE ligands within the rigid coordination environment of the cage. These findings highlight the challenges in balancing molecular design with functional performance involving main group metal assemblies. This work provides the first demonstration of integrating DTE units with supramolecular cages derived from main group metal cations, specifically Ga^3+^, setting the groundwork for future developments in main group coordination photoswitchable assemblies.

## Experimental Section

### General Methods

All commercially available reagents were used without further purification. Methanol was purified by distillation over iodine-activated magnesium under a nitrogen atmosphere and stored under 3 Å molecular sieves. Multinuclear NMR spectra were obtained using Bruker AVIIIHD 400 (9.4 T) and Bruker AVIII 500 (11.7 T with a Prodigy cryoprobe) spectrometers at 298 K. The assignments were supported by 2D NMR gCOSY, gHSQC, and gHMBC experiments. DOSY NMR experiments were performed at 298 K (see Supporting Information for full details). High-resolution mass spectra were acquired on a Bruker Compact Elite QTOF instrument equipped with an electrospray ionization (ESI) source.

### Single-Crystal X-Ray Diffraction

X-ray diffraction data were collected under nitrogen using an Oxford Cryostream 1000 unit^67^ at specified temperatures. For **2** (CCDC 2364246), a Bruker D8 Quest Eco diffractometer (Mo Kα radiation) with a Photon II detector was used. For **6** (CCDC 2364247), data were collected on a Rigaku XtaLAB Synergy diffractometer (Cu Kα radiation) with a HyPix-Arc 100 detector. Raw frame data were reduced using APEX3^68^ for **2**, whereas CrysAlisPRO^69^ was used for **6**. The structures were solved using SHELXT^70^ and refined using full-matrix least-squares refinement on all *F*^2^ data using SHELXL^71^ using the interface GUI OLEX2.^72^ Unless otherwise stated, all non-hydrogen atoms were refined anisotropically, and hydrogen atoms were geometrically placed and allowed to ride on their parent atoms. Disorder was treated by introducing a split site model and restraining the geometries and displacement parameters. Distances and angles were calculated using the full covariance matrix. Selected crystallographic and refinement data and supplementary figures and metrics are given in the Supporting Information.

### Computational Methods

The Gaussian 16 software package^73^ and CREST program (Conformer-Rotamer Ensemble Sampling Tool)^74^ were used for the optimization and conformational sampling study of the geometries. Geometry optimizations were carried out at the DFT level using the B3LYP functional,^75,76^ including dispersion corrections (D3),^77^ with the 6-31G(d) basis set for the main group elements and the scalar relativistic Stuttgart–Dresden (SDD) pseudopotential complemented with a set of d polarization functions for the gallium ions.^78^ The solvent effects were considered with the SMD continuum solvent model with DMF as the solvent in all calculations (ε = 37.219).^79^ The quasi-rigid-rotor-harmonic-oscillator (quasi-RRHO) approach^80^ was employed for thermal contributions to the Gibbs energies using the GoodVibes program.^81^ Full details of energies and Cartesian coordinates are given in the Supporting Information.

### Hydrodynamic Diameter Analyses

The estimated values for the hydrodynamic diameters (*d*_hyd_, Å) of the ΔΔ-helicate and ΔΛ-mesocate isomers of the [Ga_2_**7**_3_]^6–^ in their opened and closed forms were obtained from the electronically computed structures in DMF. These estimations were calculated using geometrically centered spherical probes at the given diameter (Å) within space-filling models, utilizing van der Waals radii for all atoms as reported by S. Álvarez.^82^ The spherical probe was fitted to the smallest dimension, representing the hydrodynamic diameter typically observed in DOSY NMR experiments for rod-like molecules, where the diffusion coefficient often corresponds to the smaller dimension of the structure.^83,84^ This method provides a practical approximation of the hydrodynamic diameter in solution, correlating well with the experimental hydrodynamic diffusion diameter from the DOSY experiments (see full details in the Supporting Information).

### Photochemical Methods

The photochemical measurements were carried out at room temperature (298 K) with air-equilibrated solutions contained in quartz cuvettes with a 1 cm optical path length. Dimethylformamide (DMF) was the solvent used for these measurements and is commercially available from Sigma-Aldrich with the highest purity level. For the photoswitching experiments, a TLC lamp (Vilber Lourmat-6.LC, 365 nm) and a 150 W xenon lamp (Oriel GmbH and Co. KG) with a 495 nm long-pass filter were employed. For the UV/vis absorption measurements, a CARY 5000 UV/vis spectrophotometer (Agilent) was used. The quantum yields of the DTE ring-closure process and ring-opening process were measured using the adequate actinometers and following a reported methodology.^85^ To carry out these experiments, the opened forms were irradiated at 365 nm, and potassium trioxalatoferrate(III)trihydrate was used (*Φ*_r_ = 1.21 in a buffer solution of 0.23 M CH_3_COONa/0.05 M H_2_SO_4_),^86^ and the closed forms were irradiated at 580 nm, with 1,2-bis(2,4-dimethyl-5-phenyl-3-thienyl)-3,3,4,4,5,5-hexafluoro-1-cyclopentene employed as the actinometer (*Φ*_r_ = 0.0173 in *n-*hexane).^38^ The photostationary state distribution for ring-closing and ring-opening was measured by following the photochemical reaction by ^1^H NMR spectroscopy.

### Synthesis and Characterization

Compound **1** was prepared by following a reported procedure^54^; see Schemes 1 and 2.

Compound **2**: an oven-dried Schlenk tube was charged with compound **1** (440 mg, 1 mmol), 2,3-dimethoxyphenylboronic acid (732 mg, 4 mmol), Na_2_CO_3_ (640 mg, 6 mmol), Pd(PPh_3_)_4_ (233 mg, 20 mol %), X-Phos (143 mg, 30 mol %), water (10 mL, degassed), and ethylene glycol dimethyl ether (40 mL, degassed) under a nitrogen atmosphere at room temperature. The reaction mixture was refluxed overnight. Then, the mixture was quenched with water and extracted with Et_2_O. The organic layer was dried over anhydrous Na_2_SO_4_, and the crude product was purified by column chromatography with silica gel (Merck-60, 230–400 mesh, 60 Å) using a mixture of *n*-hexane/AcOEt (4:1). **2** was obtained as a dark blue solid (334 mg, 73%). ^1^H NMR (400 MHz, CDCl_3_): δ, 7.47 (s, 2H), 7.21 (d, 2H, *J* = 7.8 Hz), 7.07 (t, 2H, *J* = 8.0 Hz), 6.85 (d, 2H, *J* = 8.1 Hz), 3.90 (s, 6H), 3.81 (s, 6H), 1.96 (s, 6H). ^13^C{^1^H} NMR (101 MHz, CDCl_3_): δ, 14.4, 56.1, 60.3, 111.6, 119.4, 124.5, 124.9, 125.0, 127.4, 136.8, 142.7, 145.6, 153.6. HRMS (ESI, QTOF) *m*/*z* calc. for [M + Na]^+^, [C_31_H_26_F_6_NaO_4_S_2_]^+^, 663.1069; found, 663.1053. Crystals of **2** suitable for an X-ray diffraction study were obtained by the slow diffusion of *n*-octane into a solution of the product in 1,2-dichloroethane.

Compound **3**: BBr_3_ (0.17 mL, 1.82 mmol) was added via a syringe to a solution of **2** (100 mg, 0.11 mmol) in CH_2_Cl_2_ (10 mL) under a nitrogen atmosphere at −78 °C. Then, the reaction mixture was left stirring overnight at room temperature. After this time, the volatiles were removed under vacuum, and the resulting crude product was stirred with water for 2 h at 100 °C. The product was extracted with CH_2_Cl_2_ and the organic layer was dried over anhydrous Na_2_SO_4_. After removal of all volatiles under vacuum, **3** was obtained as a dark green solid (89 mg, 95%). ^1^H NMR (400 MHz, CD_3_OD): δ, 7.57 (s, 2H), 7.01 (dd, 2H, *J* = 7.8, 1.8 Hz), 6.71 (dt, 4H, *J* = 15.5, 7.8 Hz), 1.96 (s, 6H). ^13^C{^1^H} NMR (101 MHz, CD_3_OD): δ, 14.3, 115.0, 119.4, 120.6, 121.7, 125.5, 126.0, 140.1, 142.0, 143.7, 146.9. HRMS (ESI, QTOF) *m*/*z* calc. for [M + Na]^+^, [C_27_H_18_F_6_NaO_4_S_2_]^+^, 607.0443; found, 607.0374.

Compound **5**: a suspension of 2,3-dimethoxybenzoic acid (1.25 g, 6.85 mmol), iodoaniline (1 g, 4.56 mmol), Et_3_N (1.38 g, 13.70 mmol), and HBTU (3.46 g, 9.12 mmol) in DMF (30 mL) was stirred for 48 h at room temperature. Then, the solution was diluted with water (15 mL), and the product was extracted with ethyl acetate (3 × 50 mL). The combined organic layers were dried over anhydrous Na_2_SO_4_, and the solvent was removed in vacuo to give the crude product, which was purified by column chromatography with silica gel (Merck-60, 230–400 mesh, 60 Å) using (1–5%) CH_3_OH/CH_2_Cl_2_ as eluent. **5** was obtained as a yellow solid (1.73 g, 85%). ^1^H NMR (400 MHz, CDCl_3_): δ, 10.03 (s, 1H), 7.76 (dd, 1H, *J* = 8.0, 1.7 Hz), 7.66 (d, 2H, *J* = 8.8 Hz), 7.48 (d, 2H, *J* = 8.8 Hz), 7.21 (t, 1H, *J* = 8.0 Hz), 7.10 (dd, 1H, *J* = 8.1, 1.7 Hz), 3.99 (d, 3H, *J* = 1.4 Hz), 3.93 (s, 3H). ^13^C{^1^H} NMR (126 MHz, CDCl_3_): δ, 56.6, 62.1, 87.6, 116.4, 122.3, 123.4, 125.2, 126.9, 138.4, 138.6, 147.6, 153.0, 163.5. HRMS (ESI, QTOF) *m*/*z* calc. for [M + Na]^+^, [C_15_H_14_INNaO_3_]^+^, 405.9922; found, 405.9700.

Compound **6**: first, **4** was prepared in situ as follows. An oven-dried Schlenk tube was charged with **1** (200 mg, 0.46 mmol) and dry diethyl ether (10 mL) under a nitrogen atmosphere at room temperature. Subsequently, *n*-BuLi (0.46 mL, 2.5 M in hexane, 1.15 mmol) was added dropwise, and the reaction mixture was stirred for 30 min at room temperature. Then, tris(isopropyl)borate (0.26 mL, 1.15 mmol) was added dropwise, and the reaction mixture was stirred for 1 h at room temperature to give a solution of **4**. Following this, a second Schlenk tube was charged, under a nitrogen atmosphere, with **5** (387 mg, 1.01 mmol), Na_2_CO_3_ (292 mg, 2.75 mmol), X-Phos (66 mg, 0.14 mmol), Pd(PPh_3_)_4_ (106 mg, 0.09 mmol), ethylene glycol (3 drops), THF (10 mL), and water (5 mL) (both degassed). To this stirring solution, an in situ generated solution of **4** was added dropwise via cannula at room temperature, and the reaction mixture was refluxed overnight. Then, the reaction was quenched with brine and extracted with diethyl ether. The organic layer was dried over anhydrous Na_2_SO_4_, and the crude product was purified by column chromatography with silica gel (Merck-60, 230–400 mesh, 60 Å) using a mixture of *n*-hexane/AcOEt (5:1). **6** was obtained as a dark blue solid (390 mg, 96%). ^1^H NMR (500 MHz, DMF-*d*_7_): δ, 10.35 (s, 2H), 7.97–7.91 (m, 4H), 7.73 (d, 4H, *J* = 8.7 Hz), 7.57 (s, 2H), 7.32–7.26 (m, 4H), 7.22 (dd, 2H, *J* = 8.3, 7.5 Hz), 3.95 (s, 5H), 3.94 (s, 5H), 2.11 (s, 5H). ^13^C{^1^H} NMR (126 MHz, DMF-*d*_7_): δ, 14.6, 29.8, 30.0, 30.1, 30.3, 30.5, 30.6, 30.8, 34.9, 35.1, 35.3, 35.4, 35.6, 35.8, 35.9, 56.6, 61.9, 115.9, 121.0, 121.4, 123.1, 125.1, 126.4, 126.8, 129.3, 131.4, 140.4, 141.8, 143.0, 147.5 153.8, 165.6. HRMS (ESI, QTOF) *m*/*z* calc. for [M + Na]^+^, [C_43_H_34_F_6_NaO_4_S_2_]^+^, 901.1811; found, 901.1733. Crystals of **6** suitable for an X-ray diffraction study were obtained by slow diffusion of *n*-octane into a solution of the product in 1,2-dichloroethane.

Compound **7**: this compound was prepared by reacting **6** (100 mg. 0.11 mmol) with BBr_3_ (0.172 mL, 1.82 mmol) following the same method used for **3**. **7** was obtained as a dark green solid (89 mg, 95%). ^1^H NMR (500 MHz, DMF-*d*_7_): δ, 12.10 (s, 2H), 10.57 (s, 2H), 9.56 (s, 2H), 7.92 (d, 4H, *J* = 8.7 Hz), 7.76 (d, 4H, *J* = 8.7 Hz), 7.62 (dd, 2H, *J* = 8.2, 1.5 Hz), 7.60 (s, 2H), 7.08 (dd, 2H, *J* = 7.8, 1.4 Hz), 6.82 (t, 2H, *J* = 8.0 Hz), 2.12 (s, 5H). ^13^C{^1^H} NMR (126 MHz, DMF-*d*_7_): δ, 14.7, 29.8, 30.0, 30.1, 30.3, 30.5, 30.6, 30.8, 34.9, 35.1, 35.3, 35.4, 35.6, 35.8, 35.9, 117.2, 119.0, 119.4, 120.1, 122.59, 123.3, 126.4, 126.7, 129.9, 139.3, 142.1, 142.9, 147.8, 150.5, 162.7, 162.9, 163.2, 169.5. HRMS (ESI, QTOF) *m*/*z* calc. for [M + Na]^+^, [C_41_H_28_F_6_N_2_NaO_4_S_2_]^+^, 845.1185; found, 845.1138.

[Ga_2_**7**_3_][(Me_4_N)_2_K_4_]: **7** (80 mg, 0.10 mmol) was dissolved in MeOH (20 mL) and then a solution of KOH (0.77 mL, 0.39 mmol, from a 0.5 M commercial solution in MeOH, 0.388 mmol) was added dropwise under a nitrogen atmosphere. Then, the reaction mixture was left stirring for 30 min at room temperature. After this time, Me_4_NBr (90 mg, 0.58 mmol) and Ga(acac)_3_ (24 mg, 0.06 mmol) were added under a flush of nitrogen gas, and the reaction mixture was stirred for 16 h. Then, the solvent was partially removed under vacuum (5 mL), and diethyl ether (50 mL) was added to give a gray suspension. The resulting solid was collected by filtration, washed with diethyl ether (3 × 10 mL), and dried under vacuum to give [Ga_2_**7**_3_][(Me_4_N)_2_K_4_] as a gray solid, which was further recrystallized from a DMF solution by adding a mixture of AcOEt/THF as a precipitant. [Ga_2_**7**_3_][(Me_4_N)_2_K_4_] was obtained as a crystalline solid. Despite the crystalline nature of [Ga_2_**7**_3_][(Me_4_N)_2_K_4_], all attempts gave unsuitable crystals for X-ray crystallography. ^1^H NMR (500 MHz, DMF-*d*_7_): δ, 14.49 (s, 6H), 7.97 (d, 12H, *J* = 8.2 Hz), 7.23 (s, 6H), 7.00 (dt, 19H, *J* = 8.1, 2.6 Hz), 6.35 (dd, 6H, *J* = 7.2, 1.7 Hz), 6.20–6.12 (m, 6H), 1.81 (s, 17H). ^13^C{^1^H} NMR (126 MHz, DMF-*d*_7_): δ, 15.2, 56.06, 56.09, 56.13, 113.2, 113.7, 114.0, 115.7, 120.5, 127.3, 127.5, 143.1, 145.0, 158.7, 160.4, 169.0. HRMS (ESI, QTOF) *m*/*z* calc. for [Ga_2_**7**_3_+K_2_]^4–^, [(C_41_H_24_F_6_N_2_O_6_S_2_)_3_Ga_2_K_2_]^4–^, 668.5193; found, 668.5196; [Ga_2_**7**_3_+K_3_]^3–^, [(C_41_H_24_F_6_N_2_O_6_S_2_)_3_Ga_2_K_3_]^3–^, 904.3463; found, 904.3467; [Ga_2_**7**_3_+K_4_]^2–^, [(C_41_H_24_F_6_N_2_O_6_S_2_)_3_Ga_2_K_4_]^2–^, 1376.0016; found, 1376.0017.

## Supporting Information Available

Characterization by NMR spectroscopy; opened form of DTEs and the coordination [Ga_2_**7**_3_][(Me_4_N)_2_K_4_] cage (Chart S1); ^1^H DOSY NMR analysis; ^1^H NMR, ^13^C NMR, ^19^F NMR, ^1^H-^1^H COSY, ^1^H-^13^C HMBC, ^1^H-^13^C HSQC, and DOSY spectra of compounds (Figures S1–S38, S49, and S50); characterization by HRMS (ESI-QTOF); HMRS spectra (Figures S39–S47); UV/Vis absorption spectra (Figure S48); selected crystallographic and refinement data (Tables S1 and S2); molecular structure, packing diagram, and interatomic O···O distances of compounds (Figures S51–S53); bond lengths and angles for compounds **2** and **6** (Tables S3 and S4); estimated molecular diameter for the cages (Figure S57); calculated relative energies (Tables S5 and S6); Cartesian coordinates (PDF)
