# Supplementary material for: Synthesis, Characterization, and Photochemistry of a Ga2L3 Coordination Cage with Dithienylethene-Catecholate Ligands
Source: Inorg Chem. 2024 Oct 8;63(42):19872–84. doi: 10.1021/acs.inorgchem.4c03279 (PMC11497204; doi:10.1021/acs.inorgchem.4c03279)
Supplement: Supplementary file 1 — ic4c03279_si_001.pdf [file ic4c03279_si_001.pdf]

Supporting Information

Synthesis, Characterization, and Photochemistry of a Ga_2L_3 Coordination Cage with Dithienylethene-Catecholate Ligands

Adrián Carbonell,[a] Ignacio Izquierdo,[a] David B. Guzmán Ríos,[a] Gantulga Norjmaa,[b] Gregori Ujaque,[b] Antonio J. Martínez-Martínez[a,]* and Uwe Pischel[a,]*

[a] *CIQSO – Center for Research in Sustainable Chemistry and Department of Chemistry, University of Huelva, Campus de El Carmen s/n, E-21071 Huelva, Spain.*

[b] *Departament de Química and Centro de Innovación en Química Avanzada (ORFEO-CINQA), Universitat Autònoma de Barcelona, 08193 Cerdanyola del Vallès, Catalonia, Spain.*

* Correspondence authors:

antonio.martinez@ciqso.uhu.es (A.J.M.-M.)

uwe.pischel@diq.uhu.es (U.P.).

Table of Contents

**1. Characterization by NMR spectroscopy**	**S3**
**2. Characterization by HRMS (ESI-QTOF)**	**S26**
**3. Photochemistry**	**S31**
**4. X-Ray crystallography**	**S34**
**5. Details of hydrodynamic diameter study**	**S55**
**6. Density-Functional-Theory (DFT) Calculations**	**S56**
**7. References**	**S163**

1. Characterisation by NMR spectroscopy

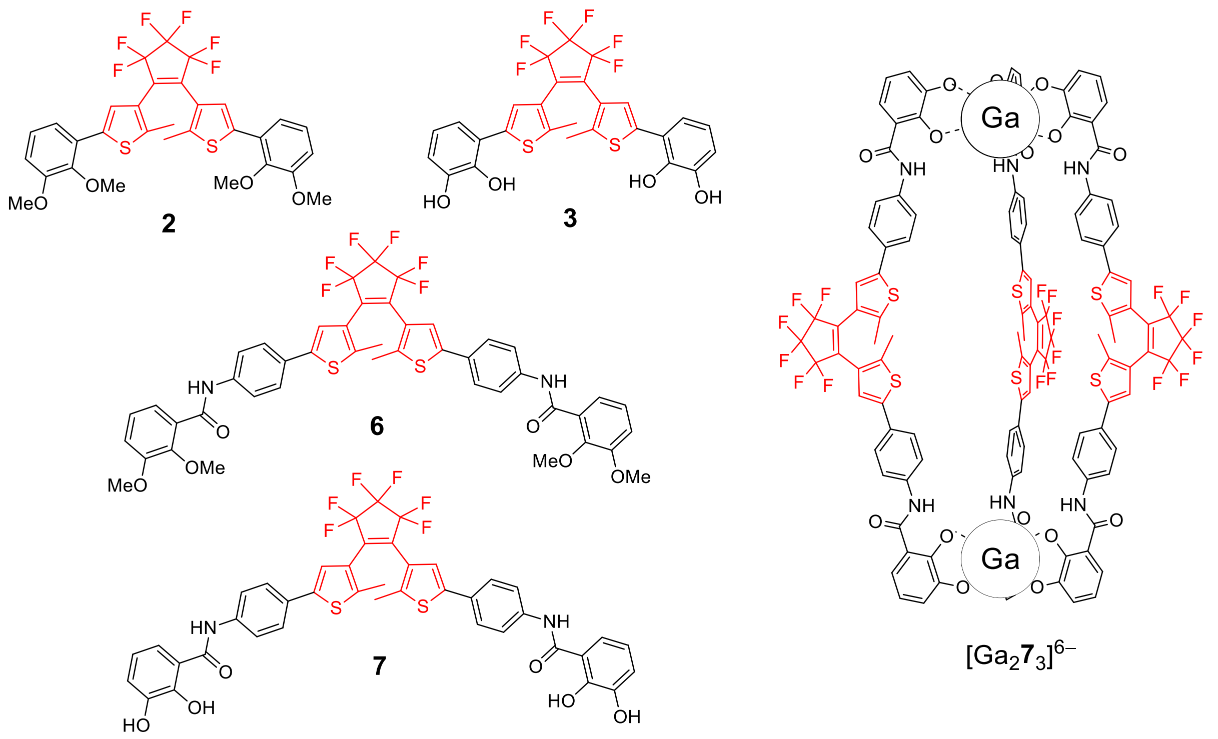

Chart S1. Open form of DTEs **2**, **3**, **6**, **7** and the coordination $[Ga_2\mathbf{7}_3][(Me_4N)_2K_4]$ cage.

The solvents for NMR measurements (CD_3OD ≥ 99.80 atom%, $CDCl_3$; ≥ 99.6 atom%, DMF-d_7 ≥ 99.5 atom% D) were commercially available on Eurisotope. The residual solvent peak (δ = 3.34, 7.26 and 2.75, 2.92, 8.03 ppm for CD_3OD, $CDCl_3$ and DMF-d_7, respectively) was used as the reference signal for the ^1H NMR spectra, and (δ= 49.86, 77.63, and 29.76, 34.89, 163.15 ppm for CD_3OD, $CDCl_3$ and DMF-d_7, respectively) for ^{13}C NMR spectra.

^{1}H DOSY NMR analysis

Diffusion-Ordered Spectroscopy (DOSY) NMR experiments were carried out on a Bruker Bruker AVIII 11.7 Tesla equipped with a BBO/19F-1H/D-5.0-z Prodigy cryoprobe at 298 K. All experiments were conducted at 298 K using TopSpin software (version 4.1, Bruker Biospin, Karlsruhe), on systems with an actively shielded z-gradient coil capable of providing a maximum gradient strength of 5.05 G mm^{-1} at 10 A. Data for diffusion-ordered NMR were collected using the Bruker pulse program ledgpgp2s1d with a double stimulated echo. Sine-shaped gradient pulses with a duration of D21 of 5 ms were used, coupled with a diffusion period D20 of 100 ms, bipolar gradient pulses P30 of 650 μs, and two spoiling gradients P19 of 600 μs. Following the application of each gradient pulse, a gradient recovery delay of 200 μs was implemented. Data were systematically compiled by linearly varying the diffusion encoding gradients from 5% to 95% of the maximum for 32 gradient increment values. The pulse program ledgpgp2s was employed for data acquisition of this pseudo-2D experiment. The signal decay dimension on the pseudo-2D data was generated by Fourier transformation of the time-domain data.

DOSY plots were generated using the DOSY processing with the T1/T2 software package module of TopSpin (version 4.1, Bruker Biospin, Karlsruhe), fitting area data (integration of all peaks of interest of the sample) of diffusion decays. Parameters were empirically optimized to achieve the highest quality of data presentation. Diffusion coefficients (D_x) were calculated by fitting intensity data to the Stejskal-Tanner expression and then normalized ($D_{x,norm}$) using adamantane as an external standard to fit the reference fixed diffusion coefficients ($D_{ref,fix}$) standardized by Stalke.[1, 2] External calibration for DOSY NMR studies was performed due to the high reactivity of the compounds being analyzed, using diffusion coefficients of external standards (1,2,3,4-tetraphenylnaphthalene, 1-phenylnaphthalene, adamantane, and tetramethylsilane, 15 mM each in either toluene-d_8 or

THF-d_8). Linear calibration graphs were obtained by plotting log$D_{x,norm}$ versus *log*MW. By utilizing the normalized diffusion coefficients for the corresponding compound's signals ($D_{x,norm}$, analyte), an estimate of the molecular weight (MW) of the species present in the solution was obtained (OEC, own external calibration method). Our external calibration method was compared with that reported by Stalke,[1, 2] resulting in comparable MW values. In addition, a correction factor was applied to MW that scales incorporating heavier elements from van-der-Waals molecular densities when appropriate.[3] Samples were prepared in 5 mm NMR tubes containing the corresponding compound to be analyzed (typically, 7–14 mg) in an appropriate solvent (0.5 mL).

The hydrodynamic diameter d of the diffusing species in solution for the corresponding compounds was estimated using the Stokes-Einstein equation: $D = k_B T/3\eta\pi d$.[4] Here, D denotes the diffusion coefficient ($m^2 \cdot s^{-1}$), π stands for 3.1415926, k_B represents Boltzmann's constant (1.3806505 J·K), T is the absolute temperature (298 K), and η signifies the solvent viscosity (not the solution viscosity) at 298 K (0.9×10^{-3} pa·s for DMF).[5] According to this equation, the hydrodynamic diameter (D_{hyd}) of the diffusing species in solution is $D_{hyd} = k_B T/3\eta\pi D$ (m). From the diffusion coefficients ($D_{x,norm}$, $m^2 \cdot s^{-1}$) of the external standards used (1,2,3,4-tetraphenylnaphthalene, 1-phenylnaphthalene, adamantane, and tetramethylsilane, 15 mM each in either toluene-d_8 or THF-d_8), linear calibration graphs were obtained by plotting *log*$D_{x,norm}$ versus *log*$D_{X\text{-}ray}$, where $D_{X\text{-}ray}$ *is* an estimate of the diameter of the compounds extracted from their corresponding molecular structures ($D_{X\text{-}ray} = 2 \times R_{X\text{-}ray}$, with $R_{X\text{-}ray} = 2.5$ Å for tetramethylsilane,[6] 3.0 Å for adamantane,[7] 4.0 Å for 1-phenylnaphthalene[8] and 5.75 Å for 1,2,3,4-tetraphenylnaphthalene).[9] For these compounds, $D_{X\text{-}ray}$ (Å) was estimated by calculating an average value from the corresponding molecular structures. This calculation utilized a centred spherical probe with a given radius (Å) in conjunction with a space-filling model that uses van-der-Waals radii reported by S. Álvarez for all atoms.[10] The correlation

between the estimated diameter values extracted from the molecular structures and calculated from the solution showed good agreement (6.4 Å for adamantane calculated from thermodynamic solution studies[8] and 5.0 Å calculated from reported[11] DOSY studies). From the diffusion coefficients of the external standards used (1,2,3,4-tetraphenylnaphthalene, 1-phenylnaphthalene, adamantane and tetramethylsilane, 15 mM each in either toluene-d_8 or THF-d_8), linear calibration graphs were obtained by plotting $log D_{x,norm}$ versus $log D_{X\text{-}ray}$. Furthermore, using the diffusion coefficients for the signals from the corresponding standard, an estimate of the diffusing species present in the solution was achieved, which showed excellent agreement with the estimated $D_{X\text{-}ray}$ values extracted from the molecular structures. Finally, by using the diffusion coefficients for the signals from the cage $[Ga_2\mathbf{7}_3]^{6-}$, D_{cage} (Å) was estimated by calculating an average value from the corresponding electronic structures (see Section 5 and 6).

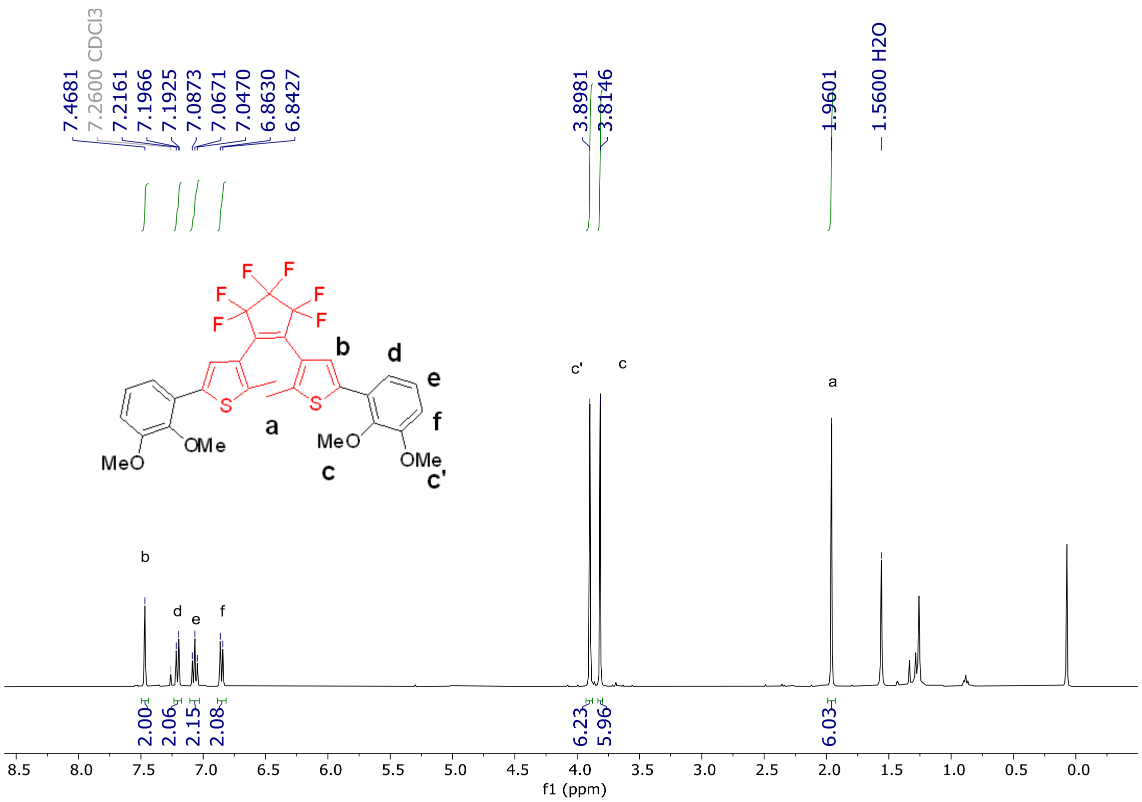

Figure S1. ^1H NMR spectrum (400 MHz) of compound **2** in $CDCl_3$ at 298 K.

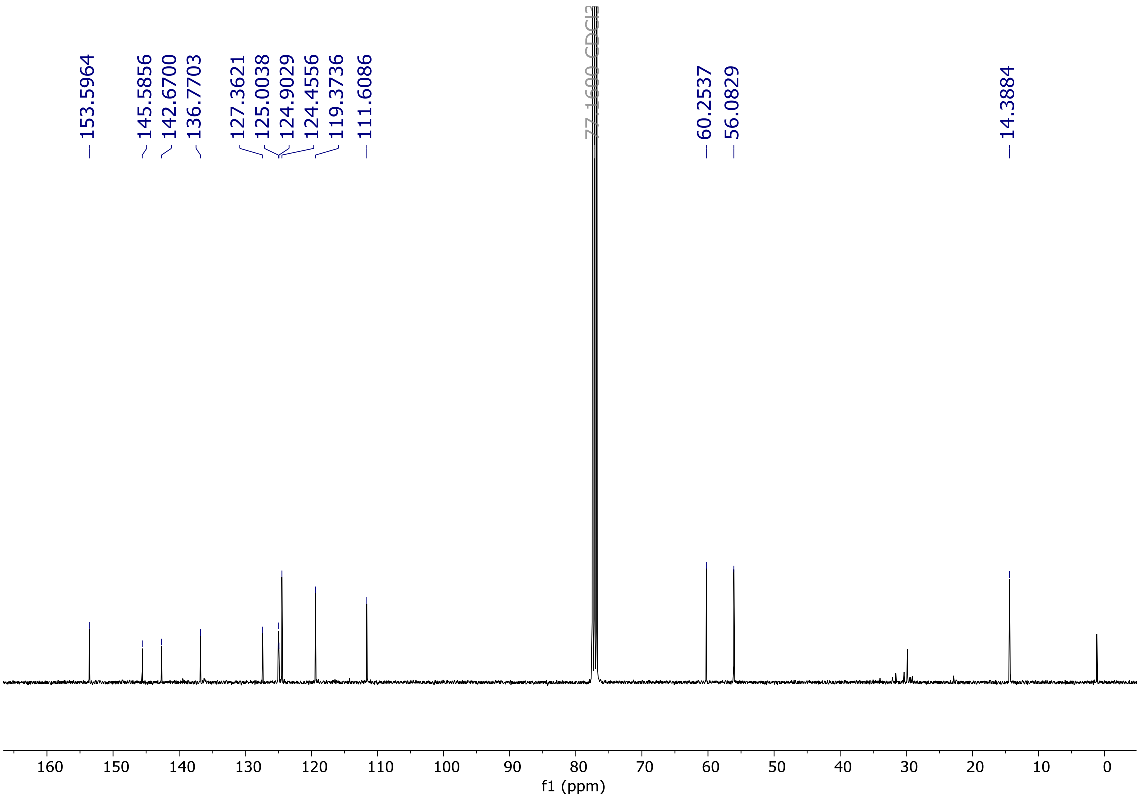

Figure S2. ^{13}C NMR spectrum (101 MHz) of compound **2** in $CDCl_3$ at 298 K.

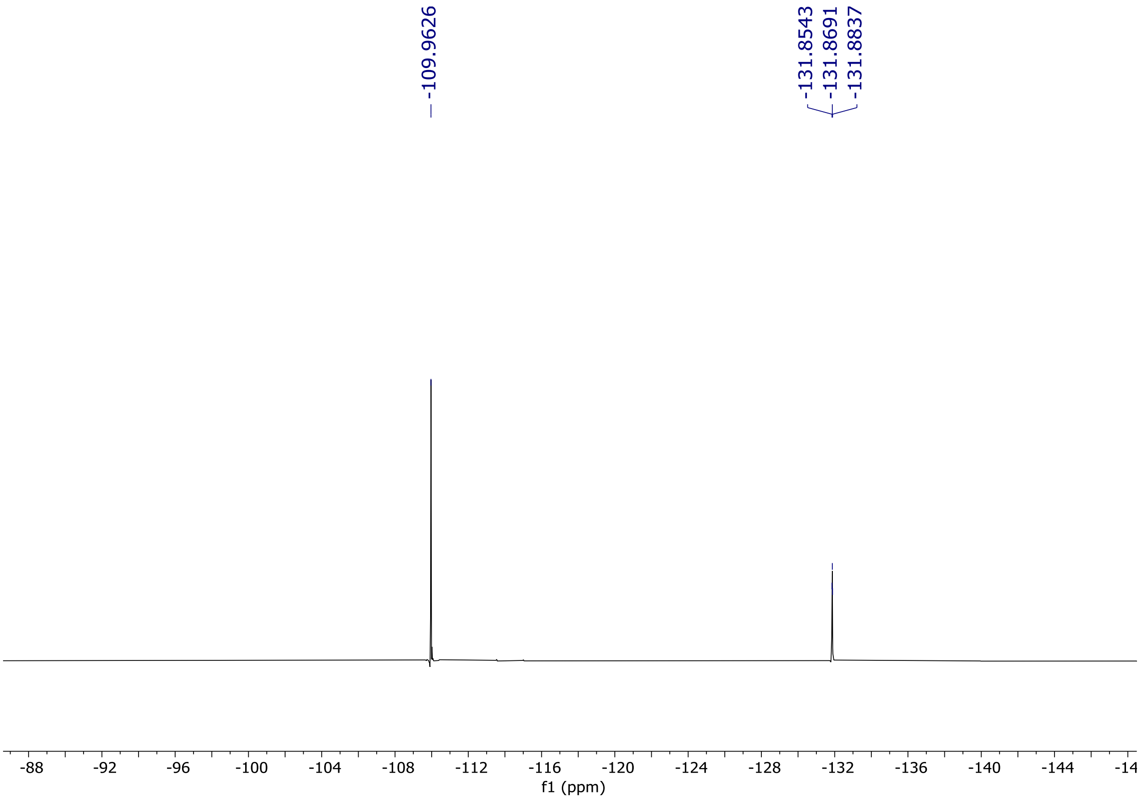

Figure S3. ^{19}F NMR spectrum (376 MHz) of compound **2** in $CDCl_3$ at 298 K.

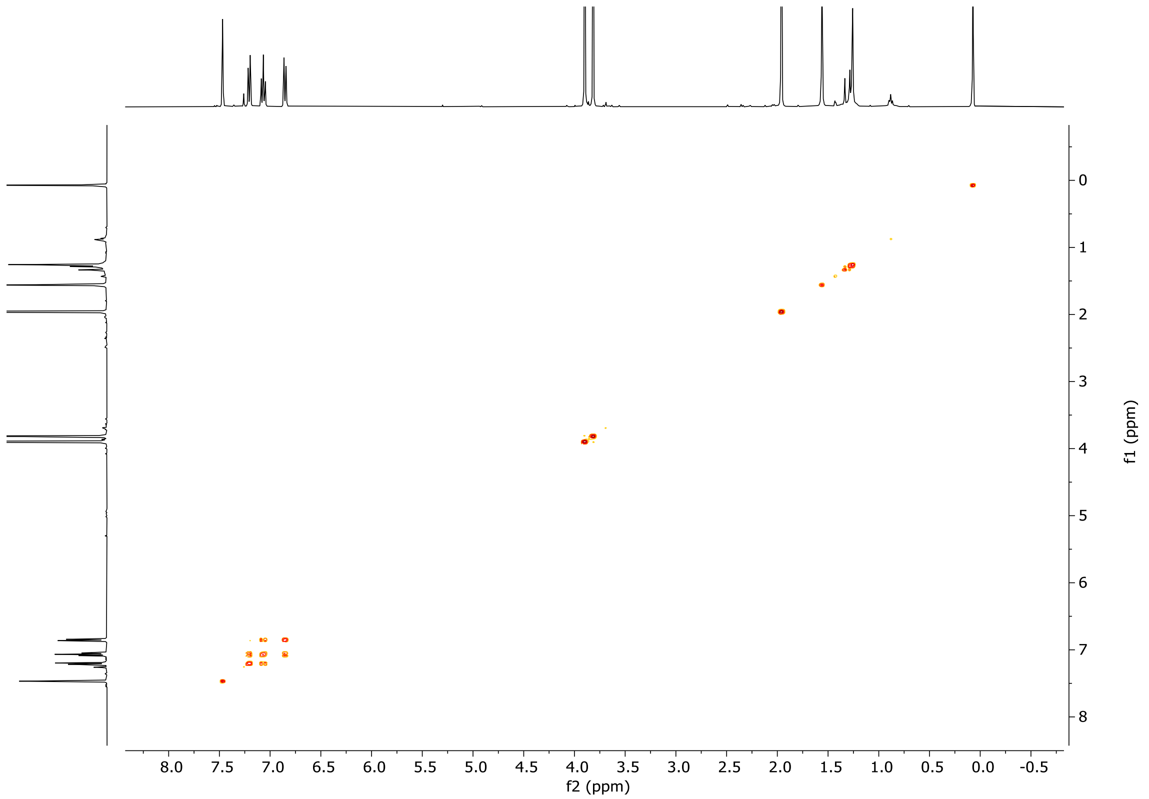

Figure S4. ^{1}H-^{1}H COSY spectrum (500 MHz) of compound **2** in $CDCl_3$ at 298 K.

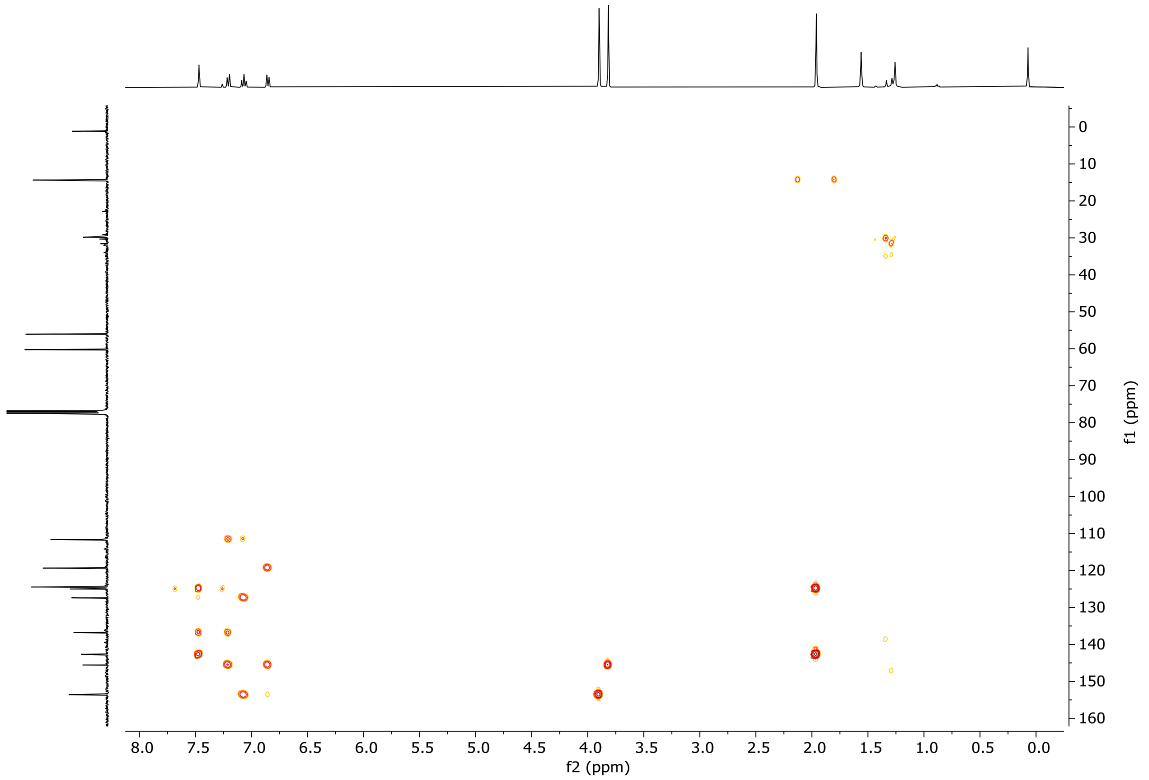

Figure S5. ^{1}H-^{13}C HMBC spectrum (500 MHz) of compound **2** in $CDCl_3$ at 298 K.

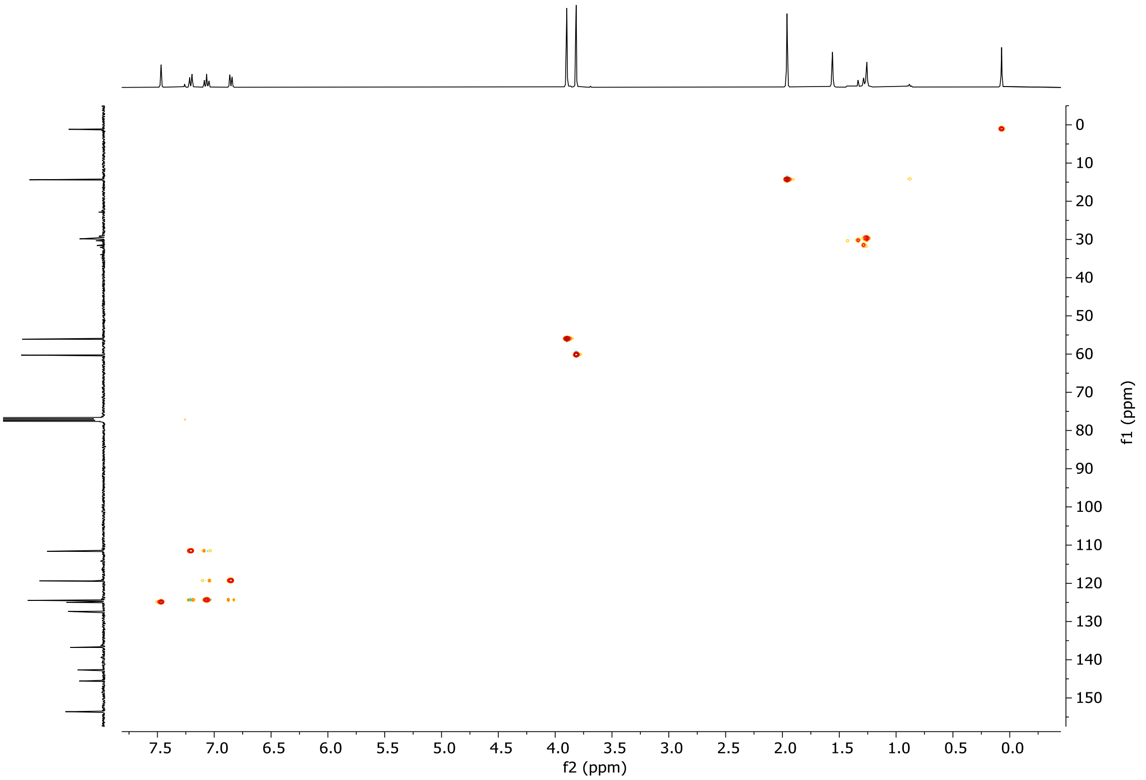

Figure S6. ^{1}H-^{13}C HSQC spectrum (500 MHz) of compound **2** in $CDCl_3$ at 298 K.

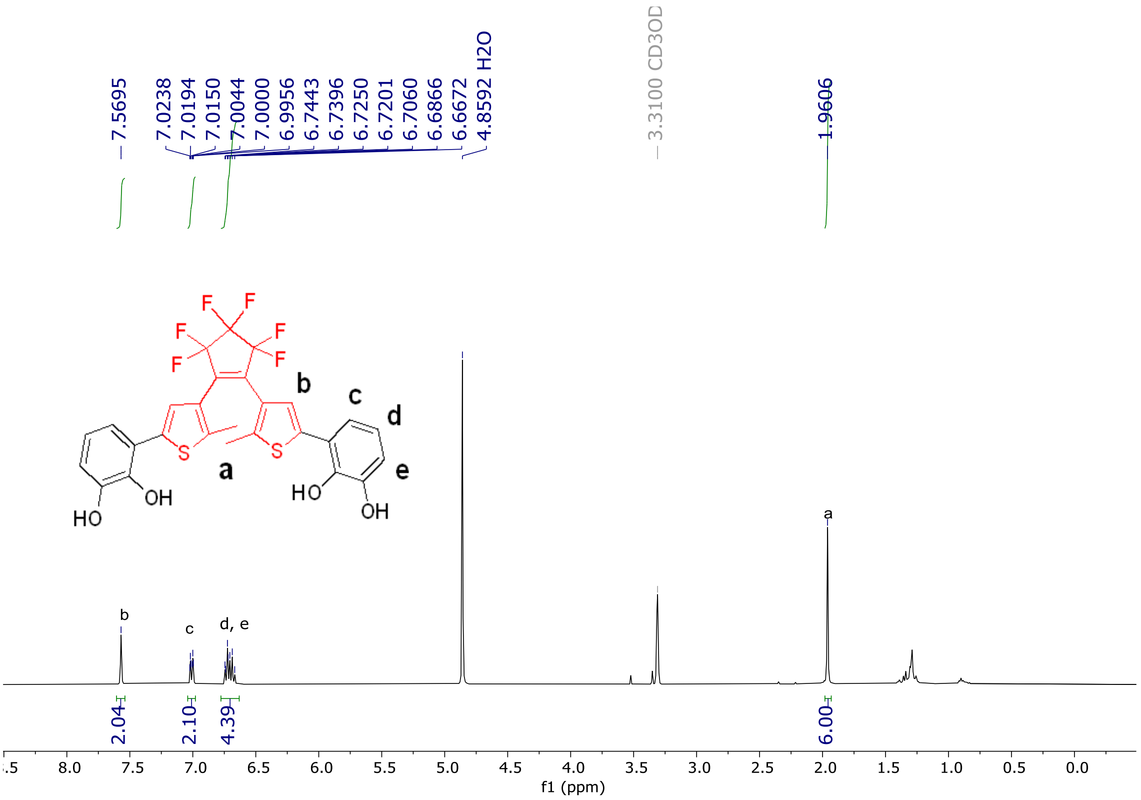

Figure S7. ^{1}H NMR spectrum (400 MHz) of compound **3** in CD_3OD at 298 K.

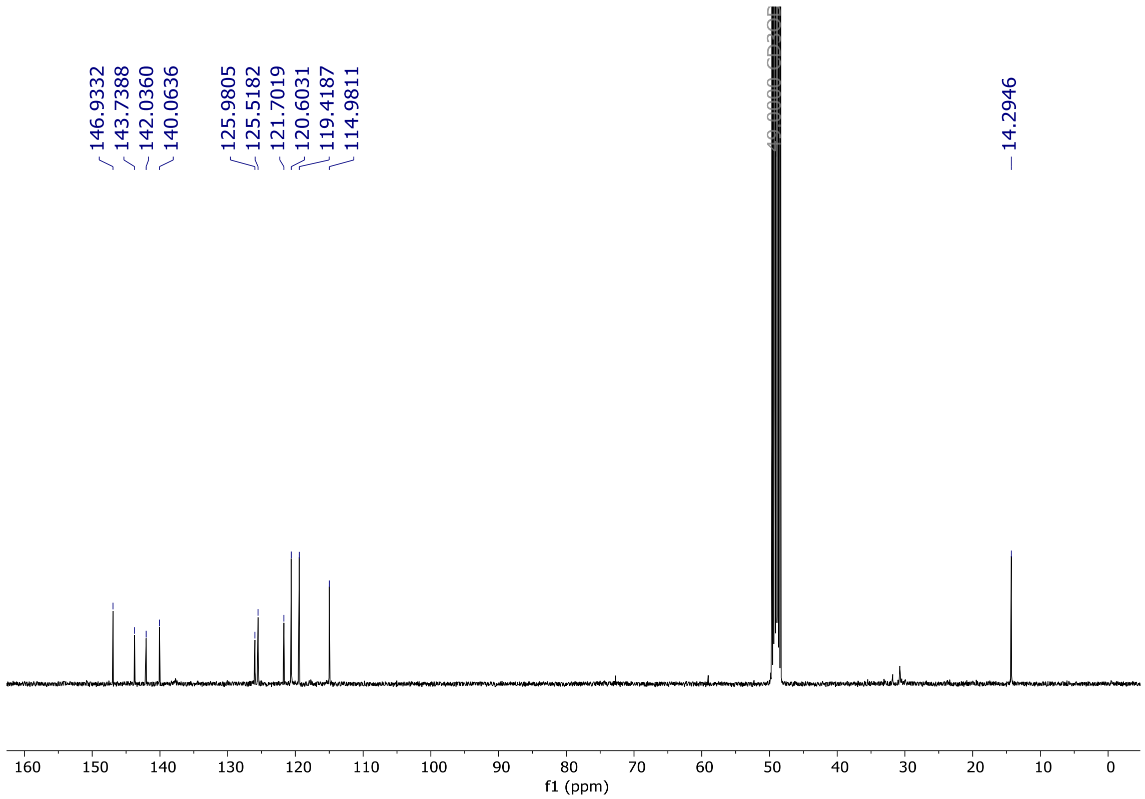

Figure S8. ^{13}C NMR spectrum (101 MHz) of compound **3** in CD_3OD at 298 K.

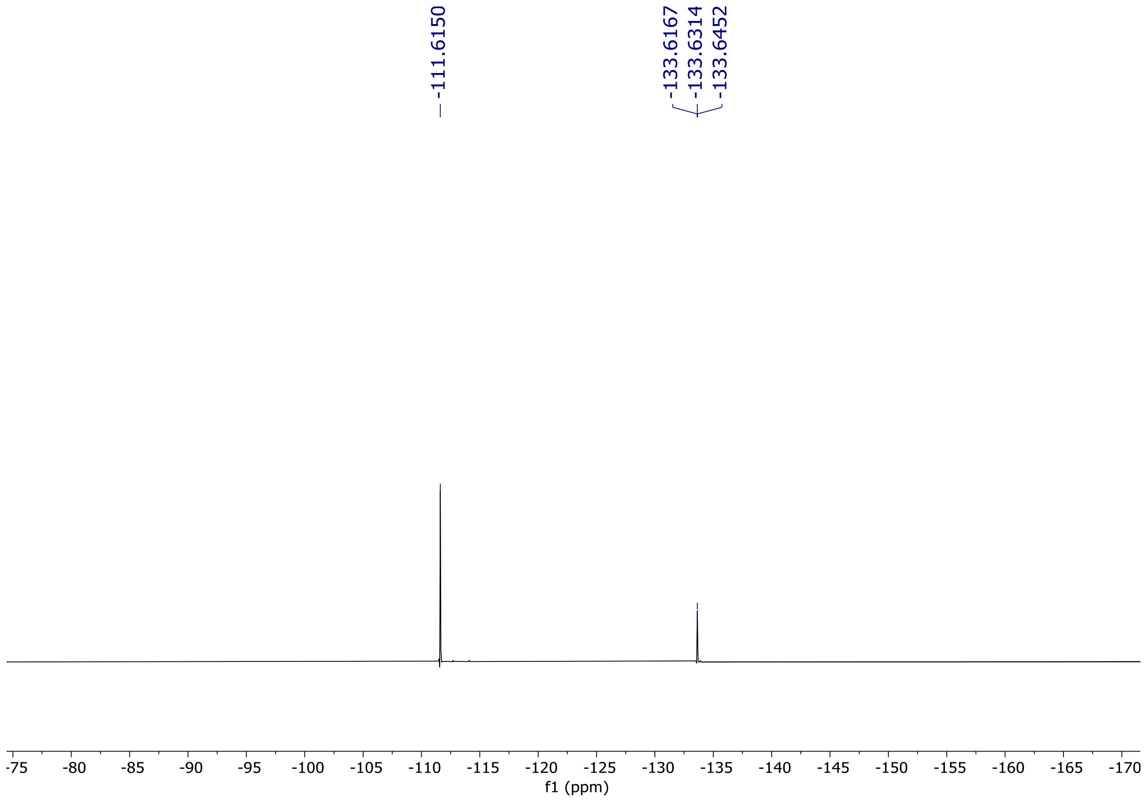

Figure S9. ^{19}F NMR spectrum (376 MHz) of compound **3** in CD_3OD at 298 K.

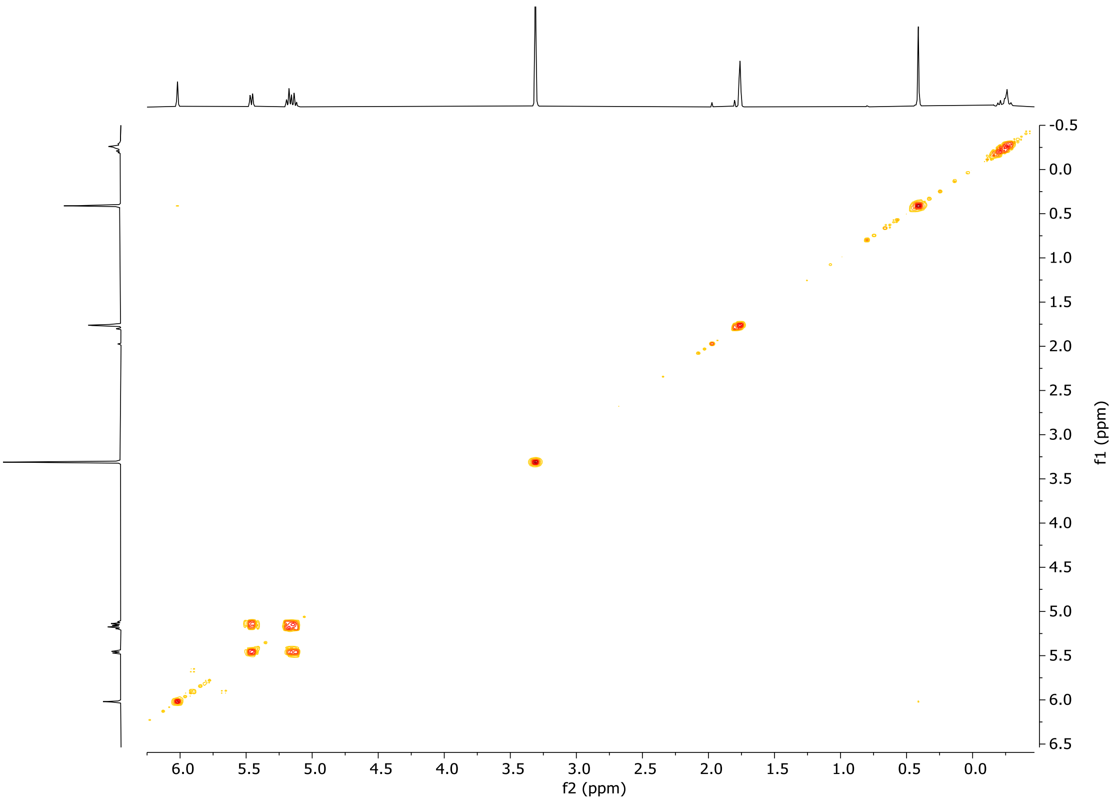

Figure S10. ^{1}H-^{13}C COSY spectrum (500 MHz) of compound **3** in CD_3OD at 298 K.

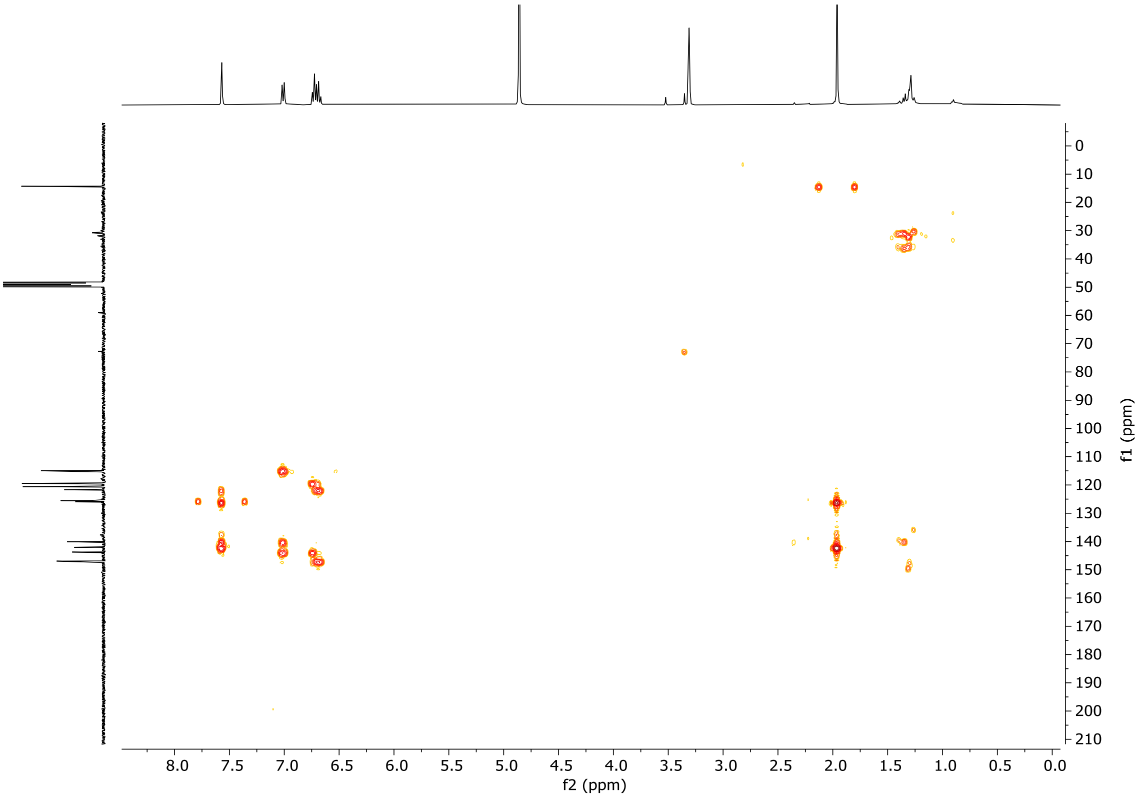

Figure S11. ^{1}H-^{13}C HMBC spectrum (500 MHz) of compound **3** in CD_3OD at 298 K.

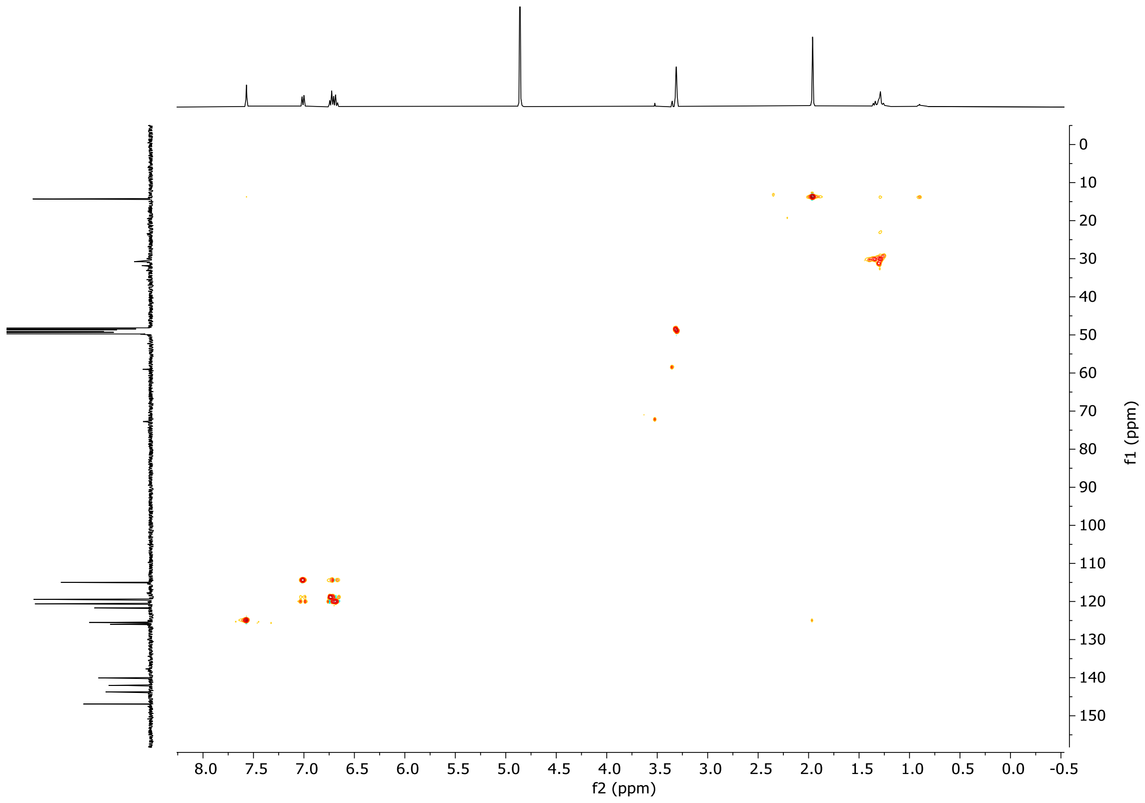

Figure S12. ^{1}H-^{13}C HSQC spectrum (500 MHz) of compound **3** in CD_3OD at 298 K.

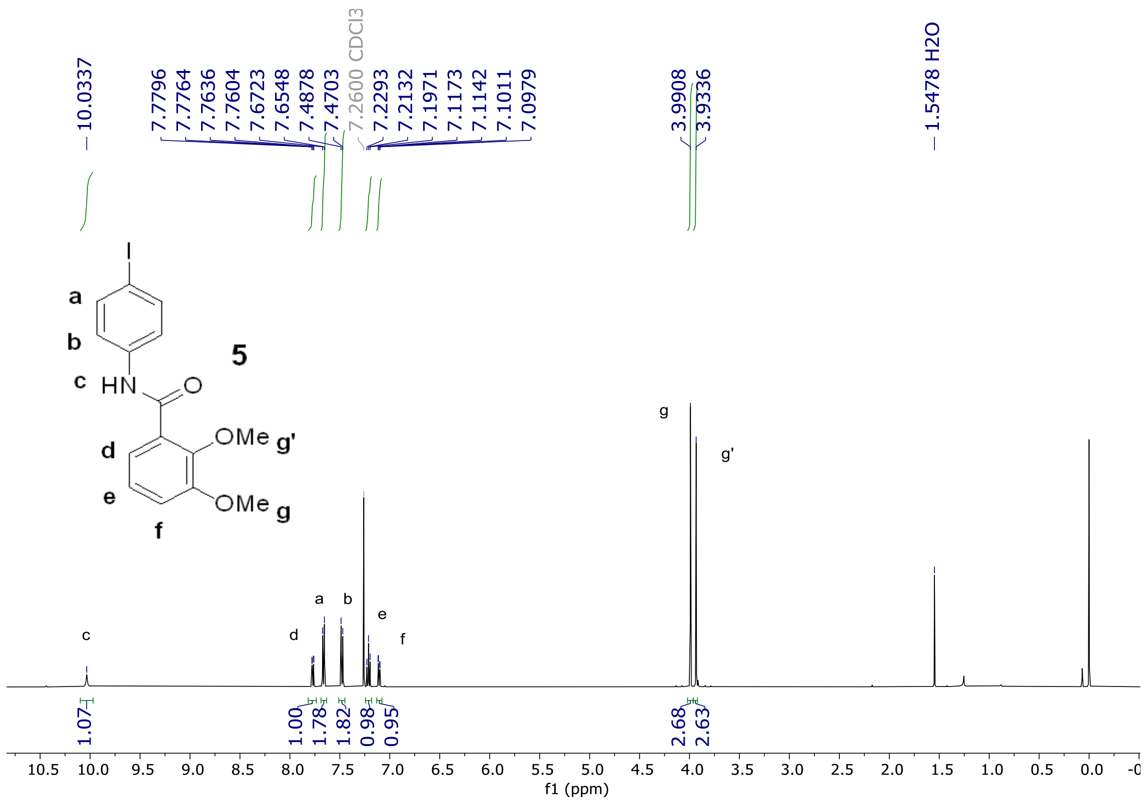

Figure S13. ^{1}H NMR spectrum (500 MHz) of compound **5** in $CDCl_3$ at 298 K.

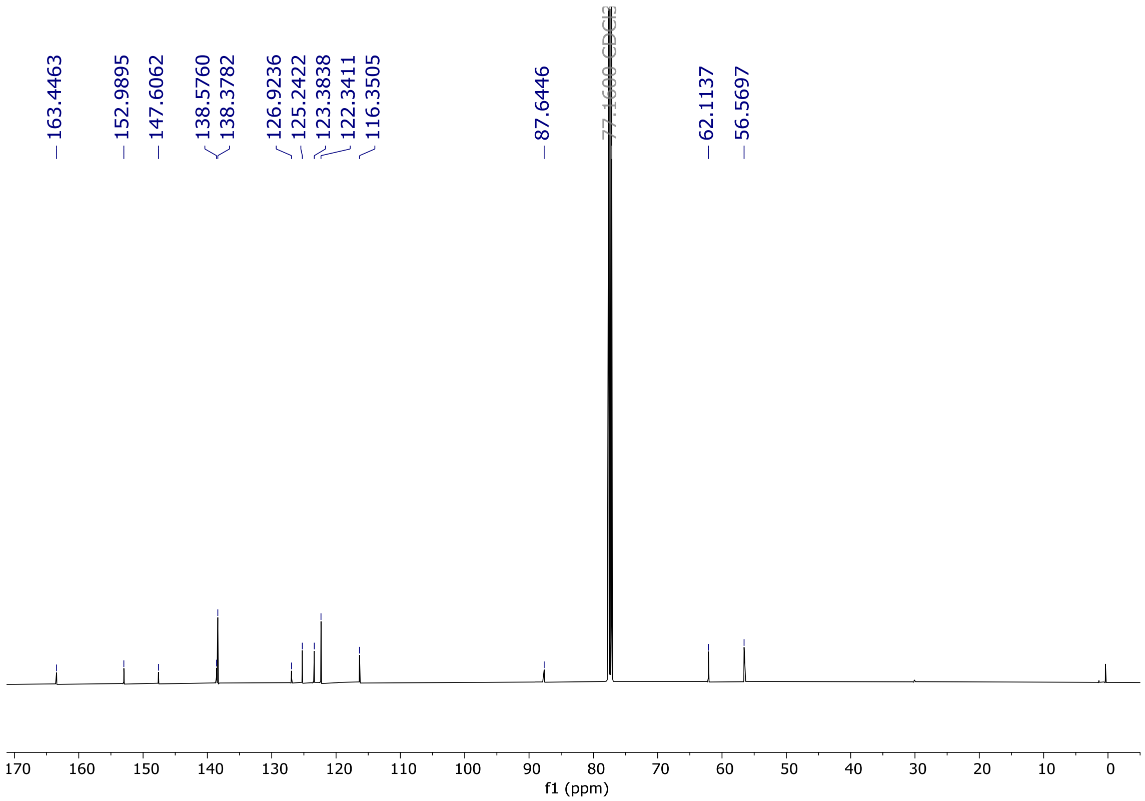

Figure S14. ^{13}C NMR spectrum (126 MHz) of compound **5** in $CDCl_3$ at 298 K.

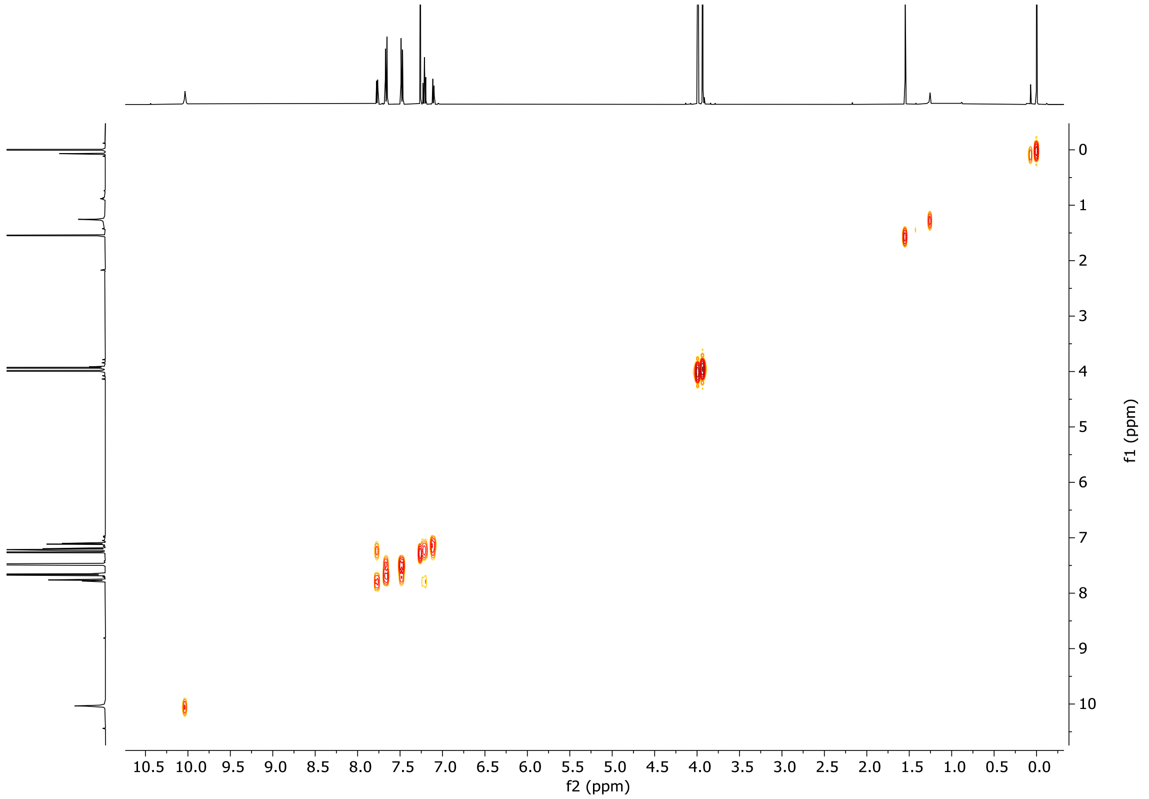

Figure S15. ^{1}H-^{1}H COSY spectrum (500 MHz) of compound **5** in $CDCl_3$ at 298 K.

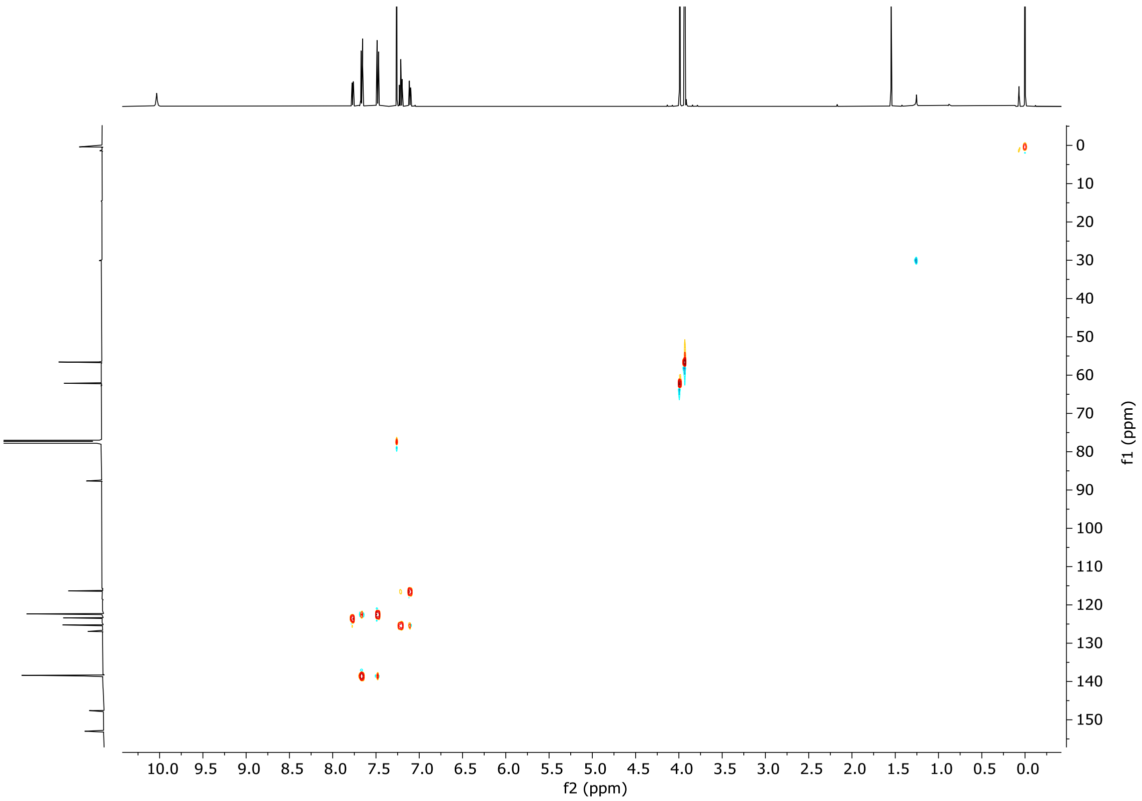

Figure S16. ^{1}H-^{13}C HSQC spectrum (500 MHz) of compound **5** in $CDCl_3$ at 298 K.

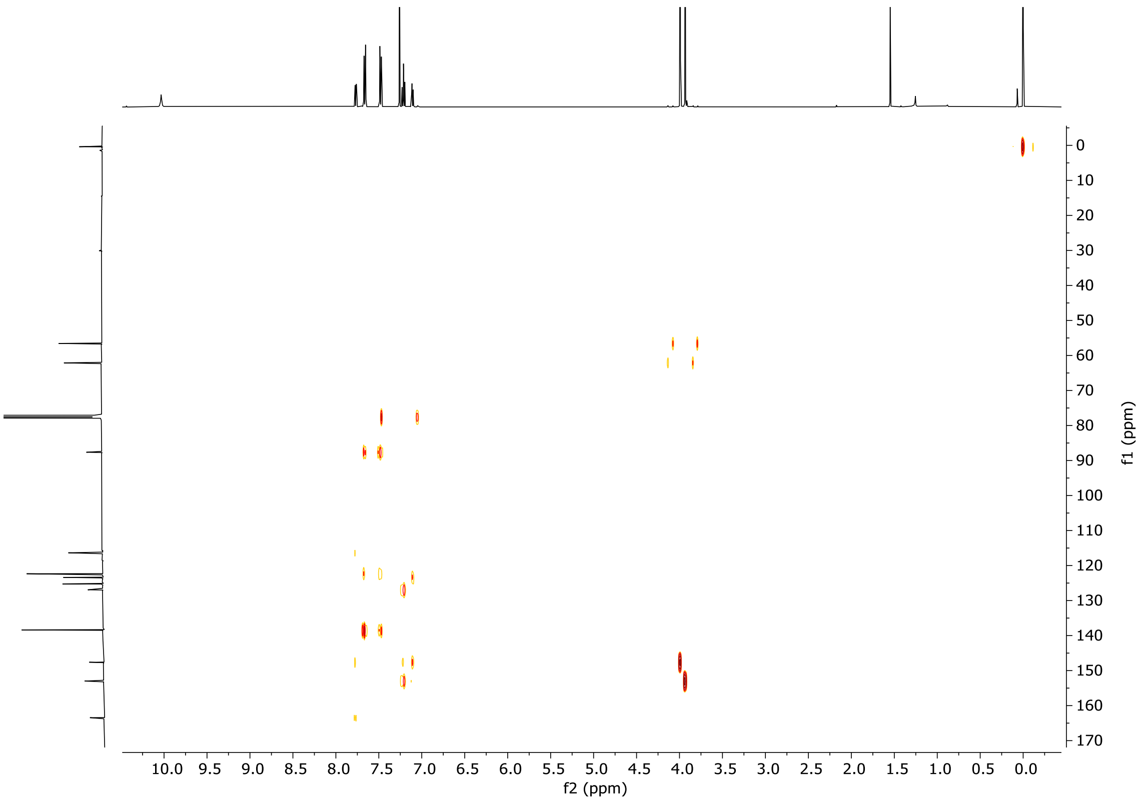

Figure S17. ^{1}H-^{13}C HMBC spectrum (500 MHz) of compound **5** in $CDCl_3$ at 298 K.

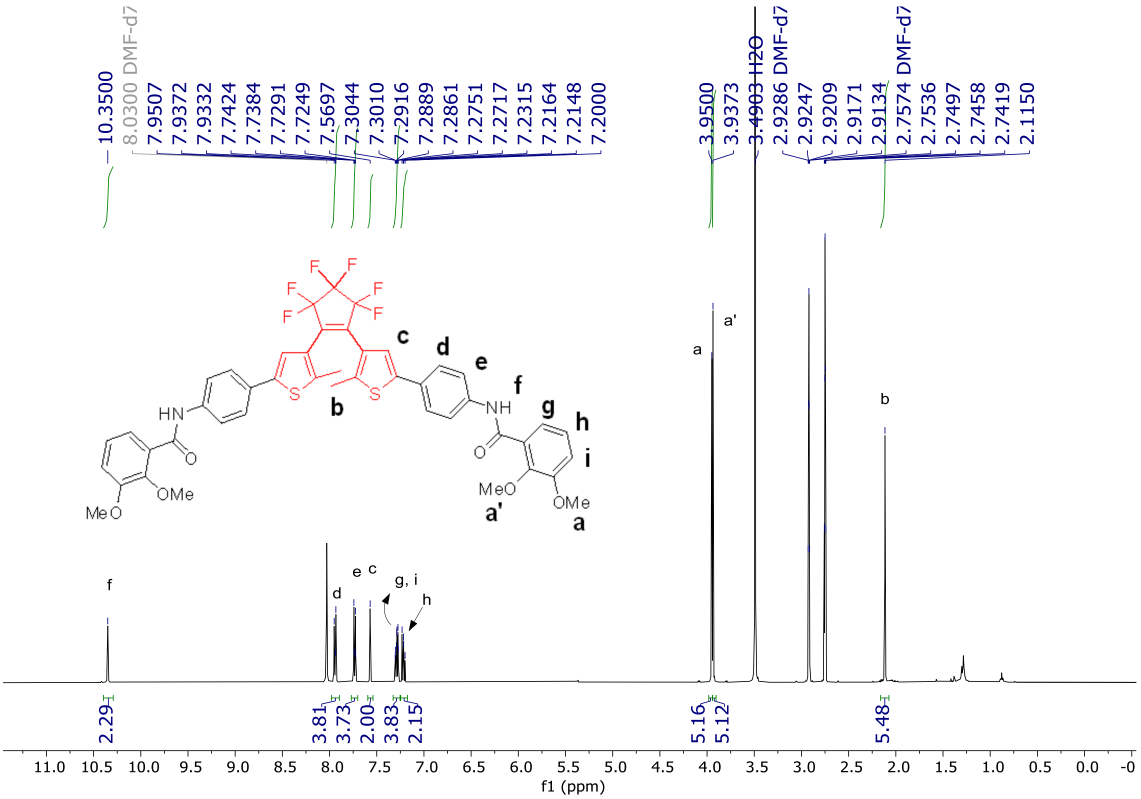

Figure S18. ^{1}H NMR spectrum (500 MHz) of compound **6** in DMF-d_7 at 298 K.

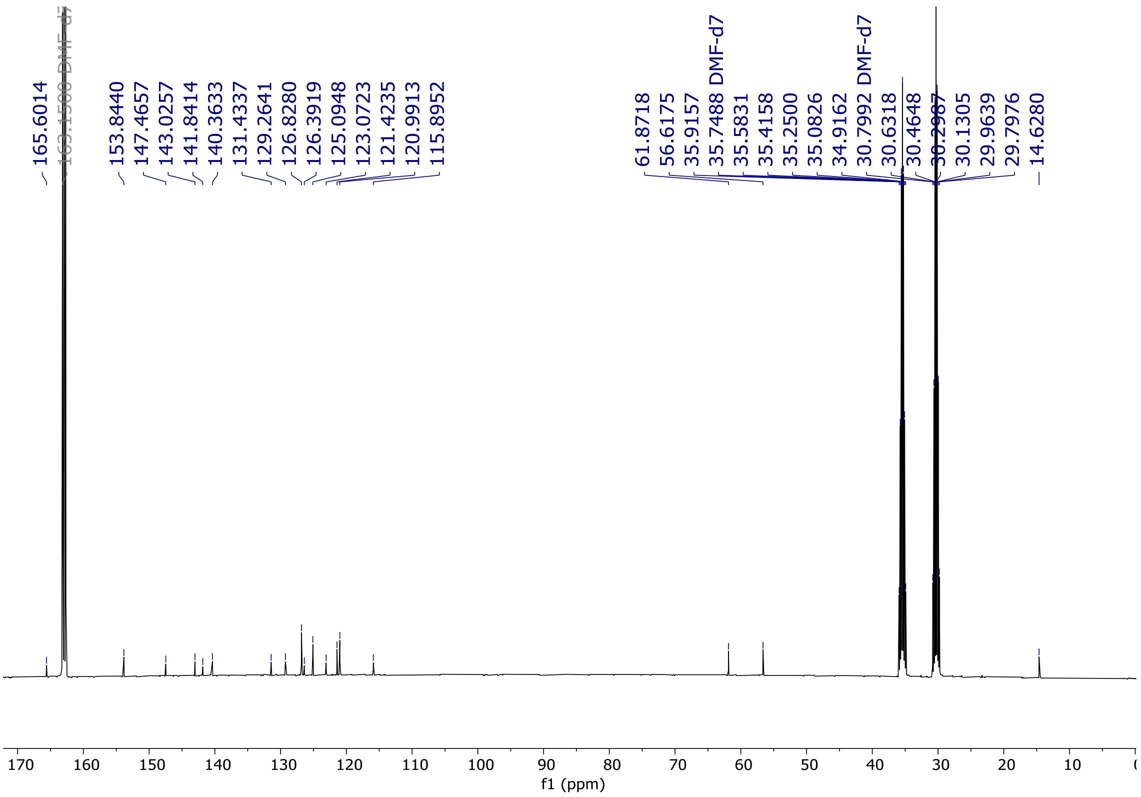

Figure S19. ^{13}C NMR spectrum (126 MHz) of compound **6** in DMF-d_7 at 298 K.

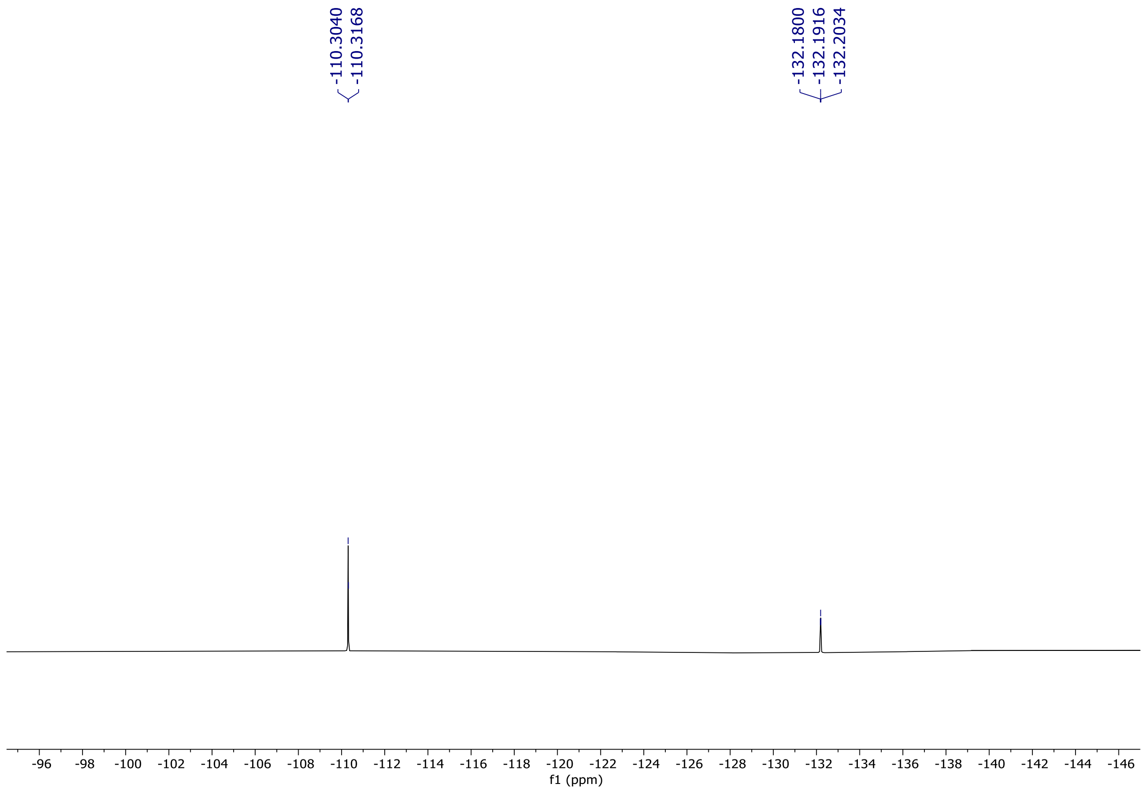

Figure S20. ^{19}F NMR spectrum (470 MHz) of compound **6** in DMF-d_7 at 298 K.

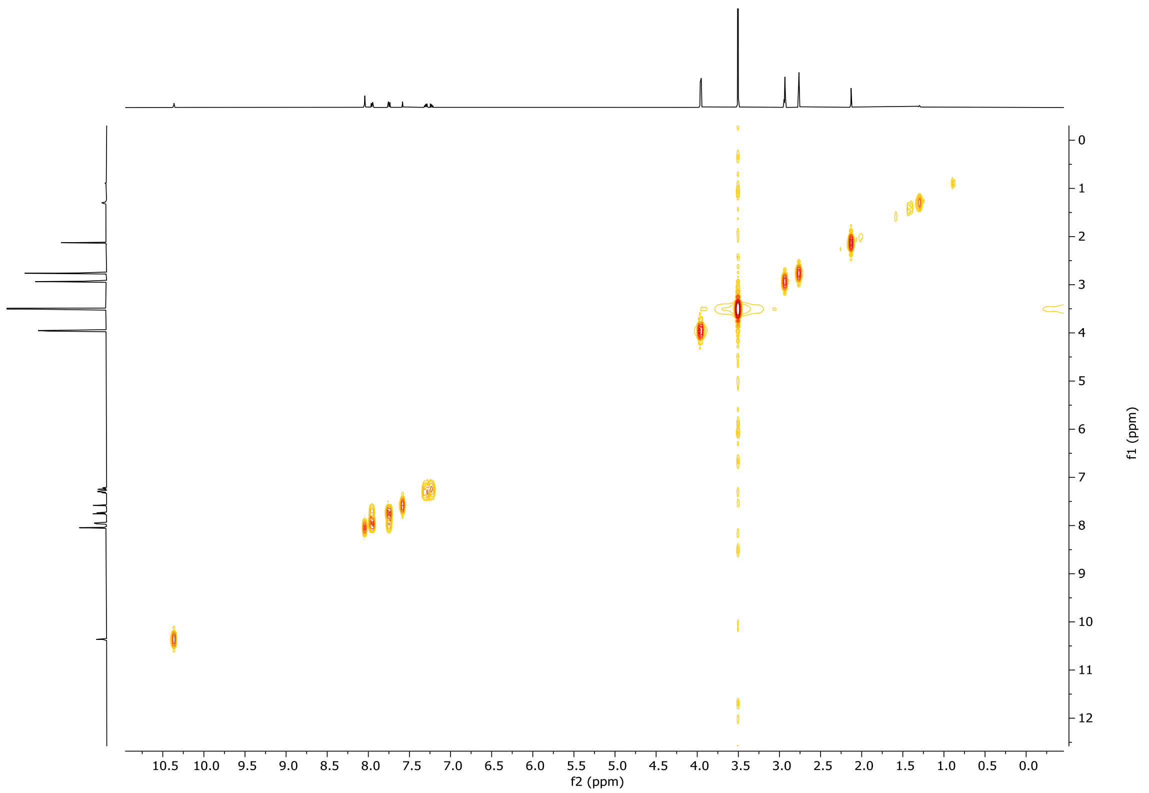

Figure S21. ^{1}H-^{1}H COSY spectrum (500 MHz) of compound **6** in DMF-d_7 at 298 K.

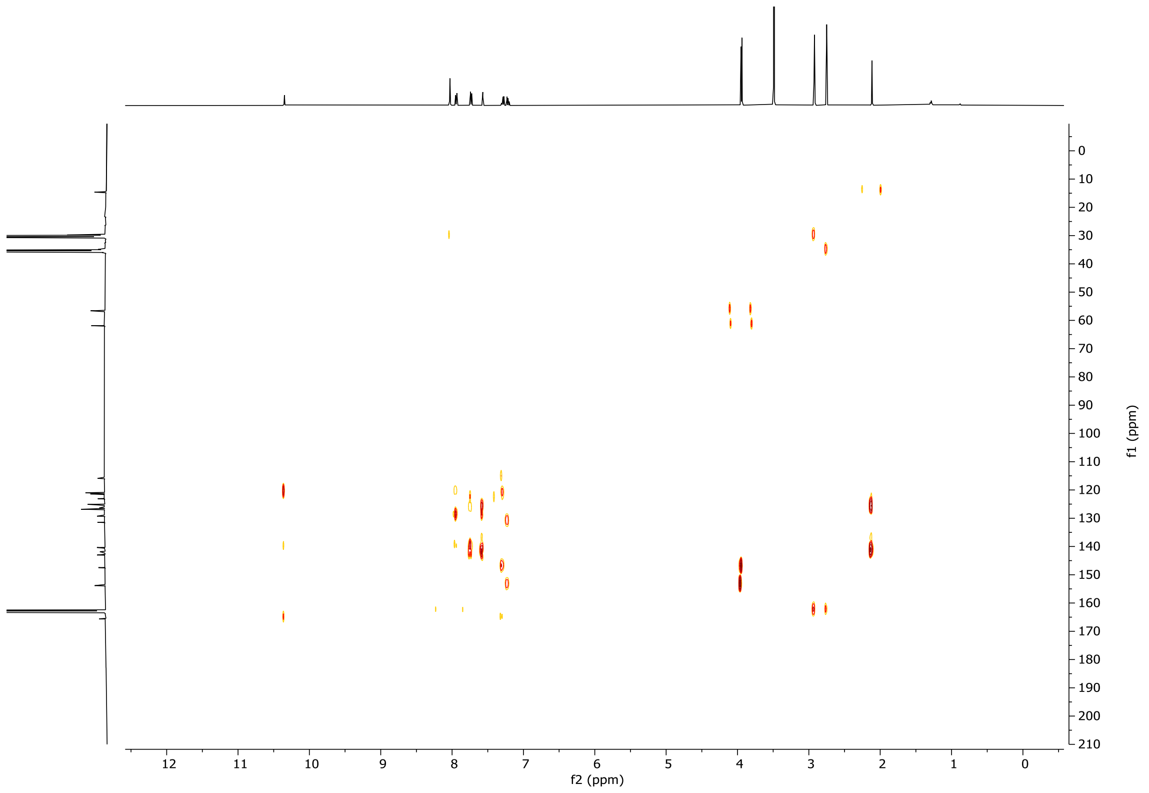

Figure S22. ^{1}H-^{13}C HMC spectrum (500 MHz) of compound **6** in DMF-d_7 at 298 K.

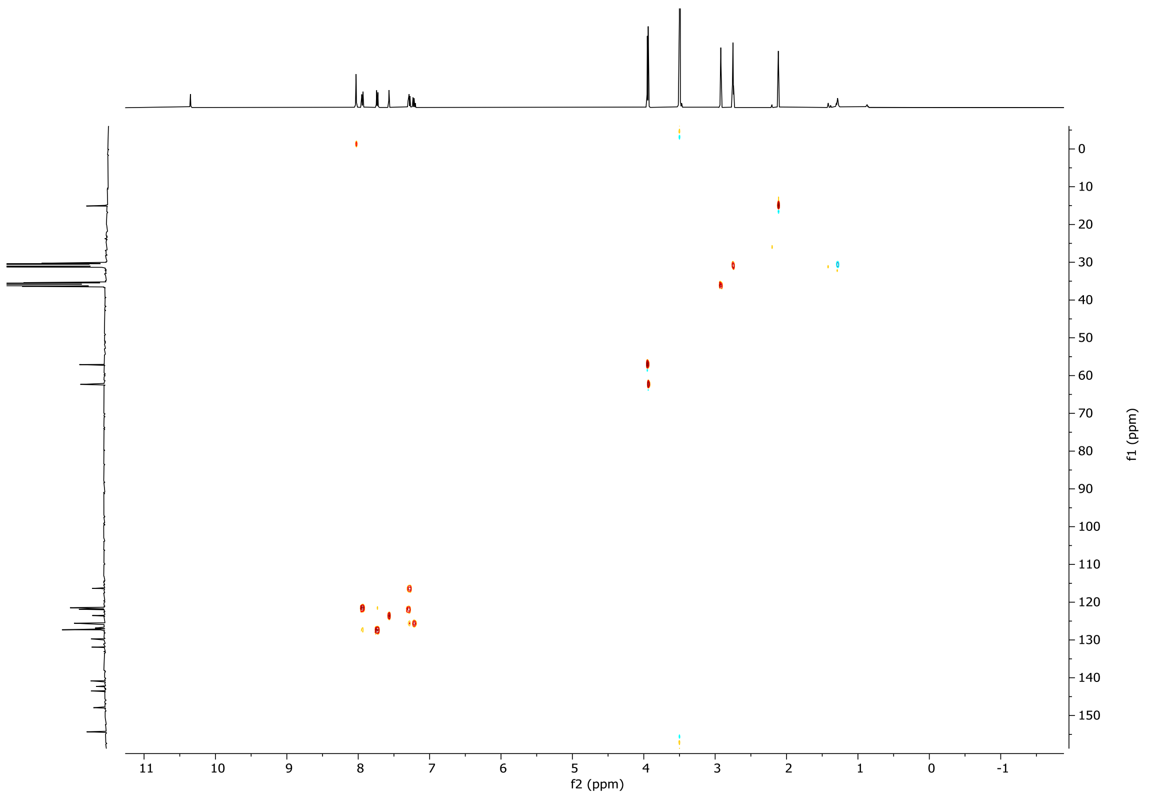

Figure S23. ^{1}H-^{13}C HSQC spectrum (500 MHz) of compound **6** in DMF-d_7 at 298 K

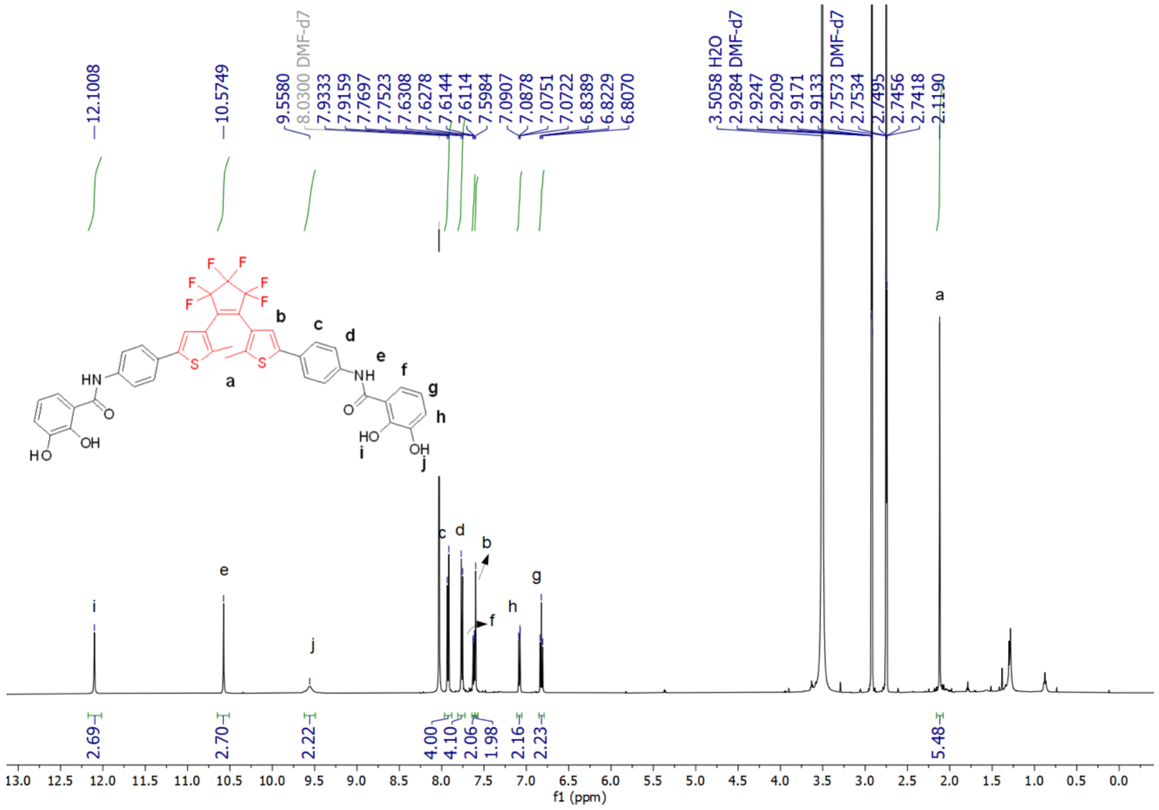

Figure S24. ^{1}H NMR spectrum (500 MHz) of compound **7** in DMF-d_7 at 298 K.

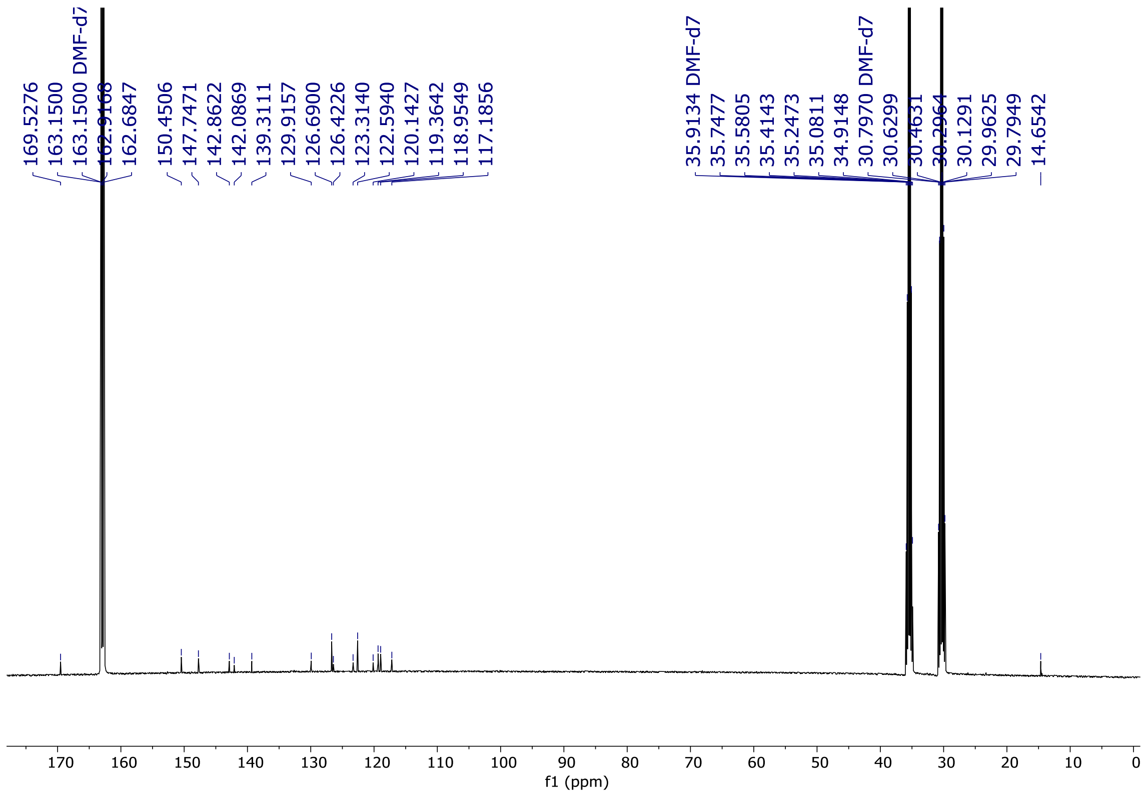

Figure S25. ^{13}C NMR spectrum (126 MHz) of compound **7** in DMF-d_7 at 298 K.

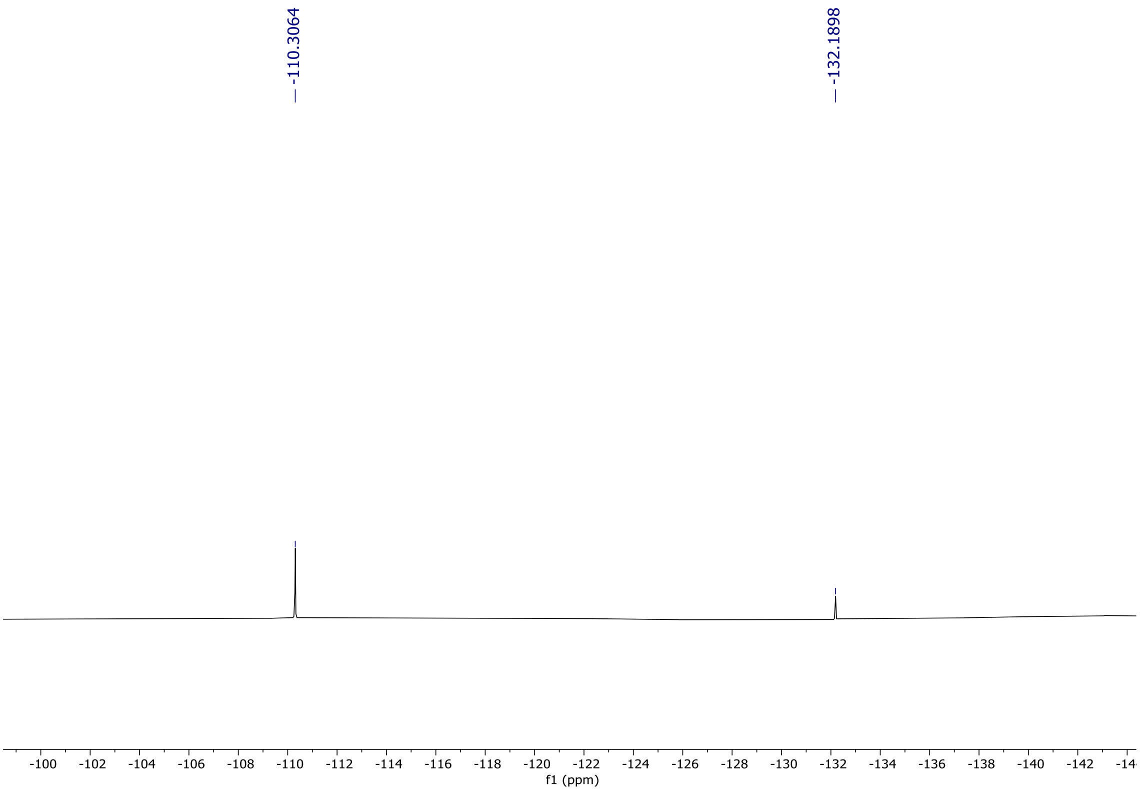

Figure S26. ^{19}F NMR spectrum (470 MHz) of compound **7** in DMF-d$_7$ at 298 K.

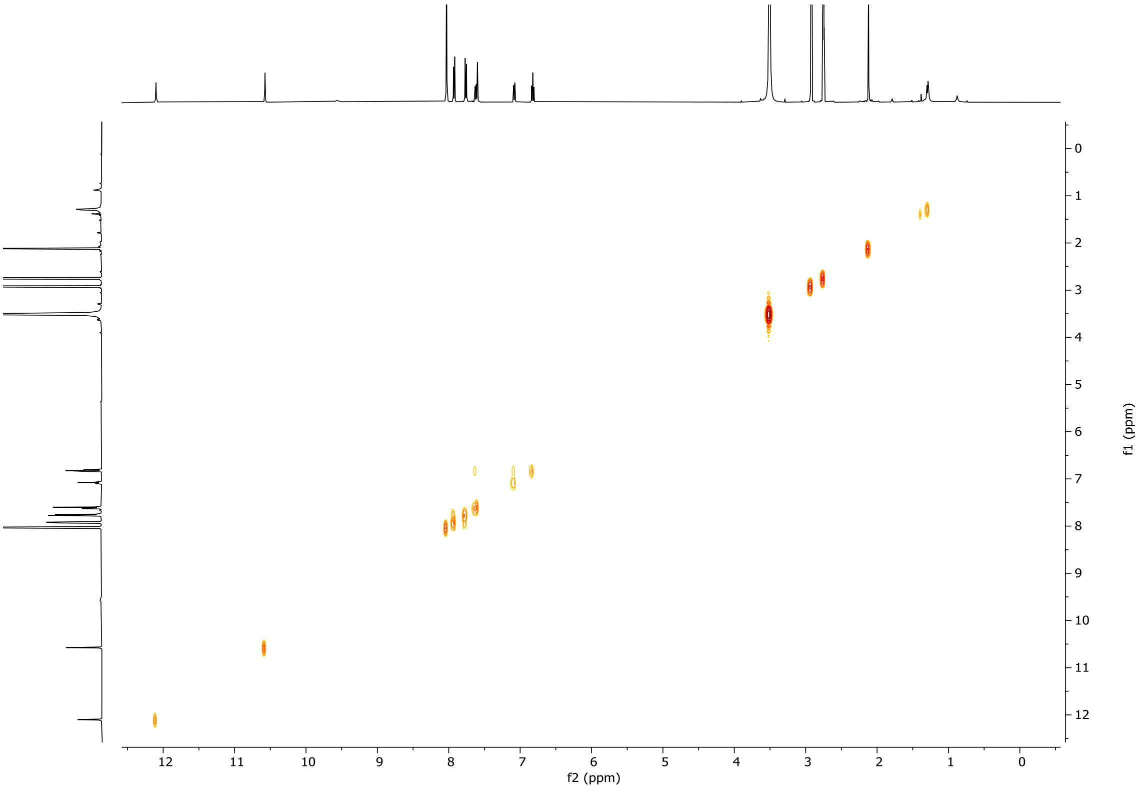

Figure S27. ^{1}H-^{1}H COSY spectrum (500 MHz) of compound **7** in DMF-d_7 at 298 K.

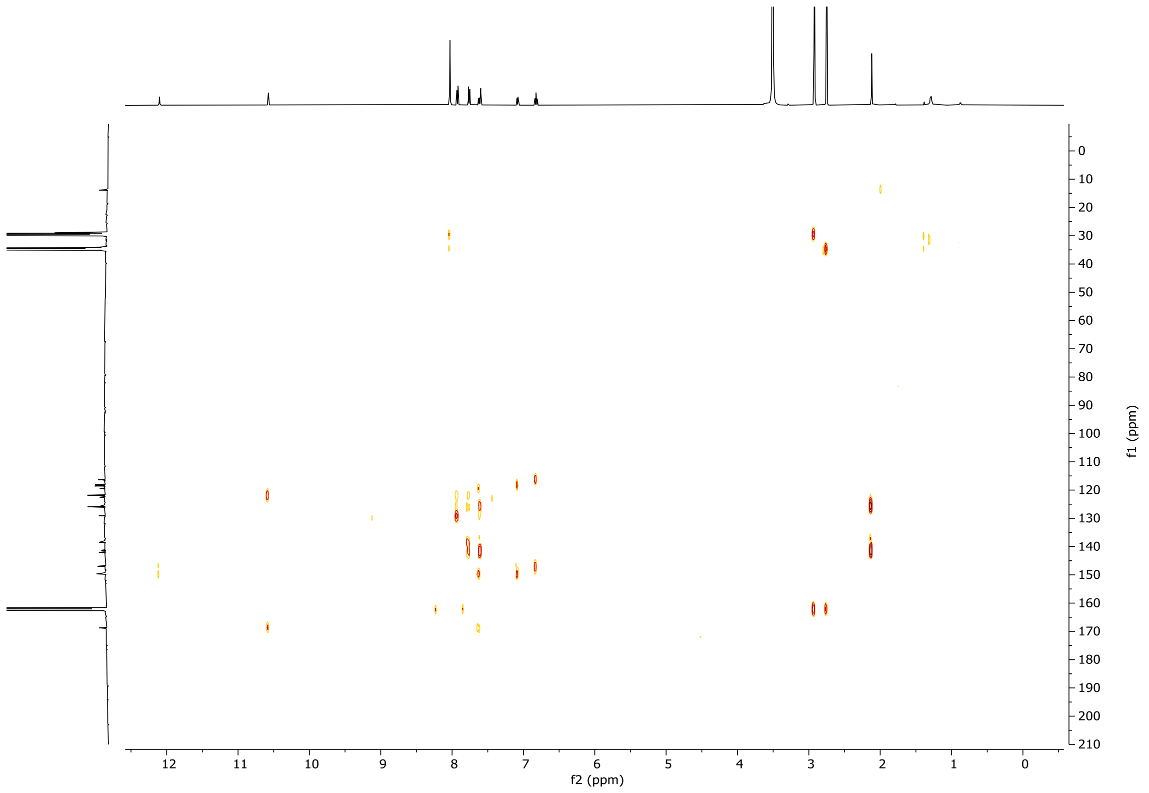

Figure S28. ^{1}H-^{13}C HMBC spectrum (500 MHz) of compound **7** in DMF-d_7 at 298 K.

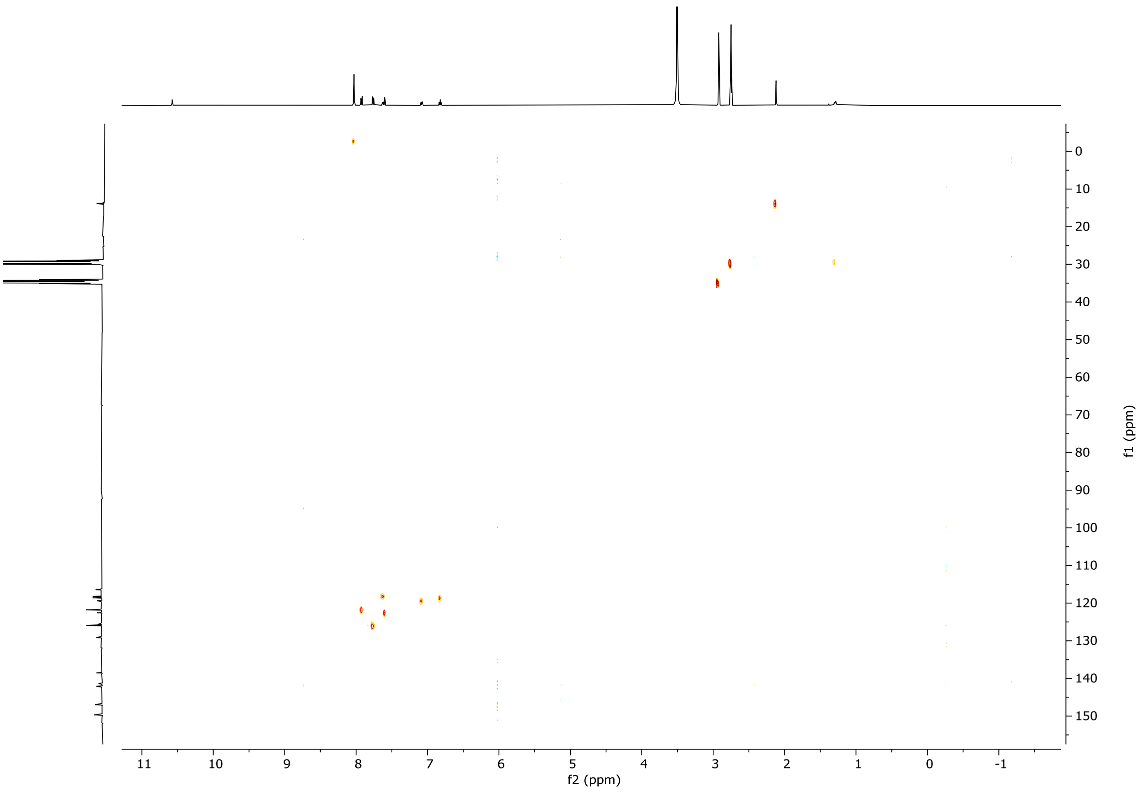

Figure S29. ^{1}H-^{13}C HSQC spectrum (500 MHz) of compound **7** in DMF-d$_7$ at 298 K.

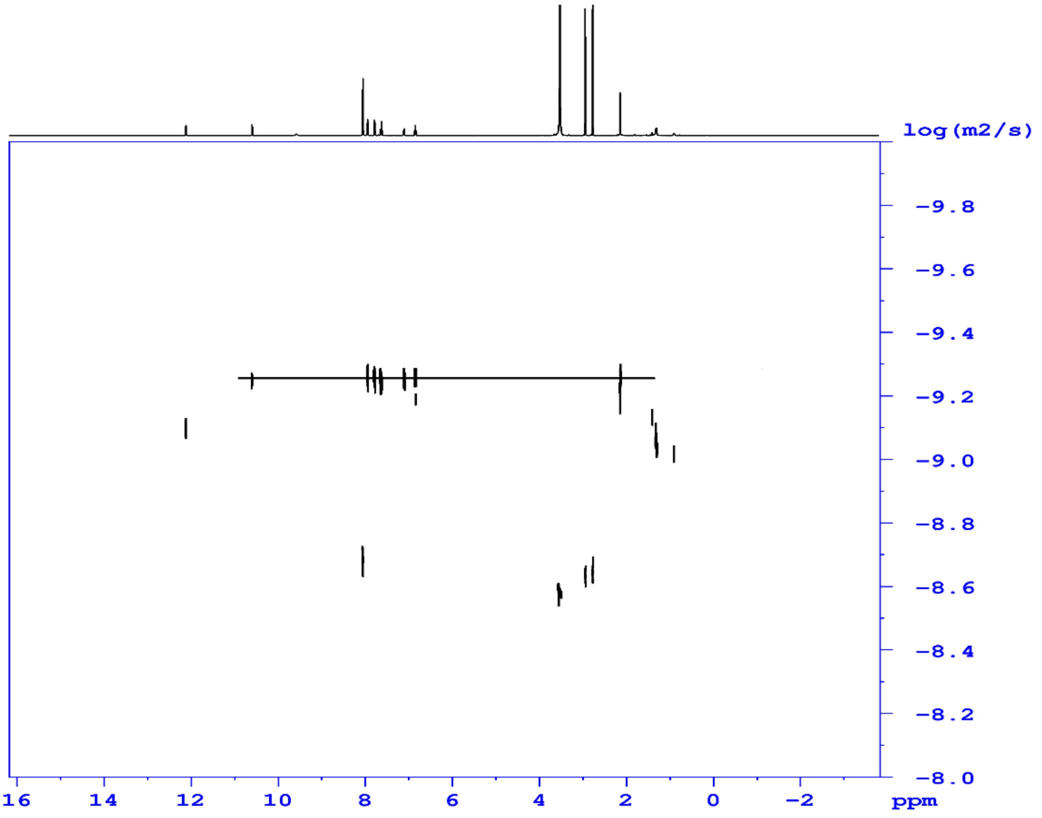

Figure S30. Full range DOSY spectrum (500 MHz) of compound **7** in DMF-d$_7$ at 298 K. D =$7.4\cdot10^{-10}$ m^2 s^{-1}.

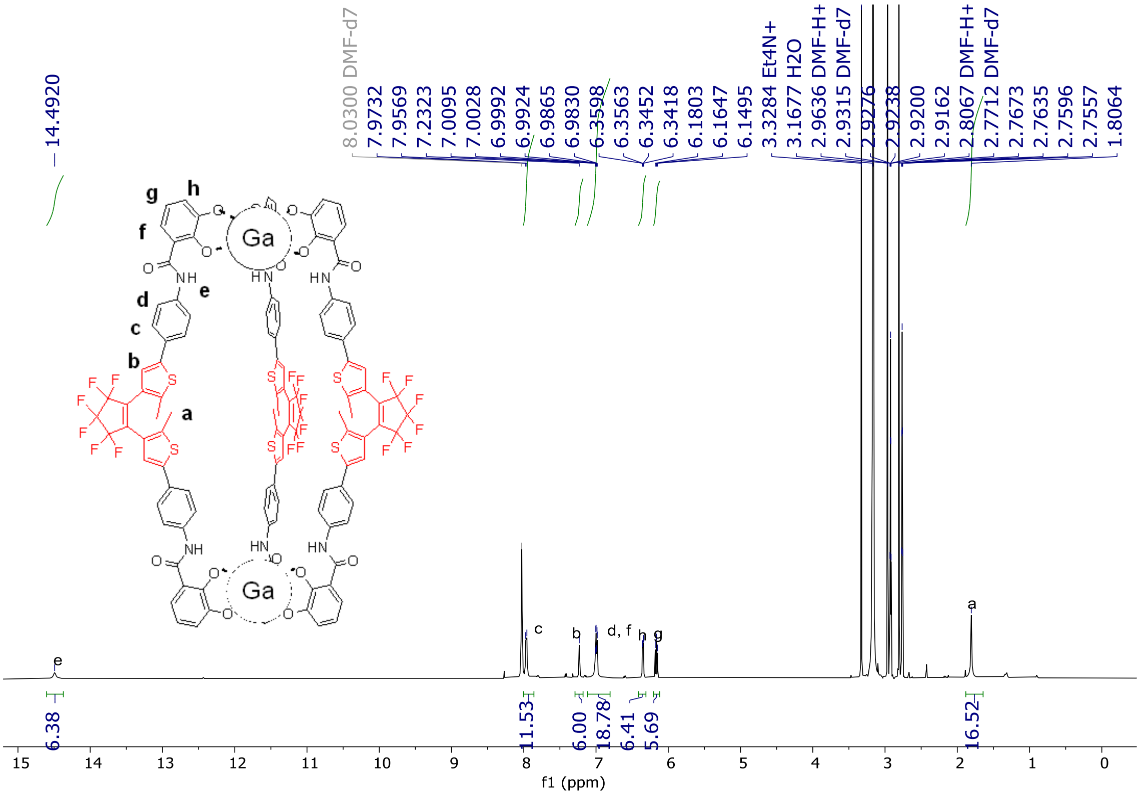

Figure S31. ^{1}H NMR spectrum (500 MHz) of compound $[Ga_2\mathbf{7}_3]^{6-}$ in DMF-d_7 at 348 K.

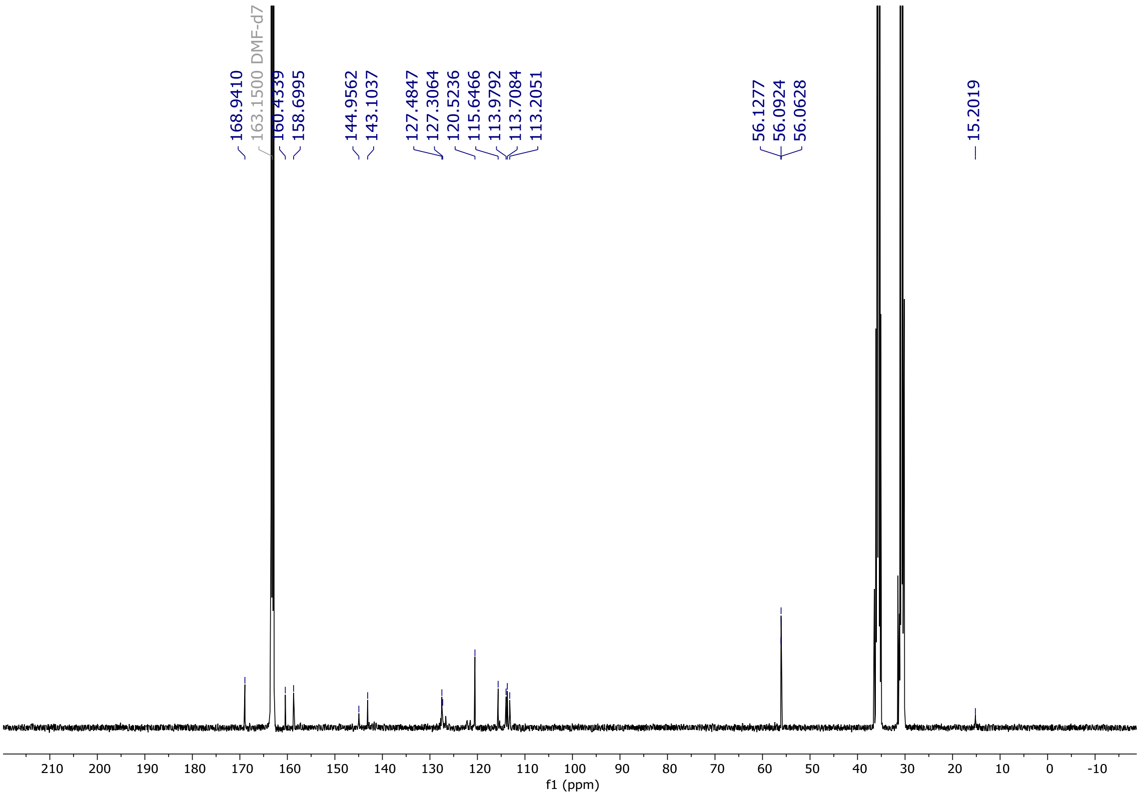

Figure S32. ^{13}C NMR spectrum (126 MHz) of compound $[Ga_2\mathbf{7}_3]^{6-}$ in DMF-d_7 at 348 K.

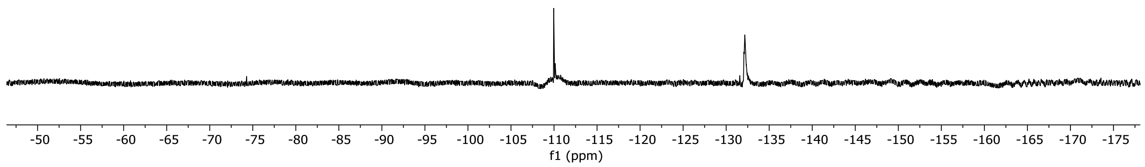

Figure S33. ^{19}F NMR spectrum (470 MHz) of compound $[Ga_2\mathbf{7}_3]^{6-}$ in DMF-d_7 at 348 K.

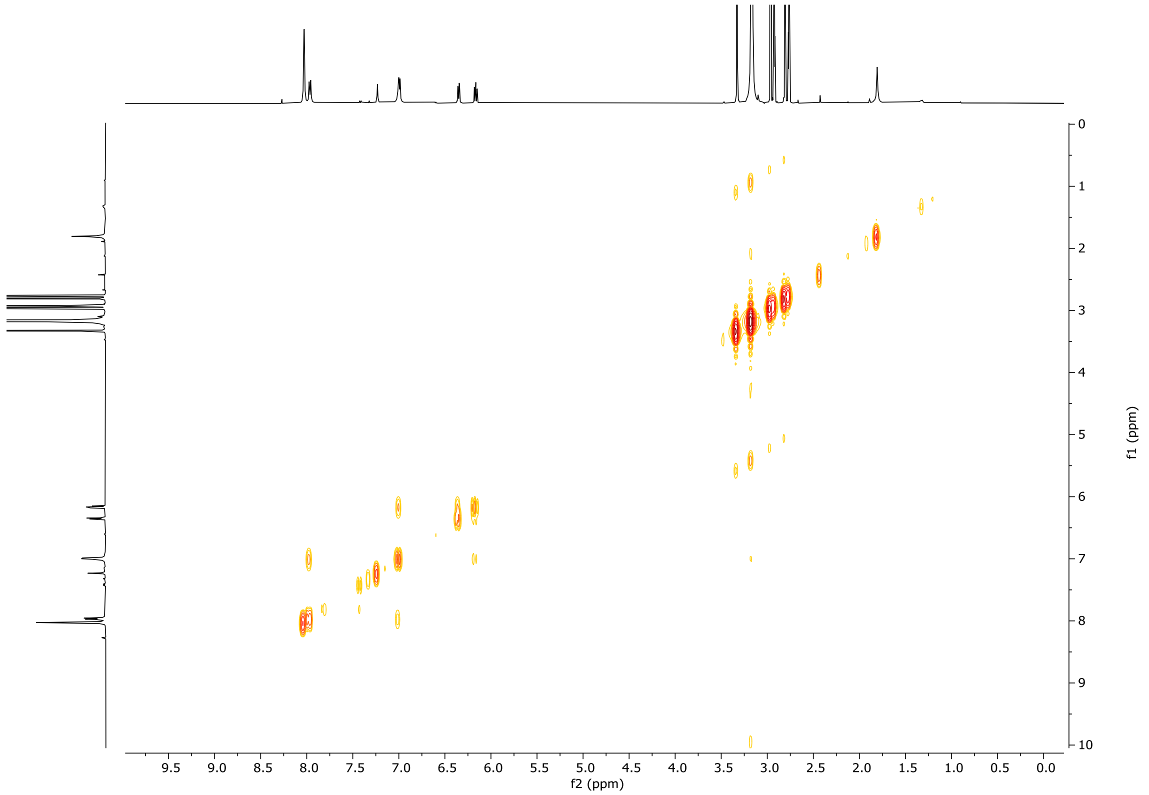

Figure S34. ^{1}H-^{1}H COSY spectrum (500 MHz) of compound $[Ga_2\mathbf{7}_3]^{6-}$ in DMF-d_7 at 348 K.

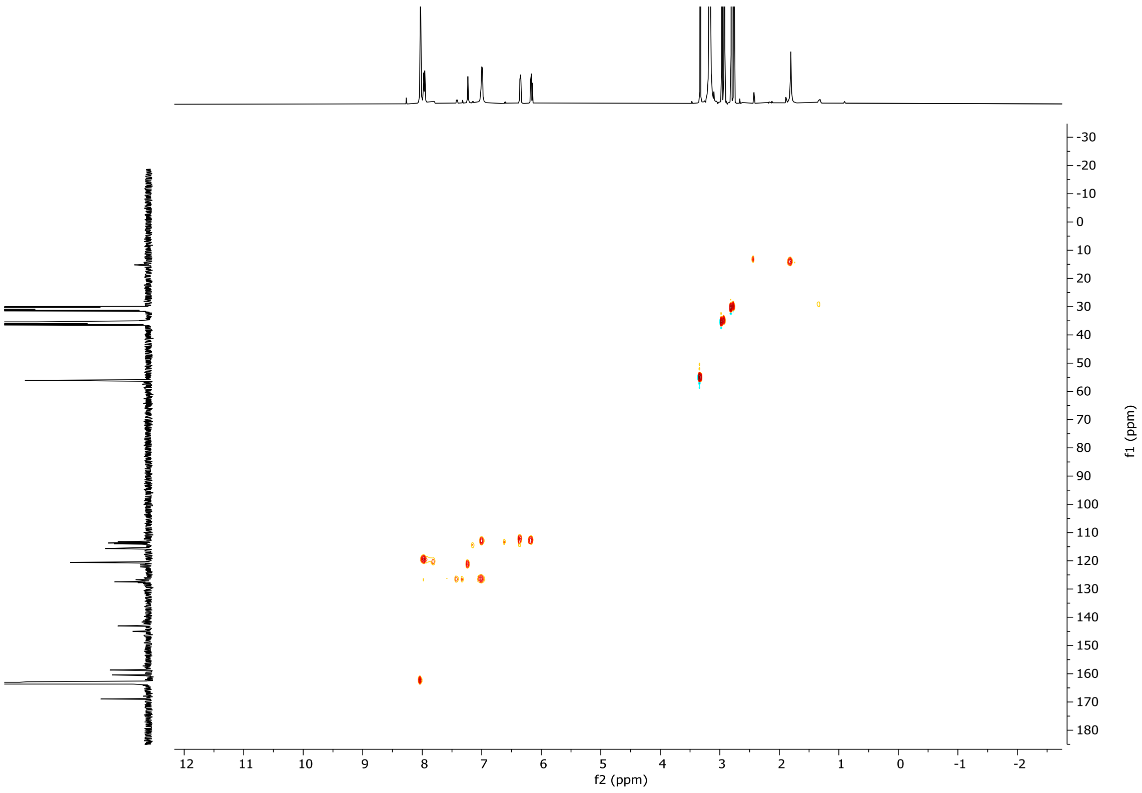

Figure S35. ^{1}H-^{13}C HSQC spectrum (500 MHz) of compound $[Ga_2\mathbf{7}_3]^{6-}$ in DMF-d_7 at 348 K.

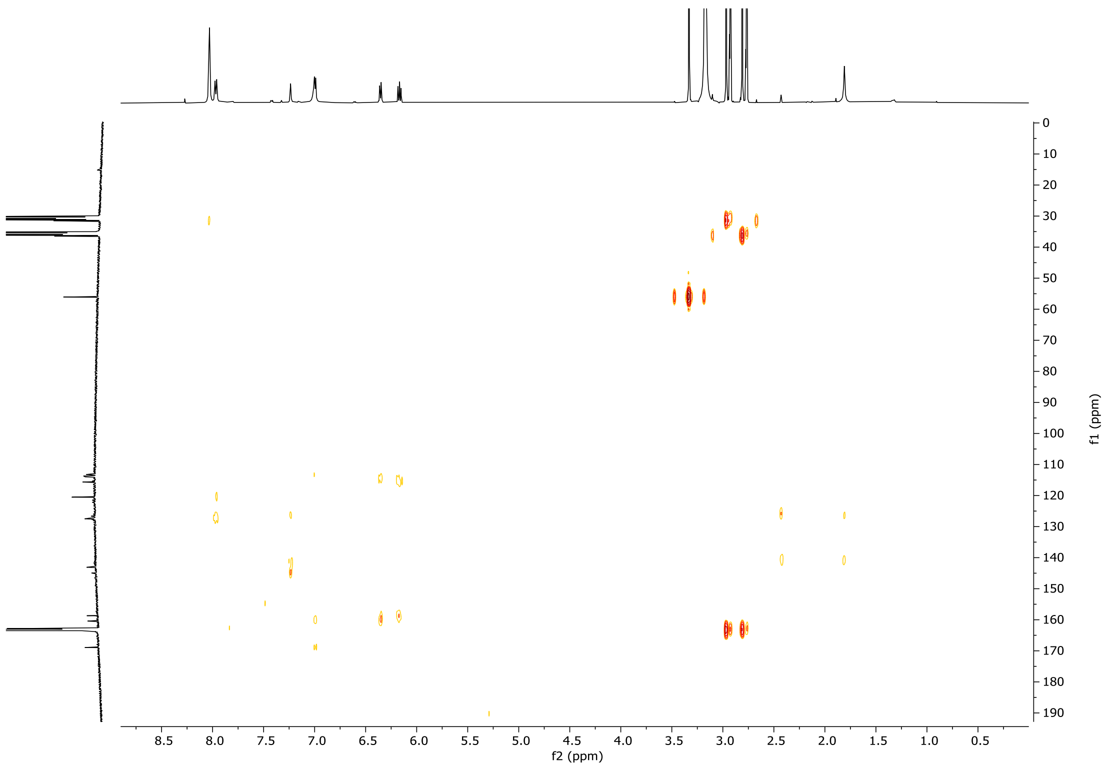

Figure S36. ^{1}H-^{13}C HMBC spectrum (500 MHz) of compound $[Ga_2\mathbf{7}_3]^{6-}$ in DMF-d_7 at 348 K.

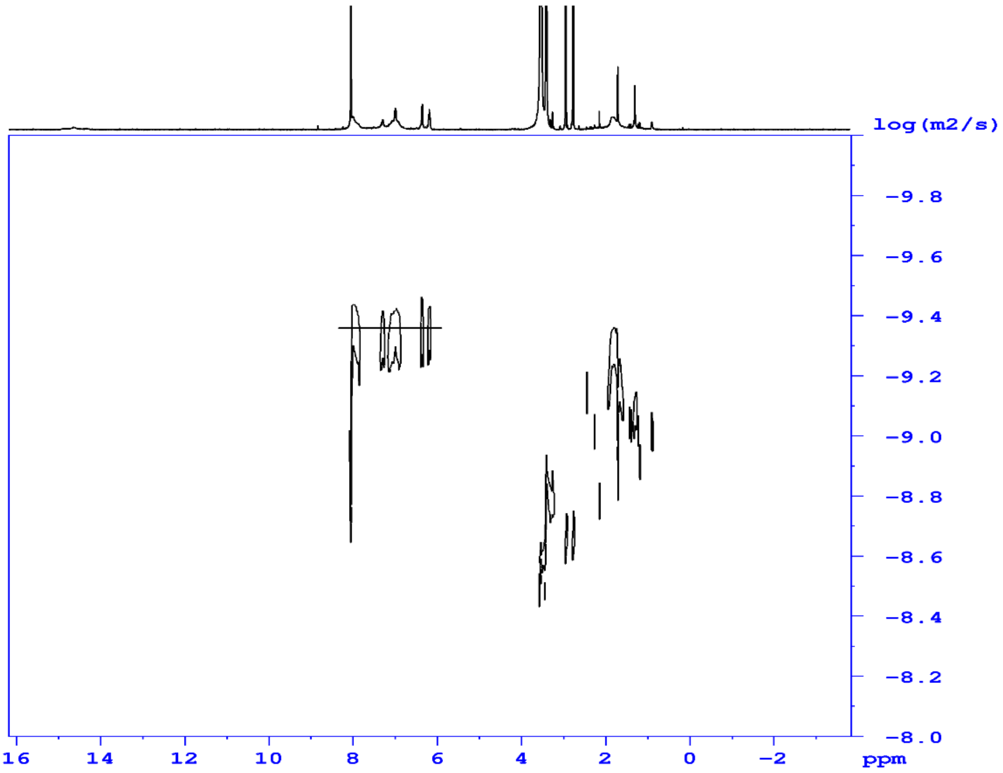

Figure S37. DOSY spectrum (500 MHz) of compound $[Ga_2\mathbf{7}_3]^{6-}$ in DMF-d_7 at 298 K. $D = 4.0\cdot10^{-10}$ m^2 s^{-1}.

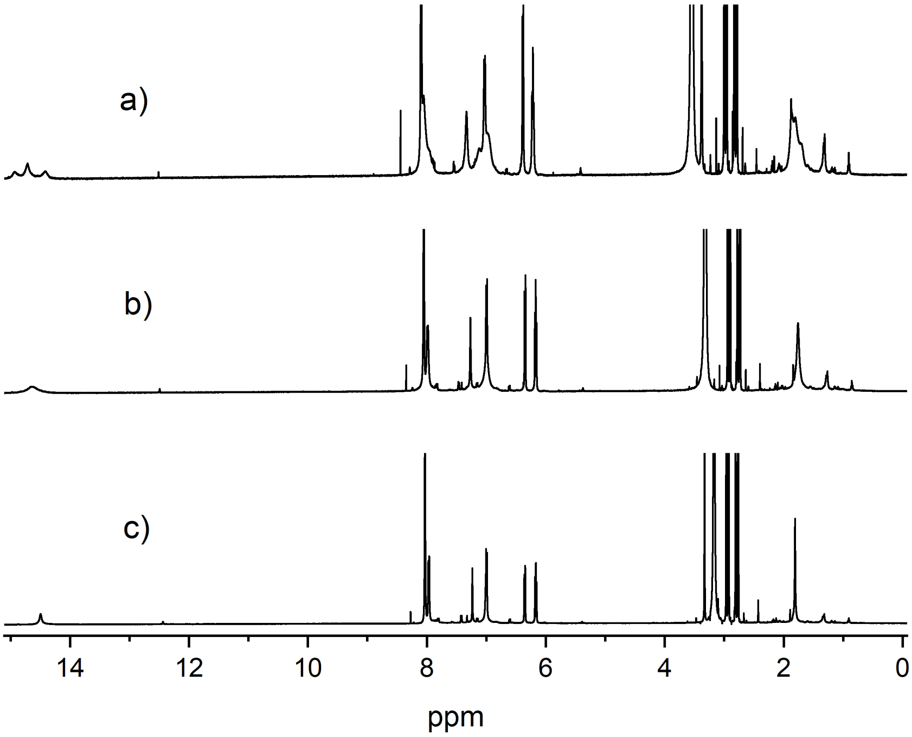

Figure S38. ^{1}H NMR spectrum (500 MHz) of compound $[Ga_2\mathbf{7}_3]^{6-}$ in DMF-d_7 a) 298 K, b) 323 K and c) 348 K.

2. Characterization by HRMS (ESI-QTOF)

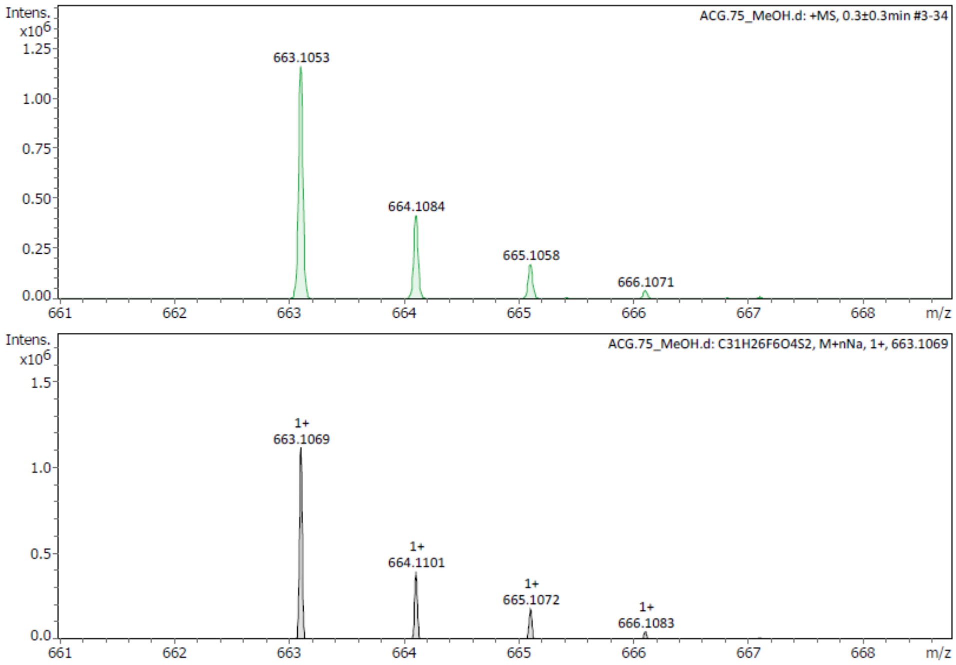

Figure S39. Top: HMRS (EI-Quadrupole) spectrum of compound **2** in methanol. Bottom: Calculated isotopic pattern (overlapped in black). Calculated for [M+Na]; $C_{31}H_{26}F_6O_4S_2$ 663.1069; found 663.1053.

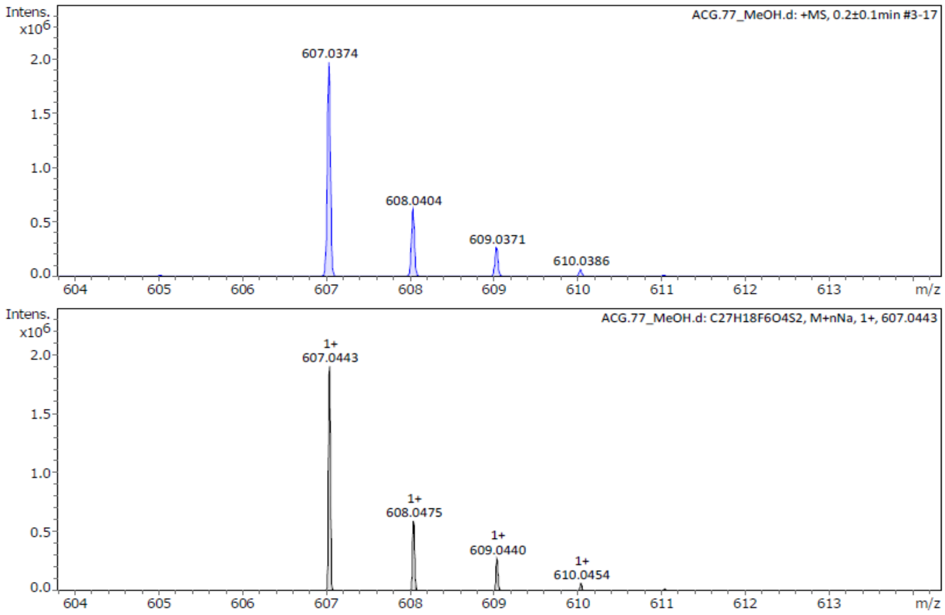

Figure S40. Top: HMRS (EI-Quadrupole) spectrum of of compound **3** in methanol. Bottom: Calculated isotopic pattern (overlapped in black). Calculated for [M+Na]; $C_{27}H_{18}F_6O_4S_2$ 607.0443; found 607.0374.

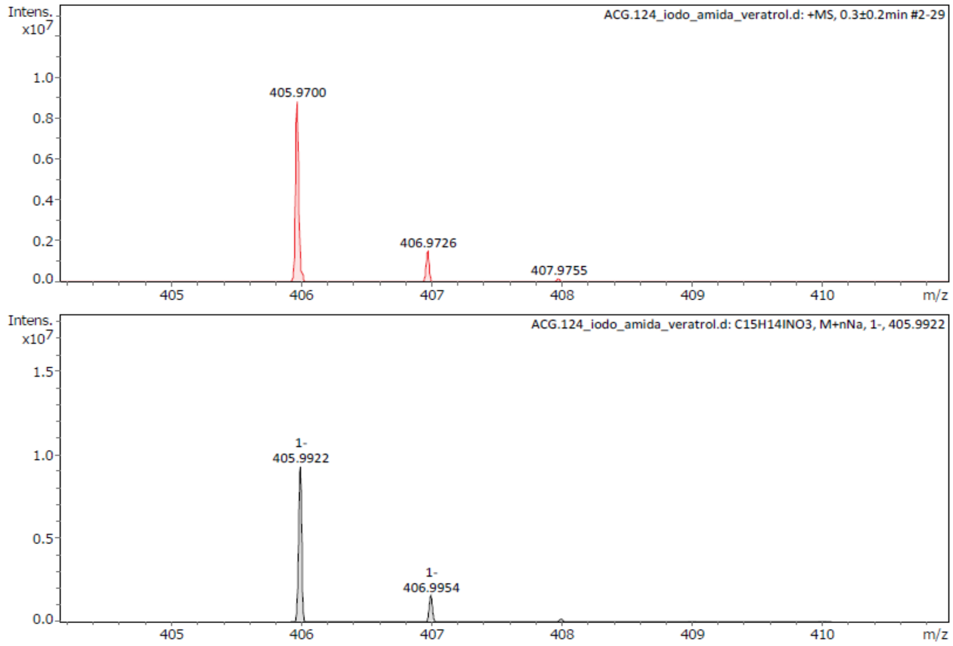

Figure S41. Top: HMRS (EI-Quadrupole) spectrum of compound **5** in methanol. Bottom: Calculated isotopic pattern (overlapped in black). Calculated for [M+Na]; $C_{15}H_{14}INO_3$ 405.9922; found 405.9700.

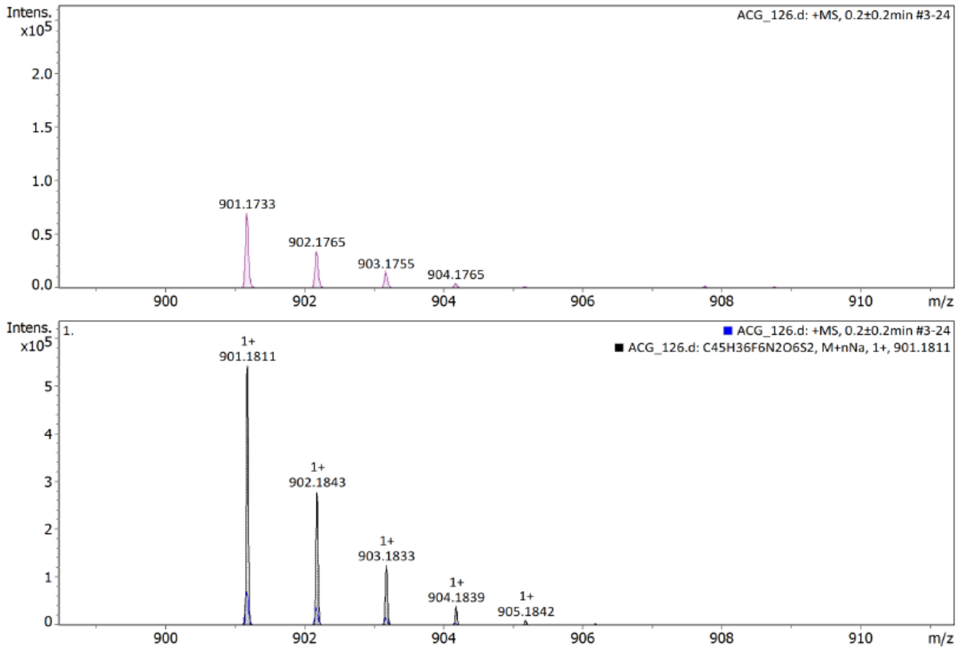

Figure S42. Top: HMRS (EI-Quadrupole) spectrum of compound **6** in methanol. Bottom: Calculated isotopic pattern (overlapped in black). Calculated for [M+Na]; $C_{45}H_{36}F_6O_6S_2Na$ 901.1811; found 901.1733.

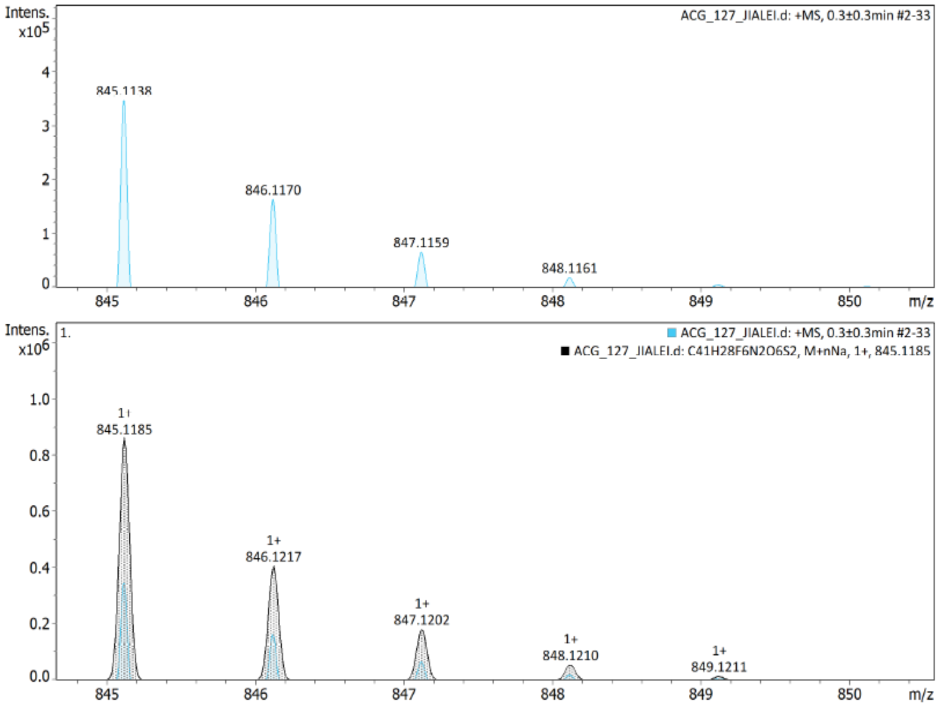

Figure S43. Top: HMRS (EI-Quadrupole) spectrum of of compound **7** in methanol. Bottom: Calculated isotopic pattern (overlapped in black). Calculated for [M+Na]; $C_{41}H_{28}F_6O_6S_2Na$ 845.1185; found 845.1138.

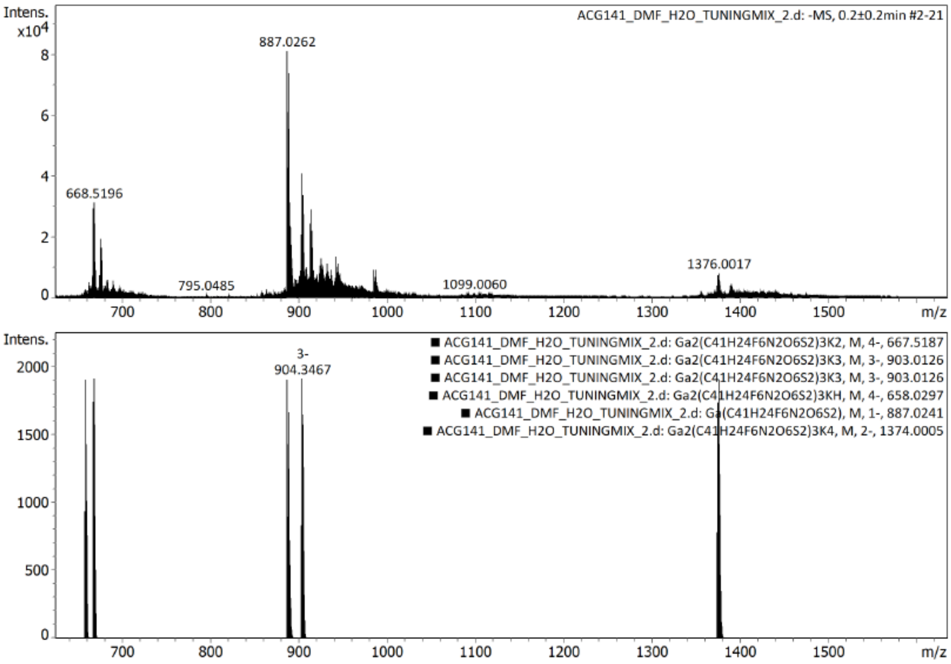

Figure S44. Top: HMRS (EI-Quadrupole) spectrum of $[Ga_2\mathbf{7}_3]^{6-}$ in DMF/H_2O. Bottom: Calculated isotopic pattern (overlapped in black) for all different ion pairs that were isolated.

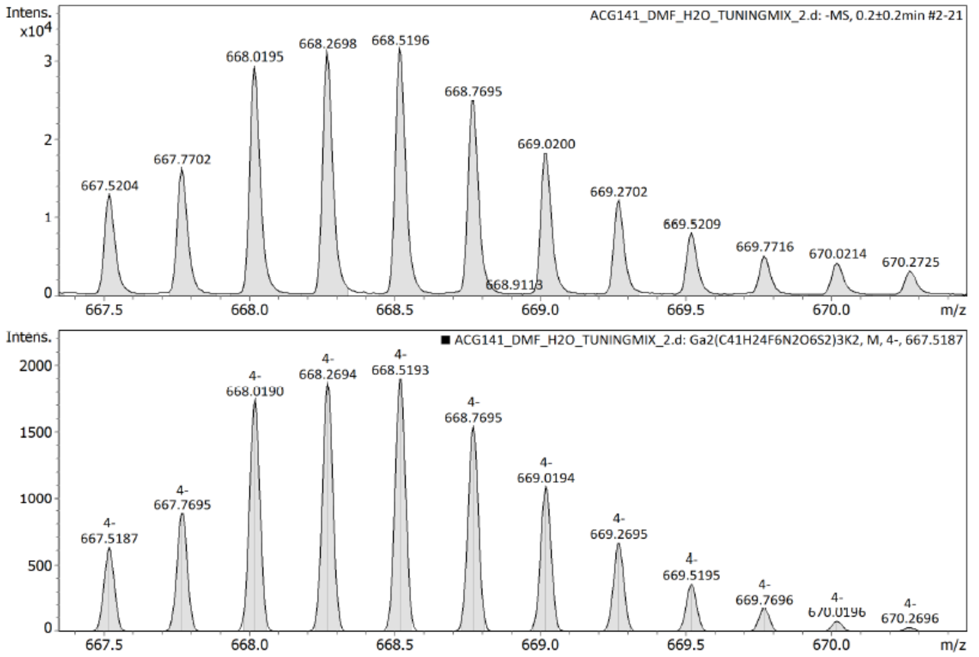

Figure S45. Top: HMRS (EI-Quadrupole) spectrum of $[Ga_2\mathbf{7}_3]^{6-}$ in DMF/H_2O. Bottom: Calculated isotopic pattern (overlapped in black). Calculated for $[M+K_2]^{4-}$; $Ga_2(C_{41}H_{24}F_6O_6S_2)_3K_2$ 668.5193; found 668.5196.

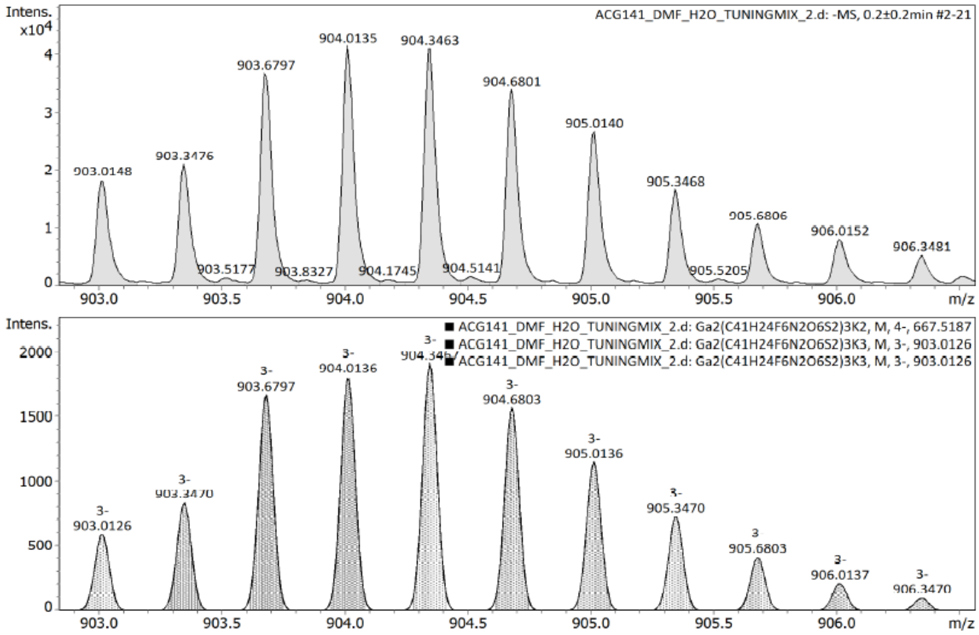

Figure S46. Top: HMRS (EI-Quadrupole) spectrum of $[Ga_2\mathbf{7}_3]^{6-}$ in DMF/H_2O. Bottom: Calculated isotopic pattern (overlapped in black). Calculated for $[M+K_3]^{-3}$; $Ga_2(C_{41}H_{24}F_6O_6S_2)_3K_3$ 904.3463; found 904.3467.

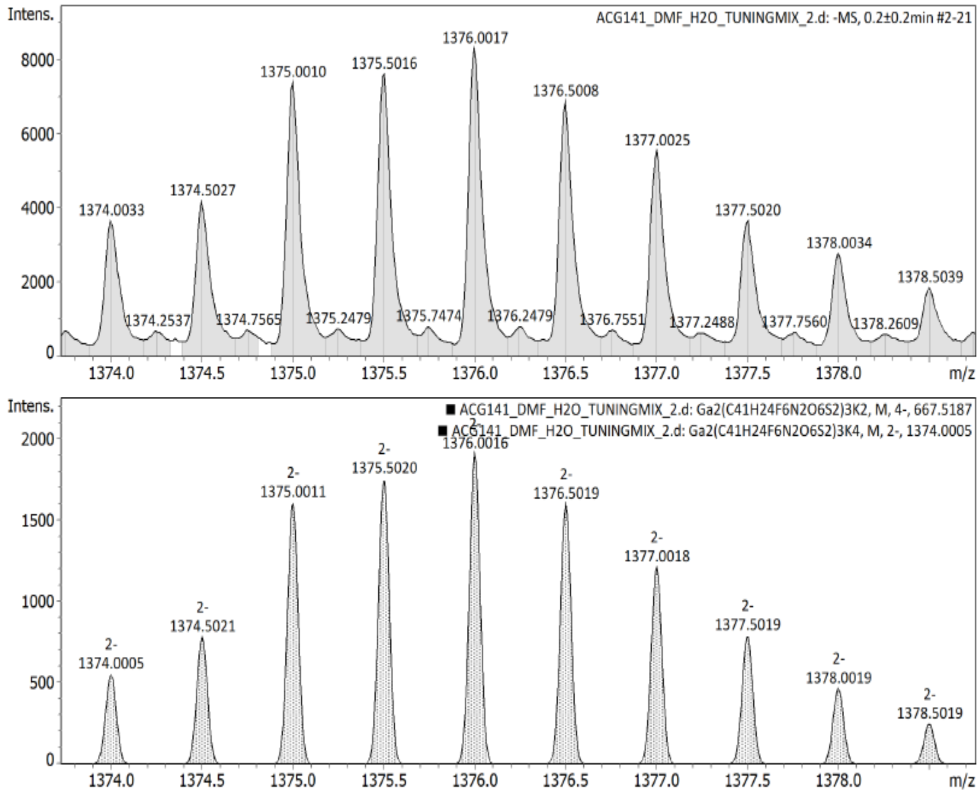

Figure S47. Top: HMRS (EI-Quadrupole) spectrum of $[Ga_2\mathbf{7}_3]^{6-}$ in DMF/H_2O. Bottom: Calculated isotopic pattern (overlapped in black). Calculated for $[M+K_4]^{-2}$; $Ga_2(C_{41}H_{24}F_6O_6S_2)_3K_4$ 1376.0016; found 1376.0017.

3. Photochemistry

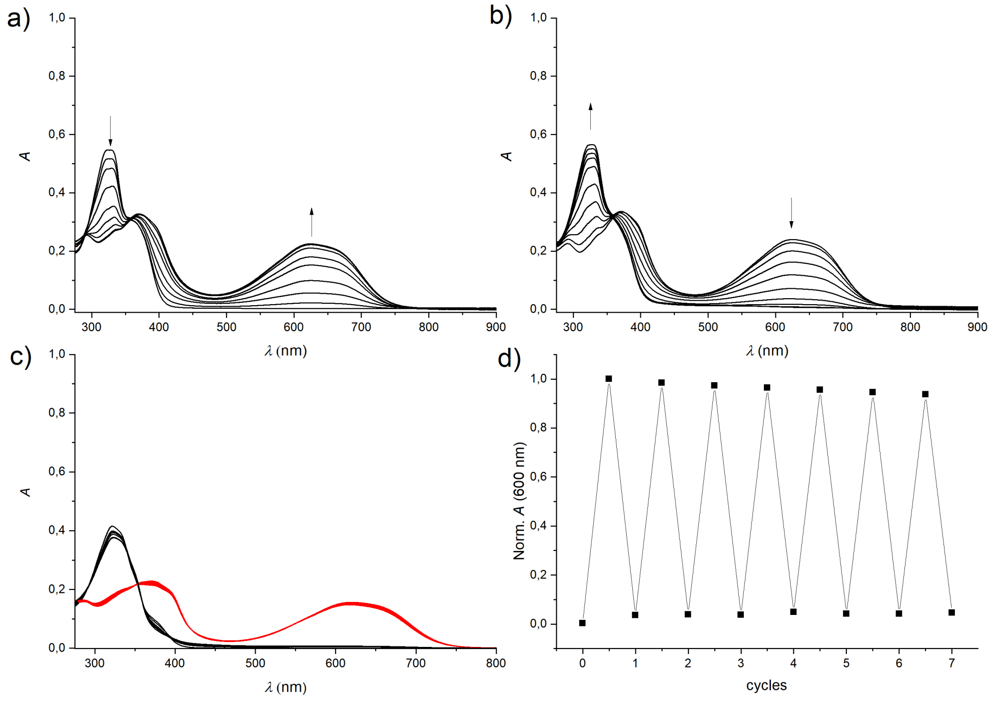

Figure S48. Photoswitching of DTE 7 (10 μM) in DMF. a) UV/vis absorption spectra upon irradiation at 365 nm (up to 300 s) of the ring-opened form, b) UV/vis absorption spectra upon irradiation at >495 nm (up to 270 s) of the ring-closed form, c) UV/vis absorption spectra upon alternating irradiation at 365 nm (red spectra) and >495 nm (black spectra), and d) cycling with observation of the absorbance at 600 nm of the ring-closed isomer.

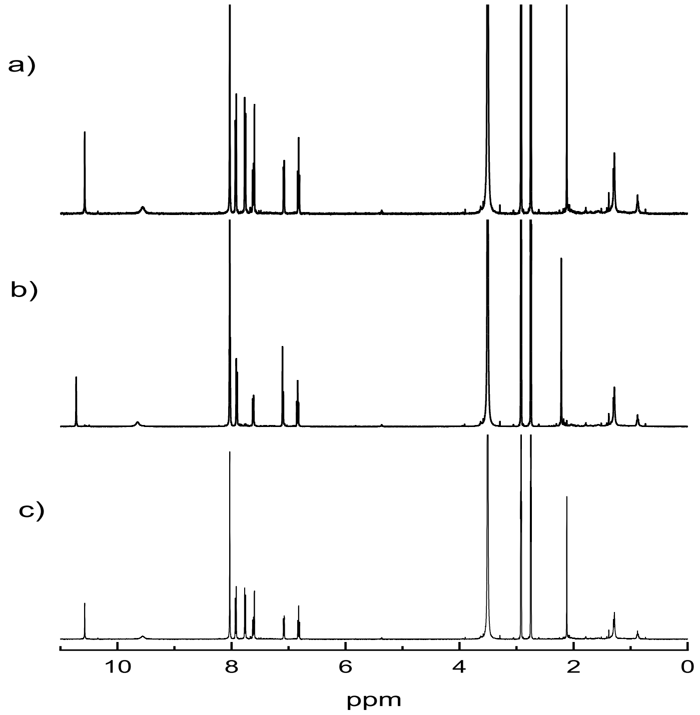

Figure S49. ^{1}H NMR spectra (500 MHz, 298 K, DMF-d_7) of (a) compound **7o**, (b) compound **7o** after irradiation 180 min at 365 nm, and (c) compound **7c** after irradiation 60 min at >495 nm (open → closed 100%, closed → open 100%).

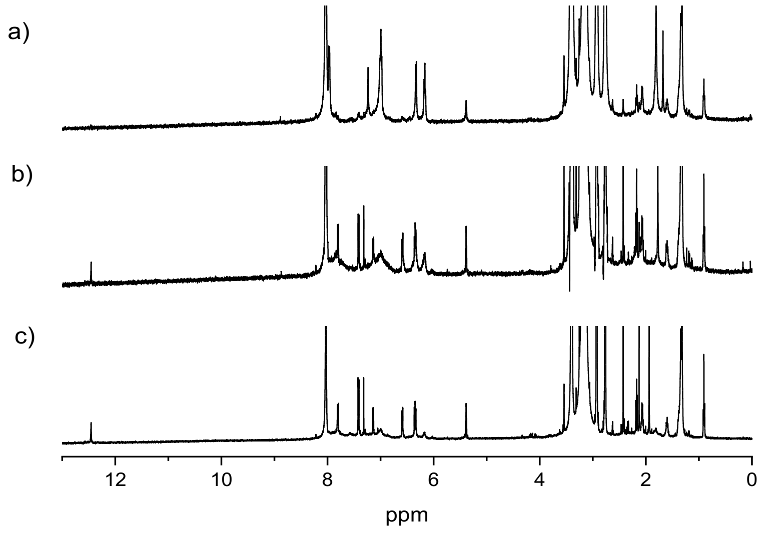

Figure S50. ^{1}H NMR spectra (500 MHz, 348 K, DMF-d_7) of (a) compound o-[Ga$_2$**7**$_3$]$^{6-}$, (b) compound o-[Ga$_2$**7**$_3$]$^{6-}$ after irradiation 240 min at 365 nm, and (c) compound c-[Ga$_2$**7**$_3$]$^{6-}$ after irradiation 120 min at >495 nm (open → closed 100%, closed → open 0%).

4. X-Ray crystallography

Selected crystallographic and refinement data

Table S1. Crystal data for **2** (CCDC 2364246)

Empirical formula	$C_{62}H_{52}F_{12}O_8S_4$	
Formula weight	1281.27	
Temperature	150.00 K	
Wavelength	0.71073 Å	
Crystal system	Triclinic	
Space group	P-1	
Unit cell dimensions	a = 8.61890(10) Å	α = 94.2030(10)°.
	b = 13.7427(2) Å	β = 93.2950(10)°.
	c = 25.1152(4) Å	γ = 98.5680(10)°.
Volume	2926.64(7) $Å^3$	
Z	2	
Density (calculated)	1.454 Mg/m^3	
Absorption coefficient	0.256 mm^{-1}	
F(000)	1320	
Crystal size	0.18 x 0.16 x 0.14 mm^3	
Theta range for data collection	1.504 to 30.531°.	
Index ranges	-12<=h<=12, -19<=k<=19, -35<=l<=35	
Reflections collected	191234	
Independent reflections	17870 [R(int) = 0.0425]	
Completeness to theta = 25.242°	99.8 %	
Absorption correction	None	
Refinement method	Full-matrix least-squares on F^2	

Data / restraints / parameters	17870 / 0 / 787
Goodness-of-fit on F^2	1.101
Final R indices [I>2sigma(I)]	R1 = 0.0563, wR2 = 0.1345
R indices (all data)	R1 = 0.0683, wR2 = 0.1410
Extinction coefficient	n/a
Largest diff. peak and hole	1.307 and -0.575 e.Å^{-3}

Additional details for 2: This compound crystallised in the triclinic space group *P*-1 and two molecules were found in the asymmetric unit.

Table S2. Crystal data for **6** (CCDC 2364247)

Empirical formula	$C_{45}H_{36}F_6N_2O_6S_2$	
Formula weight	878.88	
Temperature	240.02(10) K	
Wavelength	1.54184 Å	
Crystal system	Monoclinic	
Space group	I 1 2/a 1	
Unit cell dimensions	a = 15.67936(14) Å	α = 90°.
	b = 9.31867(9) Å	β = 93.7519(8)°.
	c = 28.2570(3) Å	γ = 90°.
Volume	4119.80(6) Å^3	
Z	4	
Density (calculated)	1.417 Mg/m^3	
Absorption coefficient	1.861 mm^{-1}	
F(000)	1816	
Crystal size	0.324 x 0.161 x 0.131 mm^3	
Theta range for data collection	3.135 to 74.615°.	
Index ranges	-19<=h<=19, -11<=k<=11, -35<=l<=35	
Reflections collected	82176	
Independent reflections	4228 [R(int) = 0.0547]	
Completeness to theta = 67.684°	100.0 %	
Absorption correction	Gaussian	
Max. and min. transmission	1.000 and 0.484	
Refinement method	Full-matrix least-squares on F^2	
Data / restraints / parameters	4228 / 92 / 302	

Goodness-of-fit on F^2	1.070
Final R indices [I>2sigma(I)]	R1 = 0.0870, wR2 = 0.2571
R indices (all data)	R1 = 0.0900, wR2 = 0.2591
Extinction coefficient	n/a
Largest diff. peak and hole	1.373 and -0.491 e.Å^{-3}

Additional details for 6: This compound was crystallised and collected several times with Mo-Kα (0.71073 Å) and Cu-Kα (1.54184 Å) at different temperatures ranging 298-100 K). The crystals showed a phase transition below 260 K, so it was best collected at 240 K in the monoclinic *I*2/a space group with half molecule within the asymmetric unit. Two F atoms were modelled as disordered over two positional main domains.

Supplementary X-ray figures and metrics

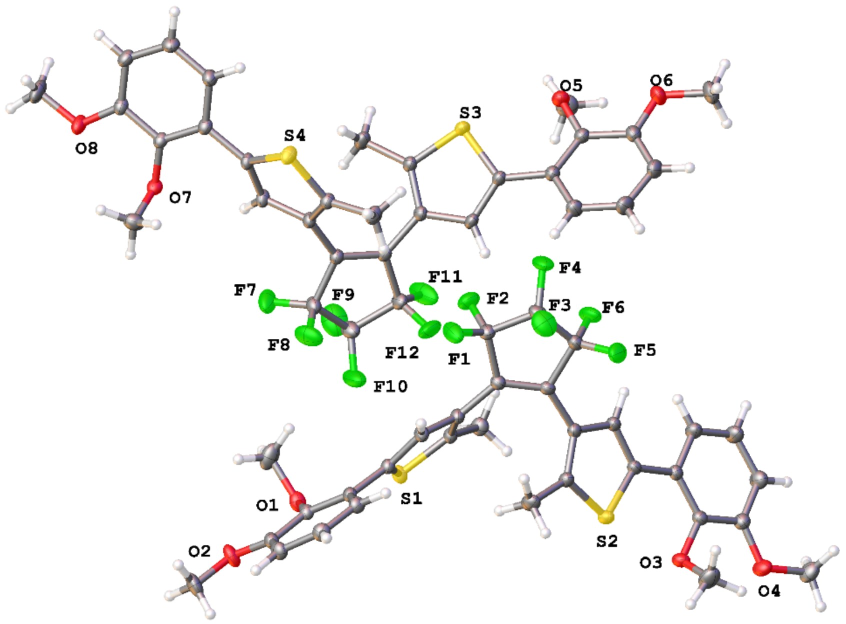

Figure S51. Molecular structure of **2** showing two independent molecules within the asymmetric unit with displacement ellipsoids at 50% probability.

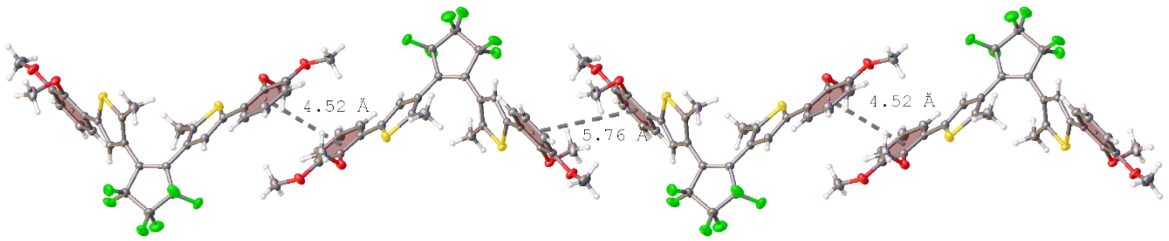

Figure S52. Packing diagram of **2** illustrating the π(arene)···π(arene) interactions between two terminal $C_6H_3(OMe)_2$ rings from two distinct molecules. The calculated distance between the parallel planes formed by $C_6H_3(OMe)_2$ rings from neighbouring molecules alternates from 3.52 and 5.76 Å. This results with π(arene)···π(arene) zig-zag chains.

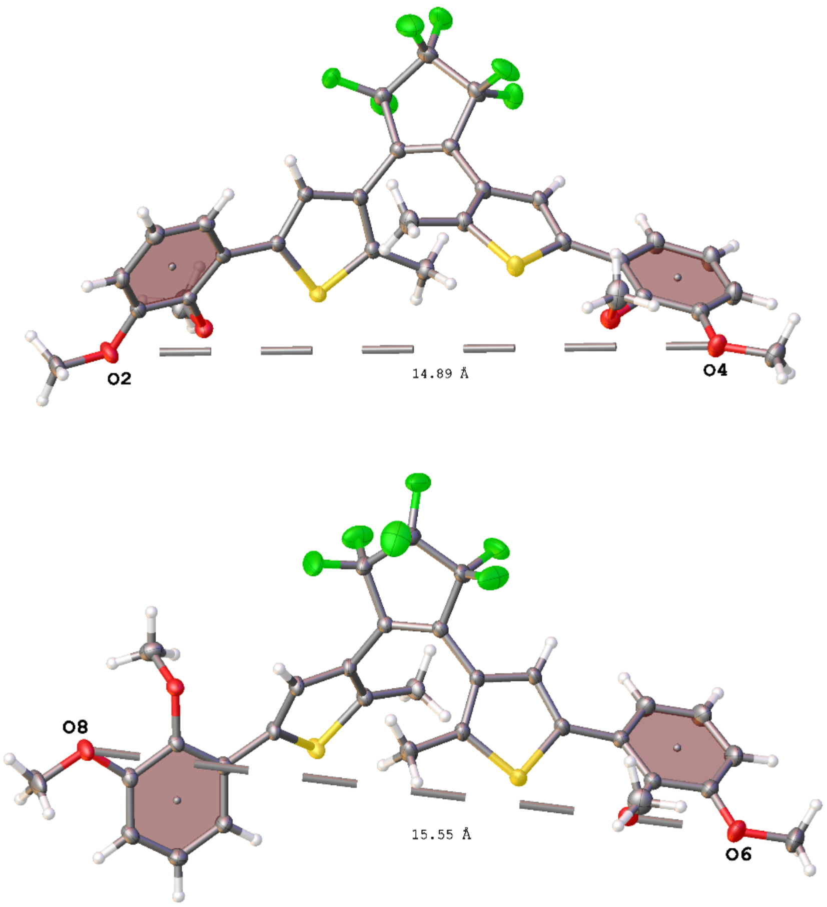

Figure S53. Interatomic O···O distances within the two independent molecules of **2** illustrating the O4···O2 of 14.89 Å (top) and O6···O8 of 15.55 Å (bottom) distances.

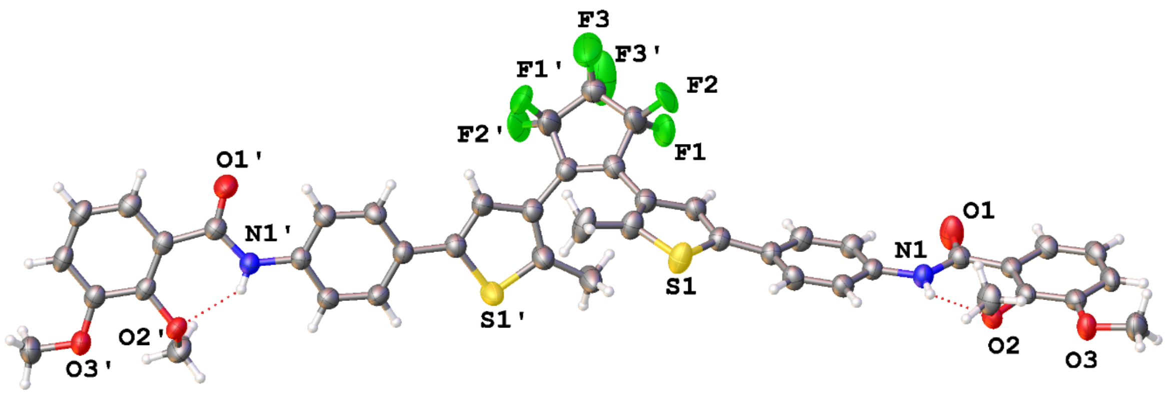

Figure S54. Molecular structure of **6** showing one complete molecule within the asymmetric unit (only half independent molecule is present within the asymmetric unit) with displacement ellipsoids at 50% probability. The atoms labelled with (') have been generated with the symmetry operator ½–x, y, 1–z.

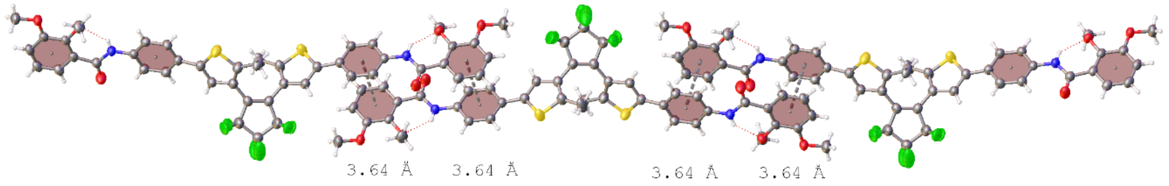

Figure S55. Packing diagram of **6** illustrating the alternate π(arene)···π(arene) interactions between two terminal $C_6H_3(OMe)_2$ and iternal C_6H_4 rings from two distinct molecules. The calculated distance between the parallel planes formed by $C_6H_3(OMe)_2$ and iternal C_6H_4 rings from neighbouring molecules are 3.64 Å. This results with π(arene)···π(arene) nearly linear chains.

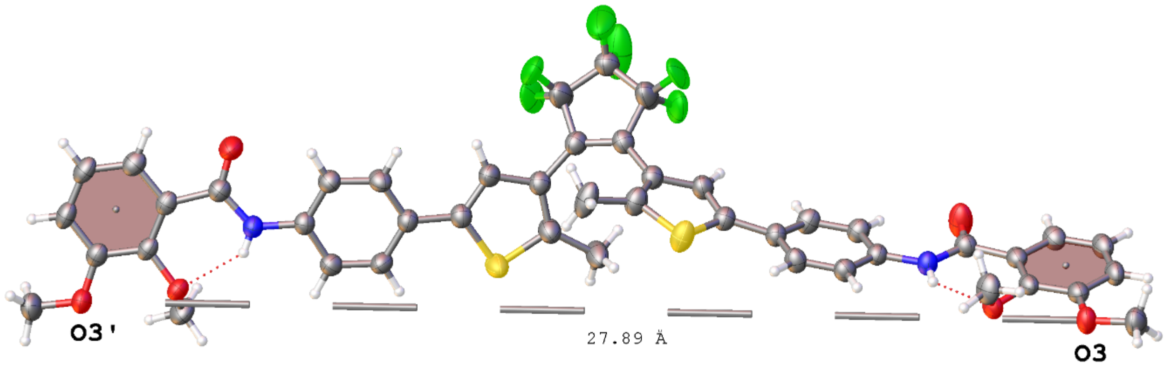

Figure S56. Interatomic O···O distances within **2** illustrating the O3'···O3 of 27.89 Å distance.

The atoms labelled with (') have been generated with the symmetry operator ½–x, y, 1–z.

Table S3. Bond lengths [Å] and angles [°] for **2**.

S(2)-C(14)	1.7166(18)	C(4)-C(5)	1.502(2)
S(2)-C(13)	1.7384(17)	C(5)-C(11)	1.466(2)
S(1)-C(9)	1.7180(18)	C(11)-C(14)	1.377(2)
S(1)-C(8)	1.7364(18)	C(11)-C(12)	1.425(2)
F(1)-C(2)	1.347(2)	C(14)-C(15)	1.500(3)
F(2)-C(2)	1.362(2)	C(15)-H(15A)	0.9800
F(3)-C(3)	1.342(3)	C(15)-H(15B)	0.9800
F(4)-C(3)	1.342(2)	C(15)-H(15C)	0.9800
F(5)-C(4)	1.349(2)	C(12)-H(12)	0.9500
F(6)-C(4)	1.367(2)	C(12)-C(13)	1.367(2)
O(3)-C(29)	1.375(2)	C(13)-C(24)	1.472(2)
O(3)-C(30)	1.429(3)	C(24)-C(25)	1.406(2)
O(4)-C(28)	1.366(2)	C(24)-C(29)	1.394(2)
O(4)-C(31)	1.422(2)	C(25)-H(25)	0.9500
O(1)-C(21)	1.380(2)	C(25)-C(26)	1.380(3)
O(1)-C(22)	1.434(3)	C(26)-H(26)	0.9500
O(2)-C(20)	1.365(2)	C(26)-C(27)	1.395(3)
O(2)-C(23)	1.426(2)	C(27)-H(27)	0.9500
C(1)-C(2)	1.504(2)	C(27)-C(28)	1.386(3)
C(1)-C(5)	1.351(2)	C(28)-C(29)	1.402(2)
C(1)-C(6)	1.466(2)	C(31)-H(31A)	0.9800
C(2)-C(3)	1.540(3)	C(31)-H(31B)	0.9800
C(3)-C(4)	1.535(3)	C(31)-H(31C)	0.9800

C(30)-H(30A)	0.9800	C(23)-H(23B)	0.9800
C(30)-H(30B)	0.9800	C(23)-H(23C)	0.9800
C(30)-H(30C)	0.9800	S(3)-C(38)	1.7180(18)
C(6)-C(9)	1.376(2)	S(3)-C(41)	1.7251(17)
C(6)-C(7)	1.430(2)	S(4)-C(51)	1.7201(18)
C(9)-C(10)	1.500(2)	S(4)-C(54)	1.7331(17)
C(10)-H(10A)	0.9800	F(7)-C(33)	1.344(2)
C(10)-H(10B)	0.9800	F(8)-C(33)	1.371(2)
C(10)-H(10C)	0.9800	F(9)-C(34)	1.364(3)
C(7)-H(7)	0.9500	F(10)-C(34)	1.321(2)
C(7)-C(8)	1.364(2)	F(11)-C(35)	1.341(2)
C(8)-C(16)	1.472(2)	F(12)-C(35)	1.354(3)
C(16)-C(21)	1.395(2)	O(5)-C(43)	1.375(2)
C(16)-C(17)	1.404(2)	O(5)-C(48)	1.432(3)
C(21)-C(20)	1.405(2)	O(6)-C(44)	1.369(2
C(20)-C(19)	1.387(3)	O(6)-C(49)	1.431(3)
C(19)-H(19)	0.9500	O(7)-C(56)	1.380(2)
C(19)-C(18)	1.395(3)	O(7)-C(61)	1.437(2)
C(18)-H(18)	0.9500	O(8)-C(57)	1.363(2)
C(18)-C(17)	1.384(3)	O(8)-C(62)	1.428(2)
C(17)-H(17)	0.9500	C(32)-C(33)	1.501(2)
C(22)-H(22A)	0.9800	C(32)-C(36)	1.352(2)
C(22)-H(22B)	0.9800	C(32)-C(50)	1.467(2)
C(22)-H(22C)	0.9800	C(33)-C(34)	1.528(3)
C(23)-H(23A)	0.9800	C(34)-C(35)	1.535(3)

C(35)-C(36)	1.502(2)	C(49)-H(49C)	0.9800
C(36)-C(37)	1.462(2)	C(50)-C(51)	1.373(2)
C(37)-C(38)	1.381(2)	C(50)-C(53)	1.433(2)
C(37)-C(40)	1.431(2)	C(51)-C(52)	1.498(2)
C(38)-C(39)	1.496(2)	C(52)-H(52A)	0.9800
C(39)-H(39A)	0.9800	C(52)-H(52B)	0.9800
C(39)-H(39B)	0.9800	C(52)-H(52C)	0.9800
C(39)-H(39C)	0.9800	C(53)-H(53)	0.9500
C(40)-H(40)	0.9500	C(53)-C(54)	1.365(2)
C(40)-C(41)	1.371(2)	C(54)-C(55)	1.475(2)
C(41)-C(42)	1.476(2)	C(55)-C(56)	1.393(2)
C(42)-C(43)	1.394(2)	C(55)-C(60)	1.407(2)
C(42)-C(47)	1.404(3)	C(56)-C(57)	1.406(2)
C(43)-C(44)	1.408(3)	C(57)-C(58)	1.390(3)
C(44)-C(45)	1.383(3)	C(58)-H(58)	0.9500
C(45)-H(45)	0.9500	C(58)-C(59)	1.389(3)
C(45)-C(46)	1.389(3)	C(59)-H(59)	0.9500
C(46)-H(46)	0.9500	C(59)-C(60)	1.382(3)
C(46)-C(47)	1.384(3)	C(60)-H(60)	0.9500
C(47)-H(47)	0.9500	C(61)-H(61A)	0.9800
C(48)-H(48A)	0.9800	C(61)-H(61B)	0.9800
C(48)-H(48B)	0.9800	C(61)-H(61C)	0.9800
C(48)-H(48C)	0.9800	C(62)-H(62A)	0.9800
C(49)-H(49A)	0.9800	C(62)-H(62B)	0.9800
C(49)-H(49B)	0.9800	C(62)-H(62C)	0.9800

C(14)-S(2)-C(13)	93.31(8)	F(6)-C(4)-C(5)	111.37(16)
C(9)-S(1)-C(8)	93.11(8)	C(5)-C(4)-C(3)	104.89(15)
C(29)-O(3)-C(30)	114.38(16)	C(1)-C(5)-C(4)	110.77(15)
C(28)-O(4)-C(31)	117.26(16)	C(1)-C(5)-C(11)	129.20(16)
C(21)-O(1)-C(22)	110.90(14)	C(11)-C(5)-C(4)	120.02(15)
C(20)-O(2)-C(23)	117.54(16)	C(14)-C(11)-C(5)	125.39(16)
C(5)-C(1)-C(2)	110.70(15)	C(14)-C(11)-C(12)	112.72(16)
C(5)-C(1)-C(6)	128.42(16)	C(12)-C(11)-C(5)	121.88(15)
C(6)-C(1)-C(2)	120.74(15)	C(11)-C(14)-S(2)	110.48(13)
F(1)-C(2)-F(2)	106.35(16)	C(11)-C(14)-C(15)	130.17(17)
F(1)-C(2)-C(1)	114.27(15)	C(15)-C(14)-S(2)	119.34(13)
F(1)-C(2)-C(3)	111.67(16)	C(14)-C(15)-H(15A)	109.5
F(2)-C(2)-C(1)	110.68(16)	C(14)-C(15)-H(15B)	109.5
F(2)-C(2)-C(3)	109.19(15)	C(14)-C(15)-H(15C)	109.5
C(1)-C(2)-C(3)	104.68(15)	H(15A)-C(15)-H(15B)	109.5
F(3)-C(3)-C(2)	109.49(16)	H(15A)-C(15)-H(15C)	109.5
F(3)-C(3)-C(4)	109.47(17)	H(15B)-C(15)-H(15C)	109.5
F(4)-C(3)-F(3)	108.16(16)	C(11)-C(12)-H(12)	123.0
F(4)-C(3)-C(2)	113.02(17)	C(13)-C(12)-C(11)	113.97(15)
F(4)-C(3)-C(4)	113.10(16)	C(13)-C(12)-H(12)	123.0
C(4)-C(3)-C(2)	103.50(15)	C(12)-C(13)-S(2)	109.52(13)
F(5)-C(4)-F(6)	106.06(15)	C(12)-C(13)-C(24)	126.49(16)
F(5)-C(4)-C(3)	111.87(17)	C(24)-C(13)-S(2)	123.94(13)
F(5)-C(4)-C(5)	113.22(15)	C(25)-C(24)-C(13)	119.87(16)
F(6)-C(4)-C(3)	109.51(15)	C(29)-C(24)-C(13)	121.93(16)

C(29)-C(24)-C(25)	118.16(16)	H(30A)-C(30)-H(30B)	109.5
C(24)-C(25)-H(25)	119.8	H(30A)-C(30)-H(30C)	109.5
C(26)-C(25)-C(24)	120.38(17)	H(30B)-C(30)-H(30C)	109.5
C(26)-C(25)-H(25)	119.8	C(9)-C(6)-C(1)	124.15(16)
C(25)-C(26)-H(26)	119.4	C(9)-C(6)-C(7)	113.04(15)
C(25)-C(26)-C(27)	121.15(18)	C(7)-C(6)-C(1)	122.81(16)
C(27)-C(26)-H(26)	119.4	C(6)-C(9)-S(1)	110.38(13)
C(26)-C(27)-H(27)	120.4	C(6)-C(9)-C(10)	129.15(16)
C(28)-C(27)-C(26)	119.27(18)	C(10)-C(9)-S(1)	120.47(13)
C(28)-C(27)-H(27)	120.4	C(9)-C(10)-H(10A)	109.5
O(4)-C(28)-C(27)	125.23(17)	C(9)-C(10)-H(10B)	109.5
O(4)-C(28)-C(29)	115.08(16)	C(9)-C(10)-H(10C)	109.5
C(27)-C(28)-C(29)	119.67(17)	H(10A)-C(10)-H(10B)	109.5
O(3)-C(29)-C(24)	119.24(16)	H(10A)-C(10)-H(10C)	109.5
O(3)-C(29)-C(28)	119.43(16)	H(10B)-C(10)-H(10C)	109.5
C(24)-C(29)-C(28)	121.30(16)	C(6)-C(7)-H(7)	123.4
O(4)-C(31)-H(31A)	109.5	C(8)-C(7)-C(6)	113.27(15)
O(4)-C(31)-H(31B)	109.5	C(8)-C(7)-H(7)	123.4
O(4)-C(31)-H(31C)	109.5	C(7)-C(8)-S(1)	110.17(13)
H(31A)-C(31)-H(31B)	109.5	C(7)-C(8)-C(16)	126.36(16)
H(31A)-C(31)-H(31C)	109.5	C(16)-C(8)-S(1)	123.26(12)
H(31B)-C(31)-H(31C)	109.5	C(21)-C(16)-C(8)	121.87(16)
O(3)-C(30)-H(30A)	109.5	C(21)-C(16)-C(17)	118.28(16)
O(3)-C(30)-H(30B)	109.5	C(17)-C(16)-C(8)	119.85(15)
O(3)-C(30)-H(30C)	109.5	O(1)-C(21)-C(16)	120.30(15)

O(1)-C(21)-C(20)	118.49(15)	H(23B)-C(23)-H(23C)	109.5
C(16)-C(21)-C(20)	121.21(16)	C(38)-S(3)-C(41)	93.46(8)
O(2)-C(20)-C(21)	114.63(16)	C(51)-S(4)-C(54)	93.29(8)
O(2)-C(20)-C(19)	125.70(16)	C(43)-O(5)-C(48)	113.79(16)
C(19)-C(20)-C(21)	19.67(16)	C(44)-O(6)-C(49)	116.72(17)
C(20)-C(19)-H(19)	120.4	C(56)-O(7)-C(61)	112.29(14)
C(20)-C(19)-C(18)	119.29(17)	C(57)-O(8)-C(62)	117.31(15)
C(18)-C(19)-H(19)	120.4	C(36)-C(32)-C(33)	110.94(15)
C(19)-C(18)-H(18)	119.4	C(36)-C(32)-C(50)	127.73(16)
C(17)-C(18)-C(19)	121.12(17)	C(50)-C(32)-C(33)	121.23(15)
C(17)-C(18)-H(18)	119.4	F(7)-C(33)-F(8)	105.31(16)
C(16)-C(17)-H(17)	119.8	F(7)-C(33)-C(32)	114.12(16)
C(18)-C(17)-C(16)	120.39(17)	F(7)-C(33)-C(34)	113.04(17)
C(18)-C(17)-H(17)	119.8	F(8)-C(33)-C(32)	110.77(16)
O(1)-C(22)-H(22A)	109.5	F(8)-C(33)-C(34)	108.02(17)
O(1)-C(22)-H(22B)	109.5	C(32)-C(33)-C(34)	105.52(15)
O(1)-C(22)-H(22C)	109.5	F(9)-C(34)-C(33)	107.65(18)
H(22A)-C(22)-H(22B)	109.5	F(9)-C(34)-C(35)	108.46(17)
H(22A)-C(22)-H(22C)	109.5	F(10)-C(34)-F(9)	107.18(18)
H(22B)-C(22)-H(22C)	109.5	F(10)-C(34)-C(33)	114.85(17)
O(2)-C(23)-H(23A)	109.5	F(10)-C(34)-C(35)	114.25(18)
O(2)-C(23)-H(23B)	109.5	C(33)-C(34)-C(35)	104.16(15)
O(2)-C(23)-H(23C)	109.5	F(11)-C(35)-F(12)	106.72(17)
H(23A)-C(23)-H(23B)	109.5	F(11)-C(35)-C(34)	111.19(18)
H(23A)-C(23)-H(23C)	109.5	F(11)-C(35)-C(36)	113.18(16)

F(12)-C(35)-C(34)	108.92(17)	C(43)-C(42)-C(47)	118.71(17)
F(12)-C(35)-C(36)	111.50(18)	C(47)-C(42)-C(41)	119.61(16)
C(36)-C(35)-C(34)	105.33(15)	O(5)-C(43)-C(42)	120.14(16)
C(32)-C(36)-C(35)	110.57(15)	O(5)-C(43)-C(44)	119.23(16)
C(32)-C(36)-C(37)	129.35(16)	C(42)-C(43)-C(44)	120.58(17)
C(37)-C(36)-C(35)	120.08(15)	O(6)-C(44)-C(43)	114.85(17)
C(38)-C(37)-C(36)	124.50(16)	O(6)-C(44)-C(45)	125.33(18)
C(38)-C(37)-C(40)	112.33(15)	C(45)-C(44)-C(43)	119.81(18)
C(40)-C(37)-C(36)	123.12(15)	C(44)-C(45)-H(45)	120.2
C(37)-C(38)-S(3)	110.55(13)	C(44)-C(45)-C(46)	119.68(18)
C(37)-C(38)-C(39)	129.66(17)	C(46)-C(45)-H(45)	120.2
C(39)-C(38)-S(3)	119.68(13)	C(45)-C(46)-H(46)	119.5
C(38)-C(39)-H(39A)	109.5	C(47)-C(46)-C(45)	120.90(19)
C(38)-C(39)-H(39B)	109.5	C(47)-C(46)-H(46)	119.5
C(38)-C(39)-H(39C)	109.5	C(42)-C(47)-H(47)	119.9
H(39A)-C(39)-H(39B)	109.5	C(46)-C(47)-C(42)	120.25(18)
H(39A)-C(39)-H(39C)	109.5	C(46)-C(47)-H(47)	119.9
H(39B)-C(39)-H(39C)	109.5	O(5)-C(48)-H(48A)	109.5
C(37)-C(40)-H(40)	123.1	O(5)-C(48)-H(48B)	109.5
C(41)-C(40)-C(37)	113.71(15)	O(5)-C(48)-H(48C)	109.5
C(41)-C(40)-H(40)	123.1	H(48A)-C(48)-H(48B)	109.5
C(40)-C(41)-S(3)	109.94(13)	H(48A)-C(48)-H(48C)	109.5
C(40)-C(41)-C(42)	127.16(16)	H(48B)-C(48)-H(48C)	109.5
C(42)-C(41)-S(3)	122.89(13)	O(6)-C(49)-H(49A)	109.5
C(43)-C(42)-C(41)	121.65(16)	O(6)-C(49)-H(49B)	109.5

O(6)-C(49)-H(49C)	109.5	O(7)-C(56)-C(55)	121.05(15)
H(49A)-C(49)-H(49B)	109.5	O(7)-C(56)-C(57)	118.56(15)
H(49A)-C(49)-H(49C)	109.5	C(55)-C(56)-C(57)	120.37(16)
H(49B)-C(49)-H(49C)	109.5	O(8)-C(57)-C(56)	114.67(15)
C(51)-C(50)-C(32)	122.81(15)	O(8)-C(57)-C(58)	124.93(16)
C(51)-C(50)-C(53)	113.20(15)	C(58)-C(57)-C(56)	120.40(16)
C(53)-C(50)-C(32)	123.85(15)	C(57)-C(58)-H(58)	120.5
C(50)-C(51)-S(4)	110.19(13)	C(59)-C(58)-C(57)	119.04(16)
C(50)-C(51)-C(52)	128.96(16)	C(59)-C(58)-H(58)	120.5
C(52)-C(51)-S(4)	120.85(13)	C(58)-C(59)-H(59)	119.5
C(51)-C(52)-H(52A)	109.5	C(60)-C(59)-C(58)	121.08(17)
C(51)-C(52)-H(52B)	109.5	C(60)-C(59)-H(59)	119.5
C(51)-C(52)-H(52C)	109.5	C(55)-C(60)-H(60)	119.7
H(52A)-C(52)-H(52B)	109.5	C(59)-C(60)-C(55)	120.54(17)
H(52A)-C(52)-H(52C)	109.5	C(59)-C(60)-H(60)	119.7
H(52B)-C(52)-H(52C)	109.5	O(7)-C(61)-H(61A)	109.5
C(50)-C(53)-H(53)	123.4	O(7)-C(61)-H(61B)	109.5
C(54)-C(53)-C(50)	113.10(15)	O(7)-C(61)-H(61C)	109.5
C(54)-C(53)-H(53)	123.4	H(61A)-C(61)-H(61B)	109.5
C(53)-C(54)-S(4)	110.15(13)	H(61A)-C(61)-H(61C)	109.5
C(53)-C(54)-C(55)	130.70(16)	H(61B)-C(61)-H(61C)	109.5
C(55)-C(54)-S(4)	119.03(13)	O(8)-C(62)-H(62A)	109.5
C(56)-C(55)-C(54)	121.46(15)	O(8)-C(62)-H(62B)	109.5
C(56)-C(55)-C(60)	118.57(16)	O(8)-C(62)-H(62C)	109.5
C(60)-C(55)-C(54)	119.96(16)	H(62A)-C(62)-H(62B)	109.5

H(62A)-C(62)-H(62C)	109.5	H(62B)-C(62)-H(62C)	109.5

Table S4. Bond lengths [Å] and angles [°] for **6**.

S(1)-C(8)	1.737(4)	C(21)-H(21)	0.9400
S(1)-C(9)	1.727(4)	C(21)-C(22)	1.372(6)
O(2)-C(19)	1.388(4)	C(21)-C(20)	1.384(5)
O(2)-C(24)	1.437(5)	C(15)-H(15)	0.9400
O(3)-C(20)	1.362(5)	C(15)-C(16)	1.375(5)
O(3)-C(25)	1.431(5)	F(3)-C(3)	1.313(5)
N(1)-C(14)	1.411(5)	C(1)-C(1)#1	1.345(8)
N(1)-C(17)	1.352(5)	C(1)-C(6)	1.474(6)
N(1)-H(1)	0.75(5)	C(1)-C(2)	1.490(6)
O(1)-C(17)	1.220(5)	C(12)-H(12)	0.9400
C(14)-C(13)	1.395(5)	C(22)-H(22)	0.9400
C(14)-C(15)	1.392(5)	C(22)-C(23)	1.374(6)
F(2)-C(2)	1.348(6)	C(16)-H(16)	.9400
C(19)-C(18)	1.386(6)	C(6)-C(7)	1.428(5)
C(19)-C(20)	1.406(5)	C(6)-C(9)	1.368(6)
F(1)-C(2)	1.404(6)	C(23)-H(23)	0.9400
C(13)-H(13)	0.9400	C(8)-C(7)	1.354(6)
C(13)-C(12)	1.387(5)	C(7)-H(7)	0.9400
C(18)-C(23)	1.399(5)	C(3)-C(2)	1.512(6)
C(18)-C(17)	1.509(5)	C(3)-C(2)#1	1.512(6)
C(11)-C(12)	1.393(5)	C(24)-H(24A)	0.9700
C(11)-C(16)	1.399(5)	C(24)-H(24B)	0.9700
C(11)-C(8)	1.472(5)	C(24)-H(24C)	0.9700

C(9)-C(10)	1.509(6)	C(19)-C(18)-C(17)	126.8(3)
C(2)-F(1A)	1.364(7)	C(23)-C(18)-C(17)	114.3(4)
C(2)-F(2A)	1.397(7)	C(12)-C(11)-C(16)	116.7(3)
C(25)-H(25A)	0.9700	C(12)-C(11)-C(8)	120.1(4)
C(25)-H(25B)	0.9700	C(16)-C(11)-C(8)	123.0(3)
C(25)-H(25C)	0.9700	C(22)-C(21)-H(21)	119.9
C(10)-H(10A)	0.9700	C(22)-C(21)-C(20)	120.1(4)
C(10)-H(10B)	0.9700	C(20)-C(21)-H(21)	119.9
C(10)-H(10C)	0.9700	C(14)-C(15)-H(15)	119.8
C(9)-S(1)-C(8)	93.0(2)	C(16)-C(15)-C(14)	120.4(4)
C(19)-O(2)-C(24)	115.9(3)	C(16)-C(15)-H(15)	119.8
C(20)-O(3)-C(25)	116.9(3)	C(1)#1-C(1)-C(6)	130.7(2)
C(14)-N(1)-H(1)	114(4)	C(1)#1-C(1)-C(2)	110.6(2)
C(17)-N(1)-C(14)	128.2(4)	C(6)-C(1)-C(2)	118.7(3)
C(17)-N(1)-H(1)	117(4)	C(13)-C(12)-C(11)	122.3(4)
C(13)-C(14)-N(1)	123.5(3)	C(13)-C(12)-H(12)	118.8
C(15)-C(14)-N(1)	117.4(3)	C(11)-C(12)-H(12)	118.8
C(15)-C(14)-C(13)	119.1(3)	C(21)-C(22)-H(22)	119.6
O(2)-C(19)-C(20)	118.7(3)	C(21)-C(22)-C(23)	120.7(4)
C(18)-C(19)-O(2)	121.1(3)	C(23)-C(22)-H(22)	119.6
C(18)-C(19)-C(20)	120.1(3)	O(3)-C(20)-C(19)	116.2(3)
C(14)-C(13)-H(13)	120.3	O(3)-C(20)-C(21)	124.2(4)
C(12)-C(13)-C(14)	119.5(4)	C(21)-C(20)-C(19)	119.6(4)
C(12)-C(13)-H(13)	120.3	C(11)-C(16)-H(16)	119.1
C(19)-C(18)-C(23)	118.9(3)	C(15)-C(16)-C(11)	121.9(4)

C(15)-C(16)-H(16)	119.1	H(24A)-C(24)-H(24B)	109.5
C(7)-C(6)-C(1)	122.9(4)	H(24A)-C(24)-H(24C)	109.5
C(9)-C(6)-C(1)	124.6(4)	H(24B)-C(24)-H(24C)	109.5
C(9)-C(6)-C(7)	112.4(4)	C(6)-C(9)-S(1)	110.5(3)
C(18)-C(23)-H(23)	119.7	C(6)-C(9)-C(10)	130.0(4)
C(22)-C(23)-C(18)	120.5(4)	C(10)-C(9)-S(1)	119.4(3)
C(22)-C(23)-H(23)	119.7	F(2)-C(2)-F(1)	103.4(6)
N(1)-C(17)-C(18)	117.5(3)	F(2)-C(2)-C(1)	117.0(5)
O(1)-C(17)-N(1)	122.7(4)	F(2)-C(2)-C(3)	117.2(6)
O(1)-C(17)-C(18)	119.8(4)	F(1)-C(2)-C(1)	107.7(6)
C(11)-C(8)-S(1)	121.5(3)	F(1)-C(2)-C(3)	103.8(6)
C(7)-C(8)-S(1)	109.5(3)	C(1)-C(2)-C(3)	106.5(4)
C(7)-C(8)-C(11)	128.9(4)	F(1A)-C(2)-C(1)	119.3(8)
C(6)-C(7)-H(7)	122.7	F(1A)-C(2)-C(3)	116.5(8)
C(8)-C(7)-C(6)	114.6(4)	F(1A)-C(2)-F(2A)	108.4(8)
C(8)-C(7)-H(7)	122.7	F(2A)-C(2)-C(1)	104.5(7)
F(3)#1-C(3)-F(3)	105.5(7)	F(2A)-C(2)-C(3)	99.1(7)
F(3)#1-C(3)-C(2)	110.9(3)	O(3)-C(25)-H(25A)	109.5
F(3)#1-C(3)-C(2)#1	112.3(3)	O(3)-C(25)-H(25B)	109.5
F(3)-C(3)-C(2)#1	110.9(3)	O(3)-C(25)-H(25C)	109.5
F(3)-C(3)-C(2)	112.3(3)	H(25A)-C(25)-H(25B)	109.5
C(2)#1-C(3)-C(2)	105.2(5)	H(25A)-C(25)-H(25C)	109.5
O(2)-C(24)-H(24A)	109.5	H(25B)-C(25)-H(25C)	109.5
O(2)-C(24)-H(24B)	109.5	C(9)-C(10)-H(10A)	109.5
O(2)-C(24)-H(24C)	109.5	C(9)-C(10)-H(10B)	109.5

C(9)-C(10)-H(10C)	109.5
H(10A)-C(10)-H(10B)	109.5
H(10A)-C(10)-H(10C)	109.5
H(10B)-C(10)-H(10C)	109.5

Symmetry transformations used to generate equivalent atoms:

#1: – x +1/2, y, –z+1

5. Details of hydrodynamic diameter study

A) ΔΔ-helicate-[$Ga_2\mathbf{7}_3$]$^{6-}$ (*open*) **B)** ΔΔ-helicate-[$Ga_2\mathbf{7}_3$]$^{6-}$ (*closed*) **C)** ΔΛ-mesocate-[$Ga_2\mathbf{7}_3$]$^{6-}$ (*open*) **D)** ΔΛ-mesocate-[$Ga_2\mathbf{7}_3$]$^{6-}$ (*closed*)

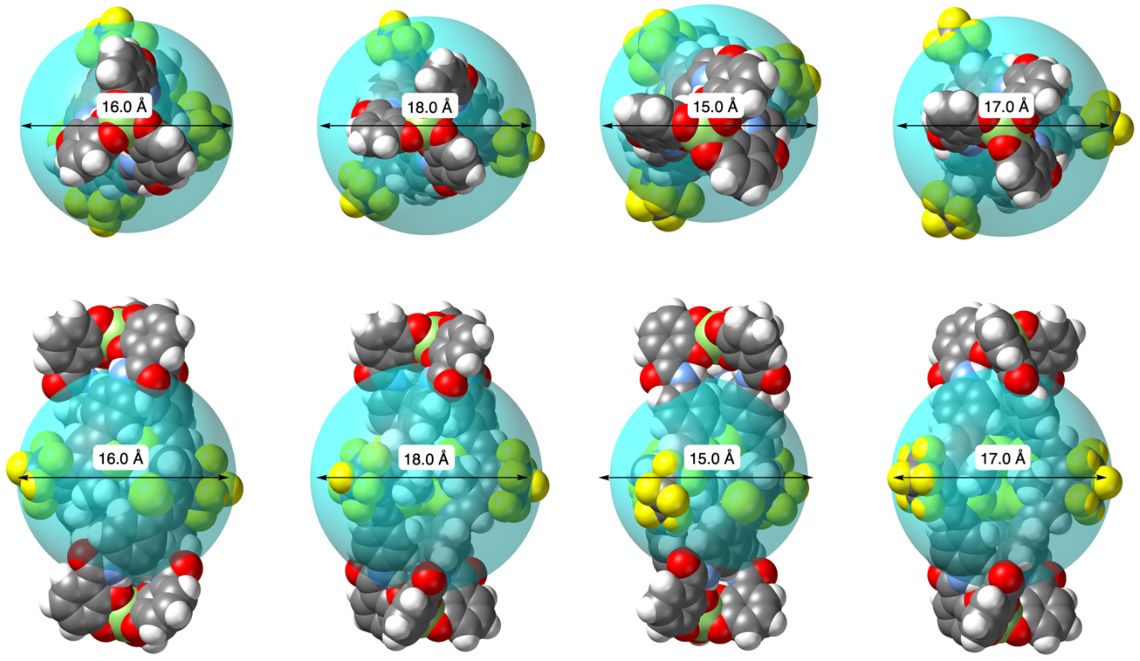

Figure S57. Estimated molecular diameter (D_{cage}, Å) for the cages: **A)** ΔΔ-helicate-[$Ga_2\mathbf{7}_3$]$^{6-}$ (*open*) (16 Å), **B)** ΔΔ-helicate-[$Ga_2\mathbf{7}_3$]$^{6-}$ (*closed*) (18 Å), **C)** ΔΛ-mesocate-[$Ga_2\mathbf{7}_3$]$^{6-}$ (*open*) (15 Å), **D)** ΔΛ-mesocate-[$Ga_2\mathbf{7}_3$]$^{6-}$ (*closed*) (17 Å), obtained from the electronic computed structures in DMF (see Section 8). These estimations were calculated using a geometrically centred spherical probes of the given diameter (Å) within space-filling models, utilizing van der Waals radii for all atoms as reported by S. Álvarez.[10] The spherical probe was fitted to the smallest dimension, representing the hydrodynamic diameter typically observed in DOSY NMR experiments for rod-like molecules, where the diffusion coefficient often corresponds to the smaller dimension of the structure.[1, 12] This method provides a practical approximation of the hydrodynamic diameter in solution, correlating well with the experimentally observed diffusion behaviour.

6. Density-Functional-Theory (DFT) Calculations

Table S5. Calculated relative energies for conformers of isomeric form of the cage $[Ga_2\mathbf{7}_3]^{6-}$ (ΔΔ, ΛΛ, and ΔΛ) in the gas phase. Energies are given in kcal mol^{-1}; open: all DTE in their open form, closed: all DTE in their closed form.

Complex	Conformer	Δ*E*	Δ*G*
ΔΔ-open	conformer_1	**0.0**	**0.0**
	conformer_2	+0.4	+0.3
	conformer_3	+1.9	+0.9
	conformer_4	+2.5	+1.3
	conformer_5	+24.4	+20.3
ΔΔ-closed	conformer_1	+41.9	+43.5
ΛΛ-open	conformer_1	0.0	0.0
ΛΛ-closed	conformer_1	+41.9	+43.5
ΔΛ-open	conformer_1	+3.0	+0.7
	conformer_2	+4.1	+2.8
	conformer_3	+4.8	+4.3
	conformer_4	+4.8	+4.5
	conformer_5	+6.6	+5.5
ΔΛ-closed	conformer_1	+54.6	+54.2

Table S6. Calculated relative energies of diastereomeric form of the cage $[Ga_2\mathbf{7}_3]^{6-}$ (ΔΔ and ΔΛ) in DMF implicit solvent. Energies are given in kcal mol^{-1}; open: all DTE in their open form, closed: all DTE in their closed form.

**Complex**	**Conformer**	**ΔE**	**ΔG**
ΔΔ-open	conformer_1	0.0	0.0
ΔΔ-closed	conformer_1	+60.3	+59.1
ΔΛ-open	conformer_1	+6.6	+4.8
ΔΛ-closed	conformer_1	+72.7	+70.5

Cartesian coordinates

ΔΔ_open_conformer_1_gas_phase

Ga	-11.691674	0.727186	0.447492
Ga	11.966156	0.754366	0.354424
C	-10.396399	5.561293	-0.849826
C	-9.170165	5.209645	-0.321795
C	-8.989128	3.943733	0.301145
C	-10.087856	3.048880	0.385972
C	-11.362176	3.406272	-0.210720
C	-11.489789	4.662957	-0.800595
C	-6.415870	1.759579	1.966891
C	-6.563938	0.416930	2.383205
C	-5.514039	-0.273048	2.966271
C	-4.265911	0.347407	3.175445
C	-4.128406	1.689065	2.774566
C	-5.169663	2.390559	2.175778
C	-3.135543	-0.413182	3.711314
C	-2.790661	-1.715122	3.455135
C	-1.534204	-2.114053	4.031614
C	-0.947121	-1.126483	4.795613
S	-1.938771	0.313345	4.788722
C	-0.904629	-3.407043	3.737639
C	-1.666815	-4.663869	4.057166
C	-0.838559	-5.812909	3.444751
C	0.561670	-5.186418	3.256464
C	0.341897	-3.690910	3.254023
C	1.435846	-2.847867	2.777216
C	2.808629	-3.292883	2.836007
C	3.713126	-2.411513	2.301447
S	2.872291	-0.994090	1.702725
C	1.316358	-1.602767	2.168940
F	-2.943422	-4.676297	3.575257
F	-1.767848	-4.852678	5.414412
F	-1.345395	-6.172815	2.241142
F	-0.809845	-6.917256	4.235205
F	1.371407	-5.585175	4.295606
F	1.144469	-5.653204	2.114803
C	0.386994	-1.142650	5.476382
C	0.103854	-0.808385	1.805715
C	5.154073	-2.545977	2.083124
C	5.721335	-3.801752	1.797570
C	7.067618	-3.946682	1.479800

C	7.903297	-2.810396	1.424048
C	7.347916	-1.549582	1.747590
C	6.005583	-1.421799	2.062209
O	-10.043764	1.869823	0.967539
O	-12.331540	2.511065	-0.154787
O	-10.657089	-0.822501	1.320875
O	-13.032649	-0.356475	-0.549118
O	-12.652193	0.649790	2.193956
O	-10.563401	0.264453	-1.233276
C	-12.080177	-1.488669	5.134110
C	-10.988455	-2.220222	4.708309
C	-10.445089	-2.013880	3.410399
C	-11.052533	-1.062549	2.551012
C	-12.172619	-0.264802	3.015921
C	-12.668997	-0.509674	4.296583
C	-7.474551	-2.745836	1.237965
C	-7.152360	-2.093574	0.024360
C	-5.905726	-2.239599	-0.557108
C	-4.918883	-3.061923	0.028774
C	-5.256892	-3.731783	1.219737
C	-6.504189	-3.586264	1.821982
C	-3.596869	-3.175867	-0.588512
C	-3.211947	-2.880485	-1.872637
C	-1.800365	-3.018375	-2.113913
C	-1.099337	-3.457966	-1.004550
S	-2.186870	-3.691712	0.335615
C	-1.210724	-2.709573	-3.412666
C	-1.975278	-3.067432	-4.659206
C	-0.932823	-2.993962	-5.795352
C	0.169709	-2.073140	-5.223681
C	-0.051135	-2.063009	-3.732428
C	0.840421	-1.253437	-2.907316
C	2.261682	-1.142934	-3.136912
C	2.884354	-0.203122	-2.361961
S	1.703445	0.591628	-1.343622
C	0.389653	-0.370335	-1.933262
F	-2.979735	-2.164642	-4.918002
F	-2.561448	-4.295393	-4.613734
F	-1.446309	-2.533422	-6.960936
F	-0.425350	-4.236882	-6.021138
F	1.404388	-2.541488	-5.594466
F	0.060478	-0.821275	-5.769832
C	0.368663	-3.702234	-0.829835

C	-0.995960	-0.136651	-1.416919
C	4.292645	0.255890	-2.370905
C	5.309855	-0.367246	-1.627471
C	6.615753	0.114709	-1.655102
C	6.961084	1.251796	-2.420790
C	5.938759	1.878571	-3.169338
C	4.638907	1.384964	-3.129766
C	6.853513	2.159601	2.376118
C	5.761731	2.255095	3.269559
C	4.483136	2.507680	2.786530
C	4.217752	2.677275	1.414450
C	5.306981	2.551302	0.526981
C	6.589569	2.305314	0.994330
C	2.878543	3.005026	0.920275
C	2.518149	3.700787	-0.207799
C	1.097703	3.819675	-0.402801
C	0.357890	3.220153	0.599853
S	1.423289	2.502016	1.780032
C	0.534278	4.655358	-1.464831
C	1.162360	6.015393	-1.684729
C	0.116108	6.803103	-2.500342
C	-0.686527	5.689541	-3.201056
C	-0.470699	4.458812	-2.353281
C	-1.235776	3.261971	-2.760379
C	-2.614377	3.024352	-2.440958
C	-3.181792	1.976680	-3.133177
S	-1.989161	1.308048	-4.252376
C	-0.741527	2.405956	-3.722764
F	2.316149	5.911216	-2.421095
F	1.481327	6.667331	-0.536228
F	0.673112	7.686769	-3.365742
F	-0.692293	7.491615	-1.652759
F	-1.998984	6.029725	-3.324624
F	-0.202006	5.513824	-4.472561
C	-1.126297	3.206069	0.815552
C	0.624133	2.366317	-4.341557
C	-4.526226	1.406124	-3.023538
C	-4.956671	0.364554	-3.869192
C	-6.243149	-0.159373	-3.814299
C	-7.174740	0.348820	-2.882960
C	-6.740136	1.365174	-1.999951
C	-5.455651	1.880268	-2.069073
C	-10.598261	-1.199010	-3.156080

C	-11.375557	-2.091012	-3.946142
C	-12.682265	-2.374680	-3.602270
C	-13.268662	-1.793374	-2.452120
C	-12.548690	-0.916116	-1.641135
C	-11.182403	-0.592571	-2.014453
N	-8.716699	-2.466982	1.783922
N	-7.522433	2.365234	1.394994
N	-8.482902	-0.089272	-2.760262
C	-9.195318	-0.966555	-3.575148
C	-7.634575	3.621441	0.811207
C	-9.214084	-2.761988	3.049855
O	-6.667378	4.394833	0.710735
O	-8.651782	-3.559845	3.816117
O	-8.688081	-1.506849	-4.570367
H	-8.408469	1.829405	1.374191
H	-9.317384	-1.800833	1.265674
H	-9.058860	0.309488	-1.997939
C	12.001616	0.888365	5.557583
C	10.682775	1.266668	5.410223
C	10.117244	1.429308	4.113703
C	10.926651	1.214840	2.967768
C	12.306109	0.785972	3.131096
C	12.811971	0.643792	4.423388
O	10.517335	1.371186	1.727405
O	13.006763	0.555782	2.040446
O	10.573293	1.385158	-1.060188
O	13.221618	-0.358532	-0.751186
O	12.821546	2.455838	-0.175249
O	10.991787	-1.046694	0.431094
C	11.893819	4.947505	-2.705529
C	10.681876	4.439664	-3.125712
C	10.167568	3.233378	-2.569642
C	10.926565	2.532612	-1.597853
C	12.181353	3.098878	-1.130521
C	12.640728	4.283720	-1.704091
C	11.274045	-3.389038	-0.100201
C	12.107803	-4.365253	-0.712679
C	13.292455	-3.992745	-1.317985
C	13.698276	-2.636320	-1.345061
C	12.917050	-1.638804	-0.758357
C	11.677795	-2.029602	-0.113246
N	8.271132	1.719382	-2.360701
N	8.160836	1.906114	2.761098

N	9.231354	-2.822850	1.042785
C	9.986078	-3.844452	0.479947
C	8.682587	1.788807	4.049598
C	8.814050	2.828655	-3.011639
O	7.985305	1.965949	5.060960
O	8.184060	3.454046	-3.879273
O	9.602013	-5.025180	0.452921
H	8.875256	1.778614	2.023324
H	8.935495	1.258446	-1.719483
H	9.706223	-1.903441	0.990276
H	-10.525501	6.534533	-1.326462
H	-8.311726	5.872549	-0.371861
H	-12.451673	4.926573	-1.238772
H	-7.519197	-0.079351	2.237644
H	-5.661069	-1.307519	3.260580
H	-3.167995	2.184500	2.901760
H	-5.044532	3.413134	1.848687
H	-3.371792	-2.340358	2.790542
H	3.107432	-4.235891	3.274951
H	0.703320	-2.173936	5.660329
H	1.160205	-0.676544	4.850514
H	0.362231	-0.610983	6.434942
H	-0.196370	-0.125268	2.605270
H	0.305741	-0.200198	0.926263
H	-0.743284	-1.462325	1.594421
H	5.076532	-4.678220	1.781703
H	7.490758	-4.910345	1.230900
H	7.987729	-0.670536	1.739746
H	5.615800	-0.434761	2.297411
H	-12.489947	-1.652446	6.132749
H	-10.503507	-2.953462	5.346201
H	-13.515873	0.083903	4.639291
H	-7.887186	-1.437990	-0.436375
H	-5.683108	-1.674745	-1.457036
H	-4.529744	-4.387001	1.694726
H	-6.743983	-4.091497	2.747454
H	-3.904599	-2.534191	-2.629838
H	2.794308	-1.737817	-3.867025
H	0.564859	-4.479609	-0.084606
H	0.824408	-3.998007	-1.780495
H	0.887542	-2.793261	-0.497741
H	-1.135383	0.907825	-1.130326
H	-1.221880	-0.770311	-0.555143

H	-1.739409	-0.371246	-2.175545
H	5.075482	-1.224876	-1.002474
H	7.376824	-0.374894	-1.052410
H	6.190077	2.746116	-3.761143
H	3.863826	1.887945	-3.704771
H	5.949240	2.139672	4.327159
H	3.668858	2.597944	3.503079
H	5.144105	2.617188	-0.544471
H	7.408001	2.210513	0.285751
H	3.243415	4.125688	-0.890807
H	-3.158665	3.611917	-1.711574
H	-1.570679	2.224002	0.616305
H	-1.613956	3.924430	0.151957
H	-1.380331	3.469882	1.848632
H	1.027412	1.348048	-4.345528
H	0.607974	2.727406	-5.378370
H	1.311679	3.000205	-3.774998
H	-4.269230	-0.060314	-4.596479
H	-6.555293	-0.948465	-4.483480
H	-7.432161	1.752776	-1.257834
H	-5.186141	2.662794	-1.365042
H	-10.899697	-2.537686	-4.814070
H	-13.270960	-3.061424	-4.213804
H	-14.294162	-2.025724	-2.166983
H	12.425687	0.763464	6.555908
H	10.035921	1.439369	6.264968
H	13.848811	0.327735	4.531365
H	12.276681	5.875229	-3.135271
H	10.075874	4.945738	-3.870677
H	13.587966	4.686678	-1.347802
H	11.771219	-5.397875	-0.693007
H	13.925449	-4.748498	-1.787858
H	14.628911	-2.339389	-1.827344

****

ΔΔ_open_conformer_2_gas_phase

Ga	-11.743087	0.537587	0.106199
Ga	11.957542	0.524508	0.037508
C	-10.820840	5.463823	-1.185301
C	-9.601693	5.231142	-0.580553
C	-9.341503	3.989454	0.063128
C	-10.356789	2.997835	0.092964

C	-11.617288	3.228105	-0.589938
C	-11.825402	4.465662	-1.197493
C	-6.642369	2.005646	1.776217
C	-6.679914	0.641540	2.145825
C	-5.579610	0.022141	2.713784
C	-4.383200	0.732387	2.949470
C	-4.356924	2.092310	2.587443
C	-5.451377	2.726759	2.009973
C	-3.211182	0.049446	3.498301
C	-2.866851	-1.275865	3.400012
C	-1.588796	-1.597429	3.976225
C	-0.966625	-0.515835	4.564276
S	-1.964745	0.911258	4.410232
C	-0.991741	-2.934747	3.886830
C	-1.770750	-4.092786	4.447782
C	-0.974317	-5.355207	4.054803
C	0.426735	-4.804970	3.701388
C	0.230920	-3.332024	3.423621
C	1.313492	-2.619325	2.751550
C	2.677527	-3.094616	2.809211
C	3.565528	-2.351310	2.076734
S	2.721800	-1.041945	1.277114
C	1.184473	-1.495932	1.939306
F	-3.053250	-4.169273	3.986268
F	-1.859441	-4.019368	5.816364
F	-1.525867	-5.943196	2.966109
F	-0.929982	-6.277197	5.051519
F	1.271950	-5.016154	4.766449
F	0.956583	-5.495452	2.650044
C	0.401248	-0.436474	5.168526
C	-0.031535	-0.741968	1.504091
C	4.998593	-2.533739	1.829530
C	5.531424	-3.810736	1.578920
C	6.866673	-3.994199	1.230677
C	7.723879	-2.878335	1.116865
C	7.202945	-1.596794	1.412084
C	5.870021	-1.428582	1.747252
O	-10.240534	1.834187	0.693901
O	-12.496065	2.242916	-0.591062
O	-10.649022	-0.890953	1.111996
O	-12.902085	-0.697038	-0.942440
O	-12.818617	0.438395	1.780140
O	-10.453752	0.120861	-1.465080

C	-12.335933	-1.589267	4.812269
C	-11.168357	-2.253796	4.490991
C	-10.537202	-2.036147	3.235346
C	-11.130697	-1.140464	2.309318
C	-12.340191	-0.419253	2.662161
C	-12.920086	-0.671215	3.905697
C	-7.404877	-2.733542	1.275008
C	-7.040789	-2.168416	0.030051
C	-5.777703	-2.360766	-0.502261
C	-4.813135	-3.143225	0.168819
C	-5.187443	-3.714741	1.400200
C	-6.450939	-3.521807	1.952862
C	-3.477240	-3.324113	-0.402397
C	-3.050935	-3.154025	-1.698282
C	-1.636069	-3.328129	-1.876011
C	-0.973111	-3.666644	-0.711684
S	-2.103522	-3.769656	0.608088
C	-0.947625	-3.235064	-3.163870
C	-1.402506	-4.112698	-4.297913
C	-0.526766	-3.696186	-5.499533
C	0.691209	-3.012851	-4.838932
C	0.189030	-2.558448	-3.490281
C	0.919133	-1.501832	-2.797564
C	2.346376	-1.342454	-2.918409
C	2.831050	-0.182831	-2.376831
S	1.504841	0.749680	-1.696966
C	0.315561	-0.441971	-2.135188
F	-2.728341	-3.975917	-4.592488
F	-1.199351	-5.442244	-4.027745
F	-1.189323	-2.792879	-6.265983
F	-0.178664	-4.744844	-6.288702
F	1.709651	-3.929758	-4.712200
F	1.168440	-2.010200	-5.623490
C	0.488153	-3.937296	-0.509063
C	-1.131391	-0.227006	-1.814511
C	4.209588	0.336722	-2.361584
C	5.276177	-0.385525	-1.795417
C	6.575132	0.110427	-1.827720
C	6.870154	1.352058	-2.435063
C	5.800126	2.080734	-3.003403
C	4.504507	1.578459	-2.949624
C	7.016944	1.993289	2.356962
C	5.957065	2.020069	3.292291

C	4.662138	2.303719	2.873655
C	4.353475	2.580315	1.528221
C	5.411153	2.531863	0.598617
C	6.708221	2.247647	1.000544
C	2.997166	2.938949	1.105668
C	2.590259	3.811744	0.126015
C	1.162217	3.922111	-0.008436
C	0.468133	3.118061	0.879584
S	1.581817	2.245690	1.888064
C	0.522079	4.952458	-0.820747
C	1.113767	6.344120	-0.796386
C	-0.023113	7.259474	-1.298006
C	-0.891222	6.306097	-2.144039
C	-0.565746	4.926874	-1.630107
C	-1.291134	3.804483	-2.256853
C	-2.622398	3.374011	-1.937044
C	-3.141208	2.450161	-2.817899
S	-1.969605	2.156530	-4.107773
C	-0.786582	3.224926	-3.402512
F	2.176532	6.432787	-1.663935
F	1.562311	6.741582	0.421819
F	0.428780	8.331760	-1.995887
F	-0.736186	7.715241	-0.235350
F	-2.212917	6.616317	-2.044208
F	-0.552593	6.445736	-3.467643
C	-1.008729	2.973076	1.083030
C	0.538864	3.449575	-4.067677
C	-4.424589	1.747644	-2.789648
C	-4.661946	0.610824	-3.587662
C	-5.887576	-0.045401	-3.608207
C	-6.948309	0.418585	-2.799909
C	-6.708163	1.536309	-1.966869
C	-5.485392	2.186395	-1.964773
C	-10.171033	-1.473468	-3.259850
C	-10.786431	-2.489352	-4.042706
C	-12.088541	-2.874169	-3.792260
C	-12.830182	-2.275979	-2.744935
C	-12.271918	-1.279271	-1.944844
C	-10.913918	-0.847852	-2.225777
N	-8.669551	-2.432627	1.753338
N	-7.792219	2.540013	1.219992
N	-8.210906	-0.146910	-2.755419
C	-8.761342	-1.135277	-3.569413

C	-7.989120	3.785816	0.635371
C	-9.247567	-2.727691	2.984179
O	-7.088520	4.639839	0.574765
O	-8.712374	-3.488345	3.805712
O	-8.114136	-1.683150	-4.474655
H	-8.629519	1.932366	1.167025
H	-9.259483	-1.809413	1.173564
H	-8.891161	0.240030	-2.076828
C	12.245163	0.379336	5.231461
C	10.940155	0.823686	5.167284
C	10.325957	1.084565	3.909742
C	11.074189	0.903616	2.717537
C	12.434477	0.396916	2.791202
C	12.990693	0.160227	4.048589
O	10.621116	1.156502	1.508538
O	13.069951	0.189928	1.656793
O	10.533968	1.301259	-1.271601
O	13.092244	-0.607857	-1.166776
O	12.875581	2.207515	-0.454282
O	10.873802	-1.218522	0.081528
C	11.875407	4.960695	-2.666613
C	10.618020	4.536196	-3.043643
C	10.097025	3.298548	-2.569024
C	10.893344	2.483029	-1.724194
C	12.198028	2.960487	-1.296206
C	12.663106	4.179311	-1.788673
C	11.033535	-3.561355	-0.499450
C	11.801394	-4.561072	-1.158247
C	12.978846	-4.229696	-1.800362
C	13.443194	-2.892156	-1.818115
C	12.728683	-1.873416	-1.185327
C	11.495869	-2.220574	-0.503603
N	8.183681	1.807383	-2.396942
N	8.334374	1.703084	2.674345
N	9.043874	-2.931196	0.706278
C	9.747143	-3.974002	0.116533
C	8.903394	1.494988	3.929989
C	8.704052	2.976882	-2.952226
O	8.255123	1.635264	4.979156
O	8.029102	3.708660	-3.693724
O	9.319759	-5.139590	0.090921
H	9.014146	1.598381	1.901064
H	8.879845	1.269285	-1.856063

H	9.550220	-2.029515	0.652454
H	-11.011437	6.419313	-1.677144
H	-8.806572	5.970532	-0.587029
H	-12.777587	4.635482	-1.698735
H	-7.590672	0.072801	1.979213
H	-5.649646	-1.028508	2.977487
H	-3.447742	2.669601	2.738729
H	-5.410214	3.767549	1.721201
H	-3.468084	-1.990749	2.853371
H	2.985166	-3.948211	3.399320
H	0.427542	0.235698	6.034140
H	0.730537	-1.429362	5.489611
H	1.137061	-0.073742	4.437443
H	-0.318103	0.026328	2.226740
H	0.156113	-0.241408	0.556420
H	-0.885549	-1.408971	1.383848
H	4.867464	-4.672261	1.617021
H	7.263261	-4.974328	1.003565
H	7.860891	-0.733042	1.362763
H	5.501987	-0.427467	1.952587
H	-12.812690	-1.761113	5.779341
H	-10.691242	-2.943914	5.180716
H	-13.831509	-0.132759	4.163305
H	-7.757769	-1.548398	-0.502053
H	-5.526107	-1.864102	-1.434638
H	-4.476703	-4.330484	1.947170
H	-6.720474	-3.958277	2.904606
H	-3.716379	-2.884680	-2.508831
H	2.981375	-2.061210	-3.423028
H	0.907545	-4.431074	-1.392999
H	1.049788	-3.006780	-0.364844
H	0.668370	-4.566601	0.366360
H	-1.450324	-0.844572	-0.969359
H	-1.765472	-0.506065	-2.661043
H	-1.337782	0.817100	-1.570984
H	5.081140	-1.330721	-1.296423
H	7.374529	-0.454994	-1.355237
H	6.013822	3.029515	-3.473227
H	3.695987	2.157104	-3.392502
H	6.180680	1.822996	4.330731
H	3.870103	2.334709	3.619651
H	5.207958	2.687553	-0.455591
H	7.502179	2.207403	0.259420

H	3.286900	4.385091	-0.474067
H	-3.159506	3.713074	-1.059531
H	-1.525491	3.907888	0.840269
H	-1.242275	2.703899	2.116769
H	-1.436409	2.195261	0.439376
H	0.417816	3.789633	-5.104170
H	1.108196	4.211780	-3.529223
H	1.137457	2.531279	-4.079351
H	-3.862068	0.216333	-4.211846
H	-6.054009	-0.906183	-4.240622
H	-7.508782	1.900720	-1.330630
H	-5.372744	3.057302	-1.324863
H	-10.192530	-2.947616	-4.827848
H	-12.552286	-3.656008	-4.396815
H	-13.851411	-2.589379	-2.531138
H	12.707111	0.179590	6.200419
H	10.339921	0.973864	6.059547
H	14.013804	-0.211444	4.090178
H	12.264230	5.912507	-3.033791
H	9.982259	5.131434	-3.691695
H	13.647503	4.515555	-1.465410
H	11.420388	-5.578190	-1.143902
H	13.560272	-5.003420	-2.306184
H	14.368696	-2.626809	-2.327959

****

ΔΔ_open_conformer_3_gas_phase

Ga	4.994072	6.654556	7.431235
Ga	-3.273912	-7.313307	-9.478021
C	8.956541	6.828712	4.115471
C	8.503972	5.575054	3.754991
C	7.429014	4.966655	4.460893
C	6.843135	5.653975	5.556025
C	7.280470	6.997859	5.890275
C	8.344210	7.544253	5.173600
C	5.065240	2.042737	4.382129
C	3.880764	1.879424	5.137583
C	3.042443	0.796811	4.929724
C	3.341201	-0.181677	3.957034
C	4.522431	-0.010729	3.210209
C	5.373384	1.070634	3.406691
C	2.423490	-1.294048	3.710103

C	1.072246	-1.371101	3.945374
C	0.461717	-2.583738	3.471156
C	1.359339	-3.466978	2.908863
S	2.974287	-2.805010	2.972569
C	-0.980285	-2.839688	3.542792
C	-1.628641	-2.821088	4.898825
C	-3.138528	-3.006433	4.636704
C	-3.190728	-3.560334	3.194249
C	-1.867073	-3.189684	2.563851
C	-1.750880	-3.280399	1.111222
C	-2.646314	-4.113822	0.339948
C	-2.457364	-4.044941	-1.014700
S	-1.162114	-2.923918	-1.373255
C	-0.876662	-2.559962	0.298595
F	-1.396452	-1.671889	5.599583
F	-1.174642	-3.849402	5.687491
F	-3.782146	-1.815910	4.695055
F	-3.725601	-3.843821	5.531257
F	-3.359020	-4.925327	3.244597
F	-4.281402	-3.067308	2.538299
C	1.087928	-4.770985	2.223995
C	0.140010	-1.513567	0.636981
C	-3.191255	-4.664206	-2.123634
C	-4.597087	-4.665107	-2.143209
C	-5.315762	-5.129467	-3.241560
C	-4.630771	-5.612786	-4.377468
C	-3.217564	-5.661545	-4.340593
C	-2.515814	-5.184068	-3.246016
O	5.882086	5.165871	6.307442
O	6.638248	7.621656	6.861194
O	3.569962	5.226500	7.863334
O	3.914394	8.220605	8.020085
O	5.621071	6.127760	9.246698
O	3.872773	6.959858	5.710437
C	4.335663	3.579879	11.569181
C	3.280179	3.074876	10.835031
C	2.980825	3.599810	9.547856
C	3.773170	4.656203	9.029966
C	4.896643	5.168974	9.793551
C	5.144839	4.620925	11.052191
C	0.762370	3.149705	6.539958
C	0.772988	3.912432	5.348506
C	-0.030575	3.573463	4.272764

C	-0.893073	2.457109	4.330379
C	-0.900878	1.704702	5.520870
C	-0.098481	2.033680	6.609916
C	-1.715112	2.088465	3.175670
C	-2.161087	2.862130	2.130843
C	-2.838134	2.118774	1.104165
C	-2.954962	0.771563	1.384393
S	-2.210057	0.416689	2.916440
C	-3.377121	2.696166	-0.127616
C	-4.409572	3.784436	-0.047495
C	-4.667761	4.195244	-1.513783
C	-4.173671	2.974773	-2.324137
C	-3.186569	2.287312	-1.413328
C	-2.213772	1.368008	-1.996662
C	-2.505953	0.566861	-3.159067
C	-1.421730	-0.089051	-3.679301
S	0.003242	0.285445	-2.719507
C	-0.878592	1.305444	-1.621598
F	-4.009415	4.856784	0.697635
F	-5.580477	3.338969	0.512042
F	-3.916831	5.281156	-1.830952
F	-5.968947	4.497303	-1.756782
F	-5.244077	2.159678	-2.609913
F	-3.652991	3.365056	-3.517930
C	-3.602536	-0.301242	0.560617
C	-0.174556	1.982664	-0.486527
C	-1.322718	-0.951544	-4.865650
C	-2.226824	-2.005158	-5.101497
C	-2.144494	-2.776007	-6.255707
C	-1.156050	-2.525979	-7.234680
C	-0.237098	-1.479462	-6.993925
C	-0.324564	-0.725465	-5.829819
C	0.327389	-6.564561	-5.194392
C	0.843487	-6.716865	-3.887613
C	1.558028	-5.682682	-3.294220
C	1.808899	-4.469012	-3.960851
C	1.275049	-4.314995	-5.254390
C	0.548281	-5.334630	-5.855791
C	2.614304	-3.414683	-3.331860
C	3.641383	-2.663271	-3.847433
C	4.215573	-1.731739	-2.911044
C	3.611283	-1.777271	-1.663410
S	2.349555	-2.967176	-1.652629

C	5.419203	-0.935870	-3.154937
C	6.661477	-1.596338	-3.693406
C	7.682408	-0.444350	-3.857710
C	7.135488	0.668133	-2.932866
C	5.671460	0.355078	-2.809840
C	4.719398	1.387195	-2.403219
C	4.832010	2.180474	-1.212203
C	3.801221	3.076505	-1.035458
S	2.720618	2.990803	-2.434202
C	3.617321	1.696984	-3.173927
F	6.460116	-2.232802	-4.887471
F	7.164792	-2.532744	-2.833065
F	7.673920	-0.001820	-5.144169
F	8.949733	-0.817701	-3.550316
F	7.771965	0.605772	-1.718991
F	7.393334	1.901185	-3.449270
C	3.923427	-1.007143	-0.416638
C	3.169116	1.074837	-4.462292
C	3.504086	3.952500	0.099154
C	2.290754	4.663342	0.188953
C	1.975162	5.474484	1.272850
C	2.889576	5.608892	2.339247
C	4.111728	4.902473	2.257302
C	4.411439	4.094574	1.174069
C	1.775842	7.969099	5.052525
C	0.752453	8.893388	5.400822
C	0.810704	9.586026	6.593805
C	1.878930	9.377807	7.499613
C	2.902414	8.476026	7.210913
C	2.863946	7.770858	5.941950
N	1.631655	3.542666	7.544071
N	5.847206	3.150565	4.664280
N	2.684118	6.383711	3.467687
C	1.636995	7.252192	3.761731
C	6.932810	3.660609	3.963111
C	1.860880	2.980176	8.795142
O	7.414856	3.085024	2.971946
O	1.188055	2.034210	9.234325
O	0.670014	7.404855	2.998937
H	5.547615	3.763083	5.443884
H	2.258134	4.342401	7.343380
H	3.412329	6.366319	4.203763
C	-2.693654	-11.721356	-6.783274

C	-1.814455	-10.968077	-6.031821
C	-1.538733	-9.616199	-6.381826
C	-2.160965	-9.052027	-7.524778
C	-3.117640	-9.837050	-8.286616
C	-3.349250	-11.158629	-7.904408
O	-1.944226	-7.830485	-7.961932
O	-3.714186	-9.250333	-9.303878
O	-2.271273	-5.490734	-9.402328
O	-4.933914	-6.871833	-10.506709
O	-2.139852	-7.517324	-11.088743
O	-4.456798	-6.635718	-7.941257
C	0.539287	-5.399695	-12.437973
C	0.534074	-4.370909	-11.518591
C	-0.393333	-4.368497	-10.437931
C	-1.338052	-5.421024	-10.326294
C	-1.293207	-6.524451	-11.271676
C	-0.366952	-6.479299	-12.312922
C	-6.754862	-6.085252	-7.427700
C	-8.065516	-5.899667	-7.948023
C	-8.309364	-6.044748	-9.300022
C	-7.262371	-6.377103	-10.193656
C	-5.958642	-6.571615	-9.734788
C	-5.702112	-6.429283	-8.313558
N	-1.125269	-3.341827	-8.359716
N	-0.410259	-7.534853	-5.857062
N	-5.236285	-6.017457	-5.554624
C	-6.549663	-5.848972	-5.976182
C	-0.630490	-8.861013	-5.488178
C	-0.267286	-3.266638	-9.457106
O	-0.106078	-9.361765	-4.480719
O	0.561917	-2.351501	-9.583305
O	-7.461037	-5.507508	-5.205101
H	-0.871475	-7.280669	-6.747713
H	-1.794865	-4.126349	-8.420835
H	-4.608837	-6.321052	-6.320108
H	9.781480	7.287947	3.567982
H	8.933625	5.026407	2.922326
H	8.677420	8.547400	5.437211
H	3.626921	2.619284	5.892455
H	2.142261	0.711272	5.530622
H	4.775436	-0.729059	2.433497
H	6.268321	1.195478	2.813713
H	0.506399	-0.548683	4.364845

H	-3.391248	-4.759233	0.786967
H	1.883307	-5.502529	2.408312
H	0.140534	-5.192347	2.573931
H	0.999793	-4.636548	1.136931
H	-0.181082	-0.913578	1.488997
H	1.107616	-1.953908	0.895747
H	0.300298	-0.848710	-0.211715
H	-5.132881	-4.248283	-1.292522
H	-6.396204	-5.092764	-3.267629
H	-2.678033	-6.064150	-5.194031
H	-1.430054	-5.216027	-3.261750
H	4.559115	3.169961	12.556189
H	2.657799	2.265250	11.204910
H	5.986101	5.012554	11.623133
H	1.443526	4.763947	5.269211
H	0.038142	4.167192	3.365519
H	-1.554369	0.838972	5.605338
H	-0.114736	1.447089	7.518024
H	-1.985641	3.929263	2.069034
H	-3.491990	0.509806	-3.604988
H	-3.981873	-1.116643	1.180544
H	-4.432794	0.117551	-0.018372
H	-2.895916	-0.735428	-0.156424
H	-0.539056	3.006890	-0.355743
H	0.903421	2.026019	-0.646130
H	-0.353378	1.463533	0.460746
H	-2.983610	-2.241523	-4.358460
H	-2.841147	-3.597769	-6.400583
H	0.523029	-1.279391	-7.734765
H	0.384957	0.085223	-5.676218
H	0.676139	-7.651285	-3.371896
H	1.955430	-5.828124	-2.291582
H	1.399385	-3.372172	-5.778039
H	0.132218	-5.182798	-6.848345
H	4.001748	-2.785947	-4.861286
H	5.618964	2.030361	-0.482234
H	4.986994	-0.749916	-0.374428
H	3.672530	-1.582628	0.478088
H	3.368493	-0.062531	-0.371163
H	2.581342	1.773680	-5.066623
H	4.034047	0.749753	-5.051083
H	2.549050	0.188230	-4.283131
H	1.553931	4.573479	-0.606556

H	1.038241	6.011614	1.318777
H	4.828880	5.001648	3.065320
H	5.364813	3.573791	1.179959
H	-0.067279	9.022821	4.700598
H	0.020354	10.292845	6.853004
H	1.917948	9.906869	8.451132
H	-2.900674	-12.756975	-6.506025
H	-1.320121	-11.369623	-5.152388
H	-4.062067	-11.739764	-8.488282
H	1.256226	-5.393399	-13.261278
H	1.238491	-3.546897	-11.576042
H	-0.354446	-7.308928	-13.018783
H	-8.850915	-5.630946	-7.247451
H	-9.318344	-5.897393	-9.690892
H	-7.450234	-6.486446	-11.261242

****

ΔΔ_open_conformer_4_gas_phase

Ga	-11.645873	-0.318334	-0.314712
Ga	11.806279	-0.452609	0.119804
C	-11.757883	4.768812	-1.312788
C	-10.506025	4.762187	-0.732279
C	-9.971195	3.565740	-0.176563
C	-10.740926	2.373662	-0.218532
C	-12.045701	2.381253	-0.861985
C	-12.526411	3.581766	-1.381756
C	-6.899884	2.249995	1.587754
C	-6.653599	0.969303	2.133637
C	-5.438409	0.656748	2.730884
C	-4.406933	1.613850	2.823833
C	-4.665355	2.893168	2.294270
C	-5.873744	3.216422	1.687651
C	-3.087220	1.298725	3.385463
C	-2.693323	0.459580	4.400522
C	-1.264252	0.401268	4.585553
C	-0.565070	1.224652	3.719396
S	-1.665663	2.046802	2.666434
C	-0.586450	-0.420508	5.583420
C	-1.081228	-0.491665	6.999335
C	0.130345	-1.024926	7.801375
C	0.984838	-1.757768	6.737876
C	0.519109	-1.202436	5.423019

C	1.132357	-1.625602	4.162821
C	2.544308	-1.623586	3.869761
C	2.853406	-2.076684	2.608347
S	1.367505	-2.516556	1.772649
C	0.359560	-2.111172	3.122566
F	-2.131462	-1.370915	7.124454
F	-1.510985	0.699448	7.498309
F	-0.231329	-1.832137	8.831500
F	0.826578	0.023548	8.314400
F	2.318745	-1.559208	6.966503
F	0.772609	-3.107857	6.835492
C	0.909166	1.468956	3.614478
C	-1.120595	-2.342098	3.057914
C	4.137883	-2.255018	1.923221
C	4.285657	-3.246513	0.933191
C	5.484283	-3.467960	0.270385
C	6.623574	-2.695823	0.587938
C	6.484213	-1.691049	1.573221
C	5.271915	-1.469691	2.215891
O	-10.351889	1.222445	0.278563
O	-12.710257	1.241426	-0.916523
O	-10.243845	-1.506294	0.615018
O	-12.628891	-1.715475	-1.360637
O	-12.587567	-0.610048	1.411966
O	-10.424989	-0.428533	-1.958784
C	-11.241141	-1.838184	4.606833
C	-9.973112	-2.219599	4.216040
C	-9.569360	-2.095782	2.857288
C	-10.493461	-1.604392	1.901598
C	-11.803069	-1.145080	2.329098
C	-12.153396	-1.293967	3.670309
C	-6.556429	-2.369043	0.610715
C	-6.407830	-1.965784	-0.737415
C	-5.193652	-2.098324	-1.398166
C	-4.068050	-2.647294	-0.749448
C	-4.225614	-3.035193	0.595243
C	-5.429227	-2.902589	1.271721
C	-2.780494	-2.825198	-1.425127
C	-2.467646	-3.062339	-2.743016
C	-1.055645	-3.215324	-2.989417
C	-0.284334	-3.116453	-1.843535
S	-1.296945	-2.782990	-0.474412
C	-0.445002	-3.512246	-4.284945

C	-0.931841	-4.654532	-5.132434
C	-0.071157	-4.585894	-6.418837
C	1.156478	-3.742013	-5.998789
C	0.673833	-2.949623	-4.818945
C	1.376493	-1.745028	-4.380252
C	2.798354	-1.653282	-4.183116
C	3.226461	-0.415883	-3.768619
S	1.841376	0.670984	-3.651980
C	0.712872	-0.557213	-4.139487
F	-2.261784	-4.572382	-5.434783
F	-0.743255	-5.865727	-4.523474
F	-0.751398	-3.931361	-7.396715
F	0.266805	-5.812570	-6.890412
F	2.191031	-4.580205	-5.656486
F	1.601825	-2.978081	-7.034118
C	1.190371	-3.322364	-1.664719
C	-0.751339	-0.262810	-4.247589
C	4.577393	0.054264	-3.443402
C	5.504919	-0.760859	-2.761237
C	6.796099	-0.317397	-2.500633
C	7.226340	0.967147	-2.911568
C	6.290520	1.798618	-3.567630
C	5.003335	1.342047	-3.817972
C	7.162953	1.792027	2.133495
C	6.148002	2.252176	2.998994
C	4.855855	2.435106	2.514414
C	4.522911	2.171747	1.174993
C	5.543599	1.734359	0.311163
C	6.839000	1.565334	0.775307
C	3.130742	2.317370	0.730198
C	2.288357	3.383540	0.877252
C	0.938070	3.157724	0.413799
C	0.772597	1.868568	-0.093459
S	2.262085	0.978424	0.008762
C	-0.077278	4.196658	0.581174
C	0.148235	5.241562	1.655785
C	-1.220267	5.928800	1.861030
C	-1.954667	5.650394	0.537416
C	-1.251209	4.461410	-0.059524
C	-1.847544	3.862945	-1.255844
C	-3.164731	3.295306	-1.263931
C	-3.543030	2.776786	-2.477852
S	-2.266740	3.074272	-3.662915

C	-1.214053	3.810784	-2.478281
F	1.063049	6.188042	1.253871
F	0.607401	4.744105	2.838868
F	-1.097698	7.254001	2.131121
F	-1.878214	5.349313	2.894662
F	-3.287321	5.458420	0.747606
F	-1.846715	6.751681	-0.278570
C	-0.430406	1.162504	-0.642541
C	0.182171	4.233053	-2.819586
C	-4.773051	2.059527	-2.805548
C	-4.850153	1.125054	-3.855054
C	-6.007145	0.392311	-4.110132
C	-7.138730	0.564747	-3.286084
C	-7.073156	1.514101	-2.239104
C	-5.925435	2.247903	-2.010570
C	-10.018703	-1.755905	-3.936064
C	-10.510202	-2.787285	-4.781580
C	-11.698991	-3.426719	-4.485420
C	-12.443026	-3.080009	-3.330882
C	-12.002626	-2.078835	-2.463817
C	-10.771619	-1.385574	-2.791932
N	-7.801371	-2.220074	1.202577
N	-8.130980	2.474045	0.982336
N	-8.321853	-0.140901	-3.390949
C	-8.709466	-1.139125	-4.274593
C	-8.610381	3.648543	0.405702
C	-8.166675	-2.442637	2.528627
O	-7.935411	4.690703	0.379701
O	-7.363701	-2.864809	3.376754
O	-8.016299	-1.481275	-5.245319
H	-8.798565	1.688190	0.918706
H	-8.582301	-1.871672	0.619884
H	-9.027612	0.031036	-2.650479
C	12.059822	-0.176365	5.304492
C	10.877161	0.524615	5.168521
C	10.316005	0.747234	3.880742
C	10.999039	0.263863	2.736856
C	12.218716	-0.508153	2.884437
C	12.727632	-0.698270	4.169932
O	10.591944	0.453338	1.500608
O	12.768754	-0.988877	1.786742
O	10.642155	0.554743	-1.286145
O	12.707468	-1.747929	-1.083411

O	13.027192	1.067773	-0.279682
O	10.307882	-1.883132	-0.001227
C	12.558537	4.053896	-2.368207
C	11.288968	3.824275	-2.857802
C	10.578343	2.643327	-2.500178
C	11.192956	1.691324	-1.647118
C	12.517079	1.956217	-1.107678
C	13.171528	3.126565	-1.491501
C	9.658113	-3.748927	-1.394008
C	10.077217	-4.693842	-2.372856
C	11.358406	-4.652907	-2.884061
C	12.273396	-3.661104	-2.456456
C	11.910047	-2.710327	-1.503753
C	10.576259	-2.773221	-0.930547
N	8.529602	1.345936	-2.627164
N	8.460377	1.503120	2.528603
N	7.863466	-2.861268	-0.015388
C	8.253364	-3.837066	-0.934320
C	8.990392	1.412323	3.812034
C	9.202567	2.501382	-3.026942
O	8.383622	1.819906	4.815122
O	8.676917	3.349596	-3.765948
O	7.469819	-4.707239	-1.345653
H	9.109505	1.152891	1.802417
H	9.117324	0.712919	-2.057720
H	8.636282	-2.231593	0.259438
H	-12.158935	5.691926	-1.735612
H	-9.890754	5.655413	-0.685530
H	-13.507459	3.574943	-1.855494
H	-7.423155	0.207048	2.059959
H	-5.282643	-0.355752	3.093563
H	-3.906820	3.668208	2.365776
H	-6.046293	4.205918	1.290163
H	-3.390831	-0.117600	4.997551
H	3.294585	-1.301478	4.581715
H	1.120952	2.501774	3.333837
H	1.402907	1.254636	4.567294
H	1.371170	0.821691	2.860361
H	-1.350503	-3.267678	2.521835
H	-1.541177	-2.414279	4.064741
H	-1.642797	-1.524707	2.546514
H	3.441852	-3.887953	0.688364
H	5.575527	-4.242722	-0.476494

H	7.339937	-1.068783	1.819564
H	5.198334	-0.653174	2.929191
H	-11.541990	-1.935622	5.651566
H	-9.245424	-2.604484	4.923936
H	-13.142763	-0.959619	3.980554
H	-7.257171	-1.531184	-1.258122
H	-5.110453	-1.738155	-2.419743
H	-3.387277	-3.481791	1.125326
H	-5.530372	-3.212486	2.301468
H	-3.216906	-3.146231	-3.520842
H	3.474464	-2.486120	-4.341101
H	1.734335	-2.374959	-1.569878
H	1.397208	-3.911625	-0.767361
H	1.609384	-3.853109	-2.523283
H	-1.225739	-0.941380	-4.963749
H	-0.933183	0.764952	-4.569878
H	-1.265704	-0.402322	-3.288786
H	5.203566	-1.742404	-2.404810
H	7.484810	-0.964439	-1.962708
H	6.606327	2.783213	-3.879505
H	4.312124	1.996526	-4.345770
H	6.393193	2.425800	4.037536
H	4.070818	2.759270	3.194586
H	5.320889	1.526527	-0.731629
H	7.617022	1.228601	0.094867
H	2.625367	4.321722	1.297888
H	-3.775372	3.205804	-0.373887
H	-0.561059	1.370505	-1.710610
H	-1.343465	1.477878	-0.136382
H	-0.338302	0.078765	-0.522489
H	0.252646	4.632594	-3.837961
H	0.521066	5.004718	-2.120856
H	0.885425	3.393357	-2.739526
H	-3.971027	0.939053	-4.470184
H	-6.055240	-0.331585	-4.912919
H	-7.941214	1.659462	-1.602108
H	-5.927708	2.981233	-1.210017
H	-9.913998	-3.053864	-5.649625
H	-12.069050	-4.218495	-5.139788
H	-13.371317	-3.596167	-3.088051
H	12.482212	-0.345230	6.297097
H	10.333078	0.906538	6.027416
H	13.648563	-1.270679	4.276009

H	13.095201	4.963229	-2.646461
H	10.790050	4.531016	-3.513957
H	14.165953	3.309797	-1.086071
H	9.351020	-5.430129	-2.703071
H	11.669638	-5.380494	-3.636002
H	13.278996	-3.607808	-2.871978

****

ΔΔ_open_conformer_5_gas_phase

Ga	11.306304	0.024515	0.188178
Ga	-11.311178	-0.036004	-0.152004
C	11.121194	5.161798	0.778514
C	9.900133	5.033178	0.147083
C	9.465218	3.765837	-0.331367
C	10.306793	2.636816	-0.166543
C	11.570943	2.768444	0.537678
C	11.954229	4.034081	0.979451
C	6.556287	2.036805	-1.996853
C	6.405446	0.671408	-2.336141
C	5.242352	0.200421	-2.931857
C	4.171176	1.071272	-3.223928
C	4.335885	2.428913	-2.887485
C	5.490002	2.914237	-2.288555
C	2.910332	0.602112	-3.808436
C	2.627644	-0.419907	-4.684114
C	1.223263	-0.567500	-4.971441
C	0.430903	0.372751	-4.337669
S	1.410354	1.404527	-3.346994
C	0.623166	-1.592478	-5.829010
C	1.036996	-1.765608	-7.260709
C	0.216171	-2.979417	-7.763237
C	-0.949759	-3.084766	-6.749182
C	-0.433202	-2.394642	-5.520458
C	-1.037355	-2.605381	-4.206525
C	-2.452156	-2.572856	-3.932907
C	-2.766234	-2.778184	-2.610564
S	-1.282539	-3.021564	-1.690178
C	-0.266926	-2.866845	-3.087451
F	2.377959	-1.988196	-7.410338
F	0.738778	-0.667462	-8.024063
F	0.971013	-4.108086	-7.699320
F	-0.209042	-2.831860	-9.044357

F	-2.055683	-2.447926	-7.260073
F	-1.305990	-4.383204	-6.548640
C	-1.051164	0.584175	-4.430866
C	1.217285	-3.048614	-2.977455
C	-4.050155	-2.806972	-1.901859
C	-4.227101	-3.654018	-0.790854
C	-5.408341	-3.691274	-0.063117
C	-6.488741	-2.859973	-0.428568
C	-6.324239	-2.007783	-1.545977
C	-5.134771	-1.980411	-2.262874
O	10.016997	1.432062	-0.601688
O	12.286760	1.673144	0.716080
O	10.011009	-1.340721	-0.667151
O	12.262101	-1.250866	1.380142
O	12.317861	-0.359259	-1.481200
O	9.969083	-0.011446	1.763516
C	11.188312	-2.015402	-4.565546
C	9.951469	-2.480832	-4.165924
C	9.492224	-2.260502	-2.837458
C	10.324752	-1.568282	-1.921999
C	11.607777	-1.044416	-2.358858
C	12.014477	-1.295009	-3.668669
C	6.543294	-2.844772	-0.559883
C	6.376504	-2.478482	0.796486
C	5.203869	-2.764635	1.483974
C	4.138186	-3.439922	0.851710
C	4.322291	-3.812673	-0.494279
C	5.485077	-3.528184	-1.196244
C	2.860308	-3.704395	1.521832
C	2.537742	-3.941279	2.837863
C	1.123888	-4.099826	3.071139
C	0.364587	-4.019347	1.917536
S	1.386287	-3.698707	0.555751
C	0.484304	-4.316330	4.371059
C	0.869584	-5.464516	5.256691
C	0.010281	-5.283086	6.532546
C	-1.140990	-4.353592	6.075523
C	-0.586447	-3.641825	4.875877
C	-1.172995	-2.398899	4.380164
C	-2.583939	-2.167284	4.192064
C	-2.880226	-0.920508	3.694146
S	-1.384235	-0.018344	3.459403
C	-0.388479	-1.307813	4.049687

F	2.201897	-5.483392	5.565013
F	0.586830	-6.678252	4.686789
F	0.737082	-4.658202	7.496437
F	-0.432067	-6.462403	7.040081
F	-2.237935	-5.115496	5.749741
F	-1.528249	-3.524820	7.083589
C	-1.115335	-4.182652	1.736259
C	1.094881	-1.127194	4.170876
C	-4.148514	-0.276615	3.335469
C	-5.240242	-0.989374	2.796825
C	-6.405206	-0.332871	2.420762
C	-6.537193	1.069066	2.556699
C	-5.452749	1.787129	3.104163
C	-4.297370	1.116692	3.479821
C	-6.507319	1.763729	-2.420192
C	-5.419028	1.885927	-3.310454
C	-4.263704	2.541361	-2.907579
C	-4.119059	3.101407	-1.623251
C	-5.212865	2.979028	-0.740149
C	-6.376926	2.329408	-1.129993
C	-2.857188	3.738703	-1.232080
C	-2.577093	4.764815	-0.360093
C	-1.170914	5.055975	-0.236640
C	-0.373746	4.268951	-1.048552
S	-1.351671	3.135708	-1.922762
C	-0.574594	6.063673	0.643464
C	-0.981129	7.506607	0.578385
C	-0.166798	8.196019	1.701371
C	0.991780	7.207025	1.980584
C	0.473034	5.884789	1.495711
C	1.066428	4.620382	1.925349
C	2.480293	4.338433	1.960260
C	2.781821	3.064835	2.381144
S	1.287333	2.204708	2.745808
C	0.284420	3.561354	2.351163
F	-2.322789	7.696813	0.762493
F	-0.670074	8.080222	-0.626494
F	-0.930723	8.320557	2.818830
F	0.267647	9.433939	1.350955
F	2.105865	7.601880	1.278493
F	1.337122	7.219489	3.297376
C	1.112099	4.310102	-1.249842
C	-1.202742	3.480949	2.522541

C	4.053951	2.352077	2.542017
C	4.192464	1.376833	3.548911
C	5.350148	0.629130	3.709716
C	6.448202	0.835940	2.847741
C	6.326624	1.823155	1.841497
C	5.158718	2.560129	1.689814
C	9.345426	-1.460501	3.590259
C	9.750914	-2.524979	4.442837
C	10.984808	-3.122140	4.278597
C	11.860355	-2.702685	3.247263
C	11.506925	-1.671034	2.378653
C	10.229435	-1.008042	2.578207
N	7.738419	-2.512738	-1.180762
N	7.744362	2.430557	-1.398687
N	7.641463	0.133519	2.929130
C	7.988250	-0.900258	3.795353
C	8.123715	3.696676	-0.959184
C	8.139010	-2.758320	-2.492055
O	7.384415	4.688330	-1.072492
O	7.409317	-3.340627	-3.311349
O	7.209124	-1.330385	4.662344
H	8.468523	1.710595	-1.231084
H	8.456057	-2.015875	-0.624996
H	8.393174	0.367712	2.257515
C	-11.089075	-1.380933	-5.146237
C	-9.858711	-0.756234	-5.098809
C	-9.424415	-0.106832	-3.909815
C	-10.275610	-0.094949	-2.776008
C	-11.551548	-0.788830	-2.817293
C	-11.933288	-1.403207	-4.009266
O	-9.985760	0.503162	-1.643190
O	-12.279436	-0.794053	-1.715429
O	-10.017143	1.000371	1.083124
O	-12.314358	-0.999229	1.271488
O	-12.292459	1.692742	-0.049954
O	-10.007782	-1.609543	0.153940
C	-11.089582	4.986021	1.064017
C	-9.863678	4.641525	1.597481
C	-9.439030	3.283663	1.612016
C	-10.295851	2.283841	1.085820
C	-11.564072	2.656859	0.482642
C	-11.937302	4.000028	0.502518
C	-9.459878	-3.220673	1.865498

C	-9.908830	-3.902127	3.031001
C	-11.150096	-3.626131	3.568863
C	-11.990247	-2.646517	2.985163
C	-11.593828	-1.943700	1.848151
C	-10.308692	-2.265395	1.251245
N	-7.726719	1.651787	2.145460
N	-7.697541	1.122107	-2.730206
N	-7.703700	-2.826792	0.240084
C	-8.095625	-3.531897	1.375159
C	-8.074913	0.507769	-3.922018
C	-8.091340	2.995529	2.158773
O	-7.329280	0.469249	-4.914376
O	-7.334941	3.885184	2.582921
O	-7.348292	-4.345967	1.942684
H	-8.429974	1.073862	-2.000859
H	-8.461072	1.037372	1.752861
H	-8.435212	-2.183321	-0.108830
H	11.446427	6.138142	1.142816
H	9.234796	5.879487	0.005530
H	12.907508	4.127000	1.498798
H	7.207337	-0.024575	-2.104566
H	5.156675	-0.862959	-3.140781
H	3.540514	3.135258	-3.116696
H	5.597112	3.960624	-2.042307
H	3.390614	-1.060905	-5.110183
H	-3.199503	-2.397083	-4.698840
H	-1.293271	1.648508	-4.505450
H	-1.455609	0.077528	-5.311454
H	-1.577695	0.187058	-3.554932
H	1.463415	-3.900758	-2.337904
H	1.655446	-3.222726	-3.963576
H	1.709637	-2.165136	-2.554592
H	-3.419838	-4.321051	-0.495674
H	-5.525514	-4.352568	0.783038
H	-7.136964	-1.345822	-1.832894
H	-5.038341	-1.282434	-3.090550
H	11.532078	-2.188661	-5.587074
H	9.292214	-3.014480	-4.843809
H	12.981245	-0.906011	-3.986648
H	7.174701	-1.942024	1.301464
H	5.109221	-2.428391	2.513382
H	3.534104	-4.358414	-1.008972
H	5.604622	-3.824505	-2.228196

H	3.277656	-3.990354	3.628207
H	-3.340681	-2.910814	4.417008
H	-1.340501	-4.748216	0.826953
H	-1.550558	-4.711928	2.588777
H	-1.626676	-3.215818	1.657447
H	1.339327	-0.132158	4.553263
H	1.603363	-1.244470	3.206742
H	1.517674	-1.870752	4.851791
H	-5.170937	-2.062132	2.636363
H	-7.224192	-0.901728	1.990199
H	-5.546253	2.856414	3.226099
H	-3.487462	1.696204	3.917507
H	-5.510685	1.468387	-4.302644
H	-3.450748	2.634976	-3.624747
H	-5.147319	3.366344	0.273388
H	-7.197657	2.233794	-0.424728
H	-3.343429	5.297828	0.190461
H	3.235222	5.062362	1.673653
H	1.622699	3.510594	-0.700045
H	1.520411	5.260769	-0.895772
H	1.369437	4.197973	-2.307264
H	-1.463365	2.957805	3.447064
H	-1.639935	4.482387	2.558617
H	-1.683014	2.946813	1.694323
H	3.371790	1.207469	4.242717
H	5.434781	-0.113447	4.489643
H	7.155490	1.988857	1.158946
H	5.096601	3.278810	0.876678
H	9.053295	-2.849391	5.208984
H	11.287381	-3.938333	4.937333
H	12.823795	-3.189034	3.097894
H	-11.413628	-1.877348	-6.062758
H	-9.185723	-0.747712	-5.950859
H	-12.895023	-1.914573	-4.035226
H	-11.406995	6.030473	1.057102
H	-9.186766	5.384553	2.008114
H	-12.893862	4.271251	0.056986
H	-9.237629	-4.628830	3.478592
H	-11.486099	-4.153797	4.463511
H	-12.959335	-2.407270	3.421882

****

ΔΔ_closed_conformer_1_gas_phase

Ga	-9.431819	-12.198880	8.677258
O	-9.338650	-10.938853	7.023736
O	-11.147529	-12.680562	7.802875
C	-12.905063	-11.189198	4.943512
C	-11.958655	-10.283601	4.509055
C	-10.707580	-10.167877	5.176922
C	-10.444439	-10.982257	6.311160
C	-11.441840	-11.938124	6.756015
C	-12.649155	-12.016257	6.061464
C	-7.334200	-8.556796	4.754534
C	-6.095883	-8.901445	5.353745
C	-4.909386	-8.297113	4.963538
C	-4.911107	-7.267601	3.998944
C	-6.147471	-6.912818	3.421558
C	-7.331848	-7.547507	3.763175
C	-3.719777	-6.490651	3.668878
C	-3.666747	-5.119513	3.653132
C	-2.381644	-4.570407	3.408396
C	-1.396515	-5.598207	2.827551
S	-2.163473	-7.259844	3.289268
C	-1.936387	-3.295616	3.611920
C	-2.746114	-2.119306	4.020310
C	-1.746628	-0.908603	4.011324
C	-0.373507	-1.466359	3.498013
C	-0.548704	-2.940363	3.455180
C	0.420961	-3.883639	3.267962
C	1.797149	-3.707542	2.975398
C	2.448599	-4.832408	2.536767
S	1.410332	-6.276677	2.495520
C	0.028777	-5.363861	3.403294
F	-3.302743	-2.242563	5.271240
F	-3.800035	-1.857293	3.178431
F	-1.605081	-0.399664	5.263915
F	-2.202439	0.089643	3.210411
F	-0.095747	-0.911612	2.270085
F	0.640619	-1.051970	4.325182
C	-1.448664	-5.465445	1.285935
C	0.133933	-5.731855	4.903915
C	3.849189	-4.858980	2.124394
C	4.283192	-5.420975	0.907090
C	5.600323	-5.284508	0.495480

C	6.567999	-4.638609	1.304555
C	6.140318	-4.102605	2.543987
C	4.808604	-4.199519	2.918569
N	-8.465766	-9.223662	5.173781
C	-9.740388	-9.205985	4.601383
O	-10.018617	-8.467167	3.644294
H	-8.393879	-9.871757	5.977745
Ga	9.843390	-5.713392	-3.252966
O	9.302188	-5.199690	-1.324554
O	11.654626	-5.575046	-2.431238
C	12.707465	-4.217476	0.811695
C	11.482593	-4.021701	1.414699
C	10.278421	-4.347613	0.729577
C	10.346207	-4.869605	-0.591124
C	11.641452	-5.076143	-1.215334
C	12.789890	-4.743816	-0.497073
N	7.861888	-4.573103	0.825761
C	9.019787	-4.140249	1.478568
O	8.997438	-3.651368	2.619073
H	8.052760	-4.901683	-0.135345
H	-13.859794	-11.274462	4.420431
H	-12.127007	-9.645801	3.646529
H	-13.387721	-12.737317	6.409423
H	-6.083297	-9.642024	6.148977
H	-3.982969	-8.586503	5.453329
H	-6.163060	-6.131376	2.665770
H	-8.267292	-7.291545	3.289520
H	-4.514343	-4.511293	3.947057
H	2.274978	-2.734802	2.978780
H	-0.865183	-6.247457	0.797288
H	-1.048191	-4.490507	0.986021
H	-2.488938	-5.537652	0.957476
H	1.122927	-5.447752	5.273026
H	-0.627732	-5.185741	5.471614
H	-0.012857	-6.801090	5.063415
H	3.571956	-5.911825	0.249545
H	5.895314	-5.653384	-0.483150
H	6.869865	-3.620302	3.174492
H	4.492254	-3.770552	3.866494
H	13.625708	-3.968747	1.347886
H	11.395750	-3.626197	2.421830
H	13.754130	-4.905175	-0.977474
O	9.234058	-7.646790	-2.807889

O	10.545321	-6.665387	-4.857377
C	10.833348	-10.249708	-5.595139
C	10.139765	-10.782460	-4.527529
C	9.557053	-9.930786	-3.547171
C	9.710671	-8.525539	-3.665557
C	10.440197	-7.972785	-4.789793
C	10.981714	-8.850347	-5.730397
C	7.205095	-10.033219	-0.606741
C	6.484410	-8.970727	-0.001787
C	5.546252	-9.203820	0.995102
C	5.326103	-10.511571	1.479969
C	6.057629	-11.559379	0.889933
C	6.960013	-11.346037	-0.139213
C	4.474262	-10.809557	2.628564
C	4.865218	-11.579896	3.692021
C	3.921291	-11.677362	4.745244
C	2.512927	-11.221390	4.328252
S	2.822967	-10.171914	2.789733
C	4.104836	-12.114467	6.025317
C	5.332663	-12.726579	6.594446
C	4.945344	-13.141907	8.058404
C	3.434604	-12.762444	8.246343
C	3.073301	-12.000626	7.024071
C	1.928151	-11.292908	6.798592
C	0.772350	-11.176595	7.612656
C	-0.329271	-10.653113	6.985101
S	-0.024742	-10.182394	5.297654
C	1.829544	-10.467072	5.505007
F	6.405536	-11.867998	6.633802
F	5.773304	-13.827124	5.899982
F	5.712070	-12.477066	8.962250
F	5.143695	-14.472523	8.250191
F	2.699898	-13.914765	8.409268
F	3.268771	-12.055249	9.410903
C	1.730646	-12.483293	3.881455
C	2.485983	-9.095232	5.789890
C	-1.631840	-10.492884	7.620599
C	-2.828581	-10.968618	7.045227
C	-4.017874	-10.931962	7.758233
C	-4.087248	-10.364430	9.054995
C	-2.891723	-9.857863	9.619502
C	-1.700219	-9.947298	8.917238
N	8.095786	-9.722665	-1.616327

C	8.790197	-10.589676	-2.467737
O	8.731750	-11.823918	-2.351918
H	8.270300	-8.726796	-1.833952
O	-7.756562	-11.188652	9.383816
O	-10.303726	-11.031590	10.028532
C	-8.872018	-8.887958	12.641158
C	-7.534214	-8.947201	12.309629
C	-7.100767	-9.714990	11.191100
C	-8.051650	-10.433533	10.422326
C	-9.457257	-10.359583	10.776042
C	-9.832915	-9.589975	11.877035
N	-5.311200	-10.345419	9.691891
C	-5.659150	-9.686600	10.875357
O	-4.823445	-9.089133	11.571994
H	-6.111262	-10.824596	9.247307
H	11.268044	-10.910229	-6.348118
H	10.003984	-11.852602	-4.405475
H	11.524021	-8.419801	-6.571186
H	6.690188	-7.950534	-0.314993
H	5.020340	-8.360256	1.435534
H	5.887638	-12.573433	1.244554
H	7.489173	-12.163155	-0.602945
H	5.873502	-11.969964	3.772553
H	0.723306	-11.575442	8.619424
H	0.750864	-12.227136	3.475595
H	1.588286	-13.152192	4.737570
H	2.308417	-13.007403	3.115153
H	2.036023	-8.663426	6.687672
H	3.560147	-9.231922	5.960720
H	2.351491	-8.404728	4.955664
H	-2.817742	-11.420972	6.056842
H	-4.913869	-11.367867	7.326739
H	-2.936508	-9.410008	10.599909
H	-0.787318	-9.565810	9.368898
H	-9.197042	-8.293146	13.497391
H	-6.778621	-8.410717	12.874269
H	-10.892060	-9.546519	12.128009
O	-8.105161	-13.431421	7.652427
O	-9.478294	-13.795777	9.868844
C	-8.208153	-17.202247	9.355959
C	-7.470160	-17.027153	8.203925
C	-7.389134	-15.746439	7.587622
C	-8.091355	-14.649448	8.152547

C	-8.860624	-14.842901	9.369481
C	-8.897484	-16.115100	9.941384
C	-5.528296	-13.884649	4.918031
C	-5.354286	-12.487174	4.770863
C	-4.507925	-11.957535	3.809449
C	-3.831902	-12.803004	2.903952
C	-4.020008	-14.192737	3.041350
C	-4.828776	-14.734949	4.028440
C	-3.051744	-12.296284	1.780280
C	-3.147525	-12.769526	0.495763
C	-2.355565	-12.082281	-0.457033
C	-1.274548	-11.201236	0.190402
S	-1.893557	-10.958442	1.955658
C	-2.425889	-12.116332	-1.821017
C	-3.278260	-13.005353	-2.650945
C	-2.910025	-12.657801	-4.136003
C	-1.710691	-11.651386	-4.070552
C	-1.637569	-11.236559	-2.646735
C	-0.913662	-10.206733	-2.117884
C	0.012680	-9.339992	-2.755461
C	0.818149	-8.625952	-1.905670
S	0.448105	-8.897066	-0.188448
C	-1.096671	-9.888738	-0.623478
F	-4.625669	-12.816281	-2.456905
F	-3.063860	-14.343969	-2.422495
F	-3.967994	-12.078550	-4.762762
F	-2.580311	-13.777081	-4.832661
F	-0.572052	-12.286277	-4.513431
F	-1.916262	-10.614021	-4.944172
C	0.023137	-12.044847	0.262639
C	-2.314335	-8.940390	-0.499721
C	1.899306	-7.745296	-2.337223
C	3.214005	-7.853278	-1.838670
C	4.242030	-7.099353	-2.386395
C	4.000235	-6.151649	-3.412635
C	2.674534	-6.021011	-3.891681
C	1.666039	-6.819635	-3.373642
N	-6.373102	-14.330789	5.913126
C	-6.528439	-15.633023	6.393881
O	-5.975410	-16.609397	5.863816
H	-6.931743	-13.642478	6.445422
O	7.691642	-5.328219	-3.781277
O	9.953506	-3.937968	-4.143492

C	7.545589	-1.697643	-5.772501
C	6.369695	-2.394004	-5.587325
C	6.368828	-3.634828	-4.889112
C	7.592497	-4.169394	-4.398749
C	8.828978	-3.427826	-4.603083
C	8.767543	-2.209201	-5.280100
N	5.071383	-5.417929	-3.882450
C	5.053028	-4.280249	-4.691978
O	4.001364	-3.835210	-5.177901
H	6.025531	-5.657489	-3.558997
H	-8.256203	-18.185363	9.828949
H	-6.921777	-17.843272	7.743582
H	-9.476575	-16.244176	10.854998
H	-5.921316	-11.820127	5.410670
H	-4.417998	-10.877524	3.720772
H	-3.496822	-14.858309	2.358570
H	-4.941022	-15.802017	4.143240
H	-3.875975	-13.522457	0.216671
H	0.167968	-9.336241	-3.828217
H	0.813368	-11.520454	0.802633
H	0.378198	-12.262217	-0.750792
H	-0.192794	-12.987813	0.772568
H	-2.173677	-8.091203	-1.173702
H	-3.227193	-9.473775	-0.787036
H	-2.433204	-8.568805	0.520129
H	3.443553	-8.568876	-1.053199
H	5.259546	-7.245681	-2.035336
H	2.476183	-5.288334	-4.658735
H	0.654547	-6.716800	-3.759776
H	7.538223	-0.740731	-6.298712
H	5.416300	-2.018578	-5.945419
H	9.699576	-1.663309	-5.421744

****

ΔΛ_open_conformer_1_gas_phase

Ga	11.978157	0.350754	0.133581
Ga	-12.208160	0.477129	0.417620
C	11.354927	0.862530	5.249536
C	10.080947	1.265331	4.904065
C	9.713710	1.406394	3.536216
C	10.677481	1.138820	2.526695
C	12.002594	0.669781	2.897631

C	12.313416	0.559617	4.251801
C	6.785770	2.191119	1.300647
C	6.733659	2.134175	-0.110664
C	5.561146	2.398748	-0.798211
C	4.374947	2.739807	-0.114641
C	4.431794	2.789257	1.291071
C	5.601470	2.523609	1.995514
C	3.144433	3.018730	-0.856805
C	2.755961	2.572303	-2.096018
C	1.453603	3.028306	-2.506833
C	0.846579	3.853767	-1.581294
S	1.893935	4.084219	-0.205486
C	0.868901	2.640079	-3.791764
C	1.745574	2.720284	-5.017535
C	0.769524	2.586038	-6.206547
C	-0.461037	1.896736	-5.585437
C	-0.386178	2.212631	-4.110539
C	-1.544686	1.924210	-3.259409
C	-2.888055	2.317566	-3.612182
C	-3.838976	1.935162	-2.701105
S	-3.084770	1.049968	-1.393362
C	-1.491111	1.228601	-2.061031
F	2.675249	1.716900	-5.087625
F	2.435713	3.892412	-5.086565
F	1.290031	1.901438	-7.251317
F	0.414670	3.825054	-6.646041
F	-1.601094	2.315746	-6.209442
F	-0.378185	0.542305	-5.801831
C	-0.505309	4.498423	-1.627161
C	-0.315245	0.679038	-1.319027
C	-5.270399	2.235616	-2.608737
C	-5.744329	3.534716	-2.860016
C	-7.063691	3.897266	-2.607723
C	-7.962370	2.950060	-2.072313
C	-7.509747	1.621624	-1.886294
C	-6.191560	1.277272	-2.136575
C	-11.054914	4.394543	-0.565898
C	-11.720954	5.613278	-0.258075
C	-12.828645	5.616082	0.567280
C	-13.324854	4.410403	1.119213
C	-12.710375	3.187176	0.845668
C	-11.541432	3.182996	-0.013156
O	10.458374	1.264542	1.237986

O	12.841590	0.376706	1.921002
O	10.727247	0.709654	-1.467671
O	13.175294	-0.991428	-0.751896
O	12.906055	1.933136	-0.625927
O	10.853108	-1.348542	0.407361
O	-13.112926	2.025957	1.316966
O	-11.002096	1.999121	-0.223888
O	-13.262969	-0.872724	1.383880
O	-11.105833	-0.709002	-0.878693
O	-10.771394	-0.094485	1.854298
O	-13.441541	0.488353	-1.155210
C	12.104330	3.983105	-3.572368
C	10.928043	3.388134	-3.983619
C	10.398820	2.271905	-3.278107
C	11.105833	1.759548	-2.160996
C	12.321851	2.412113	-1.708777
C	12.798351	3.502879	-2.435255
C	7.307450	0.148383	-2.960248
C	6.979377	-0.768081	-1.932902
C	5.716870	-1.332863	-1.848869
C	4.716037	-1.033965	-2.798741
C	5.057726	-0.135480	-3.827639
C	6.315101	0.451637	-3.916610
C	3.388843	-1.649232	-2.722027
C	2.968717	-2.752271	-2.019233
C	1.573617	-3.073782	-2.188696
C	0.917298	-2.192607	-3.035594
S	2.024196	-0.992482	-3.629980
C	0.990653	-4.308358	-1.674037
C	1.870043	-5.534315	-1.590943
C	0.880482	-6.719254	-1.525473
C	-0.410169	-6.080717	-0.970448
C	-0.268415	-4.609017	-1.246402
C	-1.371081	-3.725740	-0.856805
C	-2.727656	-3.859169	-1.303050
C	-3.600658	-2.969601	-0.718016
S	-2.738062	-1.993324	0.459927
C	-1.210645	-2.745044	0.104244
F	2.634651	-5.520582	-0.450205
F	2.724799	-5.679245	-2.637214
F	1.331110	-7.747196	-0.766241
F	0.658522	-7.189068	-2.783391
F	-1.516658	-6.634529	-1.559309

F	-0.512916	-6.345454	0.368486
C	-0.509290	-2.175938	-3.495189
C	0.045888	-2.368019	0.829917
C	-5.012771	-2.725359	-1.019085
C	-5.525899	-3.036905	-2.292864
C	-6.852003	-2.809232	-2.634723
C	-7.740504	-2.232319	-1.698574
C	-7.231870	-1.909829	-0.417835
C	-5.904684	-2.150066	-0.090023
C	-11.141810	-1.543833	-3.149091
C	-11.889509	-1.658509	-4.355420
C	-13.133917	-1.072138	-4.465577
C	-13.683358	-0.338658	-3.387622
C	-12.991604	-0.195916	-2.183512
C	-11.694739	-0.838247	-2.049773
C	-12.563884	-2.849188	4.388006
C	-11.255670	-2.488919	4.627425
C	-10.584396	-1.562345	3.775256
C	-11.277405	-0.984678	2.678923
C	-12.653383	-1.391958	2.427532
C	-13.262590	-2.304727	3.286193
C	-7.090576	-0.208676	3.252510
C	-6.237454	-0.645506	4.294634
C	-4.890835	-0.321382	4.291716
C	-4.299444	0.456572	3.278058
C	-5.144053	0.870234	2.226730
C	-6.500877	0.550442	2.212547
C	-2.870975	0.777378	3.382237
C	-2.104077	0.831147	4.524420
C	-0.701931	1.029545	4.290547
C	-0.392872	1.171043	2.949217
S	-1.835804	1.053193	1.984720
C	0.306768	1.130234	5.345868
C	0.109047	2.083479	6.492784
C	1.325702	1.847927	7.419486
C	2.364865	1.167048	6.497573
C	1.537779	0.551662	5.400143
C	2.100498	-0.517086	4.579995
C	3.437349	-0.488953	4.056022
C	3.786877	-1.615464	3.346287
S	2.441511	-2.757218	3.375475
C	1.418457	-1.683623	4.286029
F	-1.060768	1.870117	7.175527

F	0.085721	3.388085	6.086520
F	0.982201	0.989995	8.419072
F	1.782826	2.990936	7.985537
F	3.225717	2.113352	6.007497
F	3.113243	0.267956	7.190927
C	0.938199	1.407726	2.305273
C	0.026255	-2.088310	4.666388
C	5.031865	-1.932533	2.650826
C	5.167187	-3.068556	1.826653
C	6.362689	-3.385745	1.188417
C	7.482136	-2.540881	1.342536
C	7.349038	-1.393081	2.156959
C	6.161071	-1.096632	2.796895
C	10.581378	-3.568348	-0.513026
C	11.181848	-4.607220	-1.275007
C	12.437244	-4.434008	-1.825637
C	13.141065	-3.216368	-1.659478
C	12.593204	-2.162225	-0.927095
C	11.295013	-2.358969	-0.311665
N	8.573000	0.713678	-2.937090
N	8.007504	1.923009	1.897927
N	8.710170	-2.720965	0.738450
C	9.210298	-3.793977	0.010997
C	8.311576	1.790562	3.247141
C	9.087977	1.741885	-3.725536
O	7.457253	1.955194	4.135945
O	8.475775	2.196346	-4.704386
O	8.561366	-4.833813	-0.177084
H	8.811319	1.712727	1.280917
H	9.223009	0.415859	-2.189159
H	9.393464	-1.945209	0.827436
C	-9.169321	-1.302429	4.105906
N	-8.449330	-0.507209	3.204012
N	-9.066266	-1.936112	-1.962790
C	-9.787418	-2.139592	-3.139420
N	-9.255942	3.230732	-1.672546
C	-9.840562	4.466803	-1.414818
O	-9.368957	5.531517	-1.845215
O	-9.313330	-2.756679	-4.106745
O	-8.633659	-1.787153	5.115809
H	-9.617116	-1.451394	-1.232514
H	-9.030323	-0.122530	2.444717
H	-9.780619	2.449856	-1.239653

H	11.627135	0.758091	6.301277
H	9.322320	1.474969	5.651920
H	13.312026	0.216803	4.520091
H	7.633471	1.879160	-0.663730
H	5.567668	2.342737	-1.882379
H	3.530672	3.022076	1.855230
H	5.625178	2.562140	3.075390
H	3.356122	1.885376	-2.680351
H	-3.130413	2.869631	-4.510376
H	-0.499546	5.482047	-1.143438
H	-0.832060	4.624554	-2.663600
H	-1.263881	3.880653	-1.128719
H	0.529489	0.504930	-1.983748
H	0.024417	1.382001	-0.549206
H	-0.568128	-0.261485	-0.826441
H	-5.042430	4.289222	-3.210457
H	-7.411939	4.909806	-2.758856
H	-8.207394	0.870710	-1.522890
H	-5.872188	0.253008	-1.959952
H	-11.320230	6.527509	-0.686396
H	-13.331611	6.556746	0.800973
H	-14.200522	4.409073	1.767151
H	12.501527	4.841215	-4.117994
H	10.365643	3.756471	-4.836401
H	13.715672	3.981845	-2.094708
H	7.724609	-1.012420	-1.180403
H	5.501693	-1.993342	-1.014649
H	4.328357	0.110373	-4.594078
H	6.553073	1.147179	-4.708631
H	3.634112	-3.341607	-1.400109
H	-3.033098	-4.587646	-2.043946
H	-0.628491	-1.579512	-4.404449
H	-0.866640	-3.194402	-3.686143
H	-1.172668	-1.750217	-2.733375
H	0.747778	-1.834943	0.176773
H	0.568343	-3.262253	1.184062
H	-0.157806	-1.726381	1.690206
H	-4.854862	-3.440517	-3.047662
H	-7.226460	-3.045002	-3.620295
H	-7.897899	-1.473174	0.322788
H	-5.563262	-1.887460	0.908012
H	-11.436999	-2.205758	-5.176779
H	-13.698936	-1.161196	-5.395666

H	-14.656861	0.142224	-3.473669
H	-13.064130	-3.564176	5.044067
H	-10.691990	-2.903583	5.456635
H	-14.293074	-2.586870	3.074992
H	-6.659492	-1.249057	5.082698
H	-4.266524	-0.706707	5.094040
H	-4.741797	1.461817	1.407075
H	-7.114928	0.889366	1.381557
H	-2.520407	0.723687	5.517615
H	4.093511	0.365534	4.171754
H	1.630144	1.871721	3.013929
H	0.856722	2.066881	1.438246
H	1.404517	0.471101	1.974964
H	-0.715086	-1.761026	3.926468
H	-0.062401	-3.175274	4.770688
H	-0.257722	-1.624812	5.617292
H	4.312678	-3.723038	1.659409
H	6.455161	-4.265158	0.564997
H	8.205784	-0.738934	2.283161
H	6.125306	-0.208125	3.420074
H	10.614163	-5.522957	-1.412808
H	12.891075	-5.236655	-2.410195
H	14.120380	-3.069540	-2.113658

****

ΔΛ_open_conformer_2_gas_phase

Ga	12.251412	-0.334810	-0.385108
Ga	-12.409219	-0.166545	-0.033653
C	12.868025	2.105402	4.161977
C	11.592919	2.571081	3.914948
C	10.911441	2.213972	2.716595
C	11.562759	1.391609	1.762647
C	12.890105	0.872005	2.048854
C	13.515223	1.252761	3.235687
C	7.544014	2.513836	1.043056
C	7.106560	2.076205	-0.230131
C	5.800241	2.286846	-0.656463
C	4.861514	2.945028	0.164092
C	5.295341	3.343099	1.444576
C	6.596251	3.139782	1.883942
C	3.497473	3.248318	-0.281977
C	2.996056	3.545080	-1.526233

C	1.589670	3.873334	-1.535436
C	1.014215	3.813022	-0.273522
S	2.205742	3.372725	0.905304
C	0.915220	4.446303	-2.696806
C	1.697612	5.373104	-3.597893
C	0.620147	6.187239	-4.349199
C	-0.624661	5.277468	-4.289261
C	-0.371233	4.347862	-3.135452
C	-1.416936	3.383869	-2.774373
C	-2.745355	3.727363	-2.365426
C	-3.569298	2.646724	-2.138417
S	-2.691129	1.164398	-2.513438
C	-1.229104	2.024561	-2.917540
F	2.446536	4.668526	-4.507821
F	2.552951	6.198460	-2.939159
F	0.983899	6.506155	-5.616074
F	0.378924	7.344044	-3.675684
F	-1.764425	6.020416	-4.143275
F	-0.752622	4.600705	-5.473977
C	-0.387007	4.114602	0.165942
C	0.015450	1.329713	-3.383494
C	-4.942765	2.664787	-1.627352
C	-5.375783	3.777225	-0.877994
C	-6.679548	3.907624	-0.424998
C	-7.634836	2.899646	-0.692101
C	-7.199907	1.757001	-1.405554
C	-5.889957	1.645062	-1.859229
C	-10.985197	3.735295	0.826966
C	-11.651622	4.772643	1.540071
C	-12.923962	4.577355	2.036281
C	-13.584968	3.338744	1.855256
C	-12.976134	2.290946	1.165652
C	-11.647483	2.499686	0.611575
O	11.043335	1.040903	0.608091
O	13.431563	0.061741	1.162070
O	10.818741	-0.229610	-1.861727
O	13.224382	-1.994950	-0.906537
O	13.198272	0.870888	-1.657054
O	10.997871	-1.736803	0.474438
O	-13.535358	1.111718	0.985116
O	-11.139439	1.494147	-0.063103
O	-13.444192	-1.781296	0.524485
O	-10.998544	-0.975447	-1.309133

O	-11.190208	-0.832393	1.480019
O	-13.351967	0.110530	-1.763700
C	11.986302	2.523258	-4.709810
C	10.706083	2.011175	-4.781964
C	10.246063	1.079697	-3.809671
C	11.124497	0.662014	-2.779222
C	12.451855	1.240694	-2.678768
C	12.857005	2.146909	-3.658757
C	7.154071	-0.718455	-2.630108
C	7.010149	-1.658640	-1.584242
C	5.764656	-2.163200	-1.233236
C	4.602215	-1.754361	-1.911405
C	4.753326	-0.820176	-2.955329
C	5.992140	-0.310499	-3.320727
C	3.269792	-2.278166	-1.588280
C	2.834712	-3.544628	-1.294550
C	1.394239	-3.684327	-1.208716
C	0.739570	-2.471376	-1.409900
S	1.888797	-1.204363	-1.680433
C	0.763391	-5.002945	-1.118024
C	1.590183	-6.203753	-1.548584
C	0.575130	-7.353268	-1.736882
C	-0.615268	-6.917316	-0.868625
C	-0.473066	-5.430221	-0.726101
C	-1.606752	-4.712951	-0.114804
C	-2.822803	-4.415543	-0.807008
C	-3.721569	-3.667846	-0.081050
S	-3.092757	-3.431114	1.549551
C	-1.611568	-4.284420	1.191731
F	2.497355	-6.561674	-0.585800
F	2.290187	-6.008602	-2.698580
F	1.079865	-8.563238	-1.389345
F	0.193130	-7.414300	-3.040234
F	-1.800862	-7.296232	-1.430205
F	-0.547310	-7.550321	0.351728
C	-0.716889	-2.137925	-1.528596
C	-0.527763	-4.480168	2.208321
C	-4.963343	-3.065310	-0.569895
C	-5.085468	-2.783181	-1.945650
C	-6.256792	-2.289536	-2.501311
C	-7.380994	-2.042708	-1.681566
C	-7.247265	-2.261898	-0.290830
C	-6.068821	-2.759496	0.249503

C	-10.398812	-0.940725	-3.650386
C	-10.837868	-0.673211	-4.977586
C	-12.103839	-0.174224	-5.208945
C	-12.980970	0.096345	-4.131286
C	-12.596320	-0.145212	-2.812719
C	-11.283413	-0.714534	-2.566427
C	-12.817319	-4.198934	3.225674
C	-11.602193	-3.741653	3.697527
C	-10.988356	-2.597347	3.117207
C	-11.649364	-1.919157	2.061968
C	-12.908293	-2.422122	1.544380
C	-13.468384	-3.547950	2.149698
C	-7.763828	-0.685616	2.941784
C	-6.747686	-1.180286	3.786607
C	-5.439274	-0.726069	3.635399
C	-5.094430	0.222975	2.659351
C	-6.117200	0.735620	1.841721
C	-7.426187	0.303922	1.989449
C	-3.684337	0.585514	2.444351
C	-2.772498	1.152040	3.292487
C	-1.438460	1.232760	2.751157
C	-1.355063	0.723894	1.466009
S	-2.912337	0.179492	0.924055
C	-0.340353	1.846558	3.495964
C	-0.649114	3.101457	4.270980
C	0.725201	3.727521	4.576812
C	1.689551	2.524103	4.499896
C	0.970017	1.494150	3.658644
C	1.669450	0.258166	3.301207
C	3.054099	0.206740	2.898520
C	3.550597	-1.063868	2.725146
S	2.269931	-2.230419	3.052423
C	1.102815	-0.999270	3.437525
F	-1.290724	2.827927	5.459095
F	-1.444955	3.976821	3.595862
F	0.765662	4.356972	5.777645
F	1.029648	4.637430	3.614967
F	2.902214	2.916288	4.012637
F	1.918199	2.053170	5.767679
C	-0.152934	0.551552	0.596400
C	-0.263156	-1.394651	3.912118
C	4.900334	-1.521922	2.389823
C	5.245819	-2.886079	2.444414

C	6.527445	-3.345566	2.170620
C	7.544293	-2.432642	1.816591
C	7.211998	-1.057832	1.772781
C	5.926197	-0.616438	2.042822
C	10.707340	-4.124372	0.688203
C	11.269646	-5.408461	0.441623
C	12.470598	-5.529302	-0.228491
C	13.158121	-4.382025	-0.693817
C	12.649889	-3.100824	-0.478394
C	11.405999	-2.967208	0.259997
N	8.424715	-0.228905	-2.892101
N	8.862268	2.281230	1.408431
N	8.834177	-2.797887	1.467431
C	9.373739	-4.077349	1.331200
C	9.522910	2.701609	2.565802
C	8.835308	0.636969	-3.904720
O	8.972338	3.421707	3.413627
O	8.067840	1.019450	-4.801643
O	8.763685	-5.095211	1.695265
H	9.468998	1.738883	0.770251
H	9.191919	-0.491647	-2.248911
H	9.485345	-2.052480	1.163775
C	-9.639768	-2.216036	3.606982
N	-9.077635	-1.129205	2.945809
N	-8.599387	-1.583189	-2.156659
C	-9.008949	-1.420702	-3.481518
N	-8.952769	2.957023	-0.262971
C	-9.601221	4.012439	0.385062
O	-9.042444	5.102521	0.586825
O	-8.253753	-1.645937	-4.439788
O	-9.045181	-2.829737	4.507646
H	-9.358594	-1.406751	-1.475257
H	-9.716957	-0.714096	2.245218
H	-9.564295	2.136675	-0.411714
H	13.381786	2.382760	5.084556
H	11.066643	3.205273	4.621698
H	14.513162	0.863024	3.432937
H	7.803716	1.558327	-0.884001
H	5.499373	1.906847	-1.628516
H	4.603262	3.849594	2.113762
H	6.911941	3.469524	2.862483
H	3.613675	3.574824	-2.416353
H	-3.072700	4.752049	-2.243659

H	-0.798501	4.952262	-0.408230
H	-1.054903	3.261256	0.000417
H	-0.429742	4.363459	1.230390
H	0.759879	1.263153	-2.582573
H	-0.185626	0.315052	-3.738690
H	0.478738	1.892973	-4.202051
H	-4.660470	4.555423	-0.623994
H	-6.987766	4.771479	0.144759
H	-7.903564	0.953526	-1.607984
H	-5.610762	0.750113	-2.410774
H	-11.116495	5.706106	1.684619
H	-13.425745	5.377636	2.583863
H	-14.582706	3.170006	2.258625
H	12.330564	3.238688	-5.459063
H	10.011041	2.309277	-5.561091
H	13.858725	2.568100	-3.582039
H	7.891125	-1.972360	-1.030499
H	5.687377	-2.850183	-0.396492
H	3.877028	-0.499975	-3.515063
H	6.092845	0.393817	-4.134137
H	3.519586	-4.376938	-1.200050
H	-3.001226	-4.715072	-1.832652
H	-0.859358	-1.156806	-1.986974
H	-1.236430	-2.873669	-2.147434
H	-1.224224	-2.119785	-0.559375
H	-0.037059	-5.446122	2.047753
H	-0.919376	-4.458385	3.230374
H	0.240243	-3.704405	2.125571
H	-4.228858	-2.938986	-2.596648
H	-6.335026	-2.090683	-3.560257
H	-8.088362	-2.048333	0.363107
H	-6.017172	-2.919537	1.323869
H	-10.138698	-0.863183	-5.786190
H	-12.431374	0.030692	-6.229901
H	-13.971603	0.514648	-4.305192
H	-13.280347	-5.079450	3.675617
H	-11.073158	-4.242016	4.503396
H	-14.417535	-3.917448	1.763098
H	-7.004872	-1.933334	4.518552
H	-4.653566	-1.140310	4.263973
H	-5.880927	1.472943	1.081009
H	-8.204868	0.708912	1.347901
H	-3.032663	1.501269	4.284454

H	3.659121	1.091308	2.754734
H	0.651008	1.222773	0.893067
H	-0.387506	0.743414	-0.454296
H	0.243731	-0.467319	0.676762
H	-0.977390	-1.495718	3.084499
H	-0.232666	-2.349384	4.447857
H	-0.668927	-0.635952	4.586999
H	4.492165	-3.623751	2.713275
H	6.768039	-4.398185	2.211803
H	7.978190	-0.336955	1.500979
H	5.718282	0.445938	1.965473
H	10.712298	-6.275641	0.783007
H	12.893381	-6.518301	-0.416025
H	14.095673	-4.473740	-1.240961

****

ΔΛ_open_conformer_3_gas_phase

Ga	12.197448	-0.137985	-0.115698
Ga	-12.433781	-0.023724	0.203509
C	12.638746	2.029987	4.592151
C	11.361512	2.487136	4.339089
C	10.714278	2.182281	3.108133
C	11.401770	1.418989	2.129652
C	12.733707	0.912648	2.417144
C	13.323552	1.240213	3.637629
C	7.387184	2.491311	1.361982
C	6.986746	2.005005	0.094710
C	5.689383	2.173965	-0.365953
C	4.719190	2.852800	0.403000
C	5.119962	3.312146	1.672735
C	6.412621	3.139324	2.153686
C	3.375185	3.113127	-0.119951
C	2.913690	3.071410	-1.414275
C	1.528072	3.437694	-1.564843
C	0.923033	3.762292	-0.362602
S	2.062488	3.631221	0.942482
C	0.927470	3.697574	-2.873176
C	1.795716	4.395756	-3.902359
C	0.797655	4.992856	-4.916734
C	-0.443321	4.092732	-4.759391
C	-0.306777	3.470115	-3.393323
C	-1.435869	2.621443	-2.954015

C	-2.651713	3.109716	-2.379623
C	-3.645825	2.162809	-2.251740
S	-3.066615	0.628166	-2.892001
C	-1.507242	1.292389	-3.319717
F	2.607016	3.496999	-4.545879
F	2.597683	5.362316	-3.387509
F	1.279027	5.022443	-6.183232
F	0.491394	6.266587	-4.549790
F	-1.594270	4.814285	-4.895196
F	-0.453921	3.153432	-5.759255
C	-0.482334	4.201225	-0.086180
C	-0.518603	0.513499	-4.139936
C	-4.987395	2.329048	-1.689843
C	-5.300777	3.484149	-0.944540
C	-6.575529	3.730944	-0.455282
C	-7.617923	2.801570	-0.677479
C	-7.306400	1.623193	-1.395597
C	-6.028284	1.396775	-1.887020
C	-10.832152	3.818700	0.999513
C	-11.413816	4.872490	1.759891
C	-12.662881	4.720754	2.326700
C	-13.381748	3.510642	2.174544
C	-12.855682	2.447232	1.441362
C	-11.557427	2.614415	0.809580
O	10.914475	1.114764	0.947669
O	13.312745	0.164280	1.500009
O	10.811860	0.057758	-1.633645
O	13.237427	-1.721630	-0.748726
O	13.158220	1.185230	-1.249617
O	10.965742	-1.644845	0.571343
O	-13.464963	1.289599	1.284836
O	-11.133371	1.598384	0.092300
O	-13.473506	-1.606501	0.829486
O	-11.127918	-0.879580	-1.157279
O	-11.131111	-0.728093	1.634401
O	-13.478835	0.262410	-1.465989
C	12.086468	2.955723	-4.287684
C	10.822567	2.425926	-4.455456
C	10.325922	1.443722	-3.553693
C	11.151930	0.994094	-2.493029
C	12.461083	1.588605	-2.293355
C	12.903367	2.545579	-3.206743
C	7.193328	-0.419603	-2.610648

C	7.004124	-1.383269	-1.593732
C	5.743458	-1.887719	-1.303693
C	4.611040	-1.455391	-2.017324
C	4.804444	-0.497944	-3.031415
C	6.061317	0.011522	-3.337354
C	3.267771	-1.980539	-1.741074
C	2.831082	-3.263196	-1.539591
C	1.393598	-3.402305	-1.416441
C	0.747968	-2.168793	-1.483553
S	1.896972	-0.890950	-1.714622
C	0.758601	-4.723210	-1.415729
C	1.592545	-5.891010	-1.922260
C	0.586876	-7.032886	-2.188895
C	-0.612535	-6.658387	-1.306871
C	-0.481858	-5.182039	-1.070596
C	-1.636063	-4.522289	-0.433290
C	-2.852710	-4.218524	-1.122040
C	-3.782916	-3.544267	-0.362539
S	-3.170374	-3.372485	1.282776
C	-1.663895	-4.165339	0.894180
F	2.501926	-6.304803	-0.984929
F	2.291189	-5.620102	-3.058516
F	1.097852	-8.258393	-1.911699
F	0.215595	-7.017396	-3.496787
F	-1.790488	-7.010971	-1.901555
F	-0.551063	-7.364639	-0.127017
C	-0.703925	-1.806076	-1.442550
C	-0.574692	-4.360168	1.903727
C	-5.068009	-2.995077	-0.800053
C	-5.302568	-2.792969	-2.175530
C	-6.522370	-2.346686	-2.664224
C	-7.583031	-2.063588	-1.774028
C	-7.340095	-2.210427	-0.389187
C	-6.116062	-2.663529	0.082997
C	-10.728947	-0.975765	-3.539644
C	-11.269425	-0.749719	-4.836833
C	-12.532783	-0.213893	-4.985426
C	-13.306824	0.134830	-3.852518
C	-12.821280	-0.063895	-2.560204
C	-11.510252	-0.667362	-2.397359
C	-12.738269	-4.058447	3.470353
C	-11.481790	-3.639130	3.861948
C	-10.873917	-2.509946	3.246652

C	-11.582205	-1.805155	2.240588
C	-12.887630	-2.268601	1.807699
C	-13.439450	-3.381527	2.443314
C	-7.607351	-0.677744	2.919661
C	-6.580072	-1.169016	3.753333
C	-5.276379	-0.704771	3.593451
C	-4.942728	0.247037	2.615128
C	-5.972952	0.742322	1.796775
C	-7.278391	0.302927	1.956120
C	-3.538693	0.647532	2.430177
C	-2.659568	1.181620	3.334165
C	-1.310150	1.300982	2.842076
C	-1.179425	0.851911	1.539657
S	-2.714721	0.328377	0.914380
C	-0.215256	1.855675	3.645382
C	-0.392505	3.215818	4.271213
C	0.798341	3.372309	5.238186
C	1.846410	2.378828	4.687390
C	1.048257	1.394630	3.866379
C	1.695194	0.143531	3.474166
C	3.081030	0.089573	3.087368
C	3.546995	-1.176136	2.821803
S	2.236454	-2.332490	3.060940
C	1.091414	-1.099522	3.512884
F	-1.573457	3.381705	4.935884
F	-0.339712	4.211211	3.324683
F	0.418664	2.984475	6.486668
F	1.257788	4.643177	5.313165
F	2.775563	3.067430	3.951223
F	2.526896	1.782212	5.705110
C	0.055924	0.698539	0.715358
C	-0.303309	-1.472035	3.916699
C	4.885483	-1.626175	2.439151
C	5.205785	-2.992117	2.319880
C	6.481980	-3.434181	1.992180
C	7.513722	-2.499190	1.759835
C	7.203264	-1.124332	1.882940
C	5.925595	-0.700840	2.206560
C	10.703863	-4.045808	0.511740
C	11.293350	-5.287675	0.144129
C	12.518207	-5.318997	-0.492613
C	13.203316	-4.119390	-0.804038
C	12.668050	-2.875609	-0.466919

C	11.398858	-2.840465	0.237536
N	8.476539	0.070750	-2.802107
N	8.699493	2.279303	1.756513
N	8.803175	-2.829233	1.381247
C	9.350682	-4.082754	1.114768
C	9.322034	2.659148	2.946626
C	8.929175	0.988150	-3.748377
O	8.737888	3.331753	3.810692
O	8.206044	1.402593	-4.667558
O	8.737021	-5.137227	1.342768
H	9.331102	1.769026	1.115466
H	9.211416	-0.213797	-2.131538
H	9.458756	-2.053355	1.177367
C	-9.488182	-2.169776	3.656070
N	-8.924844	-1.111181	2.951992
N	-8.837109	-1.627907	-2.171773
C	-9.345365	-1.497120	-3.464660
N	-8.909662	2.954157	-0.202129
C	-9.460089	4.035064	0.488823
O	-8.831676	5.089740	0.671523
O	-8.676148	-1.781044	-4.470373
O	-8.866806	-2.794568	4.530659
H	-9.533621	-1.402011	-1.439743
H	-9.586362	-0.674450	2.286685
H	-9.574562	2.169723	-0.319236
H	13.125811	2.266639	5.540207
H	10.808155	3.075525	5.064649
H	14.324760	0.860174	3.837292
H	7.708174	1.480546	-0.525967
H	5.426702	1.753568	-1.331314
H	4.410559	3.838663	2.305667
H	6.698985	3.506171	3.128433
H	3.545754	2.814214	-2.255685
H	-2.784221	4.144228	-2.090170
H	-0.559096	4.719301	0.873441
H	-0.836920	4.869095	-0.879579
H	-1.168957	3.347310	-0.051835
H	0.489869	0.912584	-3.997622
H	-0.496031	-0.546002	-3.872566
H	-0.756675	0.586515	-5.208686
H	-4.518280	4.205769	-0.723939
H	-6.793467	4.623809	0.112414
H	-8.082711	0.881957	-1.566679

H	-5.847261	0.474678	-2.434047
H	-10.833217	5.781514	1.884454
H	-13.100201	5.533411	2.910105
H	-14.359206	3.376437	2.636312
H	12.458680	3.710247	-4.983372
H	10.166610	2.747802	-5.258660
H	13.891518	2.978090	-3.054325
H	7.861075	-1.715733	-1.013780
H	5.629195	-2.594939	-0.488228
H	3.948826	-0.156771	-3.611234
H	6.197506	0.737901	-4.125916
H	3.516084	-4.100196	-1.525074
H	-3.010575	-4.472017	-2.163090
H	-1.146167	-1.994198	-0.461538
H	-0.850643	-0.749010	-1.658490
H	-1.286013	-2.381723	-2.168137
H	-0.024874	-5.281687	1.684796
H	-0.969299	-4.427808	2.922728
H	0.145708	-3.536097	1.876371
H	-4.499819	-2.975311	-2.885296
H	-6.685108	-2.208084	-3.723308
H	-8.130972	-1.972050	0.316887
H	-5.983725	-2.762507	1.157986
H	-10.647865	-1.000941	-5.690920
H	-12.938394	-0.041452	-5.984193
H	-14.294919	0.579839	-3.963216
H	-13.196006	-4.927949	3.946350
H	-10.915815	-4.158486	4.629717
H	-14.422826	-3.719877	2.118729
H	-6.826775	-1.920720	4.490037
H	-4.484858	-1.109565	4.220870
H	-5.748072	1.479387	1.033222
H	-8.063041	0.702857	1.319793
H	-2.957897	1.469782	4.334050
H	3.700286	0.974271	3.006202
H	0.379535	-0.347576	0.702663
H	0.880961	1.274505	1.128112
H	-0.095794	1.022703	-0.318527
H	-0.328146	-2.453733	4.402125
H	-0.708196	-0.733320	4.614055
H	-0.987705	-1.503494	3.058991
H	4.436189	-3.743700	2.486125
H	6.706854	-4.487276	1.899177

H	7.982306	-0.388141	1.704085
H	5.734845	0.365630	2.263606
H	10.738306	-6.193848	0.368057
H	12.962355	-6.276030	-0.773500
H	14.159889	-4.139310	-1.324952

****

ΔΛ_open_conformer_4_gas_phase

Ga	12.210640	-0.126056	-0.090465
Ga	-12.416362	0.222745	0.155227
C	12.640398	2.161266	4.564864
C	11.412111	2.702885	4.241382
C	10.795197	2.397182	2.996056
C	11.466354	1.547726	2.080037
C	12.739449	0.949442	2.440041
C	13.302007	1.282721	3.672767
C	7.567713	2.714237	1.096133
C	7.227867	2.127073	-0.145446
C	5.940241	2.201023	-0.648474
C	4.918948	2.877516	0.052679
C	5.268569	3.463894	1.284174
C	6.555035	3.395314	1.805563
C	3.569586	2.988793	-0.501844
C	3.077339	2.583001	-1.719463
C	1.688634	2.897064	-1.933497
C	1.109304	3.560136	-0.867724
S	2.280715	3.784229	0.402584
C	1.014661	2.606382	-3.198019
C	1.788862	2.812948	-4.477643
C	0.719457	2.786664	-5.593389
C	-0.463918	2.040385	-4.944571
C	-0.256165	2.188048	-3.458609
C	-1.335175	1.780579	-2.552155
C	-2.685660	2.269514	-2.673058
C	-3.548940	1.763799	-1.737901
S	-2.708914	0.616190	-0.710233
C	-1.187438	0.885167	-1.507398
F	2.711660	1.827294	-4.714998
F	2.472010	3.991104	-4.492308
F	1.154734	2.201368	-6.733452
F	0.341166	4.060603	-5.888681
F	-1.650697	2.538048	-5.400651

F	-0.428491	0.719832	-5.324493
C	-0.280053	4.104099	-0.722964
C	0.055879	0.249462	-0.979064
C	-4.946419	2.131193	-1.459445
C	-5.270697	3.460536	-1.139760
C	-6.566296	3.838849	-0.794911
C	-7.594967	2.873140	-0.764127
C	-7.276239	1.541788	-1.116626
C	-5.978302	1.176553	-1.439993
C	-10.825237	4.099181	0.717792
C	-11.413441	5.205421	1.391164
C	-12.657372	5.086426	1.979704
C	-13.364973	3.860901	1.934351
C	-12.832439	2.745706	1.286395
C	-11.540494	2.877415	0.638391
O	11.006934	1.235113	0.888500
O	13.286171	0.120280	1.574675
O	10.896717	0.168356	-1.681635
O	13.149346	-1.778675	-0.679993
O	13.311951	1.108840	-1.189735
O	10.828777	-1.526956	0.534709
O	-13.425084	1.569394	1.226124
O	-11.107659	1.805926	0.009433
O	-13.455960	-1.356688	0.776159
O	-11.151777	-0.700817	-1.215995
O	-11.083052	-0.524848	1.553058
O	-13.483227	0.497317	-1.501533
C	12.584124	2.840841	-4.346835
C	11.298740	2.399994	-4.587321
C	10.663966	1.495467	-3.689583
C	11.371973	1.031780	-2.550995
C	12.712948	1.526690	-2.286588
C	13.289973	2.411011	-3.197550
C	7.340467	-0.148067	-3.006501
C	6.957184	-0.993223	-1.938027
C	5.659084	-1.468384	-1.823950
C	4.671053	-1.142571	-2.778419
C	5.055253	-0.282003	-3.825096
C	6.348568	0.212927	-3.945763
C	3.324702	-1.717101	-2.710240
C	2.871692	-2.810600	-2.011027
C	1.479141	-3.116751	-2.220459
C	0.859937	-2.238224	-3.093208

S	1.994673	-1.052683	-3.664057
C	0.881419	-4.374175	-1.771351
C	1.742471	-5.618952	-1.870890
C	0.739531	-6.791612	-1.853194
C	-0.483795	-6.195693	-1.130013
C	-0.344694	-4.702907	-1.287269
C	-1.460158	-3.890388	-0.754864
C	-2.688785	-3.637162	-1.442604
C	-3.665664	-3.044350	-0.671008
S	-3.053241	-2.822149	0.964892
C	-1.503987	-3.533394	0.577746
F	2.575142	-5.723556	-0.786483
F	2.522212	-5.666818	-2.980880
F	1.228761	-7.901895	-1.249483
F	0.404971	-7.116542	-3.131253
F	-1.648276	-6.673705	-1.658364
F	-0.467200	-6.582611	0.186189
C	-0.556642	-2.211687	-3.579715
C	-0.496062	-3.852087	1.645138
C	-5.013683	-2.638121	-1.072003
C	-5.353304	-2.574641	-2.438959
C	-6.635419	-2.270512	-2.873745
C	-7.658457	-1.989630	-1.938780
C	-7.320491	-2.018429	-0.565346
C	-6.035537	-2.334964	-0.147204
C	-10.902583	-1.039030	-3.596547
C	-11.511025	-0.911525	-4.877343
C	-12.760371	-0.339532	-5.005744
C	-13.452751	0.143513	-3.869258
C	-12.899740	0.044781	-2.592679
C	-11.601460	-0.591864	-2.445901
C	-12.474099	-4.158935	2.942219
C	-11.200759	-3.765831	3.301859
C	-10.665041	-2.534322	2.830368
C	-11.460929	-1.700562	2.004609
C	-12.783428	-2.139706	1.595185
C	-13.263652	-3.353451	2.086627
C	-7.511405	-0.472478	2.811949
C	-6.437636	-1.102820	3.480758
C	-5.212919	-0.459880	3.593404
C	-4.985914	0.829864	3.071093
C	-6.048174	1.429802	2.364245
C	-7.277294	0.797683	2.237328

C	-3.693367	1.480186	3.296192
C	-2.743439	1.161856	4.241190
C	-1.526189	1.905118	4.128586
C	-1.572003	2.870699	3.150933
S	-3.097372	2.810711	2.303246
C	-0.353914	1.685230	4.995465
C	-0.450380	2.236134	6.388165
C	0.760838	1.659203	7.134340
C	1.739560	1.298605	5.994825
C	0.877711	1.145690	4.749909
C	1.483918	0.468447	3.600151
C	2.918637	0.281825	3.513061
C	3.328801	-0.545794	2.501493
S	1.935010	-1.066554	1.577813
C	0.812466	-0.211077	2.585123
F	-1.617659	1.913929	7.019875
F	-0.377881	3.610779	6.392157
F	0.394960	0.529191	7.796055
F	1.297964	2.518678	8.035788
F	2.654146	2.308637	5.853741
F	2.436394	0.177321	6.326135
C	-0.488152	3.838610	2.787206
C	-0.646997	-0.348990	2.281318
C	4.662251	-1.057602	2.160541
C	4.841462	-2.417747	1.843714
C	6.089243	-2.946466	1.533212
C	7.225931	-2.107988	1.514309
C	7.050988	-0.742245	1.834902
C	5.799392	-0.229703	2.149067
C	10.324137	-3.881101	0.317820
C	10.804924	-5.158263	-0.085045
C	12.056603	-5.288398	-0.653045
C	12.876279	-4.153179	-0.861332
C	12.449267	-2.879049	-0.487252
C	11.153729	-2.743464	0.153398
N	8.654723	0.291858	-3.049937
N	8.865437	2.545596	1.546066
N	8.499856	-2.528414	1.162014
C	8.938378	-3.810995	0.838459
C	9.440240	2.945110	2.751075
C	9.262886	1.123990	-3.991075
O	8.847668	3.678371	3.558334
O	8.660390	1.534156	-4.995573

O	8.211737	-4.809408	0.959795
H	9.505782	1.973799	0.966106
H	9.300030	-0.007411	-2.298759
H	9.236836	-1.810224	1.053746
C	-9.271536	-2.212130	3.212638
N	-8.770760	-1.031455	2.662932
N	-8.955542	-1.649540	-2.284325
C	-9.531875	-1.595565	-3.554481
N	-8.903811	3.126550	-0.379951
C	-9.450513	4.273411	0.185372
O	-8.823281	5.341936	0.264091
O	-8.926112	-1.972779	-4.570043
O	-8.588814	-2.940090	3.950130
H	-9.603669	-1.351294	-1.534539
H	-9.477375	-0.510798	2.113649
H	-9.568788	2.333960	-0.402158
H	13.105656	2.398999	5.523562
H	10.874955	3.357091	4.921718
H	14.261321	0.837104	3.933387
H	7.992020	1.594940	-0.705573
H	5.726749	1.711606	-1.592881
H	4.514670	3.994303	1.863059
H	6.802577	3.846545	2.755997
H	3.679426	2.063839	-2.454712
H	-2.996089	2.986350	-3.422418
H	-0.958772	3.384277	-0.247239
H	-0.288151	5.020392	-0.122694
H	-0.703621	4.332572	-1.704938
H	-0.094330	-0.805515	-0.731357
H	0.869020	0.310553	-1.698634
H	0.396177	0.768569	-0.076827
H	-4.477449	4.205340	-1.133154
H	-6.805026	4.857717	-0.523660
H	-8.062399	0.791861	-1.114618
H	-5.760438	0.142092	-1.683451
H	-10.842531	6.128264	1.435371
H	-13.099893	5.940077	2.496991
H	-14.338300	3.757429	2.412677
H	13.061593	3.536579	-5.039670
H	10.729649	2.732925	-5.449960
H	14.298049	2.768577	-2.991036
H	7.692820	-1.267463	-1.186763
H	5.410000	-2.086808	-0.967659

H	4.331557	0.001440	-4.584713
H	6.622106	0.866844	-4.760794
H	3.514176	-3.411189	-1.378485
H	-2.842651	-3.910041	-2.478677
H	-0.919295	-3.230834	-3.756482
H	-1.224943	-1.754777	-2.840603
H	-0.652167	-1.636294	-4.504272
H	-0.732414	-4.807507	2.130496
H	-0.450993	-3.083566	2.420970
H	0.502885	-3.941063	1.208493
H	-4.585793	-2.750646	-3.188379
H	-6.873712	-2.228574	-3.926598
H	-8.081558	-1.789493	0.176126
H	-5.833754	-2.344870	0.921151
H	-10.950342	-1.265546	-5.737145
H	-13.218396	-0.243900	-5.992200
H	-14.430081	0.614890	-3.965688
H	-12.876000	-5.106376	3.306390
H	-10.567832	-4.379081	3.936089
H	-14.260223	-3.669157	1.780365
H	-6.594330	-2.087421	3.897175
H	-4.400374	-0.984217	4.089673
H	-5.922912	2.408570	1.906894
H	-8.078917	1.290573	1.693893
H	-2.887917	0.392063	4.989240
H	3.621459	0.714762	4.212055
H	-0.890238	4.750432	2.333904
H	0.220382	3.395861	2.079556
H	0.076565	4.118734	3.682773
H	-0.815801	-1.070868	1.483789
H	-1.213953	-0.677041	3.157392
H	-1.088467	0.595092	1.953782
H	3.982220	-3.085652	1.860235
H	6.214717	-3.994889	1.302822
H	7.911358	-0.078604	1.810599
H	5.695239	0.832188	2.348631
H	10.146632	-6.010181	0.056063
H	12.416615	-6.272007	-0.960840
H	13.854463	-4.245817	-1.331771

****

ΔΛ_open_conformer_5_gas_phase

Ga	12.198718	-0.088579	0.086279
Ga	-12.356671	0.179123	0.269640
C	12.659790	2.406915	4.631246
C	11.437085	2.946443	4.283492
C	10.815424	2.588181	3.054814
C	11.475523	1.686202	2.181976
C	12.743817	1.093390	2.566625
C	13.311276	1.479727	3.781628
C	7.607052	2.855125	1.118769
C	7.272748	2.202911	-0.092112
C	5.991323	2.263783	-0.610768
C	4.971277	2.990312	0.042088
C	5.315961	3.640437	1.242284
C	6.596753	3.586541	1.779426
C	3.629548	3.083653	-0.532454
C	3.145173	2.588380	-1.720096
C	1.765008	2.902167	-1.973129
C	1.181786	3.655102	-0.971470
S	2.341774	3.967702	0.290572
C	1.104334	2.551962	-3.227812
C	1.892973	2.704873	-4.505571
C	0.829808	2.672705	-5.625510
C	-0.369763	1.959150	-4.967791
C	-0.166294	2.130260	-3.482056
C	-1.250328	1.753814	-2.566459
C	-2.604130	2.224880	-2.731933
C	-3.481278	1.752269	-1.791717
S	-2.648407	0.666840	-0.698733
C	-1.114705	0.905774	-1.478713
F	2.790981	1.688233	-4.702784
F	2.605071	3.864103	-4.550080
F	1.259180	2.057382	-6.751395
F	0.474249	3.946787	-5.947341
F	-1.544293	2.472808	-5.438849
F	-0.357000	0.634648	-5.331306
C	-0.200782	4.230209	-0.907353
C	0.104452	0.272730	-0.886989
C	-4.889011	2.098548	-1.546149
C	-5.255983	3.439902	-1.345152
C	-6.557020	3.804382	-1.010314
C	-7.551958	2.812849	-0.865622

C	-7.192126	1.464870	-1.100297
C	-5.887955	1.115481	-1.418432
C	-10.790137	4.096575	0.546744
C	-11.386335	5.246031	1.136385
C	-12.622564	5.158442	1.745586
C	-13.314960	3.924983	1.803120
C	-12.774906	2.768388	1.238956
C	-11.488673	2.863076	0.572520
O	11.008695	1.318648	1.009330
O	13.280486	0.219560	1.739646
O	10.860109	0.141948	-1.508094
O	13.129681	-1.765611	-0.450942
O	13.281774	1.106663	-1.061472
O	10.821674	-1.454142	0.770204
O	-13.355145	1.586170	1.274414
O	-11.045727	1.750579	0.026513
O	-13.410939	-1.358897	0.974583
O	-11.105782	-0.810753	-1.085307
O	-11.053026	-0.471640	1.725143
O	-13.456294	0.369743	-1.366792
C	12.500271	2.791065	-4.229375
C	11.217182	2.334879	-4.449682
C	10.599584	1.435891	-3.533722
C	11.320838	0.994650	-2.394685
C	12.661798	1.504795	-2.154749
C	13.221573	2.381699	-3.082529
C	7.302066	-0.294288	-2.898225
C	6.914842	-1.200818	-1.882356
C	5.634102	-1.740549	-1.844830
C	4.670508	-1.410447	-2.821176
C	5.054426	-0.481900	-3.808117
C	6.327433	0.070992	-3.854961
C	3.333393	-2.014942	-2.842047
C	2.901745	-3.248011	-2.412630
C	1.498680	-3.499156	-2.640850
C	0.854152	-2.427352	-3.245528
S	1.978181	-1.144653	-3.558601
C	0.855408	-4.807500	-2.496528
C	1.568967	-6.028245	-3.028895
C	0.626511	-7.215361	-2.716200
C	-0.745585	-6.543895	-2.496189
C	-0.414335	-5.129388	-2.115743
C	-1.418864	-4.321922	-1.425665

C	-2.778607	-4.191773	-1.857386
C	-3.551090	-3.373268	-1.064386
S	-2.584384	-2.814794	0.301300
C	-1.155609	-3.646968	-0.252094
F	2.793322	-6.233455	-2.460321
F	1.770306	-5.953629	-4.379717
F	1.015052	-7.826431	-1.566586
F	0.609794	-8.146198	-3.703641
F	-1.470946	-6.595340	-3.666914
F	-1.475691	-7.212373	-1.562510
C	-0.575404	-2.279327	-3.671588
C	0.140051	-3.608496	0.497829
C	-4.928072	-2.936255	-1.312973
C	-5.405100	-2.930835	-2.639026
C	-6.708463	-2.589486	-2.959668
C	-7.623206	-2.210933	-1.948697
C	-7.142882	-2.169822	-0.616903
C	-5.831822	-2.524582	-0.311226
C	-10.977932	-1.347674	-3.439784
C	-11.648294	-1.324250	-4.697182
C	-12.898957	-0.755584	-4.815385
C	-13.533311	-0.175880	-3.691051
C	-12.920114	-0.173822	-2.439005
C	-11.615011	-0.801162	-2.296189
C	-12.647445	-3.761913	3.648470
C	-11.394754	-3.325145	4.029922
C	-10.795567	-2.204013	3.389469
C	-11.502906	-1.530493	2.362812
C	-12.812503	-2.004955	1.953183
C	-13.355922	-3.108260	2.611783
C	-7.624815	-0.175118	3.281226
C	-6.658654	-0.620832	4.211265
C	-5.427411	0.009855	4.293162
C	-5.083126	1.108134	3.479060
C	-6.048180	1.538763	2.544412
C	-7.286290	0.918018	2.448744
C	-3.748367	1.696568	3.601802
C	-2.824078	1.503357	4.602656
C	-1.526111	2.045515	4.322817
C	-1.475341	2.731939	3.128936
S	-3.027380	2.688011	2.332113
C	-0.386324	1.855115	5.219549
C	-0.606138	2.135731	6.679557

C	0.806015	2.288141	7.270340
C	1.710038	1.541985	6.258972
C	0.886959	1.402061	4.992169
C	1.495029	0.706588	3.853432
C	2.931665	0.590799	3.693036
C	3.330494	-0.297928	2.727917
S	1.916633	-0.955735	1.932542
C	0.804193	-0.103779	2.952022
F	-1.246714	1.087140	7.306711
F	-1.362545	3.241927	6.921949
F	0.913944	1.790183	8.527979
F	1.136279	3.605699	7.311244
F	2.865039	2.255012	6.083830
F	2.072199	0.330516	6.774186
C	-0.287335	3.366674	2.484047
C	-0.653748	-0.426653	2.812666
C	4.654384	-0.815201	2.363615
C	4.811572	-2.184034	2.068409
C	6.043218	-2.737288	1.746497
C	7.192391	-1.918673	1.697798
C	7.042846	-0.543827	1.993358
C	5.804041	-0.005131	2.316489
C	10.249764	-3.790054	0.546726
C	10.697038	-5.081558	0.151179
C	11.947863	-5.247217	-0.409636
C	12.798681	-4.135331	-0.620084
C	12.404424	-2.848615	-0.253136
C	11.113179	-2.678290	0.386858
N	8.600801	0.198995	-2.892020
N	8.897855	2.696009	1.588329
N	8.453573	-2.373880	1.346320
C	8.862413	-3.674584	1.053168
C	9.468304	3.141268	2.779169
C	9.205362	1.039774	-3.831311
O	8.879417	3.916392	3.549153
O	8.603098	1.435709	-4.841560
O	8.109336	-4.652017	1.183872
H	9.535225	2.088099	1.041759
H	9.242726	-0.091556	-2.135991
H	9.211453	-1.677576	1.235494
C	-9.437197	-1.816020	3.831093
N	-8.872445	-0.753648	3.124819
N	-8.937935	-1.852979	-2.205826

C	-9.614879	-1.915926	-3.429820
N	-8.860352	3.064480	-0.483382
C	-9.426189	4.247453	-0.015264
O	-8.819345	5.330176	-0.039987
O	-9.090090	-2.403005	-4.443928
O	-8.837359	-2.396333	4.750159
H	-9.524116	-1.486688	-1.437469
H	-9.513937	-0.353010	2.417951
H	-9.510374	2.260608	-0.431151
H	13.129139	2.685281	5.576914
H	10.908760	3.639337	4.931762
H	14.266720	1.038131	4.062563
H	8.035836	1.631186	-0.613984
H	5.782203	1.725516	-1.529701
H	4.562120	4.209729	1.783281
H	6.841582	4.086456	2.706068
H	3.747271	2.007224	-2.407869
H	-2.906830	2.901838	-3.520035
H	-0.218844	5.166923	-0.339440
H	-0.573593	4.433174	-1.916141
H	-0.915142	3.538521	-0.443891
H	-0.068737	-0.781788	-0.647479
H	0.955573	0.330015	-1.562428
H	0.395981	0.780750	0.040240
H	-4.489504	4.208612	-1.420747
H	-6.826560	4.835342	-0.829589
H	-7.952194	0.692213	-1.018372
H	-5.641591	0.069101	-1.573732
H	-10.827535	6.176532	1.100904
H	-13.070998	6.044626	2.199109
H	-14.283025	3.847772	2.296663
H	12.963687	3.482054	-4.936253
H	10.637074	2.649502	-5.311589
H	14.228923	2.749337	-2.890844
H	7.631111	-1.476125	-1.112726
H	5.373474	-2.406099	-1.026747
H	4.347607	-0.197697	-4.582958
H	6.602034	0.768744	-4.631722
H	3.569559	-3.977459	-1.972585
H	-3.156774	-4.681679	-2.746561
H	-0.681651	-1.527406	-4.456248
H	-0.971552	-3.232924	-4.037068
H	-1.212296	-1.970831	-2.833706

H	0.816256	-2.847789	0.092942
H	0.658653	-4.569670	0.407077
H	-0.002428	-3.398556	1.561340
H	-4.722485	-3.179653	-3.447543
H	-7.049051	-2.597982	-3.983859
H	-7.813266	-1.862121	0.182561
H	-5.514749	-2.491117	0.728818
H	-11.131504	-1.754895	-5.549062
H	-13.402821	-0.739029	-5.783781
H	-14.513196	0.291942	-3.776813
H	-13.098065	-4.624915	4.142510
H	-10.827863	-3.818835	4.813398
H	-14.339996	-3.455062	2.299055
H	-6.899268	-1.466746	4.838587
H	-4.696079	-0.384183	4.993982
H	-5.837689	2.370345	1.875076
H	-8.010962	1.278940	1.723468
H	-3.030527	0.937150	5.502013
H	3.645126	1.102332	4.323172
H	-0.546504	4.296579	1.968099
H	0.170720	2.694843	1.747672
H	0.483592	3.586734	3.228258
H	-1.198064	0.328499	2.235283
H	-0.793881	-1.383785	2.303842
H	-1.139966	-0.491252	3.789004
H	3.946981	-2.842577	2.117459
H	6.149078	-3.792218	1.537658
H	7.913612	0.104596	1.945540
H	5.720993	1.062195	2.496910
H	10.014730	-5.914678	0.290208
H	12.282458	-6.241251	-0.712297
H	13.774503	-4.256449	-1.088895

****

ΔΛ_closed_conformer_1_gas_phase

Ga	-9.407709	-12.149050	8.852251
O	-9.246852	-10.605269	7.477477
O	-11.254238	-12.189851	8.111618
C	-12.989698	-9.829846	5.898467
C	-11.941711	-9.010716	5.531135
C	-10.629857	-9.243212	6.035312
C	-10.409419	-10.318405	6.932416

C	-11.515434	-11.187621	7.299836
C	-12.780658	-10.916826	6.779776
C	-7.073922	-8.085382	5.617521
C	-5.864159	-8.732115	5.969232
C	-4.627259	-8.225983	5.592422
C	-4.537029	-7.006048	4.891497
C	-5.744947	-6.351390	4.564710
C	-6.983927	-6.871436	4.895332
C	-3.287990	-6.329392	4.564144
C	-3.085826	-4.988077	4.765358
C	-1.866390	-4.471808	4.264762
C	-1.185819	-5.443663	3.274517
S	-1.898148	-7.121404	3.763853
C	-1.301846	-3.247480	4.466732
C	-1.888411	-2.079708	5.181574
C	-0.797950	-0.968462	5.047995
C	0.056602	-1.398917	3.823724
C	-0.064559	-2.878618	3.826971
C	0.696262	-3.791883	3.152950
C	1.742036	-3.547622	2.234483
C	2.152104	-4.626304	1.494295
S	1.320029	-6.131813	1.950490
C	0.366864	-5.288857	3.344402
F	-2.166308	-2.305464	6.507672
F	-3.065125	-1.634559	4.632013
F	-0.011552	-0.958936	6.156143
F	-1.335638	0.272027	4.907870
F	-0.481749	-0.813555	2.695658
F	1.333528	-0.915365	3.921755
C	-1.748997	-5.106249	1.866696
C	0.957526	-5.774089	4.686752
C	3.071357	-4.518217	0.367325
C	4.156753	-5.385490	0.127031
C	5.041739	-5.135873	-0.914230
C	4.858726	-4.051867	-1.807046
C	3.756891	-3.193404	-1.577806
C	2.907308	-3.423970	-0.510795
N	-8.269347	-8.664725	6.000001
C	-9.559813	-8.347002	5.553371
O	-9.772568	-7.397852	4.783937
H	-8.250366	-9.468238	6.654860
Ga	9.853418	-5.802203	-3.937821
O	8.043069	-4.801177	-3.779480

O	9.746758	-5.048126	-5.775101
C	7.445700	-2.576401	-7.214766
C	6.528584	-2.458650	-6.190566
C	6.680903	-3.212432	-4.991297
C	7.793748	-4.079375	-4.850670
C	8.746152	-4.209830	-5.941516
C	8.552717	-3.449204	-7.094087
N	5.757838	-3.886035	-2.843858
C	5.628152	-3.056942	-3.967743
O	4.668464	-2.283142	-4.103722
H	6.618065	-4.464460	-2.860275
H	-13.991307	-9.643608	5.507009
H	-12.076575	-8.179301	4.846360
H	-13.600935	-11.572188	7.068427
H	-5.912048	-9.635445	6.565399
H	-3.725053	-8.750623	5.899409
H	-5.696925	-5.416108	4.012425
H	-7.898085	-6.376577	4.600159
H	-3.794019	-4.378657	5.315100
H	2.145580	-2.554905	2.073881
H	-1.359748	-5.794774	1.113289
H	-1.467878	-4.084125	1.589859
H	-2.839503	-5.176501	1.886495
H	2.031660	-5.570668	4.709188
H	0.479053	-5.248613	5.520045
H	0.799121	-6.847587	4.819982
H	4.345852	-6.224647	0.792656
H	5.908359	-5.773716	-1.041989
H	3.589355	-2.374031	-2.262006
H	2.065299	-2.753586	-0.357766
H	7.320467	-1.994436	-8.129520
H	5.665387	-1.804800	-6.266197
H	9.277263	-3.553192	-7.900215
O	9.698516	-6.087930	-1.885346
O	11.152464	-4.427977	-3.326759
C	12.562005	-3.775919	0.000269
C	11.795615	-4.626201	0.770697
C	10.787910	-5.435900	0.171649
C	10.584193	-5.370093	-1.229962
C	11.389491	-4.464545	-2.032556
C	12.362621	-3.693375	-1.398260
C	7.969659	-7.763026	1.067090
C	6.852706	-8.170831	0.297800

C	5.814397	-8.910406	0.849329
C	5.874425	-9.340135	2.190794
C	7.003789	-8.953736	2.946680
C	8.018930	-8.175177	2.420701
C	4.890215	-10.202285	2.835472
C	5.226675	-11.261726	3.638654
C	4.161618	-11.854256	4.355291
C	2.763947	-11.364360	3.918609
S	3.139197	-9.889902	2.802445
C	4.230683	-12.739294	5.394409
C	5.437180	-13.370128	5.986617
C	4.852189	-14.533445	6.835493
C	3.427371	-14.044889	7.252489
C	3.097763	-12.995956	6.247850
C	1.972329	-12.228861	6.181317
C	0.795736	-12.293293	6.966709
C	-0.252852	-11.526945	6.525277
S	0.115057	-10.675950	4.995407
C	1.956754	-11.027756	5.211155
F	6.131855	-12.527282	6.829915
F	6.356145	-13.835318	5.084517
F	5.627128	-14.846297	7.907401
F	4.750006	-15.643029	6.057701
F	2.555676	-15.106451	7.272455
F	3.463577	-13.577442	8.542802
C	2.088920	-12.453832	3.056151
C	2.562515	-9.827546	5.989414
C	-1.490488	-11.358131	7.277625
C	-2.784536	-11.498615	6.735665
C	-3.902550	-11.443502	7.556821
C	-3.796577	-11.179373	8.944253
C	-2.498926	-11.012286	9.484607
C	-1.388212	-11.122743	8.666750
N	8.946252	-6.996522	0.461177
C	9.992334	-6.291246	1.074696
O	10.194877	-6.351738	2.296851
H	8.907320	-6.854782	-0.565211
O	-7.544908	-11.527531	9.530241
O	-9.970369	-11.194856	10.503419
C	-7.985063	-9.838625	13.278617
C	-6.709676	-9.985461	12.772691
C	-6.503506	-10.549435	11.480828
C	-7.622846	-10.972555	10.719951

C	-8.963458	-10.803179	11.255322
C	-9.111295	-10.245769	12.524815
N	-4.956051	-11.106412	9.691052
C	-5.108679	-10.634564	11.002610
O	-4.137621	-10.282014	11.688949
H	-5.854106	-11.376690	9.248314
H	13.332184	-3.160209	0.468093
H	11.924769	-4.699271	1.845969
H	12.961265	-3.023473	-2.013448
H	6.820554	-7.907793	-0.753230
H	4.979957	-9.203702	0.215884
H	7.062253	-9.254691	3.989767
H	8.855034	-7.857999	3.027400
H	6.254877	-11.576111	3.773367
H	0.708347	-12.939354	7.832808
H	1.126542	-12.108257	2.668823
H	1.917424	-13.354516	3.655406
H	2.738156	-12.706920	2.213439
H	1.995016	-9.668607	6.909853
H	3.603921	-10.044046	6.252333
H	2.534421	-8.915532	5.390063
H	-2.916523	-11.714625	5.677505
H	-4.884613	-11.623363	7.135158
H	-2.402047	-10.791382	10.538163
H	-0.399889	-10.997126	9.102305
H	-8.132763	-9.406110	14.269705
H	-5.832213	-9.670603	13.328760
H	-10.121181	-10.130390	12.915284
O	-8.351613	-13.268679	7.461031
O	-9.576650	-13.944416	9.691765
C	-8.741550	-17.276822	8.393661
C	-8.071890	-16.936954	7.236030
C	-7.895120	-15.569699	6.877159
C	-8.428591	-14.556659	7.713734
C	-9.119898	-14.922908	8.938909
C	-9.264867	-16.275244	9.245666
C	-6.092275	-13.383379	4.404903
C	-5.756505	-12.011307	4.499835
C	-4.937512	-11.390910	3.566360
C	-4.465926	-12.101337	2.443794
C	-4.826527	-13.464386	2.338725
C	-5.597831	-14.105120	3.291980
C	-3.706344	-11.515711	1.346380

C	-3.999051	-11.742732	0.025018
C	-3.071586	-11.216340	-0.905092
C	-1.736273	-10.825545	-0.235103
S	-2.236509	-10.518409	1.559317
C	-3.174563	-11.117827	-2.261589
C	-4.264881	-11.637344	-3.132521
C	-3.808930	-11.254747	-4.577753
C	-2.272677	-11.043812	-4.466168
C	-2.089563	-10.606265	-3.059482
C	-0.993594	-10.020924	-2.489780
C	0.264702	-9.765792	-3.080302
C	1.275580	-9.430349	-2.215541
S	0.719403	-9.278748	-0.531748
C	-1.076000	-9.633685	-0.997047
F	-5.502554	-11.099053	-2.874719
F	-4.434461	-12.997323	-3.050619
F	-4.392127	-10.085573	-4.951489
F	-4.146546	-12.199681	-5.494608
F	-1.650684	-12.242840	-4.748266
F	-1.829060	-10.161715	-5.414943
C	-0.846418	-12.098655	-0.248286
C	-1.872344	-8.317388	-0.855989
C	2.674535	-9.339767	-2.614811
C	3.558191	-8.309054	-2.234918
C	4.848087	-8.261223	-2.747341
C	5.350038	-9.268773	-3.605982
C	4.471226	-10.318108	-3.968293
C	3.172514	-10.329900	-3.493041
N	-6.883819	-13.935575	5.392599
C	-7.127943	-15.292893	5.646221
O	-6.686773	-16.187734	4.909490
H	-7.320004	-13.307401	6.092961
O	8.697018	-7.517380	-4.103058
O	11.277639	-7.101034	-4.426884
C	11.048000	-10.578486	-5.584234
C	9.702321	-10.819182	-5.396975
C	8.850960	-9.803163	-4.874860
C	9.396138	-8.532194	-4.561332
C	10.817209	-8.290918	-4.750538
C	11.607543	-9.319013	-5.262706
N	6.655755	-9.171377	-4.045088
C	7.433159	-10.159233	-4.667795
O	6.966388	-11.268399	-4.967799

H	7.184312	-8.299541	-3.855418
H	-8.871779	-18.327059	8.660418
H	-7.652080	-17.688394	6.574654
H	-9.790198	-16.535892	10.163138
H	-6.167951	-11.431915	5.317395
H	-4.716947	-10.331418	3.679395
H	-4.458671	-14.036609	1.490728
H	-5.828111	-15.158262	3.214719
H	-4.906226	-12.249894	-0.282870
H	0.437209	-9.898860	-4.141674
H	0.100201	-11.920962	0.265821
H	-0.635113	-12.393005	-1.282298
H	-1.375327	-12.916403	0.247821
H	-1.409736	-7.542326	-1.473222
H	-2.905276	-8.466973	-1.188415
H	-1.889754	-7.977255	0.183316
H	3.218676	-7.506644	-1.583063
H	5.489060	-7.423735	-2.499759
H	4.843193	-11.107092	-4.606256
H	2.519673	-11.150209	-3.780185
H	11.692213	-11.363163	-5.984727
H	9.254323	-11.780888	-5.626662
H	12.669160	-9.122766	-5.404373

****

ΛΛ_open_conformer_1_gas_phase

Ga	11.691802	-0.727285	-0.446663
Ga	-11.966208	-0.754344	-0.353518
C	10.396420	-5.561289	0.850907
C	9.170238	-5.209699	0.322719
C	8.989231	-3.943824	-0.300296
C	10.087954	-3.048958	-0.385115
C	11.362206	-3.406289	0.211764
C	11.489784	-4.662920	0.801758
C	6.416023	-1.759744	-1.966228
C	6.564000	-0.417018	-2.382330
C	5.514112	0.272922	-2.965462
C	4.266098	-0.347651	-3.174959
C	4.128694	-1.689396	-2.774335
C	5.169936	-2.390852	-2.175468
C	3.135720	0.412893	-3.710883
C	2.790642	1.714762	-3.454583

C	1.534249	2.113626	-4.031257
C	0.947514	1.126147	-4.795640
S	1.939352	-0.313548	-4.788773
C	0.904480	3.406520	-3.737255
C	1.666662	4.663454	-4.056416
C	0.838198	5.812365	-3.444027
C	-0.562012	5.185758	-3.256101
C	-0.342152	3.690260	-3.253836
C	-1.436151	2.847117	-2.777286
C	-2.808887	3.292273	-2.835912
C	-3.713465	2.410876	-2.301525
S	-2.872742	0.993246	-1.703140
C	-1.316746	1.601880	-2.169279
F	2.943174	4.675900	-3.574218
F	1.767976	4.852474	-5.413599
F	1.344730	6.172141	-2.240279
F	0.809554	6.916823	-4.234334
F	-1.371542	5.584609	-4.295332
F	-1.145044	5.652369	-2.114482
C	-0.386468	1.142256	-5.476668
C	-0.104304	0.807304	-1.806246
C	-5.154389	2.545482	-2.083107
C	-5.721483	3.801292	-1.797367
C	-7.067732	3.946345	-1.479503
C	-7.903551	2.810161	-1.423852
C	-7.348338	1.549316	-1.747563
C	-6.006036	1.421406	-2.062262
O	10.043922	-1.869969	-0.966819
O	12.331562	-2.511071	0.155889
O	10.657297	0.822344	-1.320417
O	13.032645	0.356509	0.549974
O	12.652539	-0.650052	-2.193004
O	10.563306	-0.264314	1.233873
C	12.080969	1.487963	-5.133549
C	10.989221	2.219620	-4.708002
C	10.445654	2.013478	-3.410144
C	11.052920	1.062230	-2.550525
C	12.173069	0.264408	-3.015168
C	12.669637	0.509073	-4.295793
C	7.474856	2.745898	-1.238208
C	7.152563	2.093830	-0.024527
C	5.905886	2.239925	0.556819
C	4.919095	3.062165	-0.029260

C	5.257198	3.731853	-1.220294
C	6.504546	3.586258	-1.822414
C	3.597056	3.176196	0.587914
C	3.212092	2.881007	1.872064
C	1.800503	3.018943	2.113259
C	1.099509	3.458379	1.003820
S	2.187125	3.692022	-0.336313
C	1.210800	2.710182	3.411989
C	1.975323	3.068132	4.658538
C	0.932782	2.994801	5.794613
C	-0.169674	2.073865	5.222980
C	0.051219	2.063628	3.731736
C	-0.840200	1.253849	2.906678
C	-2.261476	1.143230	3.136184
C	-2.883948	0.203125	2.361417
S	-1.702872	-0.591806	1.343444
C	-0.389270	0.370518	1.932919
F	2.979736	2.165308	4.917488
F	2.561503	4.296073	4.612942
F	1.446185	2.534447	6.960306
F	0.425250	4.237738	6.020180
F	-1.404421	2.542092	5.593736
F	-0.060348	0.822037	5.769180
C	-0.368515	3.702432	0.829003
C	0.996369	0.136879	1.416673
C	-4.292195	-0.256055	2.370378
C	-5.309413	0.366770	1.626693
C	-6.615302	-0.115201	1.654495
C	-6.960582	-1.252077	2.420518
C	-5.938205	-1.878669	3.169149
C	-4.638384	-1.384981	3.129487
C	-6.853974	-2.159316	-2.376381
C	-5.762283	-2.254574	-3.269964
C	-4.483610	-2.507082	-2.787106
C	-4.218071	-2.676857	-1.415072
C	-5.307214	-2.551121	-0.527467
C	-6.589867	-2.305191	-0.994645
C	-2.878779	-3.004541	-0.921055
C	-2.518225	-3.700478	0.206855
C	-1.097755	-3.819197	0.401767
C	-0.358084	-3.219201	-0.600714
S	-1.423641	-2.500894	-1.780629
C	-0.534156	-4.655197	1.463471

C	-1.162000	-6.015421	1.682812
C	-0.115693	-6.803245	2.498251
C	0.686741	-5.689791	3.199374
C	0.470807	-4.458821	2.351982
C	1.235691	-3.262006	2.759476
C	2.614343	-3.024263	2.440374
C	3.181540	-1.976612	3.132790
S	1.988540	-1.307978	4.251598
C	0.741159	-2.406034	3.721742
F	-2.315894	-5.911746	2.419088
F	-1.480712	-6.667000	0.534035
F	-0.672627	-7.687251	3.363346
F	0.692870	-7.491378	1.650519
F	1.999218	-6.029883	3.322957
F	0.202090	-5.514522	4.470885
C	1.126094	-3.204736	-0.816457
C	-0.624711	-2.366368	4.340071
C	4.526007	-1.406068	3.023481
C	4.956301	-0.364436	3.869135
C	6.242785	0.159506	3.814400
C	7.174538	-0.348779	2.883272
C	6.740091	-1.365205	2.000267
C	5.455559	-1.880209	2.069143
C	10.597940	1.199297	3.156570
C	11.375099	2.091474	3.946570
C	12.681818	2.375188	3.602766
C	13.268371	1.793726	2.452775
C	12.548537	0.916295	1.641848
C	11.182219	0.592770	2.015056
N	8.717056	2.466902	-1.783990
N	7.522542	-2.365338	-1.394166
N	8.482706	0.089332	2.760734
C	9.194979	0.966783	3.575565
C	7.634699	-3.621562	-0.810419
C	9.214646	2.761713	-3.049888
O	6.667590	-4.395096	-0.710211
O	8.652485	3.559437	-3.816393
O	8.687575	1.507199	4.570630
H	8.408532	-1.829445	-1.373155
H	9.317694	1.800869	-1.265547
H	9.058820	-0.309616	1.998625
C	-12.002781	-0.887988	-5.556691
C	-10.683903	-1.266288	-5.409655

C	-10.118095	-1.429053	-4.113274
C	-10.927251	-1.214673	-2.967130
C	-12.306751	-0.785787	-3.130123
C	-12.812892	-0.643523	-4.422300
O	-10.517659	-1.371099	-1.726873
O	-13.007169	-0.555667	-2.039315
O	-10.573103	-1.385225	1.060793
O	-13.221419	0.358591	0.752371
O	-12.821512	-2.455827	0.176227
O	-10.991817	1.046670	-0.430340
C	-11.893352	-4.947591	2.706274
C	-10.681348	-4.439755	3.126288
C	-10.167120	-3.233472	2.570140
C	-10.926276	-2.532699	1.598469
C	-12.181141	-3.098941	1.131339
C	-12.640416	-4.283799	1.704955
C	-11.274011	3.389064	0.100801
C	-12.107639	4.365315	0.713392
C	-13.292180	3.992843	1.318938
C	-13.698001	2.636422	1.346161
C	-12.916874	1.638867	0.759383
C	-11.677744	2.029627	0.114019
N	-8.270644	-1.719627	2.360695
N	-8.161386	-1.905927	-2.761140
N	-9.231606	2.822754	-1.042559
C	-9.986171	3.844420	-0.479643
C	-8.683424	-1.788575	-4.049520
C	-8.813519	-2.828759	3.011897
O	-7.986277	-1.965262	-5.061057
O	-8.183499	-3.453928	3.879665
O	-9.601892	5.025072	-0.452430
H	-8.875742	-1.778743	-2.023251
H	-8.935025	-1.258933	1.719319
H	-9.706593	1.903411	-0.990059
H	10.525492	-6.534493	1.327623
H	8.311809	-5.872620	0.372745
H	12.451613	-4.926473	1.240091
H	7.519166	0.079359	-2.236511
H	5.661079	1.307446	-3.259615
H	3.168357	-2.184920	-2.901745
H	5.044885	-3.413494	-1.848568
H	3.371500	2.339937	-2.789698
H	-3.107593	4.235438	-3.274586

H	-0.702785	2.173525	-5.660728
H	-1.159783	0.676163	-4.850916
H	-0.361522	0.610539	-6.435194
H	0.195826	0.124295	-2.605915
H	-0.306203	0.198990	-0.926881
H	0.742896	1.461157	-1.594904
H	-5.076574	4.677682	-1.781460
H	-7.490744	4.910035	-1.230486
H	-7.988255	0.670348	-1.739803
H	-5.616391	0.434357	-2.297642
H	12.490903	1.651595	-6.132145
H	10.504395	2.952790	-5.346064
H	13.516552	-0.084567	-4.638292
H	7.887359	1.438349	0.436396
H	5.683186	1.675203	1.456816
H	4.530087	4.387015	-1.695429
H	6.744390	4.091343	-2.747948
H	3.904713	2.534816	2.629340
H	-2.794243	1.738189	3.866133
H	-0.564792	4.479606	0.083576
H	-0.824308	3.998374	1.779587
H	-0.887284	2.793328	0.497087
H	1.135994	-0.907681	1.130509
H	1.222136	0.770230	0.554638
H	1.739783	0.371969	2.175175
H	-5.075078	1.224257	1.001488
H	-7.376406	0.374221	1.051693
H	-6.189487	-2.746055	3.761208
H	-3.863294	-1.887743	3.704675
H	-5.949945	-2.139047	-4.327525
H	-3.669411	-2.597241	-3.503756
H	-5.144213	-2.617203	0.543954
H	-7.408248	-2.210609	-0.285980
H	-3.243414	-4.125649	0.889777
H	3.158840	-3.611777	1.711093
H	1.570265	-2.222645	-0.616886
H	1.613956	-3.923215	-0.153134
H	1.380160	-3.468179	-1.849623
H	-1.027988	-1.348101	4.343924
H	-0.608908	-2.727477	5.376877
H	-1.312063	-3.000258	3.773259
H	4.268780	0.060422	4.596366
H	6.554840	0.948633	4.483583

H	7.432251	-1.752894	1.258315
H	5.186156	-2.662780	1.365114
H	10.899120	2.538239	4.814386
H	13.270418	3.062063	4.214243
H	14.293890	2.026084	2.167716
H	-12.427074	-0.763013	-6.554910
H	-10.037242	-1.438917	-6.264558
H	-13.849755	-0.327460	-4.530025
H	-12.276154	-5.875318	3.136060
H	-10.075230	-4.945836	3.871154
H	-13.587716	-4.686751	1.348827
H	-11.771051	5.397935	0.693607
H	-13.925089	4.748619	1.788888
H	-14.628550	2.339530	1.828635

****

ΛΛ_closed_conformer_1_gas_phase

Ga	9.457112	12.227863	-8.602453
O	9.366897	10.948594	-6.969922
O	11.161677	12.729270	-7.707595
C	12.970804	11.125419	-4.944578
C	12.031201	10.205442	-4.527140
C	10.768146	10.116040	-5.178892
C	10.487010	10.968095	-6.278419
C	11.478305	11.942298	-6.703126
C	12.696980	11.995059	-6.026329
C	7.375873	8.555436	-4.784177
C	6.143277	8.949453	-5.361862
C	4.944468	8.362054	-4.986606
C	4.924803	7.301905	-4.055778
C	6.155238	6.897803	-3.499243
C	7.353202	7.512114	-3.828311
C	3.719118	6.542799	-3.744527
C	3.655564	5.173176	-3.728910
C	2.365654	4.632299	-3.494068
C	1.377871	5.669472	-2.929984
S	2.161901	7.323257	-3.387752
C	1.916852	3.358968	-3.693005
C	2.714812	2.147004	-4.024462
C	1.644040	1.093330	-4.419663
C	0.366227	1.535453	-3.656878
C	0.520971	3.011902	-3.559148

C	-0.446574	3.957611	-3.388983
C	-1.830471	3.788791	-3.121371
C	-2.475249	4.910935	-2.670507
S	-1.429366	6.348741	-2.614340
C	-0.044715	5.438251	-3.516848
F	3.630896	2.313544	-5.028223
F	3.425471	1.654056	-2.949708
F	1.410278	1.169331	-5.758299
F	2.022057	-0.178838	-4.133326
F	0.362358	0.898587	-2.434220
F	-0.762549	1.116198	-4.305946
C	1.416128	5.547178	-1.386499
C	-0.141108	5.812324	-5.016744
C	-3.867676	4.936170	-2.236172
C	-4.275387	5.505534	-1.011389
C	-5.581245	5.362987	-0.566999
C	-6.562175	4.701007	-1.346403
C	-6.161118	4.157062	-2.589728
C	-4.841203	4.261516	-2.999985
N	8.520989	9.209819	-5.188708
C	9.799403	9.148550	-4.624997
O	10.075001	8.364157	-3.704364
H	8.458969	9.882834	-5.972169
Ga	-9.735907	5.643805	3.278952
O	-9.224549	5.135909	1.332701
O	-11.552025	5.616892	2.466949
C	-12.690296	4.394745	-0.802738
C	-11.479250	4.165182	-1.422619
C	-10.257091	4.421387	-0.738080
C	-10.291067	4.896559	0.598717
C	-11.571249	5.148116	1.238729
C	-12.739652	4.888288	0.522173
N	-7.845535	4.625847	-0.845670
C	-9.009242	4.210195	-1.499805
O	-8.986025	3.736510	-2.646102
H	-8.023099	4.958729	0.117845
H	13.933543	11.191963	-4.434388
H	12.213252	9.537635	-3.690810
H	13.429890	12.729672	-6.356564
H	6.146991	9.713267	-6.132901
H	4.023579	8.686015	-5.465307
H	6.156261	6.091629	-2.769218
H	8.284795	7.214252	-3.370026

H	4.499008	4.560261	-4.023915
H	-2.312145	2.818343	-3.132212
H	0.837434	6.339795	-0.907176
H	1.002439	4.578910	-1.083506
H	2.453822	5.610045	-1.047128
H	-1.126360	5.526166	-5.395234
H	0.626127	5.270559	-5.581079
H	0.001542	6.883817	-5.172940
H	-3.551553	6.008308	-0.374729
H	-5.858750	5.743338	0.410996
H	-6.904537	3.664634	-3.199256
H	-4.546643	3.829110	-3.953506
H	-13.622221	4.202405	-1.337642
H	-11.416392	3.800742	-2.443315
H	-13.691292	5.080683	1.015374
O	-9.147605	7.579506	2.810140
O	-10.354775	6.574838	4.924136
C	-10.670759	10.156454	5.666788
C	-10.018108	10.698972	4.579055
C	-9.460398	9.855411	3.576327
C	-9.597481	8.447580	3.691648
C	-10.276722	7.885019	4.846785
C	-10.798212	8.754278	5.805079
C	-7.170375	9.965603	0.602456
C	-6.436888	8.910269	0.005333
C	-5.504984	9.147798	-0.993739
C	-5.303808	10.452189	-1.492605
C	-6.050919	11.496706	-0.911359
C	-6.947382	11.278221	0.122767
C	-4.457401	10.738129	-2.645240
C	-4.840486	11.517679	-3.706351
C	-3.902387	11.594829	-4.767285
C	-2.500227	11.107678	-4.359625
S	-2.823335	10.060463	-2.824299
C	-4.082715	12.035825	-6.046153
C	-5.264992	12.738230	-6.615550
C	-5.012649	12.708779	-8.148058
C	-3.468618	12.622310	-8.284349
C	-3.058004	11.893005	-7.054417
C	-1.923824	11.168115	-6.835834
C	-0.775267	11.026776	-7.658064
C	0.328660	10.512338	-7.029570
S	0.026192	10.056828	-5.336782

C	-1.827739	10.345662	-5.537983
F	-6.475334	12.176815	-6.308470
F	-5.355344	14.057051	-6.220847
F	-5.568091	11.583150	-8.674178
F	-5.545387	13.778103	-8.793068
F	-2.977801	13.908799	-8.352984
F	-3.112550	12.017856	-9.458666
C	-1.694067	12.352510	-3.913358
C	-2.486415	8.971464	-5.814849
C	1.639799	10.379634	-7.655101
C	2.827340	10.835746	-7.044952
C	4.025628	10.833017	-7.742988
C	4.115575	10.319654	-9.060672
C	2.930536	9.833066	-9.661932
C	1.729092	9.887716	-8.972857
N	-8.056496	9.650454	1.611816
C	-8.724212	10.518906	2.481289
O	-8.662910	11.751375	2.354567
H	-8.237024	8.653581	1.822174
O	7.799021	11.222699	-9.344137
O	10.361353	11.052403	-9.928200
C	8.971608	8.941323	-12.591137
C	7.628010	8.996719	-12.282611
C	7.173794	9.753196	-11.164839
C	8.112408	10.469868	-10.377832
C	9.526802	10.395352	-10.703073
C	9.920926	9.634218	-11.803708
N	5.349997	10.327092	-9.676814
C	5.727168	9.704351	-10.870540
O	4.905876	9.126247	-11.598668
H	6.139164	10.799823	-9.203167
H	-11.088715	10.810497	6.434337
H	-9.896030	11.770940	4.458484
H	-11.304778	8.317826	6.664595
H	-6.630410	7.891475	0.325484
H	-4.974069	8.306844	-1.433061
H	-5.897465	12.510471	-1.274111
H	-7.489304	12.092254	0.581314
H	-5.844379	11.917544	-3.786477
H	-0.726141	11.425515	-8.664295
H	-0.728819	12.072907	-3.485620
H	-1.519485	13.006423	-4.774970
H	-2.269072	12.902404	-3.163071

H	-2.044560	8.536734	-6.715765
H	-3.561873	9.104934	-5.976704
H	-2.341841	8.281909	-4.980314
H	2.800515	11.252398	-6.041075
H	4.913920	11.255346	-7.283996
H	2.990788	9.424459	-10.659968
H	0.825587	9.518606	-9.452823
H	9.311519	8.353079	-13.445549
H	6.882779	8.462184	-12.863530
H	10.984033	9.588799	-12.035259
O	8.126858	13.450799	-7.578458
O	9.484368	13.810844	-9.806365
C	8.166396	17.203126	-9.322318
C	7.429163	17.026070	-8.169694
C	7.368402	15.750430	-7.539456
C	8.089634	14.661402	-8.094359
C	8.854669	14.855090	-9.314781
C	8.875100	16.122287	-9.897604
C	5.492411	13.893421	-4.868872
C	5.333208	12.495210	-4.701884
C	4.471305	11.970614	-3.750591
C	3.768221	12.821402	-2.871598
C	3.945483	14.211271	-3.026288
C	4.764945	14.747957	-4.006726
C	2.972271	12.325381	-1.754733
C	3.061153	12.805592	-0.473394
C	2.263359	12.125661	0.482111
C	1.181473	11.242457	-0.165087
S	1.807710	10.992679	-1.926922
C	2.330360	12.164117	1.844644
C	3.133211	13.082621	2.697464
C	3.033395	12.456007	4.115314
C	1.691703	11.675259	4.099421
C	1.544868	11.278699	2.673104
C	0.820750	10.249260	2.148109
C	-0.089749	9.370381	2.791445
C	-0.906458	8.662114	1.948831
S	-0.564173	8.953882	0.227715
C	0.992830	9.931489	0.651641
F	4.440711	13.229572	2.318876
F	2.616491	14.360379	2.755802
F	4.056693	11.576124	4.291655
F	3.100838	13.379458	5.108309

F	0.701733	12.527481	4.540392
F	1.718775	10.634754	4.986943
C	-0.113088	12.088864	-0.246553
C	2.198457	8.967640	0.523002
C	-1.980512	7.782212	2.395925
C	-3.289225	7.846657	1.872705
C	-4.312837	7.100696	2.437367
C	-4.074677	6.206738	3.510680
C	-2.755672	6.118683	4.015946
C	-1.751104	6.906785	3.476496
N	6.345953	14.336346	-5.858139
C	6.504997	15.637151	-6.346632
O	5.928824	16.607520	-5.831652
H	6.921053	13.642685	-6.366691
O	-7.754141	5.277352	3.781040
O	-9.937336	3.835036	4.081145
C	-7.574522	1.803453	6.022378
C	-6.415507	2.532475	5.853099
C	-6.419397	3.726179	5.076482
C	-7.631671	4.175417	4.491039
C	-8.844876	3.394447	4.665177
C	-8.786207	2.228356	5.428559
N	-5.141073	5.479486	3.998108
C	-5.121487	4.407685	4.895992
O	-4.079424	4.042888	5.461583
H	-6.089531	5.687090	3.640479
H	8.201197	18.182693	-9.802754
H	6.866497	17.836965	-7.717589
H	9.453013	16.251684	-10.811463
H	5.920199	11.823990	-5.320554
H	4.390166	10.891274	-3.648131
H	3.399602	14.881937	-2.366688
H	4.866293	15.815562	-4.135361
H	3.797568	13.549576	-0.193556
H	-0.231011	9.361462	3.865630
H	-0.893426	11.574455	-0.811584
H	-0.486249	12.289090	0.763953
H	0.110681	13.040979	-0.735773
H	2.054769	8.120998	1.200061
H	3.119691	9.491413	0.801609
H	2.305022	8.592012	-0.496940
H	-3.519609	8.530643	1.059604
H	-5.326916	7.217931	2.068715

H	-2.559400	5.428485	4.823254
H	-0.744724	6.833730	3.882429
H	-7.560566	0.885721	6.613275
H	-5.471232	2.218975	6.287395
H	-9.703652	1.654486	5.550402

****

ΔΔ_open_conformer_1_DMF

Ga	-11.306803	0.300560	0.116854
Ga	11.612520	0.486181	0.115352
C	-10.222652	4.770069	-2.172377
C	-9.060990	4.678317	-1.426201
C	-8.896628	3.642064	-0.468863
C	-9.947303	2.709233	-0.275680
C	-11.141036	2.779974	-1.089883
C	-11.258630	3.820164	-2.012307
C	-6.427192	2.040703	1.877572
C	-6.607018	0.798092	2.521594
C	-5.591345	0.225232	3.268937
C	-4.344999	0.870047	3.400717
C	-4.188750	2.130105	2.797875
C	-5.204109	2.716534	2.044428
C	-3.220536	0.157688	4.014533
C	-2.957938	-1.179454	3.885381
C	-1.683858	-1.577410	4.411291
C	-0.989062	-0.543976	5.001025
S	-1.914867	0.940660	4.900217
C	-1.171136	-2.935263	4.194073
C	-1.945756	-4.095789	4.758640
C	-1.326829	-5.352533	4.109357
C	0.056312	-4.860768	3.615925
C	-0.055650	-3.354659	3.538893
C	0.992225	-2.629112	2.819458
C	2.336676	-3.153490	2.747138
C	3.202283	-2.370506	2.032334
S	2.363730	-0.968396	1.406416
C	0.849107	-1.443340	2.107936
F	-3.288684	-4.028717	4.503738
F	-1.821535	-4.167559	6.119487
F	-2.081364	-5.741474	3.049172
F	-1.230707	-6.391591	4.967389
F	1.018295	-5.267086	4.504217

F	0.374558	-5.424609	2.414210
C	0.389260	-0.553895	5.583379
C	-0.353364	-0.604481	1.811183
C	4.613890	-2.566819	1.687875
C	5.114906	-3.831701	1.335278
C	6.430985	-4.001466	0.905833
C	7.288263	-2.888151	0.812664
C	6.799696	-1.622632	1.199794
C	5.490016	-1.466597	1.620810
O	-9.917818	1.726399	0.606153
O	-12.056046	1.837438	-0.907285
O	-10.251932	-0.981923	1.318290
O	-12.386079	-1.131394	-0.727476
O	-12.371491	0.493852	1.791743
O	-9.973083	-0.269911	-1.362544
C	-11.435533	-0.422703	5.234955
C	-10.286621	-1.156570	4.996415
C	-9.827562	-1.368805	3.669372
C	-10.571452	-0.826418	2.589231
C	-11.750920	-0.027496	2.841801
C	-12.163561	0.147051	4.163644
C	-6.977151	-2.669978	1.602659
C	-6.804368	-2.346943	0.240534
C	-5.616645	-2.617603	-0.413183
C	-4.538824	-3.230901	0.260815
C	-4.735933	-3.590924	1.606225
C	-5.927777	-3.316682	2.278383
C	-3.253759	-3.392339	-0.424457
C	-2.960213	-3.136894	-1.740207
C	-1.562233	-3.209709	-2.053280
C	-0.780193	-3.570155	-0.970820
S	-1.777357	-3.816704	0.434687
C	-1.057890	-2.880262	-3.381761
C	-1.813792	-3.342599	-4.595900
C	-0.833836	-3.150362	-5.777840
C	0.210065	-2.140995	-5.233477
C	0.010518	-2.120002	-3.740024
C	0.807929	-1.187353	-2.946548
C	2.216038	-0.967636	-3.145256
C	2.709088	0.105242	-2.451480
S	1.426843	0.876070	-1.541167
C	0.239181	-0.270054	-2.073223
F	-2.935247	-2.567003	-4.811340

F	-2.250695	-4.627575	-4.512732
F	-1.447888	-2.698197	-6.894351
F	-0.235295	-4.333224	-6.064605
F	1.474032	-2.519418	-5.596939
F	0.003747	-0.908884	-5.791595
C	0.704350	-3.735418	-0.876745
C	-1.181893	-0.150531	-1.621622
C	4.081161	0.642404	-2.418126
C	5.163717	-0.136135	-1.971477
C	6.450129	0.390618	-1.923900
C	6.704709	1.719223	-2.325705
C	5.628270	2.493297	-2.803417
C	4.342902	1.959102	-2.829238
C	6.634175	1.826727	2.422059
C	5.516448	1.773449	3.279822
C	4.267620	2.197365	2.831578
C	4.071042	2.696451	1.531241
C	5.200040	2.764742	0.690470
C	6.448783	2.344447	1.123064
C	2.757333	3.129993	1.044073
C	2.462667	3.968040	-0.004244
C	1.055545	4.145780	-0.233338
C	0.266248	3.448627	0.661042
S	1.264503	2.566439	1.783593
C	0.540605	5.100815	-1.218335
C	1.118781	6.496476	-1.221904
C	0.126076	7.340201	-2.057960
C	-0.623860	6.280435	-2.899549
C	-0.388243	4.972160	-2.190611
C	-1.054600	3.780455	-2.751203
C	-2.392643	3.365143	-2.453220
C	-2.825472	2.301713	-3.211751
S	-1.569204	1.840660	-4.356732
C	-0.467087	3.052120	-3.764182
F	2.351541	6.511684	-1.826869
F	1.286182	7.014261	0.023423
F	0.745681	8.269677	-2.819224
F	-0.742872	7.968098	-1.227152
F	-1.950217	6.578746	-3.006502
F	-0.123369	6.257910	-4.173952
C	-1.222939	3.425512	0.810583
C	0.889529	3.215056	-4.378650
C	-4.073641	1.541656	-3.096368

C	-4.242053	0.290961	-3.720440
C	-5.411826	-0.454148	-3.592620
C	-6.477494	0.036969	-2.814220
C	-6.314505	1.285239	-2.176342
C	-5.147423	2.020099	-2.313796
C	-9.495570	-2.230677	-2.699371
C	-9.989180	-3.467768	-3.195099
C	-11.263177	-3.899518	-2.875004
C	-12.095099	-3.118979	-2.038962
C	-11.654859	-1.900045	-1.523394
C	-10.326945	-1.434624	-1.870114
N	-8.195529	-2.294470	2.160325
N	-7.504461	2.500241	1.127846
N	-7.693711	-0.614570	-2.620073
C	-8.110250	-1.846510	-3.088805
C	-7.590895	3.556629	0.246663
C	-8.539156	-2.097786	3.482405
O	-6.655727	4.347183	0.042186
O	-7.824416	-2.468573	4.425914
O	-7.384243	-2.577740	-3.778993
H	-8.356918	1.916617	1.141721
H	-8.870653	-1.864505	1.507251
H	-8.396882	-0.140190	-2.033676
C	11.409208	-1.125738	5.022503
C	10.156140	-0.543069	4.994510
C	9.712257	0.174628	3.849542
C	10.577029	0.310261	2.733791
C	11.874698	-0.336545	2.755602
C	12.265284	-1.029989	3.901627
O	10.284527	0.984996	1.637755
O	12.625856	-0.232316	1.672171
O	10.327171	1.483920	-1.127708
O	12.725667	-0.501149	-1.224901
O	12.615238	2.188738	-0.025230
O	10.450979	-1.178425	-0.107276
C	11.577402	5.417934	-1.442079
C	10.343538	5.073881	-1.962906
C	9.855631	3.742593	-1.857861
C	10.660140	2.757442	-1.235174
C	11.931424	3.132758	-0.653665
C	12.368302	4.451595	-0.779750
C	10.563129	-3.444835	-0.945145
C	11.311043	-4.382934	-1.705890

C	12.511173	-4.018449	-2.290466
C	13.015364	-2.705237	-2.141132
C	12.317238	-1.747003	-1.403227
C	11.062658	-2.127541	-0.792108
N	8.008099	2.198068	-2.173687
N	7.911559	1.372026	2.749553
N	8.599773	-2.919410	0.349209
C	9.258434	-3.891828	-0.377431
C	8.322649	0.704015	3.890059
C	8.479562	3.486332	-2.364609
O	7.580219	0.539939	4.869084
O	7.796348	4.376230	-2.890966
O	8.799082	-5.028967	-0.555100
H	8.636457	1.418372	2.017953
H	8.696050	1.569986	-1.734423
H	9.114605	-2.025231	0.382375
H	-10.340792	5.569906	-2.900898
H	-8.246631	5.383975	-1.558419
H	-12.161824	3.880043	-2.616969
H	-7.554303	0.278823	2.410660
H	-5.763945	-0.734199	3.747514
H	-3.238153	2.647901	2.882606
H	-5.054251	3.673112	1.562616
H	-3.601350	-1.844790	3.324948
H	2.648496	-4.068429	3.235073
H	0.700741	-1.581043	5.795024
H	1.114824	-0.120637	4.881399
H	0.444262	0.020302	6.515086
H	-0.529469	0.140200	2.591442
H	-0.217356	-0.060586	0.876423
H	-1.249537	-1.220697	1.725052
H	4.451442	-4.692316	1.365871
H	6.796377	-4.975833	0.611315
H	7.455630	-0.758328	1.149870
H	5.143538	-0.477512	1.902805
H	-11.782200	-0.269184	6.255122
H	-9.705974	-1.578773	5.811155
H	-13.057233	0.738169	4.355790
H	-7.605525	-1.837101	-0.286725
H	-5.518625	-2.320432	-1.451218
H	-3.945254	-4.089153	2.159439
H	-6.042319	-3.577214	3.321996
H	-3.702805	-2.840379	-2.469933

H	2.828504	-1.575664	-3.799765
H	0.981159	-4.468736	-0.112723
H	1.116453	-4.058738	-1.838179
H	1.187653	-2.785301	-0.617142
H	-1.417023	0.866877	-1.300600
H	-1.394333	-0.828074	-0.789140
H	-1.866478	-0.419511	-2.427827
H	4.988463	-1.152761	-1.630889
H	7.267265	-0.218405	-1.544241
H	5.809081	3.508858	-3.123504
H	3.523927	2.577333	-3.186417
H	5.636948	1.385780	4.280677
H	3.431227	2.141438	3.524305
H	5.093291	3.121043	-0.327279
H	7.294362	2.390016	0.441884
H	3.220023	4.460290	-0.602456
H	-2.985977	3.819091	-1.669135
H	-1.656203	2.488107	0.441261
H	-1.674276	4.249340	0.251958
H	-1.510740	3.531348	1.861667
H	1.426265	2.260517	-4.412893
H	0.822563	3.597234	-5.405150
H	1.486792	3.921040	-3.794978
H	-3.437827	-0.138562	-4.311766
H	-5.504843	-1.413044	-4.081756
H	-7.125995	1.675359	-1.569964
H	-5.080825	2.975779	-1.802056
H	-9.335222	-4.058344	-3.829035
H	-11.632785	-4.847005	-3.262452
H	-13.095507	-3.460304	-1.778933
H	11.739801	-1.671323	5.904428
H	9.476672	-0.624582	5.836920
H	13.245598	-1.503313	3.909916
H	11.941526	6.439881	-1.528582
H	9.712382	5.809255	-2.451609
H	13.330985	4.722023	-0.349671
H	10.909795	-5.385828	-1.815754
H	13.077414	-4.743904	-2.871679
H	13.959743	-2.419697	-2.601260

****

ΔΔ_closed_conformer_1_DMF

Ga	-9.329508	-12.203261	8.577368
O	-9.283314	-11.047054	6.880534
O	-10.969894	-12.906537	7.684720
C	-12.662270	-11.810085	4.611329
C	-11.772209	-10.850206	4.166588
C	-10.589979	-10.561377	4.902227
C	-10.335628	-11.256482	6.110362
C	-11.265470	-12.268931	6.565866
C	-12.409422	-12.521587	5.806521
C	-7.333169	-8.689672	4.617202
C	-6.120199	-8.987636	5.276041
C	-4.944384	-8.329026	4.947606
C	-4.946591	-7.300899	3.985208
C	-6.161452	-6.986299	3.350884
C	-7.335648	-7.672980	3.640926
C	-3.746826	-6.511802	3.700444
C	-3.690731	-5.147376	3.693626
C	-2.387267	-4.610964	3.490852
C	-1.394445	-5.649564	2.940568
S	-2.187270	-7.299608	3.382525
C	-1.935737	-3.345713	3.707632
C	-2.733583	-2.130226	4.044866
C	-1.663607	-1.080004	4.458701
C	-0.371909	-1.527655	3.718820
C	-0.535971	-3.005390	3.599534
C	0.426507	-3.953683	3.444550
C	1.822206	-3.793160	3.207124
C	2.454220	-4.921851	2.770325
S	1.399663	-6.349215	2.678170
C	0.012303	-5.431073	3.560890
F	-3.651170	-2.315314	5.040424
F	-3.431372	-1.636805	2.967986
F	-1.452723	-1.164896	5.798538
F	-2.030455	0.185941	4.159763
F	-0.334391	-0.888760	2.500992
F	0.745895	-1.136323	4.395495
C	-1.410840	-5.523813	1.397620
C	0.086322	-5.807913	5.060842
C	3.857264	-4.988713	2.356162
C	4.236549	-5.534505	1.114689
C	5.557890	-5.478662	0.695273

C	6.563920	-4.929368	1.519864
C	6.190522	-4.398707	2.771394
C	4.856493	-4.418225	3.164216
N	-8.455083	-9.422353	4.990700
C	-9.660590	-9.556448	4.321277
O	-9.928804	-8.914134	3.296611
H	-8.379113	-10.020017	5.826670
Ga	9.653608	-5.689895	-3.175252
O	9.204384	-5.317053	-1.205410
O	11.487136	-5.852966	-2.412364
C	12.712743	-5.221453	0.994471
C	11.520087	-4.960042	1.642776
C	10.286899	-4.993431	0.935233
C	10.288321	-5.284133	-0.451405
C	11.539928	-5.566366	-1.123420
C	12.724195	-5.527972	-0.385548
N	7.866211	-4.940157	1.029140
C	9.050002	-4.752031	1.724317
O	9.075670	-4.439209	2.922657
H	8.003788	-5.195825	0.040693
H	-13.562574	-12.027460	4.039555
H	-11.945115	-10.302186	3.245755
H	-13.104117	-13.283578	6.155207
H	-6.114506	-9.740563	6.057527
H	-4.027881	-8.584962	5.472684
H	-6.178352	-6.198408	2.602716
H	-8.254804	-7.436639	3.124706
H	-4.547231	-4.532290	3.945169
H	2.319088	-2.830666	3.247615
H	-0.836287	-6.322237	0.922847
H	-0.984745	-4.560005	1.097693
H	-2.442658	-5.573820	1.038486
H	1.064396	-5.524910	5.459915
H	-0.686140	-5.268016	5.619593
H	-0.056694	-6.879904	5.213400
H	3.487546	-5.964616	0.454909
H	5.825622	-5.854743	-0.286676
H	6.949061	-3.979446	3.416262
H	4.580566	-3.996401	4.126843
H	13.651211	-5.197537	1.545248
H	11.491467	-4.732372	2.703627
H	13.660302	-5.740729	-0.898643
O	9.016264	-7.615585	-2.864160

O	10.174650	-6.467675	-4.937060
C	9.972130	-9.926570	-6.165083
C	9.340239	-10.533472	-5.095602
C	8.976653	-9.780119	-3.945673
C	9.284156	-8.398050	-3.894161
C	9.934688	-7.764404	-5.022311
C	10.266739	-8.544322	-6.131654
C	6.956375	-10.042144	-0.775474
C	6.345447	-8.985648	-0.065542
C	5.486040	-9.235258	0.994268
C	5.248885	-10.554471	1.425743
C	5.880998	-11.603825	0.736005
C	6.707437	-11.365016	-0.357308
C	4.437403	-10.838519	2.611676
C	4.832658	-11.610240	3.666450
C	3.905202	-11.652670	4.746921
C	2.506685	-11.148696	4.350810
S	2.823885	-10.122744	2.804763
C	4.093012	-12.074707	6.026797
C	5.278034	-12.781031	6.597023
C	5.038588	-12.740668	8.133665
C	3.496330	-12.620855	8.283946
C	3.080928	-11.903384	7.044051
C	1.961502	-11.161541	6.829653
C	0.820624	-10.981045	7.665531
C	-0.265503	-10.452605	7.030041
S	0.024624	-10.036229	5.327836
C	1.869725	-10.354583	5.522621
F	6.480496	-12.218892	6.272519
F	5.352012	-14.096329	6.204196
F	5.617900	-11.623143	8.645679
F	5.546153	-13.819164	8.770922
F	2.977324	-13.892215	8.368690
F	3.159949	-11.986863	9.443721
C	1.679679	-12.386470	3.925129
C	2.552816	-8.989286	5.781578
C	-1.588031	-10.269065	7.633803
C	-2.747700	-10.802901	7.039950
C	-3.973026	-10.713071	7.684288
C	-4.102483	-10.036272	8.916352
C	-2.946346	-9.485488	9.505200
C	-1.713598	-9.621074	8.874653
N	7.780636	-9.696476	-1.841811

C	8.247936	-10.500947	-2.868153
O	8.042762	-11.722049	-2.903777
H	8.030749	-8.702891	-1.950289
O	-7.758897	-11.074425	9.275123
O	-10.352295	-10.948750	9.741378
C	-9.197350	-8.195552	11.883800
C	-7.839316	-8.235450	11.628501
C	-7.297457	-9.195262	10.730000
C	-8.161163	-10.128314	10.104598
C	-9.584433	-10.074374	10.367152
C	-10.070457	-9.110473	11.252197
N	-5.375606	-9.971565	9.475669
C	-5.834385	-9.127210	10.473530
O	-5.087463	-8.348072	11.081447
H	-6.112410	-10.559442	9.060294
H	10.242920	-10.510208	-7.042994
H	9.097070	-11.591180	-5.108634
H	10.756955	-8.061806	-6.975238
H	6.561661	-7.961781	-0.352743
H	5.029869	-8.398582	1.516764
H	5.705067	-12.627460	1.055295
H	7.163568	-12.184805	-0.893145
H	5.821637	-12.050695	3.722160
H	0.774394	-11.344618	8.685633
H	0.717635	-12.098934	3.495301
H	1.497268	-13.030252	4.792638
H	2.238912	-12.959156	3.179853
H	2.131797	-8.536551	6.683711
H	3.628050	-9.133431	5.934749
H	2.407875	-8.302266	4.945021
H	-2.683621	-11.327428	6.090244
H	-4.849523	-11.174347	7.241319
H	-3.029283	-8.959998	10.445451
H	-0.827763	-9.201411	9.343591
H	-9.603444	-7.454113	12.569384
H	-7.155710	-7.534139	12.096276
H	-11.141395	-9.077344	11.443828
O	-7.939467	-13.422017	7.679010
O	-9.291822	-13.665588	9.929822
C	-7.546946	-16.899935	9.893641
C	-6.814885	-16.773988	8.727711
C	-6.903490	-15.594149	7.938553
C	-7.764173	-14.546233	8.349279

C	-8.520798	-14.679365	9.577220
C	-8.396666	-15.854004	10.320892
C	-5.251310	-13.891959	5.008692
C	-5.200301	-12.506497	4.742037
C	-4.408015	-11.999336	3.722229
C	-3.677589	-12.866580	2.887245
C	-3.752800	-14.248330	3.135845
C	-4.508533	-14.761702	4.184740
C	-2.921967	-12.371676	1.733986
C	-3.040075	-12.846687	0.459344
C	-2.264334	-12.144708	-0.507549
C	-1.176229	-11.262911	0.129389
S	-1.775295	-11.025289	1.898557
C	-2.355115	-12.166442	-1.865067
C	-3.186160	-13.068277	-2.716693
C	-3.093373	-12.438435	-4.136103
C	-1.748874	-11.659063	-4.127431
C	-1.581175	-11.273643	-2.696486
C	-0.858270	-10.246845	-2.173736
C	0.039587	-9.350314	-2.822063
C	0.848585	-8.648975	-1.974549
S	0.535716	-8.963955	-0.253918
C	-1.016715	-9.943541	-0.673360
F	-4.486976	-13.181819	-2.313972
F	-2.690813	-14.349091	-2.777027
F	-4.112196	-11.553623	-4.298315
F	-3.161663	-13.359315	-5.122953
F	-0.762461	-12.508545	-4.573792
F	-1.778908	-10.608979	-4.997053
C	0.120417	-12.107038	0.187573
C	-2.224727	-8.986119	-0.526346
C	1.920345	-7.743734	-2.393628
C	3.225040	-7.850518	-1.874045
C	4.246737	-7.052000	-2.366929
C	3.998955	-6.075487	-3.356316
C	2.690523	-5.954170	-3.867031
C	1.681926	-6.789618	-3.398862
N	-6.049875	-14.306652	6.070731
C	-6.040977	-15.525924	6.729186
O	-5.340193	-16.477394	6.357590
H	-6.680081	-13.611886	6.496225
O	7.704421	-5.214652	-3.620342
O	9.931328	-3.811005	-3.780304

C	7.576087	-1.424572	-5.285758
C	6.397440	-2.139272	-5.180698
C	6.385404	-3.438222	-4.601717
C	7.600095	-4.007123	-4.145211
C	8.829747	-3.248394	-4.245296
C	8.790591	-1.974981	-4.815930
N	5.078505	-5.300358	-3.768892
C	5.064072	-4.108413	-4.475258
O	4.022849	-3.619578	-4.934924
H	6.017933	-5.581027	-3.452615
H	-7.469167	-17.805114	10.492988
H	-6.150601	-17.562988	8.389704
H	-8.969930	-15.946248	11.241582
H	-5.798318	-11.830785	5.344239
H	-4.389972	-10.926859	3.546695
H	-3.191893	-14.930210	2.502452
H	-4.532887	-15.824391	4.377471
H	-3.754498	-13.617638	0.193800
H	0.159587	-9.314572	-3.898655
H	0.905266	-11.599648	0.752827
H	0.487252	-12.297972	-0.826972
H	-0.090993	-13.067332	0.666453
H	-2.098599	-8.133972	-1.200132
H	-3.149497	-9.508148	-0.796018
H	-2.320213	-8.612713	0.495591
H	3.451428	-8.591021	-1.111429
H	5.257533	-7.181168	-1.994048
H	2.483133	-5.208632	-4.620862
H	0.679652	-6.691468	-3.806915
H	7.574673	-0.429688	-5.727190
H	5.454257	-1.727561	-5.525663
H	9.719541	-1.412497	-4.889675

****

ΔΛ_open_conformer_1_DMF

Ga	11.787561	0.325795	0.063995
Ga	-12.063571	0.444330	0.508489
C	11.224510	0.518227	5.182837
C	9.975131	1.018337	4.863861
C	9.624714	1.284976	3.512523
C	10.581344	1.048465	2.491952
C	11.872624	0.484110	2.825934

C	12.170724	0.244366	4.167171
C	6.720780	2.223985	1.296619
C	6.707378	2.225426	-0.114483
C	5.545670	2.496159	-0.819582
C	4.337359	2.774249	-0.149588
C	4.359225	2.782257	1.256651
C	5.521837	2.514392	1.977626
C	3.112185	3.005005	-0.921394
C	2.774467	2.469366	-2.137368
C	1.475842	2.864275	-2.610170
C	0.824335	3.729724	-1.753088
S	1.825549	4.069510	-0.365205
C	0.933803	2.364399	-3.875748
C	1.812593	2.389118	-5.097766
C	0.891997	1.997469	-6.283731
C	-0.381240	1.434884	-5.601540
C	-0.288754	1.850280	-4.156483
C	-1.419735	1.573136	-3.266107
C	-2.761048	1.987416	-3.586089
C	-3.686740	1.628424	-2.642376
S	-2.911927	0.734392	-1.351510
C	-1.334117	0.887730	-2.066001
F	2.850845	1.495424	-5.012672
F	2.379071	3.611128	-5.299969
F	1.473765	1.090251	-7.100693
F	0.580875	3.096123	-7.012497
F	-1.501875	1.884421	-6.235946
F	-0.399405	0.065871	-5.711534
C	-0.531460	4.352433	-1.878572
C	-0.142569	0.336084	-1.346526
C	-5.105761	1.976664	-2.518681
C	-5.564244	3.257447	-2.873205
C	-6.875387	3.659214	-2.623913
C	-7.769442	2.776593	-1.990487
C	-7.332300	1.469848	-1.687074
C	-6.026218	1.080957	-1.940323
C	-10.780359	4.309234	-0.401106
C	-11.403306	5.542287	-0.068937
C	-12.492143	5.573106	0.784382
C	-13.007777	4.377952	1.338967
C	-12.429940	3.141908	1.043380
C	-11.285490	3.110551	0.159251
O	10.377199	1.291682	1.212221

O	12.702228	0.225456	1.823655
O	10.544049	0.726137	-1.513907
O	12.899019	-0.990473	-0.952080
O	12.716698	1.938125	-0.631483
O	10.643516	-1.364495	0.330165
O	-12.856384	1.983151	1.519544
O	-10.777512	1.912710	-0.070375
O	-13.158380	-0.949041	1.362050
O	-11.096254	-0.722133	-0.895773
O	-10.648811	-0.261860	1.840213
O	-13.287359	0.734440	-1.055598
C	11.726539	4.253586	-3.315471
C	10.562475	3.647679	-3.753053
C	10.104024	2.443568	-3.154239
C	10.864865	1.856108	-2.112880
C	12.067894	2.506838	-1.638172
C	12.475857	3.689241	-2.256806
C	7.130154	0.135121	-3.013867
C	6.767196	-0.677074	-1.918844
C	5.529845	-1.297557	-1.870028
C	4.603332	-1.165793	-2.925733
C	4.992343	-0.390528	-4.032491
C	6.222739	0.261990	-4.080931
C	3.292382	-1.816243	-2.837304
C	2.894901	-2.801750	-1.970612
C	1.519375	-3.194513	-2.109943
C	0.860667	-2.500578	-3.111248
S	1.942119	-1.370823	-3.876870
C	0.974123	-4.325159	-1.361415
C	1.895582	-5.491038	-1.079409
C	0.964863	-6.651933	-0.654702
C	-0.325932	-5.928889	-0.209890
C	-0.244351	-4.552691	-0.816299
C	-1.378914	-3.657033	-0.527614
C	-2.674528	-3.745415	-1.134123
C	-3.625420	-2.969421	-0.515509
S	-2.921166	-2.138379	0.862498
C	-1.347285	-2.821354	0.571129
F	2.752373	-5.199013	-0.041879
F	2.672559	-5.842274	-2.137046
F	1.500161	-7.413922	0.325179
F	0.709806	-7.441732	-1.727215
F	-1.439244	-6.609787	-0.610590

F	-0.377068	-5.869476	1.157705
C	-0.556171	-2.612873	-3.580228
C	-0.216127	-2.568063	1.516777
C	-5.011041	-2.711594	-0.915799
C	-5.381803	-2.754575	-2.272225
C	-6.675693	-2.457949	-2.690428
C	-7.655022	-2.080699	-1.749233
C	-7.296739	-2.057206	-0.384774
C	-6.006131	-2.366551	0.020002
C	-10.828346	-0.777369	-3.302102
C	-11.395584	-0.437512	-4.561144
C	-12.584154	0.264998	-4.632848
C	-13.246998	0.671361	-3.452117
C	-12.729766	0.360617	-2.192946
C	-11.507996	-0.414876	-2.112762
C	-12.177521	-3.708466	3.574301
C	-10.858895	-3.374269	3.814555
C	-10.281105	-2.209328	3.236403
C	-11.073864	-1.374441	2.410848
C	-12.452858	-1.741348	2.154316
C	-12.975349	-2.892537	2.741721
C	-6.936978	-0.431554	3.098585
C	-6.117554	-0.820023	4.177983
C	-4.802917	-0.378267	4.260443
C	-4.241462	0.474580	3.292741
C	-5.069749	0.872452	2.224318
C	-6.388287	0.432721	2.128968
C	-2.831650	0.866870	3.420815
C	-2.089790	1.005323	4.570057
C	-0.694481	1.241623	4.335533
C	-0.373139	1.311971	2.992077
S	-1.793826	1.089098	2.018912
C	0.307516	1.448397	5.382540
C	0.120246	2.528867	6.410402
C	1.371023	2.440470	7.325870
C	2.370216	1.575211	6.514019
C	1.523906	0.863071	5.496636
C	2.057044	-0.292314	4.777362
C	3.354852	-0.303999	4.166435
C	3.664892	-1.490044	3.546226
S	2.351220	-2.636757	3.771117
C	1.375350	-1.487485	4.641664
F	-1.028098	2.366421	7.135386

F	0.046691	3.768740	5.840504
F	1.056535	1.804067	8.481359
F	1.871606	3.659250	7.628747
F	3.296215	2.387292	5.906321
F	3.065333	0.731875	7.324634
C	0.955672	1.549085	2.346396
C	0.012287	-1.860439	5.136853
C	4.865616	-1.838727	2.784822
C	4.895337	-2.908802	1.870323
C	6.039887	-3.225080	1.139781
C	7.204795	-2.450311	1.295042
C	7.183184	-1.382504	2.216470
C	6.045608	-1.082916	2.944918
C	10.075232	-3.323319	-0.978941
C	10.504230	-4.229369	-1.985570
C	11.726401	-4.060411	-2.611817
C	12.562178	-2.969804	-2.275288
C	12.181943	-2.049707	-1.297341
C	10.923564	-2.248668	-0.610489
N	8.372268	0.764446	-2.950508
N	7.937975	1.926523	1.904746
N	8.394331	-2.627495	0.598225
C	8.724537	-3.536954	-0.386042
C	8.234368	1.747885	3.240657
C	8.805110	1.889584	-3.629815
O	7.399657	1.922583	4.143535
O	8.146874	2.414841	-4.538779
O	7.961845	-4.443565	-0.749753
H	8.733788	1.719764	1.281121
H	9.018288	0.461704	-2.205940
H	9.131558	-1.921358	0.758117
C	-8.851269	-1.959989	3.557890
N	-8.264991	-0.857047	2.954085
N	-8.952396	-1.686235	-2.070182
C	-9.500915	-1.449127	-3.318448
N	-9.067169	3.101461	-1.599871
C	-9.576218	4.349158	-1.278688
O	-9.063004	5.405341	-1.671160
O	-8.918297	-1.743529	-4.372151
O	-8.216365	-2.708991	4.313735
H	-9.562247	-1.385070	-1.294344
H	-8.883073	-0.344310	2.314114
H	-9.596551	2.336007	-1.154057

H	11.484037	0.319558	6.220899
H	9.230409	1.209268	5.630281
H	13.146225	-0.168783	4.417317
H	7.623473	2.006221	-0.656246
H	5.574599	2.493188	-1.905189
H	3.444138	2.979755	1.808519
H	5.512701	2.520297	3.058511
H	3.408813	1.765946	-2.662472
H	-3.012570	2.551179	-4.475555
H	-0.545813	5.371242	-1.476248
H	-0.835278	4.394473	-2.928355
H	-1.287336	3.767881	-1.338262
H	0.714787	0.259574	-2.014426
H	0.148792	0.992562	-0.517241
H	-0.344550	-0.656741	-0.931601
H	-4.867845	3.966287	-3.313701
H	-7.203791	4.660246	-2.868311
H	-8.023828	0.770714	-1.225495
H	-5.715891	0.074664	-1.675292
H	-10.995340	6.452286	-0.498469
H	-12.962952	6.522377	1.033358
H	-13.867642	4.403800	2.006090
H	12.070017	5.176061	-3.779805
H	9.967264	4.078293	-4.552365
H	13.384439	4.173080	-1.902764
H	7.452947	-0.782751	-1.083611
H	5.270626	-1.867008	-0.984483
H	4.330189	-0.292048	-4.886189
H	6.486391	0.870746	-4.934224
H	3.561095	-3.249943	-1.244505
H	-2.881972	-4.362351	-2.000914
H	-0.659283	-2.314546	-4.627758
H	-0.912046	-3.642748	-3.475521
H	-1.220608	-1.973161	-2.986576
H	-0.114608	-1.504204	1.756587
H	0.726743	-2.910941	1.084968
H	-0.368330	-3.105747	2.459705
H	-4.631188	-2.994008	-3.020656
H	-6.932687	-2.475963	-3.739583
H	-8.038787	-1.778114	0.358739
H	-5.772317	-2.337165	1.080563
H	-10.859169	-0.733640	-5.457061
H	-13.010550	0.520712	-5.601216

H	-14.170686	1.245167	-3.504077
H	-12.606949	-4.602563	4.022122
H	-10.228278	-3.990885	4.446323
H	-14.013712	-3.149298	2.540093
H	-6.510758	-1.489005	4.928277
H	-4.187709	-0.724056	5.086353
H	-4.684675	1.536913	1.454565
H	-6.998080	0.747406	1.285764
H	-2.514745	0.923121	5.563399
H	4.001434	0.565410	4.149116
H	1.623465	2.078412	3.032031
H	0.856451	2.148763	1.438439
H	1.443988	0.605804	2.071685
H	-0.757143	-1.648825	4.384045
H	-0.049731	-2.924583	5.387995
H	-0.237961	-1.281893	6.031721
H	4.001532	-3.504078	1.696959
H	6.036425	-4.046758	0.436755
H	8.080604	-0.787400	2.352092
H	6.081569	-0.254687	3.645765
H	9.842099	-5.048539	-2.249162
H	12.048165	-4.762349	-3.378948
H	13.514054	-2.824932	-2.783301

****

ΔΛ_closed_conformer_1_DMF

Ga	-9.315987	-12.022985	8.830848
O	-9.178812	-10.507334	7.456275
O	-11.136853	-12.179281	8.038485
C	-12.651314	-10.391763	5.205621
C	-11.617775	-9.536861	4.870227
C	-10.399087	-9.550980	5.602943
C	-10.254807	-10.439573	6.695391
C	-11.332898	-11.346654	7.030500
C	-12.509062	-11.299116	6.280493
C	-6.905866	-8.128914	5.521195
C	-5.717531	-8.684269	6.045500
C	-4.478419	-8.110233	5.786470
C	-4.387615	-6.918615	5.044151
C	-5.578022	-6.350002	4.553390
C	-6.815453	-6.942919	4.766313
C	-3.131965	-6.208070	4.785875

C	-2.958373	-4.870358	4.994893
C	-1.722123	-4.348790	4.521547
C	-1.021991	-5.315916	3.546138
S	-1.716210	-6.999854	4.038628
C	-1.169728	-3.123098	4.721251
C	-1.766084	-1.947978	5.423759
C	-0.626970	-0.885975	5.418016
C	0.270316	-1.278147	4.209694
C	0.078268	-2.752497	4.100355
C	0.853890	-3.663415	3.451502
C	1.919729	-3.424241	2.542693
C	2.305565	-4.508027	1.809242
S	1.477914	-6.014166	2.263420
C	0.521530	-5.156493	3.644976
F	-2.186708	-2.208175	6.696958
F	-2.852229	-1.430064	4.760884
F	0.099322	-1.005007	6.560058
F	-1.095612	0.378713	5.334905
F	-0.175399	-0.592276	3.102729
F	1.563222	-0.892057	4.406528
C	-1.571037	-4.997468	2.129811
C	1.104723	-5.626261	4.994101
C	3.179264	-4.427969	0.633211
C	4.273539	-5.278675	0.393207
C	5.066462	-5.102629	-0.735810
C	4.761363	-4.115230	-1.698919
C	3.660785	-3.268248	-1.463835
C	2.902124	-3.421734	-0.311767
N	-8.105047	-8.782525	5.792725
C	-9.324008	-8.631567	5.147941
O	-9.496869	-7.812843	4.234589
H	-8.109295	-9.507133	6.525098
Ga	9.725204	-5.710763	-3.865015
O	7.949108	-4.706862	-3.710921
O	9.606787	-5.043676	-5.736193
C	6.921424	-3.179137	-7.417720
C	6.031094	-3.028156	-6.370767
C	6.332450	-3.549451	-5.082573
C	7.563778	-4.214715	-4.873001
C	8.485059	-4.386592	-5.976927
C	8.145121	-3.860343	-7.224056
N	5.573885	-4.026918	-2.826648
C	5.302781	-3.380967	-4.024292

O	4.262433	-2.735375	-4.212756
H	6.471767	-4.531733	-2.821215
H	-13.579881	-10.375888	4.638078
H	-11.705271	-8.842285	4.040811
H	-13.316541	-11.981117	6.540771
H	-5.777831	-9.574531	6.663233
H	-3.582670	-8.572927	6.192684
H	-5.523886	-5.434483	3.971203
H	-7.712467	-6.506263	4.352690
H	-3.701660	-4.260763	5.497002
H	2.319641	-2.432496	2.362933
H	-1.216259	-5.721324	1.393646
H	-1.252341	-3.997007	1.818494
H	-2.663777	-5.020962	2.146836
H	2.168681	-5.377686	5.040098
H	0.593563	-5.126586	5.823537
H	0.993816	-6.706670	5.120083
H	4.525796	-6.061906	1.103363
H	5.936725	-5.734380	-0.883689
H	3.408804	-2.510177	-2.190460
H	2.052826	-2.764514	-0.149356
H	6.681825	-2.778847	-8.401084
H	5.082662	-2.517825	-6.504587
H	8.843954	-3.990174	-8.048502
O	9.592184	-6.047080	-1.847429
O	10.937618	-4.256687	-3.245112
C	12.039068	-3.372160	0.149203
C	11.323948	-4.294103	0.891001
C	10.461263	-5.226148	0.251334
C	10.347538	-5.212608	-1.159518
C	11.092839	-4.240205	-1.932170
C	11.923478	-3.342530	-1.259688
C	7.844635	-7.826032	1.040391
C	6.742194	-8.227664	0.254155
C	5.753525	-9.058351	0.769618
C	5.863020	-9.569900	2.075808
C	6.985843	-9.199195	2.840612
C	7.955185	-8.335836	2.348822
C	4.881160	-10.464932	2.696091
C	5.207292	-11.568079	3.430411
C	4.133177	-12.139057	4.164771
C	2.747857	-11.585587	3.774961
S	3.147585	-10.072277	2.723029

C	4.189796	-13.048586	5.175950
C	5.358929	-13.845519	5.645259
C	4.705538	-14.998674	6.458788
C	3.377530	-14.393056	7.001728
C	3.061862	-13.293512	6.041485
C	1.961390	-12.496647	6.012716
C	0.788517	-12.544220	6.818075
C	-0.222852	-11.727723	6.402709
S	0.145038	-10.853013	4.889818
C	1.975975	-11.265020	5.085800
F	6.188584	-13.141479	6.488505
F	6.149369	-14.330364	4.645338
F	5.505377	-15.465522	7.442880
F	4.416072	-16.021987	5.613012
F	2.406498	-15.348550	7.099611
F	3.594564	-13.935515	8.278607
C	2.034411	-12.617798	2.876226
C	2.616922	-10.106124	5.894705
C	-1.452550	-11.493757	7.166883
C	-2.746491	-11.624381	6.630858
C	-3.862873	-11.451210	7.440653
C	-3.733481	-11.076689	8.796501
C	-2.438327	-10.934341	9.331899
C	-1.328046	-11.158602	8.527931
N	8.768553	-6.952173	0.472900
C	9.699572	-6.156323	1.125123
O	9.863775	-6.193901	2.352371
H	8.736028	-6.802865	-0.545868
O	-7.469542	-11.424297	9.495738
O	-9.905156	-10.949184	10.398203
C	-7.936875	-9.046488	12.851696
C	-6.659565	-9.267120	12.370795
C	-6.448649	-10.064742	11.212672
C	-7.560648	-10.648465	10.559008
C	-8.897864	-10.403400	11.058758
C	-9.055628	-9.610667	12.196485
N	-4.900533	-10.880168	9.530775
C	-5.047666	-10.214926	10.738966
O	-4.084819	-9.749792	11.364375
H	-5.790015	-11.203884	9.124506
H	12.694407	-2.660041	0.647271
H	11.395615	-4.326449	1.973503
H	12.480794	-2.614581	-1.846497

H	6.667555	-7.876291	-0.769924
H	4.908376	-9.331150	0.142949
H	7.081478	-9.574084	3.855466
H	8.789613	-8.038200	2.966481
H	6.229174	-11.917180	3.527830
H	0.694979	-13.187679	7.686108
H	1.096093	-12.219482	2.480771
H	1.812347	-13.527169	3.444310
H	2.682031	-12.881943	2.035551
H	2.100920	-9.999148	6.852460
H	3.671681	-10.323923	6.093390
H	2.552176	-9.160249	5.353785
H	-2.882634	-11.895659	5.587147
H	-4.854469	-11.606365	7.027940
H	-2.319558	-10.645852	10.365858
H	-0.335093	-11.048927	8.954745
H	-8.089071	-8.432033	13.737071
H	-5.790550	-8.834096	12.855885
H	-10.063542	-9.432663	12.567078
O	-8.314387	-13.177134	7.461553
O	-9.412150	-13.766408	9.790212
C	-8.093025	-17.068253	8.855701
C	-7.483642	-16.761657	7.653292
C	-7.517019	-15.435162	7.141636
C	-8.198516	-14.427532	7.866028
C	-8.818973	-14.749514	9.134753
C	-8.756327	-16.064486	9.598394
C	-6.012686	-13.302219	4.418496
C	-5.794112	-11.906258	4.418620
C	-5.055636	-11.292447	3.413826
C	-4.560278	-12.044883	2.332673
C	-4.812989	-13.429810	2.318360
C	-5.509042	-14.058833	3.341843
C	-3.832866	-11.468133	1.199442
C	-4.110271	-11.752864	-0.106909
C	-3.184744	-11.216121	-1.043831
C	-1.875691	-10.758960	-0.369461
S	-2.407966	-10.411561	1.408239
C	-3.263363	-11.166339	-2.400044
C	-4.310311	-11.762524	-3.282282
C	-3.908886	-11.297009	-4.713682
C	-2.383068	-11.009501	-4.622229
C	-2.186701	-10.628649	-3.194324

C	-1.136668	-9.973201	-2.628565
C	0.121085	-9.660986	-3.211387
C	1.090914	-9.288109	-2.327102
S	0.513816	-9.143375	-0.651202
C	-1.258524	-9.561463	-1.147654
F	-5.585581	-11.375135	-2.981915
F	-4.311034	-13.135783	-3.249670
F	-4.556206	-10.138144	-5.003323
F	-4.214006	-12.209927	-5.662168
F	-1.707253	-12.157152	-4.970036
F	-2.004966	-10.053943	-5.518677
C	-0.939623	-11.995885	-0.326743
C	-2.097975	-8.269960	-1.054930
C	2.513570	-9.190197	-2.675416
C	3.351341	-8.115764	-2.324591
C	4.680111	-8.094885	-2.737073
C	5.240484	-9.170813	-3.460712
C	4.404062	-10.250450	-3.806320
C	3.068778	-10.242842	-3.427982
N	-6.722443	-13.850244	5.483532
C	-6.789324	-15.181102	5.870908
O	-6.251240	-16.090402	5.224341
H	-7.211970	-13.207828	6.123208
O	8.592637	-7.412999	-4.080561
O	11.197834	-6.993192	-4.248094
C	11.110232	-10.636436	-4.726601
C	9.753610	-10.870843	-4.603087
C	8.851336	-9.798061	-4.362255
C	9.349914	-8.476594	-4.267894
C	10.775148	-8.239033	-4.378207
C	11.622271	-9.323730	-4.610008
N	6.590687	-9.104886	-3.799854
C	7.417609	-10.148501	-4.192153
O	6.998915	-11.302954	-4.354981
H	7.077883	-8.203342	-3.699348
H	-8.060536	-18.085530	9.241324
H	-6.959891	-17.517022	7.076258
H	-9.227785	-16.300394	10.550742
H	-6.212828	-11.305506	5.219838
H	-4.892899	-10.218504	3.454037
H	-4.430053	-14.027214	1.495860
H	-5.662497	-15.127674	3.326530
H	-4.980670	-12.328245	-0.402831

H	0.328104	-9.814012	-4.264615
H	-0.034163	-11.789762	0.247177
H	-0.651360	-12.285404	-1.342906
H	-1.463984	-12.836454	0.135337
H	-1.674160	-7.507927	-1.714911
H	-3.130140	-8.464171	-1.364672
H	-2.111450	-7.877883	-0.034324
H	2.958533	-7.278765	-1.753225
H	5.301741	-7.237710	-2.498846
H	4.813500	-11.085377	-4.355006
H	2.441506	-11.087636	-3.697248
H	11.793962	-11.463710	-4.907560
H	9.344297	-11.873250	-4.677078
H	12.691078	-9.135979	-4.695496

****

7. References

(1) Neufeld, R.; Stalke, D. Accurate molecular weight determination of small molecules via DOSY-NMR by using external calibration curves with normalized diffusion coefficients. *Chem. Sci.* **2015**, *6*, 3354-3364.

(2) Bachmann, S.; Neufeld, R.; Dzemski, M.; Stalke, D. New External Calibration Curves (ECCs) for the Estimation of Molecular Weights in Various Common NMR Solvents. *Chem. Eur. J.* **2016**, *22*, 8462-8465.

(3) Kreyenschmidt, A.-K.; Bachmann, S.; Niklas, T.; Stalke, D. Molecular Weight Estimation of Molecules Incorporating Heavier Elements from van-der-Waals Corrected ECC-DOSY. *ChemistrySelect* **2017**, *2*, 6957-6960.

(4) Edward, J. T. Molecular Volumes and the Stokes-Einstein equation. *J. Chem. Educ.* **1970**, *47*, 2178-2191.

(5) Haynes, W. M. *CRC Handbook of Chemistry and Physics*; CRC Press, 2014.

(6) Gajda, R.; Katrusiak, A. The Interplay of Molecular Conformation and Crystal Packing in Pressure-Frozen Tetramethylsilane. *Cryst. Growth Des.* **2008**, *8*, 211-214.

(7) Donohue, J.; Goodman, S. H. The crystal structure of adamantane: an example of a false minimum in least squares. *Acta Crystallogr.* **1967**, *22*, 352-354.

(8) Mercier, A.; Yeo, W. C.; Chou, J.; Chaudhuri, P. D.; Bernardinelli, G.; Kündig, E. P. Synthesis of highly enantiomerically enriched planar chiral ruthenium complexes via Pd-catalysed asymmetric hydrogenolysis. *Chem. Commun.* **2009**, 5227-5229.

(9) Ojala, W. H.; Gustafson, H. L.; Ojala, C. R. A polymorph of 1,2,3,4-tetraphenylnaphthalene. *Acta Crystallogr. C* **1994**, *50*, 1602-1604.

(10) Alvarez, S. A cartography of the van der Waals territories. *Dalton Trans.* **2013**, *42*, 8617-8636.

(11) Virk, A. S.; Torres, A. M.; Willis, S. A.; Price, W. S. NMR diffusion studies of spherical molecules: Tetramethylsilane and buckyballs. *J. Mol. Liq.* **2016**, *214*, 157-161.

(12) Han, M.; Engelhard, D. M.; Clever, G. H. Self-assembled coordination cages based on banana-shaped ligands. *Chem. Soc. Rev.* **2014**, *43*, 1848-1860.
